# Personalized Nutrition Using Microbial Metabolite Phenotype to Stratify Participants and Non-Invasive Host Exfoliomics Reveal the Effects of Flaxseed Lignan Supplementation in a Placebo-Controlled Crossover Trial

**DOI:** 10.3390/nu14122377

**Published:** 2022-06-08

**Authors:** Destiny A. Mullens, Ivan Ivanov, Meredith A. J. Hullar, Timothy W. Randolph, Johanna W. Lampe, Robert S. Chapkin

**Affiliations:** 1 Department of Veterinary Physiology and Pharmacology, Texas A&M University, College Station, TX 77843, USA; destiny_mullens@tamu.edu (D.A.M.); iivanov@cvm.tamu.edu (I.I.); 2 Program in Integrative Nutrition & Complex Diseases, Texas A&M University, College Station, TX 77843, USA; 3 Public Health Sciences Division, Fred Hutchinson Cancer Center, Seattle, WA 98109, USA; mhullar@fredhutch.org (M.A.J.H.); jlampe@fredhutch.org (J.W.L.); 4 Clinical Research Division, Fred Hutchinson Cancer Center, Seattle, WA 98109, USA; trandolp@fredhutch.org; 5 School of Public Health, University of Washington, Seattle, WA 98195, USA; 6 Department of Nutrition, Texas A&M University, College Station, TX 77843, USA

**Keywords:** human intervention, colon, lignan, enterolactone, secoisolariciresinol, gene expression, exfoliome

## Abstract

High-fiber plant foods contain lignans that are converted to bioactive enterolignans, enterolactone (ENL) and enterodiol (END) by gut bacteria. Previously, we conducted an intervention study to gain mechanistic insight into the potential chemoprotective effects of flaxseed lignan supplementation (secoisolariciresinol diglucoside; SDG) compared to a placebo in 42 men and women. Here, we expand on these analyses to further probe the impact of the microbial metabolite phenotype on host gene expression in response to lignan exposure. We defined metabolic phenotypes as high- or low-ENL excretion based on the microbial metabolism of SDG. RNA-seq was used to assess host gene expression in fecal exfoliated cells. Stratified by microbial ENL excretion, differentially expressed (DE) genes in high- and low-ENL excreter groups were compared. Linear discriminant analysis using the ENL phenotypes identified putative biomarker combinations of genes capable of discriminating the lignan treatment from the placebo. Following lignan intervention, a total of 165 DE genes in high-ENL excreters and 1450 DE genes in low-ENL excreters were detected. Functional analysis identified four common upstream regulators (master genes): CD3, IFNG, IGF1 and TNFRSF1A. Our findings suggest that the enhanced conversion of flaxseed lignan to ENL is associated with a suppressed inflammatory status.

## 1. Introduction

Colorectal cancer (CRC) is the third most commonly occurring cancer worldwide and is second in mortality [1] with an estimated 1.9 million new cases and 935,000 deaths in 2020. Aspects of diet are important risk factors for CRC [1]. Higher intake of dietary fiber-rich foods is associated with lower CRC risk [2,3,4]. While dietary fiber itself is strongly linked to reduction of CRC [5], high-fiber foods also contain lignans that are converted to bioactive enterolignans, enterodiol (END) and enterolactone (ENL) by gut bacteria. Higher lignan exposure has also been associated with lower CRC risk in humans and reduced colon tumorigenesis in animal models [6]. In humans, differences in ENL concentrations in blood and urine reflect substantial heterogeneity in gut microbial metabolism and the capacity to convert plant lignans to ENL, suggesting a possible microbial metabolic phenotype [7]. In observational studies, it has been difficult to ascertain whether ENL is an active mediator in disease etiology or a marker of a healthier diet [8]; however, we have shown experimentally in a controlled feeding study that the capacity to produce ENL in response to low and high glycemic load diets results in differences in plasma proteome and metabolome pathways [9]. Our findings suggest that better characterization of ENL phenotypes and how they impact host response may improve precision in optimizing diets for health.

Previously, we conducted a controlled dietary intervention in healthy men and women to examine the effects of a flaxseed lignan extract on gene expression in colonic epithelium. Differentially expressed (DE) genes in fecal exfoliated colonocyte RNA (exfoliome) were detected and there was a significant difference in urinary enterolignan excretion in response to the lignan intervention [6]. Differential gene expression was also seen between the two different phenotypes (high and low urinary ENL excretion) without respect to the intervention. The primary goal of this paper is to determine whether the conversion of flaxseed lignan to ENL in adult humans is associated with a protective anti-inflammatory phenotype. Thus, we extended the previous work and more formally evaluated the effects of the flaxseed lignan supplement on gene expression in stool exfoliated cells in the context of the high or low urinary ENL excretion phenotype. As a result of this effort, by stratifying participants using their microbial metabolite phenotype, we noninvasively identified a novel host gene expression signature that distinguishes host responses to flaxseed lignan extract between the two ENL excretion phenotypes.

## 2. Materials and Methods

### 2.1. Data Source

The details of the study design and data collection procedures are described in Lampe et al. [6]. Briefly, 42 healthy men and women participated in a 2-period randomized, crossover intervention comparing a supplemental flaxseed lignan extract (50 mg/d secoisolariciresinol diglucoside; Barlene’s Organic Oils) with a visually identical placebo. Each intervention period lasted 60 days with a 60-day washout period between interventions. Participants completed stool and 24-h urine samples at the end of each period. For stool exfoliome samples, approximately 46 million 50-bp single-end reads were sequenced using standard Illumina protocols on an Illumina HiSeq 2500 platform at Texas A&M. Urinary lignans (secoisolariciresinol, END, ENL) were measured by gas chromatography–mass spectrometry. The observed ENL excretion values at the end of the lignan extract period were used to identify the participants with two distinct levels of ENL excretion. High and low urinary ENL excretion phenotypes were defined as the 21 participants above or the 21 below the median ENL excretion after the lignan extract intervention period (23.4 µmol/24 h). Host fecal exfoliome samples available for DE analysis after collection, sequencing and quality control consisted of: (i) the group of high-ENL participants: 14 lignan supplement and 13 placebo samples; and (ii) the group of low-ENL participants: 15 lignan supplement and 12 placebo samples (Table S1).

### 2.2. Differential Gene Expression Analysis

The study was designed for paired treatment analysis with each participant being their own control; however, 14 participants lacked either a lignan or placebo sample after collection, sequencing and quality control (Table S1). Thus, an unpaired analysis approach was utilized. Outlines of the data pipeline for pre-processing, normalization and analyses are described in Figure S1. An iterative leave-one-out approach was used to detect genes exhibiting extreme (outlier) counts [10]. The effect of any outliers was mitigated by winsorzing the top and bottom 5% of values to the 5th and 95th percentiles, respectively. RNA-seq data were normalized using the upper-quartile method with edgeR [11]. DE genes were identified using edgeR-robust [12] in several contrasts. For comparison, lignan supplement vs. placebo and high-ENL vs. low-ENL excreter contrasts were performed, along with intervention contrasts (lignan supplement vs. placebo) in each of the ENL excretion phenotypes. To account for multiple testing, the Benjamini-Hochberg (BH) [13] false discovery rate (FDR) procedure was utilized and genes were considered to be DE if the corresponding corrected *p*-values were less than 0.05. 

### 2.3. Linear Discriminant Analysis (LDA)

Given that genes interact within a complex regulatory system via their gene products, it is expected that combinations of several genes could better discriminate phenotypes than each one of those genes individually [14]. Therefore, we utilized a classification approach based on LDA to identify multivariate discriminators (based on sets of genes) between the placebo and lignan intervention, in the context of the ENL phenotypes (low- and high-ENL excreters). For the purpose of LDA classification, we used a list of 840 a priori identified genes that relate to the gene-regulatory processes in the human gut (Table S2) and were also detected in the exfoliome samples [6]. Bolstered error estimation (bresub) was used to estimate the LDA classification error. Bresub has been used to mitigate problems associated with classification error estimation when the sample size is limited because error estimators often have large variance and could be biased [15], and many gene sets may have optimistically low re-substitution error estimates [16]. In addition, rather than only counting incorrectly classified points, bresub places a Gaussian kernel at each data point giving more weight to the points near the classification boundary [15,16]. This approach provides a list of highly ranked gene sets with respect to bresub estimates of the classifiers based on those gene sets [14]. 

Finally, a novel LDA-derived method to build a frequency signature of the two ENL excretion phenotypes was applied. For the purpose of identifying phenotype-defining gene signatures, we considered the top 500 performing (with respect to their bresub error) three-gene LDA classifiers of lignan vs. placebo intervention in the context of each phenotype. This frequency signature represents, in a condensed format, the importance of a particular gene in the context of discrimination between the two types of dietary supplementation (lignan or placebo) for each phenotype. 

### 2.4. Ingenuity Pathway Analysis

To compare the different pathways and upstream regulators of each group, five different sets of genes were used to generate enriched gene regulatory pathways using the Ingenuity Pathway Analysis (IPA) software [17]: (1) 165 DE genes for the high-ENL group; (2) 1450 DE genes for the low-ENL group; (3) 28 DE genes common for both the low and high-ENL groups; (4) genes in the top ten three-gene LDA classifiers from the high-ENL group; and (5) genes in the top ten three-gene LDA classifiers from the low-ENL group.

Relevant data sets containing gene identifiers and corresponding measurements were uploaded into the application. Each gene identifier was mapped onto its corresponding gene regulatory pathway in Ingenuity’s Knowledge Base. A threshold of 0.05 for the BH FDR adjusted *p*-value was set to identify molecules whose expression was significantly perturbed. These molecules, called Network Eligible molecules, were overlaid onto a global molecular network developed from information contained in the Ingenuity Knowledge Base. Networks of Network Eligible molecules were then generated based on their connectivity [17]. IPA Upstream Regulator Analysis was used with relevant datasets to identify upstream regulators (master genes) that may be responsible for the observed gene expression changes. IPA uses a z-score algorithm to make predictions designed to reduce the chance that random data generates significant predictions [17]. Typically, predictions of activation are made only if the respective z-score is ≥2. Predictions of inhibition are only made if the respective z-score ≤ −2.

## 3. Results

Urinary ENL values measured in the post-lignan period (mean ± SD) in the high- and low-ENL phenotype groups were 61.75 ± 36.42 and 7.46 ± 6.97 µmol/24 h, respectively. Urinary ENL excretion (24 h) was also statistically significantly higher in the high-ENL excreters compared to the low-ENL excreters at the end of the placebo intervention (Table 1).

### 3.1. Gene Expression in Stool Exfoliated Cells

**Figure 1:**
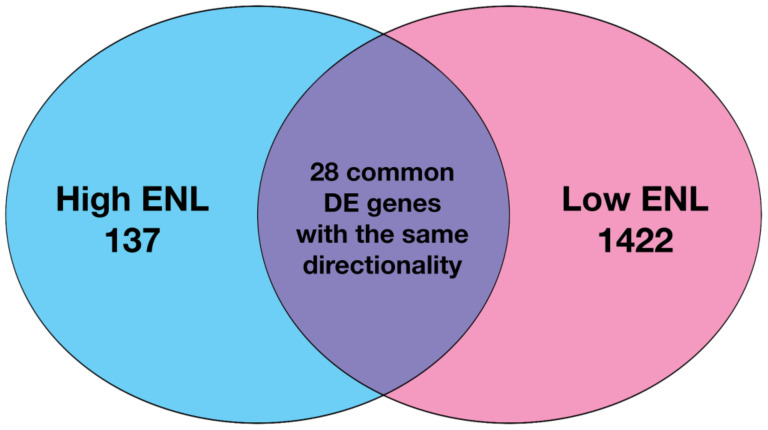
Venn diagram of differentially expressed genes following flaxseed lignan extract supplementation in participants exhibiting high and low ENL phenotypes.

Data preprocessing and filtering resulted in the identification of 11,557 genes from the exfoliated epithelial cell samples that were subsequently used to test for differential gene expression. Testing for intervention effects in the exfoliated samples based on an adjusted *p* < 0.05 identified 165 DE genes (71 downregulated and 94 upregulated) in high-ENL excreters (Table S3) and 1450 DE genes (1140 downregulated and 310 upregulated) in low-ENL excreters (Table S4). There were 28 genes in common between the high- and low-ENL DE genes with the same fold-change directionality (Figure 1); these are identified in Tables S3 and S4.

### 3.2. LDA Classification

Our complementary LDA classification focused on finding sets of genes that could discriminate between the flaxseed lignan extract and placebo intervention diets. We only considered classifiers based on one gene, two genes, or three genes. This restriction avoids the potential peaking phenomenon [16], which manifests itself in the increase of the expected classification rate if larger and larger sets of genes are used to build the respective classifiers. 

Without regard to the ENL phenotype, (i) the top 500 single-gene classifiers exhibited misclassification errors of 0.4650 or less; (ii) the top 500 two-gene LDA classifiers had misclassification errors of 0.2556 or less; and (iii) the top 500 three-gene LDA classifiers had misclassification errors of 0.223 or less when discriminating between lignan and placebo intervention (Tables S5–S7). 

Within the high-ENL excretion group (Table S1), (i) the top 500 single-gene classifiers exhibited misclassification errors of 0.4562 or less; (ii) the top two-gene LDA classifiers had misclassification errors of 0.2272 or less; and (iii) the top 500 three-gene LDA classifiers had misclassification errors of 0.1675 or less when discriminating between lignan and placebo intervention (Tables S8–S10). 

Within the low-ENL excretion group (Table S1), (i) the top 500 single-gene classifiers exhibited misclassification errors of 0.4562 or less; (ii) the top 500 two-gene LDA classifiers had misclassification errors of 0.2208 or less; and (iii) the top 500 three-gene LDA classifiers had misclassification errors of 0.1770 or less when discriminating between lignan and placebo intervention (Tables S11–S13).

The top 10 performing one-, two- and three-gene classifiers that discriminated lignan from placebo intervention in the context of either high- or low-ENL phenotypes are listed in Table 2; Table 3, respectively. Interestingly, both prostaglandin I_2_ receptor (PTGIR) and matrix metallopeptidase 1 (MMP1) participated in the top two high-ENL performing three-gene classifiers but provided poor classification when considered individually (misclassification errors of 0.2626 and 0.3287, respectively) (Table 2). When these two genes were combined in a two-gene classifier, the misclassification error improved to 0.1568. Furthermore, with the addition of a third gene, delta-like canonical notch ligand 1 (DLL1), Wnt family member 5A (WNT5A) or cytochrome P450 family 4 subfamily F member 3 (CYP4F3) error rates dropped to 0.1342, 0.1346 and 0.1375, respectively.

**Figure 2:**
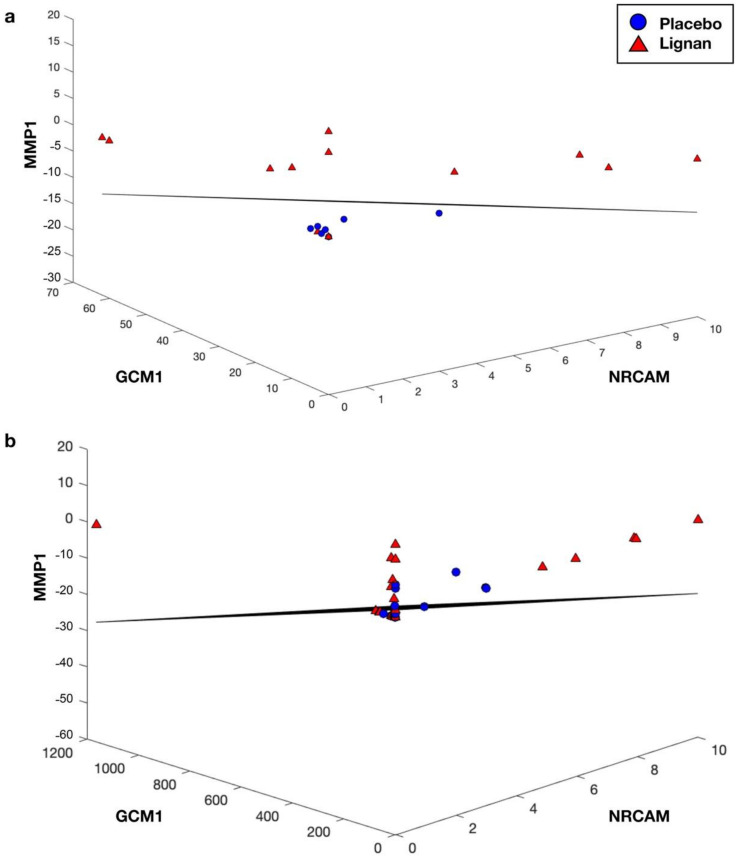
The top performing high-ENL phenotype three-gene classifier based on MMP1, GCM1 and NRCAM expression: (**a**) high-ENL participants only (13 placebo, 14 lignan); (**b**) all participants (25 placebo, 29 lignan).

**Figure 3:**
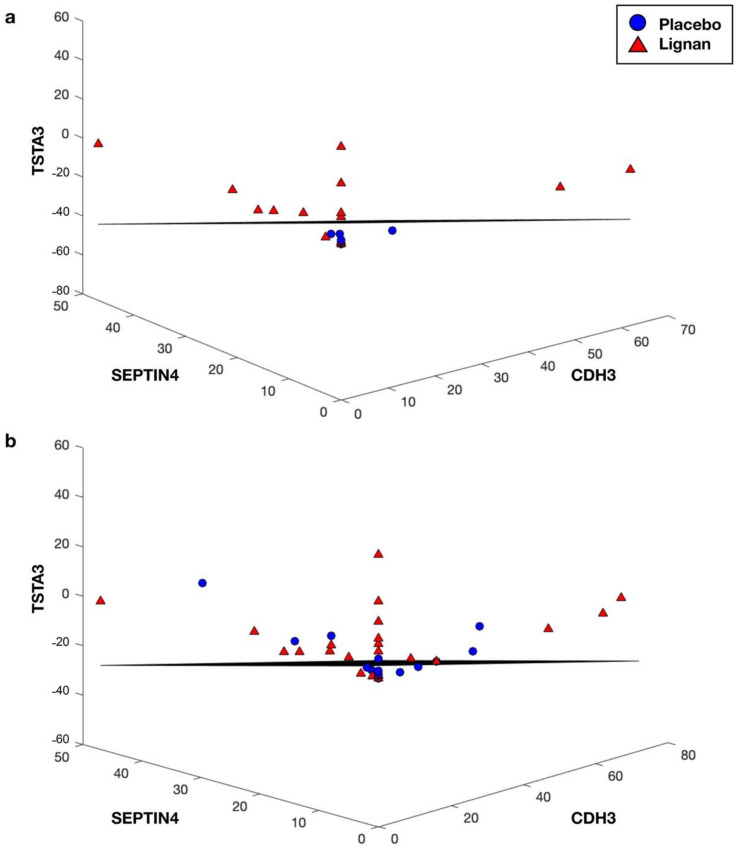
The top performing low-ENL phenotype three-gene classifier based on TSTA3, SEPTIN4 and CDH3 expression: (**a**) low-ENL participants only (12 placebo, 15 lignan); (**b**) all participants (25 placebo, 29 lignan).

Within the low-ENL phenotype, GDP-L-fructose synthase (TSTA3) and septin 4 (SEPTIN4) were in the top three-gene classifiers but performed poorly as single-gene classifiers with misclassification errors of 0.4744 and 0.4457, respectively (Table 3). When combined into a two-gene classifier, the error improved to 0.1773 and with the addition of cadherin 3 (CDH3), niban apoptosis regulator 1 (FAM129A) or RELA proto-oncogene, NF-kB subunit (RELA) error rates decreased to 0.1427, 0.1450 and 0.1537, respectively. Figure 2; Figure 3 show the top performing three-gene classifiers for both ENL phenotypes used for classification within the entire data set and within only the samples of the respective ENL excretion phenotype. 

**Figure 4:**
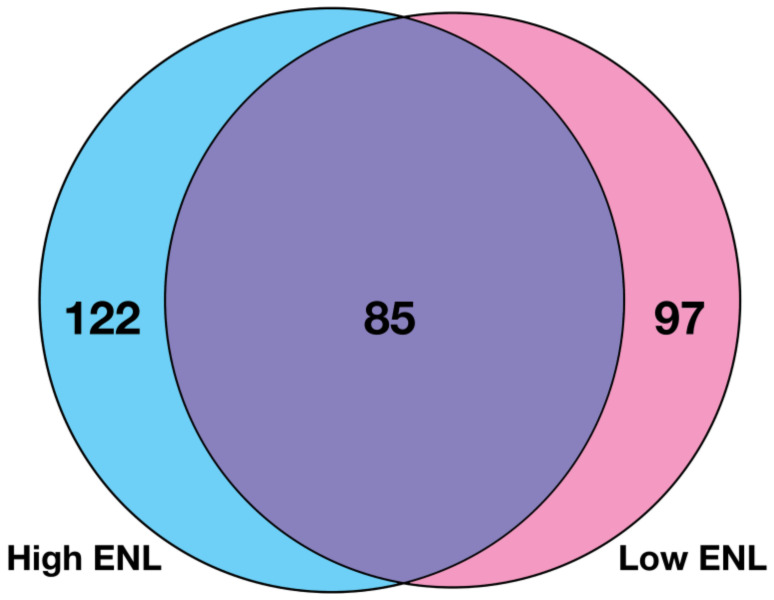
Venn diagram representing individual genes used to generate the top 500 LDA three-gene classifiers for each ENL phenotype.

The LDA classifiers based on three genes contained 304 distinct genes, with 122 unique to high-ENL excreters and 97 unique to low-ENL excreters (Figure 4). 

**Figure 5:**
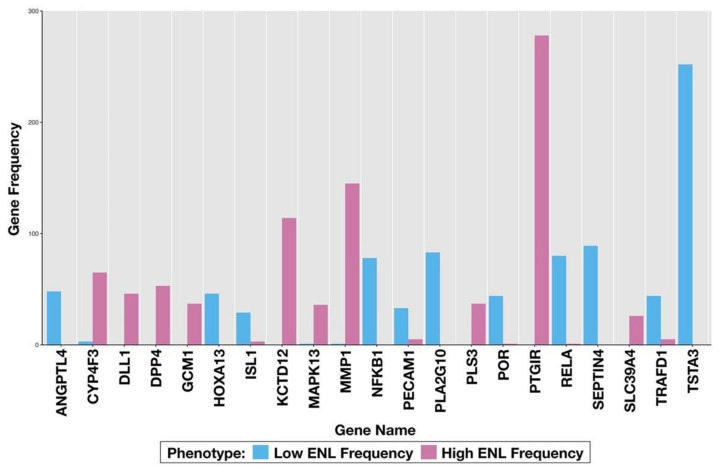
Genes with a frequency of 5% or higher in the top 500 LDA classifiers from flaxseed lignan vs. placebo intervention in each of the two ENL phenotypes. Gene frequencies were determined by their appearance in the top (with respect to their bresub error) 500 LDA three-gene classifiers.

Within the top three-gene classifiers for the high-ENL group, PTGIR was present in 56%, MMP1 was present in 29%, potassium channel tetramerization domain containing 12 (KCTD12) was present in 23% and CYP4F3 was present in 11% of the gene sets, but none of these classifiers were significantly DE (FDR adjusted *p*-value < 0.05). Within the top three-gene classifiers for the low-ENL group, SEPTIN4 was present in 18% and RELA was present in 16% of the top classifiers but neither gene was significantly DE (FDR adjusted *p*-value < 0.05). The frequency of occurrence of genes in the top 500 classifiers is listed in Table S14. We subsequently constructed a histogram of the gene frequencies, including signatures specific to each ENL phenotype in response to the lignan intervention (Figure 5).

### 3.3. IPA Functional Analysis

**Figure 6:**
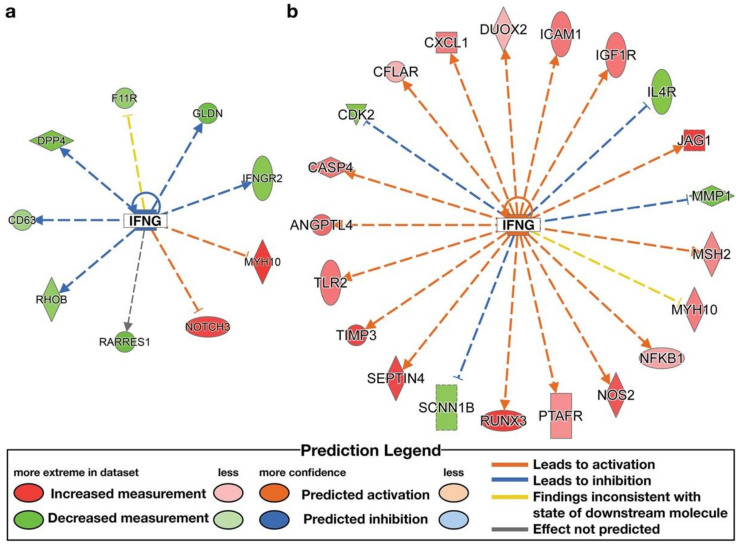
Differentially expressed genes (FDR adjusted *p*-value < 0.05) contributing to the identification of the upstream regulator, interferon gamma (IFNG), following lignan flaxseed intervention: (**a**) predicted inhibition of IFNG in high-ENL excreters (z-score −2.094); (**b**) predicted activation of IFNG in low-ENL excreters (z-score 3.343). The full network of genes contributing to activation of IFNG in low-ENL excreters is described in Supplemental Figure S2.

**Figure 7:**
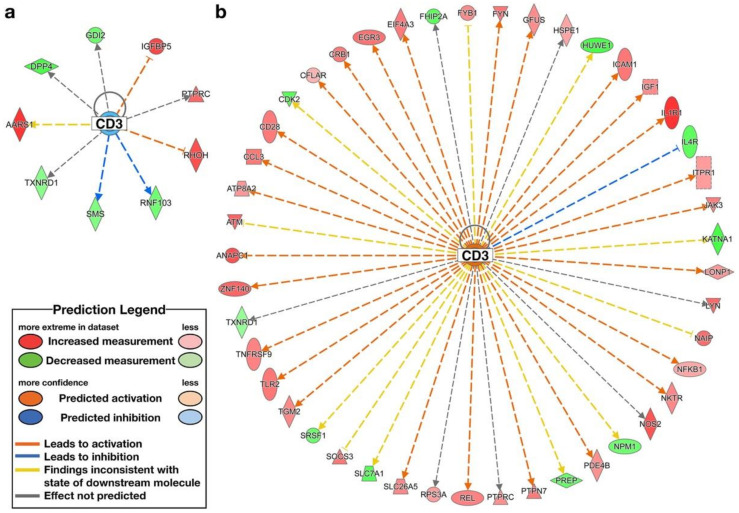
Differentially expressed genes (FDR adjusted *p*-value < 0.05) contributing to the identification of the upstream regulator, cluster of differentiation 3 (CD3) following lignan flaxseed intervention: (**a**) no statistically significant effect in high-ENL excreters (z-score −1.342); (**b**) predicted activation of CD3 in low-ENL excreters (z-score 2.802).

**Figure 8:**
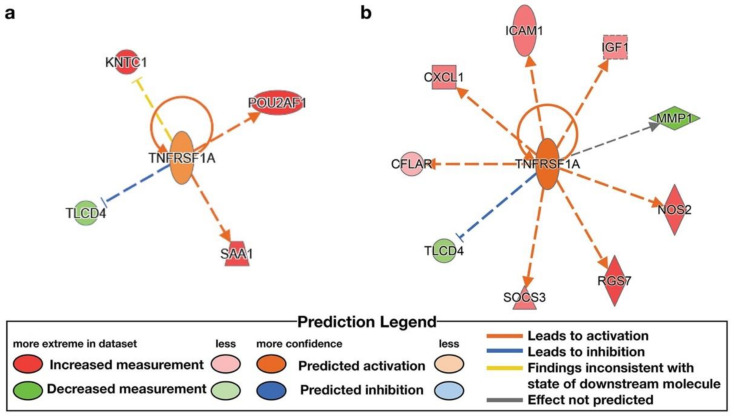
Differentially expressed genes (FDR adjusted *p*-value < 0.05) contributing to the identification of upstream regulator, TNF superfamily receptor 1A (TNFRSF1A) following lignan flaxseed intervention: (**a**) no statistically significant effect in high-ENL excreters (z-score 1.091); (**b**) predicted activation of TNFSF1A in low-ENL excreters (z-score 2.758).

**Figure 9:**
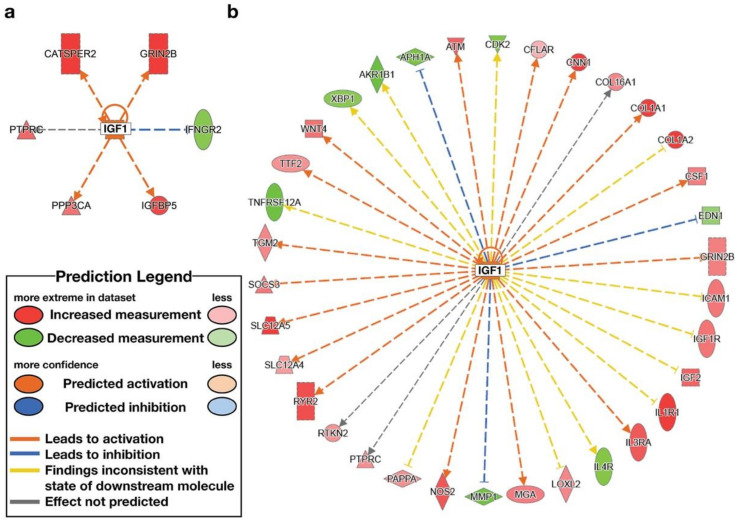
Differentially expressed genes (FDR adjusted *p*-value < 0.05) contributing to the identification of upstream regulator, Insulin like growth factor 1 (IGF1) following lignan flaxseed intervention: (**a**) predicted activation of IGF1 in high-ENL excreters (z-score 2.172); (**b**) no statistically significant effect in low-ENL excreters (z-score 1.828).

IPA analysis was used to identify upstream regulators. The high-ENL exfoliome phenotype exhibited predicted activation of one upstream regulator and inhibition of 2 of 119 potential upstream regulators. The low-ENL exfoliome phenotype exhibited predicted activation of 50 upstream regulators and inhibition of 20 of 327 potential upstream regulators. Out of the 119 high-ENL and 327 low-ENL potential upstream regulators, there were only 14 common potential upstream regulators identified for both phenotypes. Eight of the common potential upstream regulators did not have z-score values listed for one or both ENL phenotypes, three had z-scores indicating the same directionality and the remaining three had z-scores indicating different directionality. Following flaxseed intervention, only four common upstream regulators had z-scores that predicted activation (z-score ≥ 2) or inhibition (z-score ≤ −2) (Table 4), e.g., interferon gamma (IFNG), tumor necrosis factor receptor superfamily member 1A (TNFRSF1A), insulin-like growth factor 1 (IGF1) and cluster of differentiation 3 (CD3) (Figure 6, Figure 7, Figure 8 and Figure 9, Figure S2 and Table 4). 

IFNG, CD3 and TNFRSF1A exhibited predicted activation in low-ENL excreters with z-scores of 3.317, 2.802 and 2.758, respectively. In contrast, IFNG exhibited predicted inhibition (z-score −2.094) in high-ENL and IGF1 exhibited predicted activation (z-score 2.172) in the context of high-ENL phenotype. In addition, there was no statistically significant effect of lignan supplementation on CD3 status in low-ENL excreters (z-score −1.342). Each upstream regulator was associated with different sets of genes in each phenotype that contributed to the IPA predictions. In the case of IFNG, both phenotypes shared increased expression of myosin heavy chain 10 (MYH10). In TNFSF1A, both phenotypes shared decreased expression of TLC domain containing 4 (TLCD4). In IGF1, both phenotypes shared increased expression of glutamate ionotropic receptor NMDA type subunit 2B (GRIN2B) and protein tyrosine phosphatase receptor type C (PTPRC), and in CD3 both phenotypes also shared decreased expression of PTPRC.

## 4. Discussion

Our current findings indicate that systematic analysis of the effects of dietary flaxseed lignan intervention stratified by ENL-excretion yields additional insight into the contributions of the microbial metabolite phenotype with respect to host gene expression. In our previous analysis [6], we computed the effects of flaxseed lignan on gene expression in all 42 participants, regardless of their ENL phenotype, and subsequently compared gene expression between the two ENL phenotypes without consideration of the lignan intervention. Overall, that analysis showed that in response to lignan intervention, there were 973 genes in the exfoliome that exhibited a significant difference (DE with FDR adjusted *p*-value < 0.05); 32 passed the variance filter and were also identified by 2-gene LDA with a bolstered resubstitution error < 0.227. Gene ontology analysis indicated the enrichment of several genes involved in mucosal barrier function and/or cancer—for example, translocation-associated membrane protein 1 (TRAM1), interleukin 18 (IL-18), homeobox A10 (HOXA10), hes family bHLH transcription factor 1 (HES1), ATP binding cassette subfamily A member 5 (ABCA5), ASXL transcriptional regulator 2 (ASXL2), cAMP responsive element binding protein 3 like 3 (CREB3L3), eukaryotic translation initiation factor 5 (EIF5), iron responsive element binding protein 2 (IREB2) and nuclear receptor coactivator 2 (NCOA2). Examination of differences between the low- and high-ENL phenotypes, without consideration of intervention period, suggested that low-ENL excreters were predisposed to proinflammatory events due to the upregulation of nuclear factor kappa B (NF-κB) [18] and nitric oxide synthase 2 (NOS2) [19] and inhibition of the peroxisome proliferator-activated receptor-gamma (PPARγ) [20] network. 

In contrast, herein we examined the effect of lignan intervention in the context of the ENL-phenotype and observed that ABCA5, CREB3L3, HES1, PPAR*γ*, ASXL2 and NCOA2 were not DE (FDR adjusted *p*-value < 0.05), while EIF5, HOXA10, TRAM1, IREB2 were downregulated and NFKB1 and NOS2 were upregulated in response to the lignan intervention for low-ENL excreters only. Overall, low-ENL excreters had approximately 9-fold as many DE genes in response to the lignan extract. This finding suggests that there was a more pronounced effect of the dietary intervention on host gene expression within the group of low-ENL excreters. Few DE genes (*n* = 24) with the same directionality in response to the intervention were shared by the two phenotypes, suggesting this phenotype could be important in determining if a dietary intervention is beneficial to an individual (Figure 1). The differences in upstream regulators identified by the IPA analysis further support important differences between the phenotypes. On several levels, the exfoliome RNA-seq data suggest that key pathways important to cell proliferation, apoptosis and immunomodulation are differentially regulated. For example, high-ENL excreters exhibited activation of the upstream regulator IGF1 and inhibition of IFNG, while low-ENL excreters exhibited activation of IFNG, TNFRSF1A and CD3. For each of these upstream regulators, different gene sets contributed to the predicted activation/inhibition in the two phenotypes (Table 4). Gene ontology analysis also indicated that IFNG, TNFRSF1A and CD3 are involved in the positive regulation of tyrosine phosphorylation of STAT proteins, which are known to be involved in cancer and inflammation [21]. In addition, IPA identified IFNG as an upstream regulator for the genes participating in the top LDA classifiers DLL1, KCTD12, WNT5A, MMP1 in high-ENL and RELA and SEPTIN4 in low-ENL excreters. This striking pattern indicates that IFNG regulates sets of genes that can discriminate between the two phenotypes following lignan intervention, with low-ENL excreters exhibiting activation of this upstream regulator (Figure 6 and Figure S2). This is noteworthy because interferon gamma, in part produced by CD3 positive T cells and TNFRSF1A, are proinflammatory mediators that play a regulatory role in inflammation and immune response [22,23]. 

The results of the LDA classification (Tables S5–S13) underscore the difference in response to lignan extract supplementation within each ENL excretion phenotype. In general, comparison of LDA classification to DE analysis identified a significant number of genes that perform very well when participating in the multivariate LDA analysis while not being significantly DE. Thus, our novel analysis based on the frequency distribution of exfoliome-derived genes in the top LDA three-gene classifiers provides additional insight into the host response to specific ENL phenotypes. The unique frequency signature of each ENL phenotype emphasizes the importance of considering ENL-excretion status when attempting to discriminate between host responsiveness to placebo and lignan treatments (Table S14). Importantly, the frequency distributions show that there is no overlap between the set of the top five genes that were present in at least 10% of the top 500 three-gene LDA classifiers in the low- and high-ENL phenotypes. Finally, the LDA gene frequency signatures allowed us to detect potentially important genes not detected by the traditional univariate DE analysis. 

In our study population, there were no baseline differences in demographic and lifestyle factors between individuals classified as low- and high-ENL excreters [6,24]. Generally, in cross-sectional, observational studies, factors that affect ENL measures include intake of lignan-rich foods, antibiotic use, body mass index, smoking, sex and age [7]. Interpretation of cross-sectional data is difficult insofar as intake of plant lignans, the ENL-precursors, varies considerably across individuals, often influenced by food choices based on ethnicity, sex and socioeconomic status. If exposure to plant lignans is low, the production and excretion of ENL is likely to be low. Consequently, higher ENL excretion is often associated with a high-fiber diet because it reflects exposure to the plant lignans in the high-fiber foods. Here, by phenotyping the participants using a dose of SDG that far exceeds habitual intake, we were able to characterize the capacity of the microbiome to produce ENL. We detected significant associations between ENL excretion at the end of lignan intervention and fecal microbial community composition at baseline and the end of both placebo and lignan intervention periods [6], reflecting the known role of the microbiome in ENL production [7]. 

Gut microbial community metabolism strongly contributes to heterogeneity of metabolite exposure experienced by the host and is an important factor in refining precision nutrition [25]. Our consideration of a microbial metabolite phenotype, i.e., low- and high-ENL excreter, demonstrates the importance of applying metabolite-based stratification to studies of response to diet. In the context of tailoring dietary recommendations to individuals, the strengths of this study include the use of non-invasive-transcriptomic analyses in the context of a randomized, placebo-controlled crossover design in the parent study and supplementation with a high dose of SDG, which allowed for identification of differences in ENL production. The small sample size somewhat limits power for the stratified analysis. Nonetheless, the robust differences (adjusted for multiple comparisons) in intervention response between ENL phenotypes suggest that differences in exposure of the host to ENL and/or the associated microbial metabolism have measurable effects on the modulation of pathways important in immune function and inflammation.

## 5. Conclusions

We have demonstrated that the conversion of flaxseed lignan to ENL in adult humans is associated with a protective anti-inflammatory phenotype. This finding suggests that lignan-derived microbial metabolites are more biologically active than their precursors. Further characterization of the ENL phenotype is required to determine how phenotypic differences in biologic responses mediate risk of CRC and other diseases and will provide further insight for precision nutrition initiatives. 

## Figures and Tables

**Table 1:** Urinary lignan excretion (over 24 h) of SECO, END and ENL at the end of the placebo and flaxseed lignan extract period in low- and high-ENL excreters.

	Low ENL Placebo	High ENL Placebo	*p* Value *	Low ENL Flax	High ENL Flax	*p* Value *
SECO (µmol/24 h)	0.48 (0.63)	0.30 (0.36)	0.28	4.37 (4.45)	7.16 (5.71)	0.007
END (µmol/24 h)	1.27 (3.12)	1.12 (2.46)	0.12	10.83 (15.97)	17.77 (14.15)	0.002
ENL (µmol/24 h)	3.29 (4.05)	11.83 (19.94)	0.002	7.46 (6.97)	61.75 (36.42)	<0.001

Abbreviations: SECO, secoisolariciresinol; END, enterodiol; ENL, enterolactone. Mean (SD) urinary lignan excretion after each intervention period, stratified by low and high ENL excretion (calculated by median excretion after flaxseed lignan extract intervention = 23.36 µmol/24 h), *n* = 42. * *t*-test comparing log-transformed values between low- and high-ENL excreters for each intervention (placebo and flaxseed).

**Table 2:** Top ten one-, two- and three-gene linear discriminant analysis classifiers in high-ENL excreters are shown. Highlighted genes were also differentially expressed after flaxseed lignan extract vs. placebo treatment in high-ENL excreters. “bresub error” indicates the bolstered resubstitution error for the respective classifier (classifiers are ranked according to that error measurement); “∆ε bolstered” represents the decrease in error for each gene set relative to its highest ranked subset of genes.

Gene 1	Gene 2	Gene 3	Bresub Error	∆ε Bolstered
ANXA3			0.2454	
NR6A1			0.2498	
KCTD12			0.2521	
EPHB1			0.2557	
PRKCH			0.2573	
GCM1			0.2597	
PTGIR			0.2626	
PROX1			0.2662	
DLL1			0.2681	
PAX6			0.2687	
MMP1	PTGIR		0.1568	0.1058
CYP4F3	PTGIR		0.1721	0.0905
DLL1	PTGIR		0.1729	0.0897
PLS3	PTGIR		0.1741	0.0885
FOXA1	KCTD12		0.1770	0.0751
COX4I1	SLC39A4		0.1785	0.1179
KCTD12	MAPK13		0.1788	0.0733
KCTD12	RBL2		0.1846	0.0675
NR6A1	PTGIR		0.1847	0.0651
VDR	KCTD12		0.1848	0.0674
NRCAM	GCM1	MMP1	0.1157	0.1066
NRCAM	MMP1	PLD3	0.1282	0.0920
CA14	GCM1	MMP1	0.1291	0.0932
OTUB1	CYP4F3	PTGIR	0.1296	0.0425
GCM1	MMP1	PECAM1	0.1298	0.0924
BMP4	GCM1	MMP1	0.1331	0.0740
COX4I1	FOXO3	SLC39A4	0.1340	0.0445
DLL1	MMP1	PTGIR	0.1342	0.0226
MMP1	PTGIR	WNT5A	0.1346	0.0222
CYP4F3	MMP1	PTGIR	0.1375	0.0193

Highlights indicate differentially expressed genes following flaxseed lignan extract vs. placebo treatment in high-ENL excreters.

**Table 3:** Top ten one-, two- and three-gene linear discriminant analysis classifiers in low-ENL excreters are shown. Highlighted genes were also differentially expressed after flaxseed lignan extract vs. placebo treatment in low-ENL excreters. “bresub error” indicates the bolstered resubstitution error for the respective classifier (classifiers are ranked according to that error measurement); “∆ε bolstered” represents the decrease in error for each gene set relative to its highest ranked subset of genes.

Gene 1	Gene 2	Gene 3	Bresub Error	∆ε Bolstered
ANXA3			0.2454	
NR6A1			0.2498	
KCTD12			0.2521	
EPHB1			0.2557	
PRKCH			0.2573	
GCM1			0.2597	
PTGIR			0.2626	
PROX1			0.2662	
DLL1			0.2681	
PAX6			0.2687	
ANGPTL4	RELA		0.1702	0.0164
SEPTIN4	TSTA3		0.1773	0.1020
ISL1	TSTA3		0.1799	0.1111
ANGPTL4	GJB1		0.1803	0.0063
NFKB1	TRAFD1		0.1824	0.1428
NFKB1	TSTA3		0.1835	0.1076
ANGPTL4	TSTA3		0.1861	0.0005
PECAM1	TSTA3		0.1862	0.0990
BCL2	ANGPTL4		0.1871	0.2701
NFKB1	PROX1		0.1906	0.1360
CDH3	SEPTIN4	TSTA3	0.1427	0.0346
PLA2G10	HOXA13	ULK1	0.1429	0.0779
PLA2G10	HOXA13	MAML1	0.1429	0.0779
FAM129A	SEPTIN4	TSTA3	0.1450	0.0323
CACNB4	RELA	TSTA3	0.1465	0.0708
NANOG	RELA	TSTA3	0.1468	0.0639
CYP4F3	HOXA13	KCTD17	0.1523	0.0685
POR	NFKB1	TRAFD1	0.1537	0.0288
RELA	SEPTIN4	TSTA3	0.1537	0.0237
WNT4	RELA	TSTA3	0.1540	0.0565

Highlights indicate differentially expressed genes following flaxseed lignan extract vs. placebo treatment in high-ENL excreters.

**Table 4:** Putative upstream regulators showing activation or inhibition in high- ENL and/or low-ENL excreters. The upstream regulators are ordered by difference in the high-ENL activation z-score and low-ENL activation z-score.

Upstream Regulator	Molecule Type	Phenotype	Predicted Activation State	Activation z-Score	Target Molecules in Dataset
IFNG	cytokine	High ENL	Inhibited	−2.094	CD63, DPP4, F11R, GLDN, IFNGR2, MYH10, NOTCH3, RARRES1, RHOB
IFNG	cytokine	Low ENL	Activated	3.343	ACE, ADCY5, ADGRG2, ADRA2A, AGER, ANGPTL4, APOBEC3G, APOL1, ATM, ATP2A2, BLNK, CASP4, CCL3, CDH5, CDK2, CFB, CFLAR, CHRNG, CIITA, COL1A1, COL1A2, CRIM1, CSF1, CSF2RB, CTSC, CXCL1, CXCL11, CYRIA, DEPP1, DUOX2, EDN1, EGR3, ELK1, ETV7, FANCF, FCGR3A/FCGR3B, FKBP5, FTX, GAL3ST1, GBP2, GNAO1, GNG7, HDAC9, HERC6, HLADMA, ICAM1, IFI16, IGF1, IGF1R, IL1R1, IL1RN, IL3RA, IL4R, IREB2, ISL1, ITPR1, JAG1, JAK3, KYNU, LCP2, LY75, LYN, MAP2, MITF, MMP1, MSH2, MX2, MYH10, NEURL3, NFE2L3, NFKB1, NLRP3, NOS2, P2RY14, PAPPA, PECAM1, PHF11, PIM2, PLA2G7, PLAAT3, PSME2, PTAFR, QPRT, RAE1, RUNX3, SCIMP, SCLY, SCNN1B, SCUBE1, SEPTIN4, SLC1A2, SNAP25, SOCS3, TIMP3, TLR2, TNFAIP2, TNFRSF12A, TSC22D3, UBA2, VRK2
CD3	complex	High ENL		−1.342	AARS1, DPP4, GDI2, IGFBP5, PTPRC, RHOH, RNF103, SMS, TXNRD1
CD3	complex	Low ENL	Activated	2.802	ANAPC1, ATM, ATP8A2, CCL3, CD28, CDK2, CFLAR, CRB1, EGR3, EIF4A3, FHIP2A, FYB1, FYN, GFUS, HSPE1, HUWE1, ICAM1, IGF1, IL1R1, IL4R, ITPR1, JAK3, KATNA1, LONP1, LYN, NAIP, NFKB1, NKTR, NOS2, NPM1, PDE4B, PREP, PTPN7, PTPRC, REL, RPS3A, SLC26A5, SLC7A1, SOCS3, SRSF1, TGM2, TLR2, TNFRSF9, TXNRD1, ZNF140
TNFRSF1A	trans- membrane receptor	High ENL		1.091	KNTC1, POU2AF1, SAA1, TLCD4
TNFRSF1A	trans- membrane receptor	Low ENL	Activated	2.758	CFLAR, CXCL1, ICAM1, IGF1, MMP1, NOS2, RGS7, SOCS3, TLCD4
IGF1	growth factor	High ENL	Activated	2.172	CATSPER2, GRIN2B, IFNGR2, IGFBP5, PPP3CA, PTPRC
IGF1	growth factor	Low ENL		1.828	AKR1B1, APH1A, ATM, CDK2, CFLAR, CNN1, COL16A1, COL1A1, COL1A2, CSF1, EDN1, GRIN2B, ICAM1, IGF1, IGF1R, IGF2, IL1R1, IL3RA, IL4R, LOXL2, MGA, MMP1, NOS2, PAPPA, PTPRC, RTKN2, RYR2, SLC12A4, SLC12A5, SOCS3, TGM2, TNFRSF12A, TTF2, WNT4, XBP1

## Data Availability

Data is available at NCBI BioProject, accession# PRJNA308214. (https://www.ncbi.nlm.nih.gov/bioproject/PRJNA308214).

## Supplementary Materials

The following supporting information can be downloaded at: https://www.mdpi.com/article/10.3390/nu14122377/s1, Figure S1: Data pipeline used for pre-processing, normalization, and analysis; Figure S2: Low-ENL IFNG Network; Table S1: Available Exfoliome Samples; Table S2: Linear Discriminate Analysis Genes; Table S3: High-ENL DE Genes; Table S4: Low-ENL DE Genes; Table S5: Top 500 Single-Gene LDA Classifiers; Table S6: Top 500 Two-Gene LDA Classifiers; Table S7: Top 500 Three-Gene LDA Classifiers; Table S8: Top 500 high-ENL Single-Gene LDA Classifiers; Table S9: Top 500 high-ENL Two-Gene LDA Classifiers; Table S10: Top 500 high-ENL Three-Gene LDA Classifiers; Table S11: Top 500 low-ENL Single-Gene LDA Classifiers; Table S12: Top 500 low-ENL Two-Gene LDA Classifiers; Table S13: Top 500 low-ENL Three-Gene LDA Classifiers; Table S14: Frequencies of Top 500 Three-Gene Classifiers.
