# Supplementary material for: Personalized Nutrition Using Microbial Metabolite Phenotype to Stratify Participants and Non-Invasive Host Exfoliomics Reveal the Effects of Flaxseed Lignan Supplementation in a Placebo-Controlled Crossover Trial
Source: Nutrients. 2022 Jun 8;14(12):2377. doi: 10.3390/nu14122377 (PMC9230005; doi:10.3390/nu14122377)
Supplement: Supplementary file 1 [file nutrients-14-02377-s001.zip › ENL_Manuscript_Supplemental_Figures__Final_04222022.pdf]

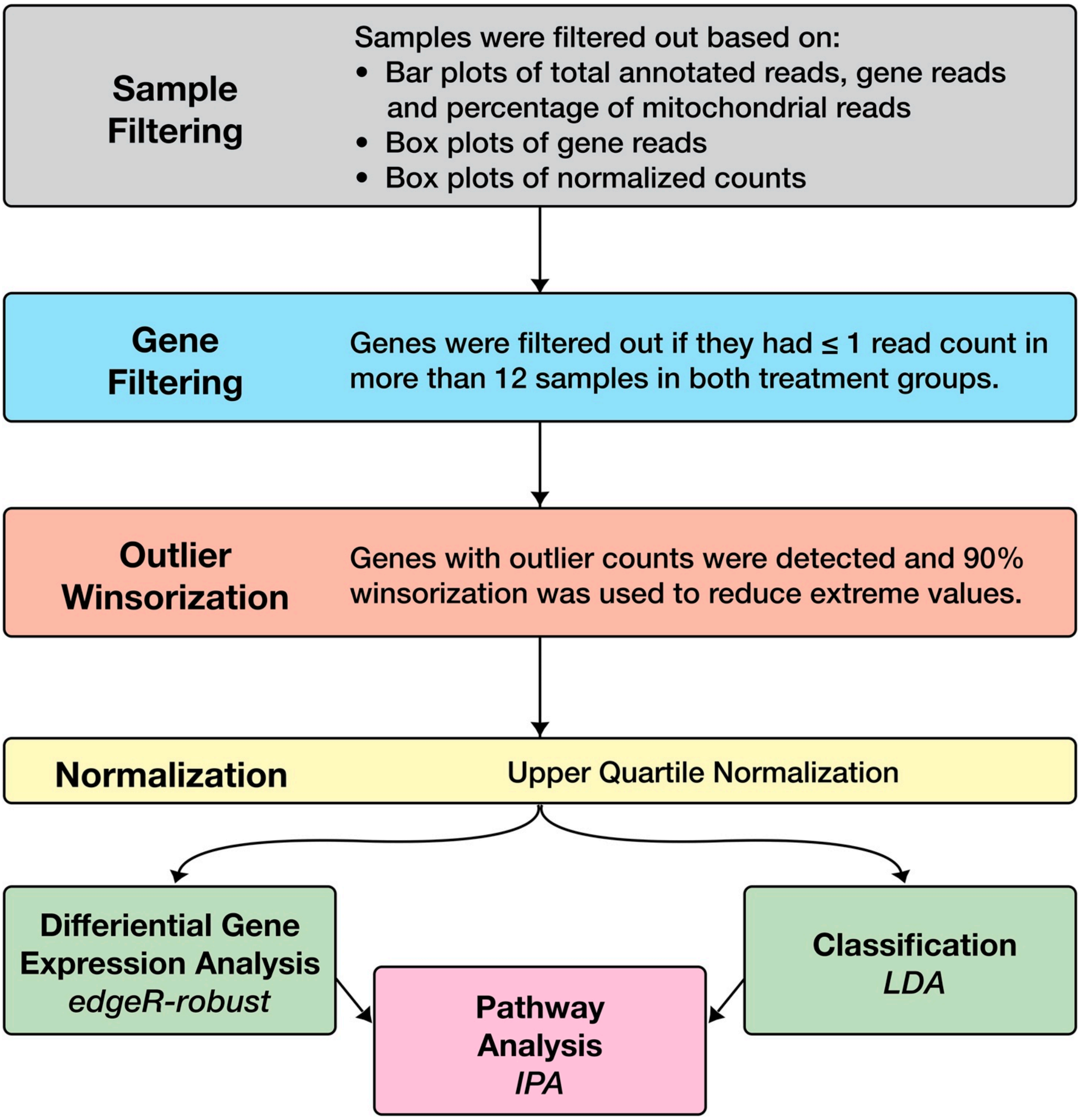

Figure S1. Data pipeline used for pre-processing, normalization, and analysis. Genes were filtered out if they had a zero read count in more than twelve samples in both treatment groups. Outliers were detected using iterative leave-one-out approach and then a 90% winsorization was applied. Data were normalized using the upper quartile method with edgeR-robust prior to differential expression analysis and LDA classification. Pathway analysis was subsequently performed using IPA for DE genes. Genes in the top ten performing triplicates from the LDA classification were identified.

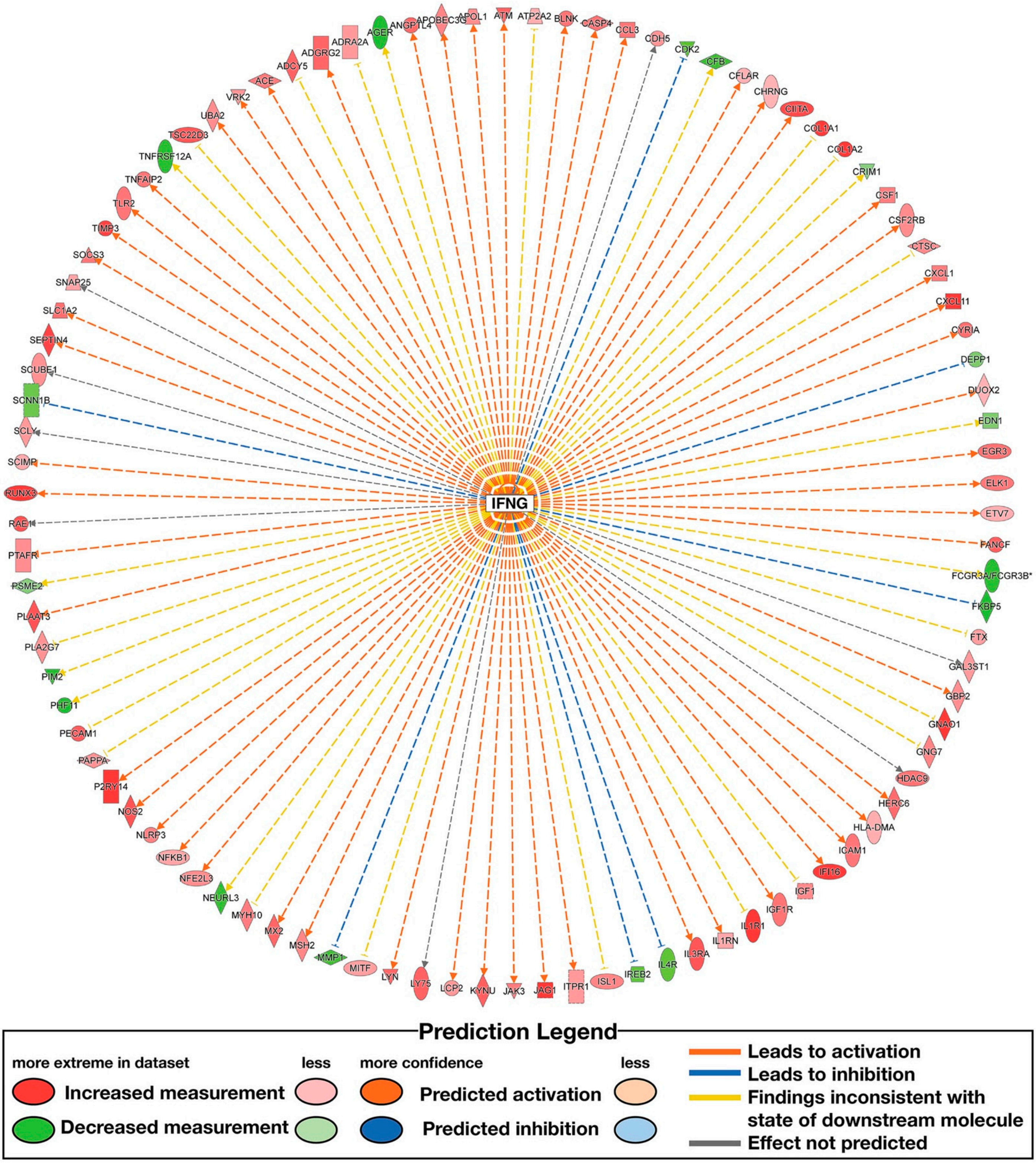

Figure S2. Low-ENL IFNG Network IPA analysis using differentially expressed (DE) genes (FDR adjusted p-value < 0.05) contributing to the identification of upstream regulator, interferon gamma (IFNG) following lignan flaxseed intervention. The network predicts activation in low-ENL excreters (z-score 3.343).
