# Supplementary material for: Personalized Nutrition Using Microbial Metabolite Phenotype to Stratify Participants and Non-Invasive Host Exfoliomics Reveal the Effects of Flaxseed Lignan Supplementation in a Placebo-Controlled Crossover Trial
Source: Nutrients. 2022 Jun 8;14(12):2377. doi: 10.3390/nu14122377 (PMC9230005; doi:10.3390/nu14122377)
Supplement: Supplementary file 1 [file nutrients-14-02377-s001.zip › ENL_Manuscript_Supplemental_Tables_Final_04222022.pdf]

Table S1. Available Exfoliome Samples. Number of exfoliome samples available for DE analysis after collection, sequencing and quality control for the lignan supplement and placebo intervention periods among individuals in the high and low ENL groups.

Sample Type	High ENL	Low ENL
Lignan	14	15
Placebo	13	12
Paired	11	9
Unpaired	5	9

Table S2. Linear Discriminate Analysis Genes. List of 840 *a priori* genes related to the gene-regulatory processes in the human gut and detected in the exfoliome samples used for Linear Discriminant Analysis.

AATF	CDC42EP3	FGF5	IGF2	MYO9B	PTGS2	SLC5A3
ABCB1	CDCA7	FGF9	IGF2BP1	NAA10	PTK2	SLC5A8
ABCC1	CDCP1	FGFBP1	IGFBP3	NAIP	PTK2B	SLC5A9
ABCG2	CDH1	FGFR1	IHH	NANOG	PTK7	SLC6A14
ABCG5	CDH2	FGFR2	IKBKAP	NCAM1	PTPN11	SLC6A19
ABHD5	CDH3	FGFR3	IKBKB	NEU3	PTPN2	SLC6A20
ACADS	CDH5	FLNB	IL13RA1	NEUROD1	PTPN22	SLC6A6
ACAT1	CDK15	FLT1	IL18	NFE2L2	PTPN6	SLC7A11
ACKR2	CDK2	FN1	IL1A	NFIL3	PTPRD	SLC7A6
ACSL4	CDK2AP1	FOS	IL1B	NFKB1	PYGO2	SLC7A7
ACTA1	CDK7	FOXA1	IL1R1	NFKB2	RAC1	SLC7A9
ACTA2	CDK8	FOXA2	IL1R2	NIFK	RAD51D	SLC9A2
ACTB	CDKAL1	FOXA3	IL22RA1	NOD2	RAD52	SLC9A3R1
ACTC1	CDKN1A	FOXC1	IL23R	NOG	RASSF1	SLC9A8
ACTG1	CDKN1B	FOXD2	IL2RB	NOS1	RB1	SLCO2A1
ACTG2	CDKN1C	FOXJ2	IL2RG	NOS2	RBBP9	SLFN5
ADAM10	CDKN2A	FOXJ3	IL36RN	NOTCH1	RBL1	SMAD3
ADAM17	CDKN2B	FOXK2	IL4R	NOTCH3	RBL2	SMAD7
ADAM9	CDX1	FOXO1	IL6ST	NOTCH4	RBP4	SMPD1
ADAMTS4	CDX2	FOXO3	ILF2	NPAT	RBPJ	SOCS2
ADRB1	CEACAM1	FOXP1	ILF3	NR2C2	RCN3	SOCS6
AGPAT3	CEACAM19	FOXP2	ILKAP	NR3C1	REG1A	SOD1
AIFM2	CEACAM3	FOXP4	INSR	NR5A2	REG1B	SOD2
AKTIP	CEACAM5	FPR1	IRF8	NR6A1	REG3A	SORD
ALCAM	CEACAM6	FSTL1	ISL1	NRAS	REG4	SOX4
ALDH1A1	CEACAM7	FTH1	ITGAL	NRCAM	REL	SOX9
ALOX15	CEBPB	FTL	ITGAM	NRIP1	RELA	SP1
ALPI	CFLAR	FUT2	ITGB1	NTHL1	RELB	SPDEF
AMPD3	CFTR	FZD7	ITGB2	NTS	REST	SPI1
ANGPTL4	CGNL1	G6PD	ITLN1	NUMB	RHOB	SPIB
ANXA1	CHAC1	GABRB3	JAG1	NUPR1	RIPK1	SPP1
ANXA3	CHGA	GADD45A	JAK1	OAT	ROCK1	SPPL3
ANXA5	CHI3L1	GADD45B	JAK2	OCLN	RPS4X	SPRY2
APC	CHP1	GATA6	JUN	ODC1	RPSA	SQSTM1
APEX1	CKAP4	GCLC	KCNH2	OGG1	RUNX2	SREBF2
APOA1	CKS2	GCLM	KCNS3	OLFM4	RUNX3	SST
APOB	CLDN3	GCM1	KCTD12	ORMDL3	RXRA	STAT3
APOC3	CLDN4	GCNT3	KCTD17	OTUB1	S100A8	STIM1
APP	CLDN8	GDF15	KDM4A	P2RX4	S100A9	STK11
AQP11	COL1A1	GEMIN6	KDM4B	P4HA1	SAR1B	SYP
AQP3	COL1A2	GJB1	KDM4C	P4HB	SAT2	TAT
AQP7	COL3A1	GJB2	KDM5A	PAG1	SCD	TAZ
AQP8	COL6A3	GJC1	KEAP1	PARD3	SCNN1A	TBRG1
ARNT	COLEC12	GJD2	KIT	PARK7	SCNN1B	TCF4
ARRB2	COMMD3	GNL1	KITLG	PAWR	SCP2	TCF7L2

ATF3	COX4I1	GPX1	KLF4	PAX6	SDC1	TCN2
ATF4	COX5B	GPX2	KLF6	PCDH7	SELL	TDG
ATG16L1	CSPG5	GPX4	KLK1	PCNA	SEPTIN4	TFAP2A
ATG5	CTDSPL	GRB7	KLK6	PDE4A	SERPINA1	TFAP2C
ATOH1	CTNNA1	GREM1	KRAS	PDE4B	SERPINB2	TFCP2L1
ATP2A3	CTNNB1	GSK3A	KRT19	PDE4C	SERPINH1	TFF3
ATP5B	CTNND1	GSN	KRT8	PDE4D	SFN	TFRC
ATP8A1	CXADR	GSR	LAMA1	PDE6D	SHH	TGFA
AXIN1	CXCL1	GSS	LAMB1	PDK1	SIGIRR	TGFB2
AXIN2	CXCL16	GSTA1	LAMC2	PDX1	SIRT1	TGFBR2
BACH1	CXCL2	GSTA4	LAMP1	PDZK1IP1	SLC10A2	TGM2
BAD	CXCL3	GSTM3	LATS2	PECAM1	SLC13A1	TGM4
BAK1	CXCR2	GSTP1	LCT	PIK3CA	SLC13A2	THBS1
BAMBI	CXCR4	GUCA2A	LDHB	PIK3R1	SLC16A1	THRA
BAX	CYB5R1	GUCA2B	LEF1	PIWIL2	SLC16A3	TIMP3
BBC3	CYBB	HAS2	LGALS3	PKM	SLC16A4	TJP1
BCL10	CYP4F2	HAS3	LGALS3BP	PKN2	SLC16A9	TJP2
BCL2	CYP4F3	HCAR2	LGR4	PLA2G10	SLC17A5	TJP3
BCL2A1	DAB2IP	HDAC1	LIFR	PLA2G2A	SLC1A1	TLR2
BCL2L1	DAPK1	HDAC2	LIG3	PLA2G4B	SLC1A4	TLR3
BCL2L11	DAPK2	HES1	LIN28A	PLA2G4C	SLC20A1	TLR4
BCL2L13	DAPK3	HEY1	LMNB2	PLA2G4D	SLC22A23	TLR5
BCL2L14	DBI	HHIP	LPAR1	PLA2G7	SLC23A3	TNF
BCL2L2	DBN1	HIF1A	LPAR4	PLAA	SLC25A13	TNFAIP1
BCO2	DCN	HIF3A	LRIG1	PLAT	SLC25A20	TNFAIP3
BDKRB2	DDIT4	HIST3H3	LRRK2	PLAU	SLC25A28	TNFRSF10A
BECN1	DDR2	HLTF	LTA4H	PLAUR	SLC25A33	TNFRSF10B
BIRC2	DDX11	HMGA1	LTBP1	PLCB2	SLC25A36	TNFRSF10D
BIRC5	DDX17	HMGB1	LYPD8	PLCB3	SLC25A37	TNFRSF11A
BMF	DDX5	HMGCL	LYPLA1	PLCB4	SLC25A39	TNFRSF1A
BMI1	DEFA5	HMGCR	MAB21L2	PLCD1	SLC26A2	TNFRSF1B
BMP2	DEFA6	HMGCS1	MACC1	PLCD3	SLC26A3	TNFRSF21
BMP4	DFFA	HMGCS2	MALL	PLCE1	SLC26A6	TNFSF10
BMPR2	DHFR	HMOX1	MAML1	PLCG2	SLC27A2	TNFSF13B
BRAF	DHRS9	HNF4A	MAML3	PLD3	SLC28A1	TNK2
BRD2	DLL1	HNF4G	MAP2K2	PLIN2	SLC28A2	TOLLIP
BRIX1	DLL4	HNRNPA1	MAPK1	PLIN3	SLC2A1	TP53
BST1	DNAJB6	HOPX	MAPK10	PLS3	SLC2A10	TP63
BTD	DOT1L	HOXA10	MAPK13	PODXL	SLC2A13	TPM3
C11orf30	DPEP1	HOXA13	MAPK14	POLB	SLC2A2	TPT1
CA1	DPP4	HOXA2	MAPK3	POLG	SLC2A5	TRAFD1
CA12	DPYSL3	HOXA3	MAPK6	POR	SLC30A4	TREH
CA13	DSG2	HOXA5	MAPK7	POU2F3	SLC30A5	TRIM31
CA14	DUOX2	HOXA9	MAPK8	PPAP2A	SLC30A6	TSC2
CA2	DUSP1	HOXB13	MAPK9	PPAP2B	SLC30A7	TSC22D1
CA3	DUT	HOXB3	MAX	PPAP2C	SLC30A9	TSC22D3
CA4	ECM1	HOXB5	MEF2C	PPARD	SLC31A1	TSTA3

CA5B	EFNB1	HOXB6	MEIS1	PPARG	SLC34A2	TTN
CA7	EGF	HOXB7	MELK	PPARGC1A	SLC35A1	TYRO3
CACNA1C	EGFR	HOXB8	MEN1	PPM1D	SLC35A2	UHRF1
CACNA2D1	EIF6	HOXC4	MEP1A	PRKACA	SLC35A3	ULK1
CACNB2	ELF3	HOXC6	MET	PRKACB	SLC35B3	VDR
CACNB4	ELP3	HOXC8	MFSD2A	PRKAR1A	SLC35D1	VIL1
CAMK1D	ENC1	HOXD1	MGAT3	PRKAR1B	SLC37A4	VIM
CAMK2B	EP400	HOXD10	MGST1	PRKCA	SLC38A1	VNN1
CAMK2D	EPAS1	HOXD13	MLH1	PRKCB	SLC38A2	WLS
CASK	EPHB1	HOXD8	MLLT4	PRKCD	SLC39A4	WNT2B
CASP1	EPHB4	HPGD	MMP1	PRKCE	SLC39A5	WNT3
CASP3	EPHB6	HRAS	MMP9	PRKCH	SLC39A7	WNT4
CASP4	ERBB2	HRH1	MPC1	PRKCI	SLC39A8	WNT5A
CAT	ERBB4	HSD17B2	MPC2	PRKDC	SLC39A9	WNT7A
CAV1	ESR1	HSPA1A	MSH2	PRNP	SLC3A1	XIST
CBX5	F11R	HSPA1B	MSH3	PROM1	SLC3A2	XRCC6
CCDC80	FABP1	HSPD1	MSH6	PROM2	SLC40A1	YAP1
CCL15	FABP2	ICAM1	MSI1	PROX1	SLC43A2	YWHAB
CCL28	FABP3	ICK	MSI2	PRSS23	SLC44A1	YWHAE
CCND1	FABP6	ID1	MUC1	PSME2	SLC44A3	YWHAG
CD24	FAM129A	ID2	MUC17	PTAFR	SLC4A4	YWHAH
CD36	FAS	ID3	MUC19	PTBP1	SLC4A7	YWHAQ
CD44	FASLG	ID4	MUC2	PTCH1	SLC50A1	YWHAZ
CD59	FASN	IER3	MUC3A	PTEN	SLC51A	YY1
CD74	FDFT1	IFITM2	MUC4	PTGER4	SLC51B	ZBTB33
CD9	FFAR2	IFNAR1	MXI1	PTGES	SLC5A1	ZDHHC20
CDC25B	FFAR4	IGF1	MYB	PTGFRN	SLC5A11	ZEB2
CDC42	FGF18	IGF1R	MYC	PTGIR	SLC5A12	ZNF365

Table S3. High-ENL DE Genes. Differentially expressed (DE) genes in the contrast flaxseed lignan supplement vs placebo treatment in high-ENL excreters, sorted by their respective FDR.
Abbreviations: logFC, log fold change; logCPM, log counts per million; LR, likelihood ratio; FDR, False Discovery Rate corrected p-values; FC, fold change.
*Genes that also appear as DE in the Low ENL participants with the same directionality (upregulation/downregulation).

	**logFC**	**logCPM**	**LR**	**P-Value**	**FDR**	**FC**
ARMCX4	6.0477	1.5671	26.7692	2.29E-07	2.65E-03	66.1533
AIM1	-4.5554	3.5694	24.6864	6.75E-07	3.11E-03	0.0425
CREB3L1	-3.6000	8.5348	24.3374	8.09E-07	3.11E-03	0.0825
POU2AF1	4.6753	1.0426	23.1157	1.53E-06	4.41E-03	25.5512
RP11-370I10.2	4.7831	1.0020	22.5790	2.02E-06	4.66E-03	27.5326
SAMD15*	5.0488	1.1090	21.8922	2.88E-06	5.56E-03	33.1012
FAM83H	-6.7172	2.5243	20.6628	5.48E-06	9.04E-03	0.0095
GRHL2	-4.6545	4.2347	19.8287	8.47E-06	1.22E-02	0.0397
PRDX6	-2.8653	10.2168	19.1092	1.23E-05	1.40E-02	0.1372
ZAK	-6.1162	3.1571	19.0036	1.30E-05	1.40E-02	0.0144
RALA	-4.2525	6.4419	18.9572	1.34E-05	1.40E-02	0.0525
CACNG8*	5.2391	1.6536	18.5980	1.61E-05	1.50E-02	37.7674
GTF2F1	-5.5037	2.3855	18.4073	1.78E-05	1.50E-02	0.0220
GEN1*	4.6283	1.1685	18.3763	1.81E-05	1.50E-02	24.7321
HPS1	-3.7463	7.2443	18.2116	1.98E-05	1.52E-02	0.0745
MEIS1-AS2	4.1466	0.8861	17.8528	2.39E-05	1.68E-02	17.7113
TMEM38B	-4.4023	4.1493	17.7863	2.47E-05	1.68E-02	0.0473
GLRX5	-3.3949	3.9272	17.6521	2.65E-05	1.70E-02	0.0951
IFNGR2	-3.0034	9.8143	17.5277	2.83E-05	1.72E-02	0.1247
SNX5	-3.5364	4.3347	17.1200	3.51E-05	1.90E-02	0.0862
UBE2N	-3.3893	6.2470	17.0291	3.68E-05	1.90E-02	0.0954
SAA1	3.6714	0.6673	16.9472	3.84E-05	1.90E-02	12.7413
TRPS1	5.6531	4.7965	16.8152	4.12E-05	1.90E-02	50.3200
HIGD1A	-2.4801	9.0244	16.8112	4.13E-05	1.90E-02	0.1792
KIF20B*	4.1406	1.0094	16.7600	4.24E-05	1.90E-02	17.6378
TRAPPC12	4.6587	1.1248	16.6830	4.42E-05	1.90E-02	25.2589
ADD3	-3.7702	4.3604	16.6034	4.61E-05	1.90E-02	0.0733
TDG	-4.8059	3.8774	16.4593	4.97E-05	1.90E-02	0.0358
F11R	-2.5455	6.9395	16.4493	5.00E-05	1.90E-02	0.1713
SH3BGR	4.3756	0.8932	16.3248	5.34E-05	1.90E-02	20.7574
CCDC146	5.9065	1.6622	16.3091	5.38E-05	1.90E-02	59.9846
GHDC	3.4554	0.7468	16.3091	5.38E-05	1.90E-02	10.9692
ADIPOR2*	-2.7360	8.3881	16.2334	5.60E-05	1.90E-02	0.1501
TIMMDC1	-4.2061	3.5221	16.2168	5.65E-05	1.90E-02	0.0542
MIR222HG	4.8809	1.3071	16.1780	5.77E-05	1.90E-02	29.4648
PPP3CA	2.8325	6.9668	16.0084	6.31E-05	1.94E-02	7.1231
NOTCH3	3.8016	0.8174	15.9985	6.34E-05	1.94E-02	13.9444
GREB1L	4.3685	0.9699	15.9837	6.39E-05	1.94E-02	20.6564
YBX1	-3.1446	5.3421	15.8028	7.03E-05	1.99E-02	0.1131
LRRN2	4.8952	1.3362	15.7788	7.12E-05	1.99E-02	29.7572
MYH10*	4.1373	1.1666	15.7514	7.22E-05	1.99E-02	17.5971

GRIN2B*	4.6850	1.6430	15.7038	7.41E-05	1.99E-02	25.7237
RHOB	-2.7067	8.9046	15.7019	7.42E-05	1.99E-02	0.1532
TNFRSF21	-2.7893	6.5795	15.6572	7.59E-05	1.99E-02	0.1447
XPO4	3.6871	1.1784	15.4803	8.34E-05	2.14E-02	12.8804
SRPK2	4.1132	3.0635	15.2794	9.27E-05	2.30E-02	17.3062
ATP10B	3.5229	5.3105	15.2310	9.51E-05	2.30E-02	11.4944
ARPC5	2.2167	8.1810	15.2266	9.54E-05	2.30E-02	4.6482
ANO5	3.9639	1.9658	15.1595	9.88E-05	2.32E-02	15.6046
COA5	-6.1043	2.2570	15.1328	1.00E-04	2.32E-02	0.0145
PTPRC*	3.0748	5.5875	15.0171	1.07E-04	2.40E-02	8.4256
ELF1	-2.3973	3.4464	14.9943	1.08E-04	2.40E-02	0.1898
RNF103	-2.7475	6.8063	14.8480	1.17E-04	2.46E-02	0.1489
RGP1	4.1201	1.4286	14.8346	1.17E-04	2.46E-02	17.3885
PLEK2	-5.0679	3.4114	14.7930	1.20E-04	2.46E-02	0.0298
KTN1	2.2703	6.9790	14.7801	1.21E-04	2.46E-02	4.8243
TRBVA	5.6996	1.2832	14.7735	1.21E-04	2.46E-02	51.9693
PPP1R13L	-4.4569	1.5330	14.6131	1.32E-04	2.61E-02	0.0455
MFI2	3.3110	1.7605	14.5965	1.33E-04	2.61E-02	9.9247
SPIN1	-3.1876	4.5931	14.4705	1.42E-04	2.71E-02	0.1098
TMEM56*	-2.4639	6.6609	14.4629	1.43E-04	2.71E-02	0.1813
BBOX1	3.6382	1.3675	14.3274	1.54E-04	2.84E-02	12.4510
COX7A2	-2.2539	6.9775	14.2837	1.57E-04	2.84E-02	0.2097
EPN2	3.3850	1.1459	14.2783	1.58E-04	2.84E-02	10.4472
TFAP2D	4.8542	2.4525	14.2529	1.60E-04	2.84E-02	28.9247
ARHGAP21	-3.2726	4.5048	14.2102	1.63E-04	2.86E-02	0.1035
AC079612.2*	4.3148	1.7490	14.0947	1.74E-04	3.00E-02	19.9018
SRRD	4.0932	0.9203	14.0144	1.81E-04	3.04E-02	17.0676
EIF1AD	-4.3271	5.6577	13.9839	1.84E-04	3.04E-02	0.0498
DPP4	-3.6145	3.5270	13.9833	1.84E-04	3.04E-02	0.0816
PRELID2	-3.0099	8.2757	13.9589	1.87E-04	3.04E-02	0.1241
RARRES1	-4.6457	2.6933	13.9158	1.91E-04	3.07E-02	0.0399
PIGV	5.1255	1.3413	13.8693	1.96E-04	3.07E-02	34.9085
WAC	-2.9209	6.5527	13.7991	2.03E-04	3.07E-02	0.1320
CEP72	4.4509	0.8110	13.7767	2.06E-04	3.07E-02	21.8708
NUDT4	-2.1448	8.7856	13.7650	2.07E-04	3.07E-02	0.2261
NDUFA5	-3.1834	5.7380	13.7348	2.11E-04	3.07E-02	0.1101
CLN5	4.0922	0.9086	13.7325	2.11E-04	3.07E-02	17.0562
SH3D19	-2.8230	6.5792	13.6991	2.15E-04	3.07E-02	0.1413
ACAP3*	3.8667	1.3390	13.6768	2.17E-04	3.07E-02	14.5876
NPM1P4	4.0017	1.2595	13.6712	2.18E-04	3.07E-02	16.0190
C11orf49	4.2268	1.4874	13.6704	2.18E-04	3.07E-02	18.7239
RP11-445O3.3*	4.0930	1.0503	13.6379	2.22E-04	3.07E-02	17.0659
RP11-565F19.2	3.4096	0.6265	13.6247	2.23E-04	3.07E-02	10.6268
AARS	4.2334	1.5132	13.4935	2.39E-04	3.25E-02	18.8099
XKR4*	5.2123	2.7999	13.4722	2.42E-04	3.25E-02	37.0736
IGFBP5	3.7572	1.4216	13.3858	2.54E-04	3.37E-02	13.5214
RP11-888D10.4	5.3603	1.3633	13.3130	2.64E-04	3.46E-02	41.0790

UQCRB	-2.9087	6.1866	13.2723	2.69E-04	3.50E-02	0.1332
KNTC1	3.9931	1.0764	13.1637	2.85E-04	3.65E-02	15.9236
GLE1	-6.4319	4.6756	13.1533	2.87E-04	3.65E-02	0.0116
TRIM6	-5.0518	2.8793	13.0692	3.00E-04	3.77E-02	0.0301
KIAA1841*	3.7393	2.3484	13.0516	3.03E-04	3.77E-02	13.3547
TMEM161B	2.8030	4.1961	12.9420	3.21E-04	3.92E-02	6.9790
ZBED1	3.7469	3.7916	12.9374	3.22E-04	3.92E-02	13.4254
CHRM2	5.3480	1.8069	12.8546	3.37E-04	4.02E-02	40.7288
RP11-102M11.2	2.8825	0.6955	12.8431	3.39E-04	4.02E-02	7.3744
FCRL5	3.3146	0.6318	12.8312	3.41E-04	4.02E-02	9.9494
AC005537.2	3.4779	1.0791	12.7502	3.56E-04	4.16E-02	11.1419
PLEKHM1	3.7830	1.7056	12.7013	3.65E-04	4.20E-02	13.7658
NPM1P25	3.4037	0.6602	12.6817	3.69E-04	4.20E-02	10.5835
EID2B	4.1059	0.8719	12.6604	3.73E-04	4.20E-02	17.2190
CHP2	-3.3183	4.5616	12.6451	3.77E-04	4.20E-02	0.1003
PI4KA*	3.6989	1.0550	12.6272	3.80E-04	4.20E-02	12.9857
RP11-401P9.6	3.2522	0.6668	12.6134	3.83E-04	4.20E-02	9.5281
CD63	-2.1695	5.9462	12.5855	3.89E-04	4.20E-02	0.2223
SMS	-2.5002	8.8068	12.5709	3.92E-04	4.20E-02	0.1767
ZSWIM4	3.1488	6.5142	12.5586	3.94E-04	4.20E-02	8.8690
MRPL19	4.7913	3.0346	12.5510	3.96E-04	4.20E-02	27.6894
MTM1	-3.2429	3.9469	12.5174	4.03E-04	4.21E-02	0.1056
SETDB1	2.9549	0.7734	12.5137	4.04E-04	4.21E-02	7.7537
SRSF5	-3.0444	4.3771	12.4901	4.09E-04	4.21E-02	0.1212
NRCAM	3.7825	0.8471	12.4782	4.12E-04	4.21E-02	13.7608
FOXO3*	-2.6267	5.2326	12.4222	4.24E-04	4.30E-02	0.1619
POFUT2	2.9274	2.0494	12.3906	4.31E-04	4.34E-02	7.6076
CYP4B1	3.6301	1.1122	12.3642	4.38E-04	4.35E-02	12.3812
LAPTM4A	-2.7660	9.0607	12.3511	4.41E-04	4.35E-02	0.1470
CNOT2	-2.6600	5.2919	12.3194	4.48E-04	4.37E-02	0.1582
HECA	-2.7256	5.8736	12.2840	4.57E-04	4.37E-02	0.1512
POP5	4.0020	0.9919	12.2809	4.58E-04	4.37E-02	16.0218
KIAA0101*	3.3443	0.8733	12.2796	4.58E-04	4.37E-02	10.1560
PARL	-4.7270	4.3755	12.2204	4.73E-04	4.46E-02	0.0378
MIR1199	-5.2697	1.6221	12.2124	4.75E-04	4.46E-02	0.0259
MGAT4C*	4.2534	2.5802	12.1893	4.81E-04	4.48E-02	19.0716
SPTLC3*	3.1610	0.7713	12.1468	4.92E-04	4.49E-02	8.9446
DNAJC27*	4.7018	1.3558	12.1254	4.97E-04	4.49E-02	26.0237
UBXN1	-3.0643	2.4417	12.1112	5.01E-04	4.49E-02	0.1195
ERCC4	3.6967	2.0354	12.0922	5.06E-04	4.49E-02	12.9664
PRPSAP2	2.8534	2.2422	12.0703	5.12E-04	4.49E-02	7.2272
HDGFRP2	2.9052	2.6531	12.0549	5.17E-04	4.49E-02	7.4913
DCAF8	-3.0948	3.0608	12.0298	5.24E-04	4.49E-02	0.1171
RHOH	3.5446	2.0210	12.0297	5.24E-04	4.49E-02	11.6688
MTHFSD	-4.8003	1.0764	12.0289	5.24E-04	4.49E-02	0.0359
PRKAA1	-2.8988	5.0167	12.0220	5.26E-04	4.49E-02	0.1341
PROX1*	3.8586	2.3350	12.0118	5.29E-04	4.49E-02	14.5059

CTBP1	-2.8818	6.1859	12.0065	5.30E-04	4.49E-02	0.1357
ADORA2A-AS1	3.5729	0.8930	11.9990	5.32E-04	4.49E-02	11.9002
GSTO1	3.9892	1.6372	11.9646	5.42E-04	4.54E-02	15.8803
PPP1R2	-2.4834	3.6082	11.8712	5.70E-04	4.74E-02	0.1788
CATSPER2	4.4765	1.1054	11.8579	5.74E-04	4.74E-02	22.2626
TMEM132B*	4.0982	1.1740	11.8383	5.80E-04	4.76E-02	17.1274
SIAH3*	4.3062	0.9506	11.8034	5.91E-04	4.76E-02	19.7832
GDI2	-2.9317	7.3302	11.7850	5.97E-04	4.76E-02	0.1311
PPP1CB	-1.9915	11.0357	11.7796	5.99E-04	4.76E-02	0.2515
TXNRD1*	-2.2916	8.0706	11.7731	6.01E-04	4.76E-02	0.2043
PGS1	4.2567	1.1014	11.7713	6.02E-04	4.76E-02	19.1152
TRIM4	-4.5637	3.9131	11.7326	6.14E-04	4.80E-02	0.0423
GGPS1	-3.0995	5.6808	11.7299	6.15E-04	4.80E-02	0.1167
ZNF100	3.5210	1.0097	11.7120	6.21E-04	4.82E-02	11.4792
OSGIN1	5.1939	1.4185	11.6831	6.31E-04	4.86E-02	36.6034
KSR1	3.8470	0.9505	11.6705	6.35E-04	4.86E-02	14.3896
GLDN	-4.3722	1.0384	11.6103	6.56E-04	4.88E-02	0.0483
FAM222B*	-2.3316	6.7209	11.6062	6.57E-04	4.88E-02	0.1987
TMEM108	3.9425	1.1107	11.5889	6.63E-04	4.88E-02	15.3746
RAB18	-2.3045	5.6872	11.5802	6.67E-04	4.88E-02	0.2024
FAM133A*	3.0895	1.7707	11.5714	6.70E-04	4.88E-02	8.5119
ATP6V0E1	-1.8627	7.5789	11.5556	6.75E-04	4.88E-02	0.2750
FAM212B	3.7414	1.1405	11.5533	6.76E-04	4.88E-02	13.3748
RPGR*	4.5634	3.0035	11.5454	6.79E-04	4.88E-02	23.6435
TMEM167B	-2.9407	3.6647	11.5341	6.83E-04	4.88E-02	0.1302
RHOBTB1	-4.4411	3.3948	11.5284	6.85E-04	4.88E-02	0.0460
MPHOSPH9	4.7324	1.3601	11.5220	6.88E-04	4.88E-02	26.5831
FRAS1	3.5866	0.8666	11.5111	6.92E-04	4.88E-02	12.0138
SGOL2	5.8117	1.2415	11.5021	6.95E-04	4.88E-02	56.1695
AC114498.1	2.7350	1.6236	11.4972	6.97E-04	4.88E-02	6.6574

Table S4. Low-ENL DE Genes. Differentially expressed (DE) genes in the contrast flaxseed lignan supplement vs placebo treatment in low-ENL excreters, sorted by their respective FDR.
Abbreviations: logFC, log fold change; logCPM, log counts per million; LR, likelihood ratio; FDR, False Discovery Rate corrected p-values; FC, fold change.
*Genes that also appear as DE in the high ENL participants with the same directionality (upregulation/downregulation).

	**logFC**	**logCPM**	**LR**	**p-Value**	**FDR**	**FC**
OTOP2	-5.2307	4.4961	37.4709	9.28E-10	1.07E-05	0.0266
RP11-217O12.1	5.8100	2.1304	30.5429	3.27E-08	1.89E-04	56.1032
SIRT5	2.8775	0.8518	26.5376	2.58E-07	8.60E-04	7.3486
CHMP4C	4.2710	3.2839	26.0712	3.29E-07	8.60E-04	19.3057
HERC6	3.6319	0.9521	25.7585	3.87E-07	8.60E-04	12.3970
PTCHD1	5.6572	1.6304	25.4816	4.47E-07	8.60E-04	50.4629
CD93	3.5470	0.8208	25.0248	5.66E-07	9.34E-04	11.6882
ACADSB	-4.5375	1.0660	24.1622	8.86E-07	1.28E-03	0.0431
UBR1	5.5486	1.5936	23.7907	1.07E-06	1.36E-03	46.8054
ANKLE1	3.5713	0.9636	23.6083	1.18E-06	1.36E-03	11.8870
STPG1	5.3562	1.5952	23.3188	1.37E-06	1.44E-03	40.9611
GPC2	3.8837	1.2120	22.9002	1.71E-06	1.49E-03	14.7610
SKI	-3.9048	4.7478	22.7352	1.86E-06	1.49E-03	0.0668
FKBP5	-5.5872	3.9782	22.6707	1.92E-06	1.49E-03	0.0208
OTX2-AS1	5.5594	1.5793	22.5448	2.05E-06	1.49E-03	47.1576
CIITA	4.5324	1.1617	22.5417	2.06E-06	1.49E-03	23.1408
NPTX1	5.1805	1.2975	22.3883	2.23E-06	1.49E-03	36.2641
RP11-6N17.4	4.6828	2.3832	22.2549	2.39E-06	1.49E-03	25.6833
COL1A2	6.6847	2.1134	22.2009	2.46E-06	1.49E-03	102.8732
RBP4	-3.3714	0.8125	21.9789	2.76E-06	1.52E-03	0.0966
LDOC1L	-4.5939	1.3650	21.9646	2.78E-06	1.52E-03	0.0414
SAE1	4.3707	1.0658	21.8554	2.94E-06	1.52E-03	20.6877
SRP68	-3.6917	1.0332	21.7950	3.03E-06	1.52E-03	0.0774
FEZ1	3.4321	0.8161	21.6475	3.28E-06	1.56E-03	10.7939
RUNX1T1	5.7551	2.2500	21.4876	3.56E-06	1.56E-03	54.0071
RP11-583F2.2	4.3462	1.0840	21.4846	3.57E-06	1.56E-03	20.3391
TPD52L1	3.0407	0.7469	21.3875	3.75E-06	1.56E-03	8.2287
FEM1A	4.2457	1.4310	21.3359	3.85E-06	1.56E-03	18.9709
PECAM1	4.0708	1.0050	21.3016	3.92E-06	1.56E-03	16.8051
LINC00560	4.0314	1.2525	21.1457	4.26E-06	1.64E-03	16.3522
CCDC117	-3.8419	5.1205	20.8759	4.90E-06	1.83E-03	0.0697
TMEM132B*	4.6368	1.1740	20.7844	5.14E-06	1.86E-03	24.8786
RP5-1198O20.4	4.7441	1.3064	20.7049	5.36E-06	1.88E-03	26.7983
MUC19	5.4532	1.5954	20.6082	5.64E-06	1.88E-03	43.8114
RP4-569M23.5	4.5351	0.9439	20.5849	5.70E-06	1.88E-03	23.1839

SLC38A7	5.3221	1.4334	20.4908	5.99E-06	1.88E-03	40.0039
ZNF613	2.9859	0.8087	20.4815	6.02E-06	1.88E-03	7.9224
PIM2	-5.5573	4.5733	20.3873	6.32E-06	1.92E-03	0.0212
SLC16A10	4.1722	1.0532	20.1914	7.01E-06	2.08E-03	18.0285
RPL32P3	3.1068	0.9208	20.1419	7.19E-06	2.08E-03	8.6146
ATG2A	-3.5956	3.8326	20.0691	7.47E-06	2.11E-03	0.0827
KLHL18	-3.8090	1.3592	19.7180	8.97E-06	2.45E-03	0.0713
TNPO3	-4.7615	3.3196	19.6922	9.10E-06	2.45E-03	0.0369
CACNG2	4.2627	1.3020	19.4472	1.03E-05	2.72E-03	19.1950
RNF14	-4.2243	5.2797	19.3492	1.09E-05	2.80E-03	0.0535
CTC-384G19.1	2.9732	0.7000	19.2101	1.17E-05	2.90E-03	7.8527
GNAO1	5.3704	2.8183	19.1701	1.20E-05	2.90E-03	41.3675
WDYHV1	4.4661	0.9322	19.1274	1.22E-05	2.90E-03	22.1019
WFDC1	3.3747	0.7758	19.0835	1.25E-05	2.90E-03	10.3723
PGBD4	3.5314	1.1288	19.0527	1.27E-05	2.90E-03	11.5628
NCBP2-AS2	-4.4936	1.2599	19.0398	1.28E-05	2.90E-03	0.0444
C6orf222	-3.7951	5.7969	18.9825	1.32E-05	2.93E-03	0.0720
SCN8A	2.9110	0.6725	18.9307	1.36E-05	2.93E-03	7.5215
AGO1	-4.5822	5.8383	18.9095	1.37E-05	2.93E-03	0.0417
OR2H1	6.2544	2.1540	18.8478	1.42E-05	2.93E-03	76.3430
BAZ1B	-3.3347	4.2225	18.8416	1.42E-05	2.93E-03	0.0991
MEP1B	-4.5641	1.5769	18.7942	1.46E-05	2.95E-03	0.0423
IVD	3.9897	0.8282	18.7020	1.53E-05	3.05E-03	15.8859
FAM222B*	-3.0026	6.7209	18.6379	1.58E-05	3.08E-03	0.1248
DLEU1	3.4114	0.8634	18.6178	1.60E-05	3.08E-03	10.6400
JAKMIP1	4.4087	2.7634	18.5003	1.70E-05	3.08E-03	21.2395
C11orf30	4.5329	1.7966	18.4685	1.73E-05	3.08E-03	23.1497
DSCAM	3.7802	0.8846	18.4601	1.74E-05	3.08E-03	13.7390
GREB1	4.3922	1.3018	18.4386	1.75E-05	3.08E-03	20.9987
MALRD1	2.7590	0.7200	18.4350	1.76E-05	3.08E-03	6.7694
RP11-157L3.4	4.8304	1.2296	18.4325	1.76E-05	3.08E-03	28.4500
SLC1A2	3.5694	0.9411	18.3590	1.83E-05	3.16E-03	11.8712
TENM4	3.7079	0.9708	18.1927	2.00E-05	3.35E-03	13.0674
MKL2	-4.4562	2.6087	18.1872	2.00E-05	3.35E-03	0.0456
KYNU	3.9582	1.2881	18.0888	2.11E-05	3.48E-03	15.5426
UTP23	6.9483	2.2594	18.0590	2.14E-05	3.49E-03	123.4957
JAG1	5.2154	4.5756	17.9664	2.25E-05	3.49E-03	37.1533
ZC3H8	3.0884	0.9502	17.9123	2.31E-05	3.49E-03	8.5055
SDHA	3.5545	1.3209	17.9006	2.33E-05	3.49E-03	11.7490
RP11-295K2.3	4.7979	1.3663	17.8927	2.34E-05	3.49E-03	27.8179
PITPNC1	4.4124	1.4335	17.8846	2.35E-05	3.49E-03	21.2936

IFI16	4.9818	3.9500	17.8655	2.37E-05	3.49E-03	31.5991
AC109357.1	4.6067	1.2466	17.8534	2.39E-05	3.49E-03	24.3640
SLC9A7	4.8620	1.3805	17.8516	2.39E-05	3.49E-03	29.0806
CD300E	4.8984	1.9053	17.8056	2.45E-05	3.52E-03	29.8231
ZNF346	2.8152	0.7430	17.7636	2.50E-05	3.52E-03	7.0384
GNPDA2	3.9283	1.1722	17.7305	2.55E-05	3.52E-03	15.2240
C11orf83	-3.2704	1.3653	17.7277	2.55E-05	3.52E-03	0.1036
DDX11	3.3008	0.8269	17.7055	2.58E-05	3.52E-03	9.8548
NABP1	2.7255	5.8385	17.6958	2.59E-05	3.52E-03	6.6140
ZNF567	4.3232	0.9272	17.6760	2.62E-05	3.52E-03	20.0174
SORBS1	-3.3324	4.1719	17.6353	2.68E-05	3.55E-03	0.0993
KIAA1683	4.8291	1.0941	17.4476	2.95E-05	3.80E-03	28.4252
PGBD2	-3.9887	1.4205	17.4428	2.96E-05	3.80E-03	0.0630
NAE1	3.6515	1.2432	17.4318	2.98E-05	3.80E-03	12.5662
NMNAT2	4.0087	1.2252	17.3799	3.06E-05	3.80E-03	16.0964
FAM19A2	4.9208	1.0683	17.3585	3.10E-05	3.80E-03	30.2900
AC079776.3	3.9420	1.0386	17.3107	3.17E-05	3.80E-03	15.3698
SEZ6L2	3.7386	1.3442	17.2783	3.23E-05	3.80E-03	13.3480
CYB5D2	-4.2619	0.9507	17.2503	3.28E-05	3.80E-03	0.0521
MFN2	-3.3498	4.6943	17.2472	3.28E-05	3.80E-03	0.0981
ZNF318	4.7453	1.8838	17.2293	3.31E-05	3.80E-03	26.8208
TMEM126A	-6.0603	2.1213	17.2251	3.32E-05	3.80E-03	0.0150
NUSAP1	4.2050	1.0206	17.2089	3.35E-05	3.80E-03	18.4424
WIPF3	2.7859	0.9127	17.2046	3.36E-05	3.80E-03	6.8966
IPO7	2.8896	5.6632	17.1982	3.37E-05	3.80E-03	7.4108
INTU	5.7403	2.4303	17.1898	3.38E-05	3.80E-03	53.4558
WNT4	3.4839	1.0804	17.1895	3.38E-05	3.80E-03	11.1883
ELFN1	5.6731	2.0592	17.1704	3.42E-05	3.80E-03	51.0238
MARCH4	3.9313	1.0179	17.0707	3.60E-05	3.89E-03	15.2556
PPP1R18	4.6449	1.4936	17.0671	3.61E-05	3.89E-03	25.0186
TSSC1	3.4818	0.7854	17.0669	3.61E-05	3.89E-03	11.1721
CNKSR1	5.6834	3.1468	17.0520	3.64E-05	3.89E-03	51.3904
FAM122C	3.1508	0.7761	16.9585	3.82E-05	4.03E-03	8.8815
TMEM221	2.9622	0.6320	16.9507	3.84E-05	4.03E-03	7.7930
ERGIC3	-3.2348	4.9915	16.9272	3.88E-05	4.04E-03	0.1062
ZNF793	5.9580	2.9863	16.8464	4.05E-05	4.11E-03	62.1658
DPY19L4	3.3081	0.7569	16.8065	4.14E-05	4.11E-03	9.9043
RAPGEF4	4.0070	1.6945	16.8049	4.14E-05	4.11E-03	16.0773
ATP6V0A2	-3.9884	3.7034	16.8026	4.15E-05	4.11E-03	0.0630
C15orf57	5.3535	2.2870	16.7919	4.17E-05	4.11E-03	40.8856
CTD-2349P21.11	5.2367	1.2636	16.7853	4.19E-05	4.11E-03	37.7046

CDH12	2.6904	0.6918	16.7682	4.22E-05	4.11E-03	6.4550
SLC22A9	4.0951	0.9546	16.7522	4.26E-05	4.11E-03	17.0905
PLEKHA2	4.5989	3.0813	16.7432	4.28E-05	4.11E-03	24.2334
MAS1	4.2563	1.0264	16.7323	4.30E-05	4.11E-03	19.1109
SYCP1	3.6813	0.8403	16.6907	4.40E-05	4.14E-03	12.8283
NUP93	2.8808	0.8203	16.6893	4.40E-05	4.14E-03	7.3653
RP11-467L13.7	4.6345	5.1442	16.6708	4.45E-05	4.14E-03	24.8386
KMT2C	2.4902	6.2329	16.6414	4.52E-05	4.17E-03	5.6184
NFE2L3	2.8139	0.8079	16.5838	4.65E-05	4.24E-03	7.0318
ONECUT3	5.7957	2.6667	16.5303	4.79E-05	4.24E-03	55.5496
SNORA31	7.9558	3.1357	16.5119	4.83E-05	4.24E-03	248.2681
LINC01183	4.4191	1.1829	16.5096	4.84E-05	4.24E-03	21.3930
AP000704.5	3.1716	0.7337	16.5048	4.85E-05	4.24E-03	9.0103
HDHD2	4.5795	1.0587	16.4990	4.87E-05	4.24E-03	23.9095
RP11-296E23.2	4.4480	1.1127	16.4983	4.87E-05	4.24E-03	21.8257
POLI	3.8140	0.8836	16.4956	4.88E-05	4.24E-03	14.0650
RP4-724E16.2	3.8261	0.8053	16.4679	4.95E-05	4.27E-03	14.1833
SHPRH	4.7023	1.4993	16.4057	5.11E-05	4.38E-03	26.0334
AMER1	4.8636	1.4893	16.3774	5.19E-05	4.39E-03	29.1130
CTB-43E15.3	3.4050	0.9928	16.3728	5.20E-05	4.39E-03	10.5927
RP11-108M9.3	3.0283	0.6631	16.3330	5.31E-05	4.45E-03	8.1587
MROH7	3.6736	1.1906	16.3092	5.38E-05	4.46E-03	12.7608
RANBP17	3.6905	1.3885	16.2946	5.42E-05	4.46E-03	12.9107
RP11-38O23.4	3.4731	2.4869	16.2868	5.44E-05	4.46E-03	11.1047
RP11-445O3.3*	3.5824	1.0503	16.2084	5.67E-05	4.62E-03	11.9790
PMM1	-5.0968	3.6061	16.1780	5.77E-05	4.62E-03	0.0292
C1GALT1C1	-4.1592	2.2484	16.1683	5.80E-05	4.62E-03	0.0560
DDX6	-2.0588	7.8222	16.1574	5.83E-05	4.62E-03	0.2400
GDF11	4.0788	1.3202	16.1495	5.85E-05	4.62E-03	16.8988
RYR2	4.4814	1.9034	16.1308	5.91E-05	4.62E-03	22.3379
KIAA1257	4.5789	1.3540	16.1242	5.93E-05	4.62E-03	23.8991
C9orf37	3.1362	0.7452	16.1149	5.96E-05	4.62E-03	8.7920
TRADD	-4.0502	1.0865	16.0967	6.02E-05	4.64E-03	0.0604
SCRN1	3.9978	1.1242	16.0774	6.08E-05	4.64E-03	15.9754
CTC-559E9.5	5.6768	1.7305	16.0686	6.11E-05	4.64E-03	51.1537
DPYD	4.9155	1.6859	16.0533	6.16E-05	4.65E-03	30.1802
MYBPC1	4.9776	1.5176	16.0390	6.21E-05	4.65E-03	31.5063
CDH19	5.2503	2.1142	16.0305	6.23E-05	4.65E-03	38.0615
MYH10*	3.2098	1.1666	16.0116	6.30E-05	4.66E-03	9.2523
NRXN3	4.2113	1.7591	16.0007	6.33E-05	4.66E-03	18.5239
ANAPC1	4.3394	1.0232	15.9616	6.46E-05	4.71E-03	20.2431

GGT7	3.3052	0.9598	15.9560	6.48E-05	4.71E-03	9.8846
SIGLEC14	-5.3312	3.2337	15.9185	6.61E-05	4.72E-03	0.0248
ZC4H2	5.5087	1.4414	15.9174	6.62E-05	4.72E-03	45.5280
C10orf67	3.4498	1.2413	15.9065	6.65E-05	4.72E-03	10.9271
PIEZO2	4.5461	1.1888	15.9043	6.66E-05	4.72E-03	23.3614
RNF24	3.7829	2.6269	15.8710	6.78E-05	4.78E-03	13.7651
NAIP	3.6749	0.9062	15.8223	6.96E-05	4.87E-03	12.7718
CACNG3	3.6993	1.0513	15.7731	7.14E-05	4.97E-03	12.9899
TGM2	2.9674	0.8378	15.7231	7.33E-05	5.04E-03	7.8210
SCGB2B2	3.5186	1.1293	15.7163	7.36E-05	5.04E-03	11.4603
PHF14	5.0628	2.0490	15.7008	7.42E-05	5.04E-03	33.4248
KLF12	2.9654	0.7244	15.6898	7.46E-05	5.04E-03	7.8102
RP11-179A10.2	3.3421	0.6653	15.6893	7.46E-05	5.04E-03	10.1409
ARHGAP10	4.6480	1.0998	15.6450	7.64E-05	5.13E-03	25.0727
MTCO3P1	-7.3367	2.3695	15.6257	7.72E-05	5.14E-03	0.0062
NUP210	3.9965	0.9950	15.6165	7.76E-05	5.14E-03	15.9617
CACNA2D1	4.3309	1.0147	15.6109	7.78E-05	5.14E-03	20.1247
KIF20B*	3.0504	1.0094	15.5998	7.83E-05	5.14E-03	8.2842
COL28A1	3.1459	0.6928	15.5572	8.00E-05	5.17E-03	8.8515
FERMT2	3.0857	0.6912	15.5434	8.06E-05	5.17E-03	8.4898
PTPRS	3.6249	1.8201	15.5411	8.07E-05	5.17E-03	12.3367
NEB	2.9854	1.0972	15.5344	8.10E-05	5.17E-03	7.9195
NOS2	4.2387	1.1026	15.5270	8.13E-05	5.17E-03	18.8790
CLUAP1	4.7907	1.3467	15.5236	8.15E-05	5.17E-03	27.6788
CSMD3	4.5173	1.1751	15.5101	8.21E-05	5.18E-03	22.9003
PSMC3	3.5052	1.7274	15.4944	8.27E-05	5.20E-03	11.3544
MOSPD3	2.9276	0.8387	15.4794	8.34E-05	5.21E-03	7.6083
RP11-430C1.2	2.8944	0.7771	15.4369	8.53E-05	5.30E-03	7.4352
MYCBPAP	3.9448	0.8961	15.4079	8.66E-05	5.35E-03	15.3993
KIF5C	4.5135	3.0546	15.3745	8.82E-05	5.38E-03	22.8401
BEST4	-4.0470	1.2244	15.3566	8.90E-05	5.38E-03	0.0605
RP4-639F20.1	3.6634	0.7633	15.3542	8.91E-05	5.38E-03	12.6704
CADPS	4.7960	2.2523	15.3505	8.93E-05	5.38E-03	27.7797
RP11-315A16.1	4.0212	0.9810	15.3481	8.94E-05	5.38E-03	16.2372
PPEF1	3.7501	0.8278	15.3326	9.01E-05	5.40E-03	13.4553
PTK7	4.2440	0.9204	15.3219	9.07E-05	5.40E-03	18.9487
AVL9	3.1422	3.5953	15.3007	9.17E-05	5.43E-03	8.8285
MYPN	-4.3098	1.1131	15.2541	9.40E-05	5.53E-03	0.0504
DBNL	-2.9564	6.8255	15.2443	9.45E-05	5.53E-03	0.1288
HEATR3	2.6042	0.7579	15.2398	9.47E-05	5.53E-03	6.0807
TRIM3	3.4306	1.3220	15.2142	9.60E-05	5.57E-03	10.7821

ANO4	3.0810	0.6461	15.1854	9.75E-05	5.63E-03	8.4619
CACNB4	4.3542	1.0960	15.1319	1.00E-04	5.74E-03	20.4520
DNM3OS	4.3245	2.4476	15.1301	1.00E-04	5.74E-03	20.0360
LYPLAL1	4.8044	1.7287	15.1164	1.01E-04	5.75E-03	27.9425
CTC-207P7.1	3.6366	0.8305	15.1003	1.02E-04	5.78E-03	12.4377
RP11-369K17.1	4.4447	1.0093	15.0849	1.03E-04	5.79E-03	21.7761
ST7L	3.3382	0.7781	15.0773	1.03E-04	5.79E-03	10.1134
RP1-274L7.4	3.5369	1.0350	15.0593	1.04E-04	5.79E-03	11.6071
HMG20B	4.5513	1.2237	15.0502	1.05E-04	5.79E-03	23.4466
NPR3	3.0577	0.8374	15.0449	1.05E-04	5.79E-03	8.3263
MAPK6	-2.2731	7.4329	15.0393	1.05E-04	5.79E-03	0.2069
LRP4-AS1	3.5141	0.9228	15.0000	1.08E-04	5.86E-03	11.4251
RP11-637A17.2	4.1134	0.9976	14.9992	1.08E-04	5.86E-03	17.3087
ACAP3*	3.5371	1.3390	14.9757	1.09E-04	5.87E-03	11.6086
MUL1	-3.7682	5.8426	14.9781	1.09E-04	5.87E-03	0.0734
GTDC1	5.7224	2.9773	14.9715	1.09E-04	5.87E-03	52.7983
AC116366.6	2.6614	0.9501	14.9608	1.10E-04	5.87E-03	6.3264
ZNF181	5.6063	1.8379	14.9384	1.11E-04	5.92E-03	48.7140
PPP5D1	3.2608	0.9467	14.9251	1.12E-04	5.92E-03	9.5854
NRN1	3.6272	0.7780	14.9182	1.12E-04	5.92E-03	12.3562
YIPF4	2.5418	6.0313	14.9034	1.13E-04	5.94E-03	5.8233
CTB-111H14.1	4.0536	1.1343	14.8930	1.14E-04	5.95E-03	16.6055
MED12L	4.1383	0.9716	14.8845	1.14E-04	5.95E-03	17.6100
TMEM170A	-2.6796	4.6863	14.8706	1.15E-04	5.95E-03	0.1561
C15orf41	3.3509	3.0892	14.8618	1.16E-04	5.95E-03	10.2030
HELB	3.2755	0.8562	14.8594	1.16E-04	5.95E-03	9.6833
RALGPS1	5.0891	2.3858	14.8386	1.17E-04	5.99E-03	34.0377
HUWE1	-3.2119	6.5856	14.8279	1.18E-04	6.00E-03	0.1079
ANK1	3.8407	0.7653	14.8192	1.18E-04	6.00E-03	14.3269
RINL	3.8336	0.9552	14.7828	1.21E-04	6.08E-03	14.2565
NOVA2	6.3502	5.5779	14.7772	1.21E-04	6.08E-03	81.5844
IGF2	3.9037	1.9587	14.7515	1.23E-04	6.12E-03	14.9664
COL1A1	4.9831	3.0188	14.7474	1.23E-04	6.12E-03	31.6283
FOXD2-AS1	-4.7628	1.7948	14.6800	1.27E-04	6.28E-03	0.0368
VEZT	-3.0955	3.0156	14.6760	1.28E-04	6.28E-03	0.1170
RNF165	4.5498	1.7821	14.6727	1.28E-04	6.28E-03	23.4220
MTR	5.2052	2.7740	14.6594	1.29E-04	6.28E-03	36.8909
ARAP1	5.0164	1.4605	14.6529	1.29E-04	6.28E-03	32.3666
RP11-467D6.1	4.5751	1.9019	14.6523	1.29E-04	6.28E-03	23.8370
FAM212B*	3.2582	1.1405	14.6225	1.31E-04	6.33E-03	9.5681
BRIX1	4.3756	0.9158	14.6206	1.31E-04	6.33E-03	20.7575

EXOSC4	-2.7005	1.1264	14.5897	1.34E-04	6.41E-03	0.1538
CTD-2540F13.2	3.6531	1.2367	14.5755	1.35E-04	6.42E-03	12.5806
MX2	3.7876	2.3653	14.5716	1.35E-04	6.42E-03	13.8096
PELO	2.3239	0.9260	14.5636	1.36E-04	6.42E-03	5.0070
DLGAP2	4.6459	1.1403	14.5478	1.37E-04	6.42E-03	25.0350
RP11-702H23.4	3.9409	1.0111	14.5466	1.37E-04	6.42E-03	15.3582
RNLS	-4.2560	2.6139	14.5236	1.38E-04	6.45E-03	0.0523
MBD5	4.0593	1.0013	14.5152	1.39E-04	6.45E-03	16.6716
RASGEF1A	3.0352	0.9205	14.5107	1.39E-04	6.45E-03	8.1975
ALKBH2	5.3287	1.4198	14.5088	1.40E-04	6.45E-03	40.1868
ACE	3.3038	1.1008	14.4888	1.41E-04	6.47E-03	9.8754
UQCC1	3.7884	1.0692	14.4830	1.41E-04	6.47E-03	13.8169
CCDC88A	4.6572	2.0545	14.4791	1.42E-04	6.47E-03	25.2320
CD28	3.3316	0.7604	14.4606	1.43E-04	6.51E-03	10.0675
COQ5	2.6403	0.8363	14.4418	1.45E-04	6.53E-03	6.2347
RP11-266L9.2	3.8725	0.9380	14.4387	1.45E-04	6.53E-03	14.6472
CASS4	3.5546	1.1297	14.4340	1.45E-04	6.53E-03	11.7504
TMF1	-2.9172	5.3879	14.4026	1.48E-04	6.57E-03	0.1324
BEST2	-4.9653	2.2313	14.3974	1.48E-04	6.57E-03	0.0320
FZD3	3.9618	2.4536	14.3875	1.49E-04	6.57E-03	15.5820
OLR1	-3.6267	5.4906	14.3816	1.49E-04	6.57E-03	0.0810
CD1A	5.5848	1.5779	14.3795	1.49E-04	6.57E-03	47.9948
LINC01197	3.5002	0.9416	14.3785	1.50E-04	6.57E-03	11.3150
EVC2	4.9501	1.0229	14.3674	1.50E-04	6.57E-03	30.9113
PLAG1	4.3632	1.6223	14.3581	1.51E-04	6.57E-03	20.5798
ZNF140	3.8700	0.8188	14.3563	1.51E-04	6.57E-03	14.6214
KIAA0319	3.5011	0.9958	14.3408	1.53E-04	6.60E-03	11.3221
L3MBTL4	3.5094	0.8587	14.3298	1.53E-04	6.62E-03	11.3878
VMA21	-4.4390	1.3665	14.2965	1.56E-04	6.69E-03	0.0461
CREB5	4.3403	2.5211	14.2961	1.56E-04	6.69E-03	20.2558
CLMN	-2.9098	5.0067	14.2690	1.58E-04	6.76E-03	0.1331
FXYD6	5.0078	1.1866	14.2568	1.59E-04	6.77E-03	32.1742
RP11-61L19.1	4.5266	1.2297	14.2435	1.61E-04	6.77E-03	23.0492
SLC26A4-AS1	3.4784	1.1730	14.2415	1.61E-04	6.77E-03	11.1456
FAT3	3.7661	2.2001	14.2370	1.61E-04	6.77E-03	13.6055
IGF1R	3.4273	1.5140	14.2024	1.64E-04	6.79E-03	10.7581
PCBD2	4.8086	2.0108	14.2005	1.64E-04	6.79E-03	28.0247
NANOG	4.6547	1.0439	14.1838	1.66E-04	6.79E-03	25.1885
AC025284.1	5.0678	1.5575	14.1732	1.67E-04	6.79E-03	33.5389
STK33	3.5074	1.0705	14.1690	1.67E-04	6.79E-03	11.3722
ZKSCAN5	2.5328	0.7045	14.1683	1.67E-04	6.79E-03	5.7871

LYN	3.7697	3.7583	14.1666	1.67E-04	6.79E-03	13.6395
ANKRD17	-2.8973	4.6272	14.1655	1.67E-04	6.79E-03	0.1342
TUBGCP5	3.4791	0.7490	14.1631	1.68E-04	6.79E-03	11.1510
MIR17HG	3.0262	0.7829	14.1508	1.69E-04	6.79E-03	8.1466
ZNF496	4.9141	1.5336	14.1480	1.69E-04	6.79E-03	30.1511
COL19A1	3.2042	0.9617	14.1447	1.69E-04	6.79E-03	9.2163
CDK5RAP2	5.0035	1.8763	14.1438	1.69E-04	6.79E-03	32.0787
AP000708.1	5.6077	2.0461	14.1398	1.70E-04	6.79E-03	48.7616
LHX5	4.2484	1.5417	14.1298	1.71E-04	6.80E-03	19.0061
RP11-664H7.2	4.5992	1.1754	14.1187	1.72E-04	6.82E-03	24.2379
CCDC135	4.0108	1.0185	14.0563	1.77E-04	6.98E-03	16.1198
RP11-354K4.1	2.4249	0.7147	14.0552	1.78E-04	6.98E-03	5.3701
CXCL11	5.2178	3.5152	14.0510	1.78E-04	6.98E-03	37.2140
AP1S2	-3.2928	3.8951	14.0475	1.78E-04	6.98E-03	0.1020
CYTH3	3.4656	0.8024	14.0393	1.79E-04	6.99E-03	11.0471
FMO1	3.5742	1.7214	14.0156	1.81E-04	6.99E-03	11.9109
STK24	4.0898	1.6061	14.0136	1.81E-04	6.99E-03	17.0275
TMEM138	3.6601	1.2359	14.0021	1.83E-04	6.99E-03	12.6413
POLD3	2.8251	2.7621	14.0018	1.83E-04	6.99E-03	7.0865
LSAMP	4.2183	1.3798	14.0015	1.83E-04	6.99E-03	18.6137
RP11-159H10.3	3.5300	1.1173	14.0002	1.83E-04	6.99E-03	11.5512
RP11-1C8.5	4.5480	1.5300	13.9922	1.84E-04	6.99E-03	23.3930
FMO4	-4.1353	1.1098	13.9824	1.85E-04	6.99E-03	0.0569
ANKRD26	4.4219	1.1097	13.9790	1.85E-04	6.99E-03	21.4349
RP11-82O19.1	5.3787	1.9478	13.9767	1.85E-04	6.99E-03	41.6045
SLC16A1-AS1	3.5448	3.0792	13.9602	1.87E-04	7.03E-03	11.6702
XKR4*	5.1157	2.7999	13.9326	1.89E-04	7.06E-03	34.6715
GFOD1	3.4463	1.8775	13.9389	1.89E-04	7.06E-03	10.9007
FANCE	3.2295	0.8866	13.9327	1.89E-04	7.06E-03	9.3797
NAV1	4.9857	4.8321	13.9219	1.91E-04	7.08E-03	31.6842
EGR3	3.5201	6.5915	13.9040	1.92E-04	7.13E-03	11.4727
CACNG8*	3.9063	1.6536	13.8691	1.96E-04	7.19E-03	14.9935
RP11-410C4.5	3.1399	0.6855	13.8808	1.95E-04	7.19E-03	8.8147
HIPK3	-3.3028	6.3631	13.8749	1.95E-04	7.19E-03	0.1013
CHD5	3.1453	0.7519	13.8571	1.97E-04	7.21E-03	8.8479
MAD1L1	3.6872	0.9190	13.8074	2.03E-04	7.35E-03	12.8817
NTRK3	4.6671	2.3416	13.8042	2.03E-04	7.35E-03	25.4066
NCS1	4.3215	1.8618	13.8034	2.03E-04	7.35E-03	19.9939
WDR87	6.9593	7.9770	13.7929	2.04E-04	7.36E-03	124.4427
RP11-515O17.3	3.7081	1.7730	13.7909	2.04E-04	7.36E-03	13.0688
AGER	-3.9688	0.8574	13.7652	2.07E-04	7.43E-03	0.0639

SMYD3	2.4830	0.6003	13.7566	2.08E-04	7.45E-03	5.5907
BMPR2	-2.6892	6.6002	13.7300	2.11E-04	7.49E-03	0.1550
RAB3D	-3.5485	3.5158	13.7284	2.11E-04	7.49E-03	0.0855
GPS2	3.4601	4.6375	13.7273	2.11E-04	7.49E-03	11.0054
NR2C1	3.7131	1.0810	13.7185	2.12E-04	7.51E-03	13.1143
RAI2	-4.6739	1.4352	13.7022	2.14E-04	7.52E-03	0.0392
CPED1	3.2122	0.8465	13.6965	2.15E-04	7.52E-03	9.2679
RP11-439E19.7	3.1787	0.9640	13.6912	2.15E-04	7.52E-03	9.0547
NUAK1	2.3712	0.6500	13.6912	2.15E-04	7.52E-03	5.1738
RPS6KA6	3.8847	1.8747	13.6609	2.19E-04	7.62E-03	14.7715
ATP7B	-3.6471	1.0002	13.6456	2.21E-04	7.64E-03	0.0798
CTD-2008L17.2	4.3444	1.8668	13.6449	2.21E-04	7.64E-03	20.3146
LGSN	4.4770	1.3718	13.6377	2.22E-04	7.65E-03	22.2699
BEND4	3.2641	0.9454	13.6303	2.23E-04	7.66E-03	9.6071
RP11-473P24.2	3.5975	2.2325	13.6172	2.24E-04	7.69E-03	12.1049
CASP4	3.4990	3.6283	13.5770	2.29E-04	7.83E-03	11.3059
IGHA1	3.5132	0.8297	13.5606	2.31E-04	7.87E-03	11.4176
IKZF4	4.7194	1.6931	13.5515	2.32E-04	7.89E-03	26.3431
RGMA	3.2984	0.9682	13.5464	2.33E-04	7.89E-03	9.8381
RP11-440G5.2	4.0637	0.8745	13.5246	2.35E-04	7.95E-03	16.7227
ZNF445	4.6256	1.8537	13.5117	2.37E-04	7.95E-03	24.6862
CTD-2566J3.1	-5.7590	3.4835	13.5070	2.38E-04	7.95E-03	0.0185
PCDHA4	3.5141	1.0231	13.5008	2.38E-04	7.95E-03	11.4247
LINC00998	-3.1558	2.6046	13.4894	2.40E-04	7.95E-03	0.1122
RP11-626H12.2	3.5449	1.0019	13.4893	2.40E-04	7.95E-03	11.6715
MAEL	3.1887	0.8732	13.4883	2.40E-04	7.95E-03	9.1179
PDE10A	3.6714	1.0463	13.4865	2.40E-04	7.95E-03	12.7407
ZSCAN30	2.9566	0.7568	13.4806	2.41E-04	7.95E-03	7.7630
MOSPD2	4.5899	4.0835	13.4778	2.41E-04	7.95E-03	24.0816
KIAA1147	-3.9200	1.4921	13.4607	2.44E-04	8.00E-03	0.0661
LINC01320	4.4752	1.2909	13.4486	2.45E-04	8.03E-03	22.2423
SLC22A11	4.9057	1.2064	13.4213	2.49E-04	8.11E-03	29.9746
WDR65	8.2175	5.9416	13.4168	2.49E-04	8.11E-03	297.6479
ASCC3	4.1096	1.7542	13.4127	2.50E-04	8.11E-03	17.2623
TMEM63A	4.2447	1.5663	13.4027	2.51E-04	8.11E-03	18.9575
HIBCH	2.9970	1.8046	13.4019	2.51E-04	8.11E-03	7.9833
YEATS4	2.6872	0.6890	13.3956	2.52E-04	8.11E-03	6.4407
MZT2A	4.0497	1.1278	13.3929	2.53E-04	8.11E-03	16.5604
FAM179A	4.3316	1.9675	13.3738	2.55E-04	8.13E-03	20.1341
ABCA12	4.3665	1.3102	13.3729	2.55E-04	8.13E-03	20.6281
TIMP3	4.7861	1.1397	13.3709	2.56E-04	8.13E-03	27.5911

RP11-285E9.5	5.7780	1.9806	13.3675	2.56E-04	8.13E-03	54.8713
COL21A1	2.9607	0.7901	13.3428	2.59E-04	8.20E-03	7.7853
ARMC9	4.1384	1.3335	13.3416	2.60E-04	8.20E-03	17.6109
C17orf105	4.6240	1.5861	13.3280	2.61E-04	8.23E-03	24.6587
KATNA1	-3.2233	0.8512	13.2981	2.66E-04	8.33E-03	0.1071
RP11-216P16.8	-5.2057	1.1747	13.2950	2.66E-04	8.33E-03	0.0271
PRKAG1	3.8582	2.5100	13.2618	2.71E-04	8.46E-03	14.5023
RP11-654D12.3	3.4855	0.8469	13.2513	2.72E-04	8.49E-03	11.2006
ABCC4	6.1645	2.6441	13.2385	2.74E-04	8.51E-03	71.7320
SFI1	4.4274	1.5880	13.2352	2.75E-04	8.51E-03	21.5163
AC004076.5	2.7161	1.3366	13.2286	2.76E-04	8.51E-03	6.5708
SIPA1L1	5.1611	3.1637	13.2253	2.76E-04	8.51E-03	35.7801
SLC25A45	3.0747	1.0538	13.1920	2.81E-04	8.64E-03	8.4254
RP11-338E21.2	2.9053	0.8667	13.1846	2.82E-04	8.65E-03	7.4920
ITPR1	2.4653	0.6442	13.1805	2.83E-04	8.65E-03	5.5224
INHBA-AS1	3.6713	0.8947	13.1755	2.84E-04	8.65E-03	12.7402
PARVB	2.4208	0.6053	13.1636	2.85E-04	8.68E-03	5.3546
UHRF2	4.3799	2.1678	13.1553	2.87E-04	8.69E-03	20.8200
IGLON5	4.3513	1.2658	13.1515	2.87E-04	8.69E-03	20.4109
C1GALT1	2.9135	3.2152	13.1425	2.89E-04	8.71E-03	7.5346
SF3A3	4.6814	1.9348	13.1241	2.92E-04	8.77E-03	25.6594
EFR3B	4.3769	1.6105	13.1151	2.93E-04	8.78E-03	20.7774
RP11-214K3.24	4.6500	1.3755	13.1122	2.93E-04	8.78E-03	25.1068
NAPB	-4.9529	2.4958	13.0997	2.95E-04	8.82E-03	0.0323
AL136380.1	3.5018	2.1097	13.0821	2.98E-04	8.82E-03	11.3279
RP11-383H13.1	3.5369	1.3302	13.0756	2.99E-04	8.82E-03	11.6066
C14orf64	3.4882	1.6013	13.0732	3.00E-04	8.82E-03	11.2213
RP11-43F13.3	3.9976	1.3430	13.0728	3.00E-04	8.82E-03	15.9734
AGBL2	3.7528	1.1497	13.0720	3.00E-04	8.82E-03	13.4807
B3GALTL	3.0611	0.9057	13.0701	3.00E-04	8.82E-03	8.3460
ASPSCR1	2.3256	0.9529	13.0619	3.01E-04	8.82E-03	5.0126
SLC25A51	-3.4298	1.8917	13.0615	3.01E-04	8.82E-03	0.0928
TNFAIP2	3.4181	6.9092	13.0534	3.03E-04	8.84E-03	10.6897
STAG1	2.6655	0.8163	13.0389	3.05E-04	8.87E-03	6.3445
ILDR1	4.7429	1.8169	13.0373	3.05E-04	8.87E-03	26.7775
GRIP1	4.3312	1.1387	12.9986	3.12E-04	9.00E-03	20.1284
RCL1	3.9372	0.9452	12.9955	3.12E-04	9.00E-03	15.3179
DOCK7	2.4401	0.7404	12.9947	3.12E-04	9.00E-03	5.4267
JAK3	3.2134	1.1582	12.9870	3.14E-04	9.02E-03	9.2754
SEPT11	2.7665	5.8887	12.9669	3.17E-04	9.08E-03	6.8045
DDR1	-2.2170	4.8351	12.9604	3.18E-04	9.08E-03	0.2151

NPM3	5.3581	2.8083	12.9562	3.19E-04	9.08E-03	41.0151
FYN	3.3709	1.2739	12.9554	3.19E-04	9.08E-03	10.3453
RP11-109N23.5	2.8099	2.3910	12.9278	3.24E-04	9.19E-03	7.0123
HIST1H3D	7.4636	5.1277	12.9146	3.26E-04	9.22E-03	176.5133
NUP88	2.8259	0.8403	12.9127	3.26E-04	9.22E-03	7.0905
QSOX2	4.0329	1.0100	12.9009	3.28E-04	9.24E-03	16.3693
PDGFD	5.8555	1.7106	12.8994	3.29E-04	9.24E-03	57.9008
RP11-788M5.3	3.8596	0.9698	12.8839	3.31E-04	9.28E-03	14.5168
KRI1	2.8582	0.8075	12.8783	3.32E-04	9.28E-03	7.2510
CUX2	3.9329	1.4092	12.8754	3.33E-04	9.28E-03	15.2733
NCLN	-4.3382	2.1112	12.8735	3.33E-04	9.28E-03	0.0494
NAT2	2.6743	1.2741	12.8624	3.35E-04	9.28E-03	6.3831
RP11-64D24.4	4.8163	1.7451	12.8578	3.36E-04	9.28E-03	28.1750
CTD-2269F5.1	3.2003	0.9121	12.8571	3.36E-04	9.28E-03	9.1916
RP11-759A24.3	4.8001	1.0341	12.8549	3.37E-04	9.28E-03	27.8594
RP11-296A18.3	4.3558	1.3546	12.8416	3.39E-04	9.31E-03	20.4745
HMGB3P5	3.8981	1.2081	12.8390	3.39E-04	9.31E-03	14.9092
DDHD2	4.0079	1.3212	12.8338	3.40E-04	9.31E-03	16.0882
SCYL2	-2.5468	6.5706	12.8315	3.41E-04	9.31E-03	0.1711
UGT1A7	3.4428	0.8758	12.7999	3.47E-04	9.45E-03	10.8742
PLEKHH3	3.4954	1.1372	12.7793	3.50E-04	9.53E-03	11.2775
PPM1K	4.0158	2.7514	12.7757	3.51E-04	9.53E-03	16.1761
CNN1	5.0001	1.8403	12.7580	3.54E-04	9.59E-03	32.0012
TMTC1	3.6514	0.8218	12.7490	3.56E-04	9.62E-03	12.5657
PPIH	-3.9731	0.8318	12.7251	3.61E-04	9.71E-03	0.0637
ISL1	2.9982	0.7966	12.7235	3.61E-04	9.71E-03	7.9898
ZNF788	3.1332	0.9844	12.7150	3.63E-04	9.73E-03	8.7736
KCNJ16	4.1747	1.5172	12.6973	3.66E-04	9.80E-03	18.0593
NTNG1	4.0197	1.0932	12.6919	3.67E-04	9.80E-03	16.2203
RP11-335K5.2	3.8186	1.1753	12.6748	3.71E-04	9.86E-03	14.1099
TIGD6	3.8662	0.9340	12.6663	3.72E-04	9.86E-03	14.5826
EIF5	-2.3202	6.3340	12.6627	3.73E-04	9.86E-03	0.2002
KIAA1328	3.8841	1.0110	12.6577	3.74E-04	9.86E-03	14.7652
CSMD2	3.3476	0.9109	12.6554	3.74E-04	9.86E-03	10.1795
COL25A1	3.0976	0.9285	12.6550	3.75E-04	9.86E-03	8.5598
ROCK2	2.5654	4.7743	12.6509	3.75E-04	9.86E-03	5.9190
PMVK	-2.7730	0.9398	12.6377	3.78E-04	9.91E-03	0.1463
PHF11	-4.5398	3.8647	12.6322	3.79E-04	9.91E-03	0.0430
POLR1B	4.3396	1.2471	12.6170	3.82E-04	9.96E-03	20.2468
CRB1	3.3559	1.8708	12.6145	3.83E-04	9.96E-03	10.2380
LIMCH1	-4.0990	2.8548	12.6099	3.84E-04	9.97E-03	0.0584

ITPR2	2.6603	1.0503	12.5917	3.87E-04	1.00E-02	6.3216
TDRD12	5.2249	3.1337	12.5866	3.89E-04	1.00E-02	37.3987
MTND1P4	4.2712	1.5564	12.5706	3.92E-04	1.01E-02	19.3091
C19orf66	-3.0256	3.6354	12.5678	3.92E-04	1.01E-02	0.1228
LIN7B	3.1702	0.8593	12.5661	3.93E-04	1.01E-02	9.0017
DGKB	2.3726	0.8396	12.5232	4.02E-04	1.03E-02	5.1789
ZNF500	2.7896	0.7140	12.5185	4.03E-04	1.03E-02	6.9146
TRAF3IP2	-2.8479	3.7948	12.5096	4.05E-04	1.03E-02	0.1389
SLC12A5	5.6606	1.1733	12.5041	4.06E-04	1.03E-02	50.5823
ANGPT1	2.7563	0.6482	12.4927	4.09E-04	1.04E-02	6.7566
CPS1	3.9250	2.3817	12.4846	4.10E-04	1.04E-02	15.1900
PTPN7	3.2904	1.0221	12.4819	4.11E-04	1.04E-02	9.7841
ZNF350	2.7020	0.9769	12.4771	4.12E-04	1.04E-02	6.5071
FAM76B	3.9441	2.5891	12.4753	4.12E-04	1.04E-02	15.3917
HOXB13	3.1326	2.1837	12.4655	4.15E-04	1.04E-02	8.7703
TERF2IP	-2.8668	7.3165	12.4517	4.18E-04	1.05E-02	0.1371
RP1-79C4.4	4.2404	1.4925	12.4350	4.21E-04	1.05E-02	18.9005
FBXO42	2.9448	1.4531	12.4273	4.23E-04	1.06E-02	7.6999
APH1A	-3.1763	3.0540	12.4063	4.28E-04	1.06E-02	0.1106
ZNF577	2.4320	0.7292	12.4018	4.29E-04	1.06E-02	5.3965
STAT5A	4.0492	1.0799	12.3988	4.30E-04	1.06E-02	16.5550
NWD2	-3.2170	0.8084	12.3963	4.30E-04	1.06E-02	0.1075
HMGB3P19	4.6438	1.0106	12.3833	4.33E-04	1.07E-02	24.9987
POP7	-4.1651	1.1377	12.3816	4.34E-04	1.07E-02	0.0557
KPNA1	-2.7763	4.1577	12.3814	4.34E-04	1.07E-02	0.1460
CUTA	-2.7516	2.5565	12.3752	4.35E-04	1.07E-02	0.1485
SAMD5	3.8397	1.4152	12.3742	4.35E-04	1.07E-02	14.3174
AC069363.1	3.9322	1.0782	12.3630	4.38E-04	1.07E-02	15.2659
ZFC3H1	3.7237	2.5589	12.3392	4.44E-04	1.08E-02	13.2115
RP11-679C8.2	3.6519	1.2867	12.3309	4.46E-04	1.08E-02	12.5695
RP11-503C24.4	2.8226	0.9830	12.3223	4.48E-04	1.09E-02	7.0744
LINC00899	2.9227	0.9147	12.3148	4.49E-04	1.09E-02	7.5829
KBTBD11-OT1	-4.4882	1.3660	12.3093	4.51E-04	1.09E-02	0.0446
IARS	3.5051	0.7961	12.3041	4.52E-04	1.09E-02	11.3536
CTD-3088G3.8	4.5087	2.7502	12.3003	4.53E-04	1.09E-02	22.7645
ZNF609	2.7862	1.0622	12.2852	4.57E-04	1.10E-02	6.8982
FAM105A	-2.3659	5.3346	12.2740	4.59E-04	1.10E-02	0.1940
ADCY5	3.8544	0.8905	12.2676	4.61E-04	1.10E-02	14.4636
HOXA10	-3.3733	4.9143	12.2663	4.61E-04	1.10E-02	0.0965
TNC	5.6765	5.6851	12.2540	4.64E-04	1.11E-02	51.1439
LMTK2	-3.5740	3.9712	12.2383	4.68E-04	1.11E-02	0.0840

RP11-982M15.8	4.5725	2.2894	12.2362	4.69E-04	1.11E-02	23.7934
ACOX3	-2.9349	0.9243	12.2125	4.75E-04	1.12E-02	0.1308
CRHR2	2.3138	0.7151	12.2037	4.77E-04	1.13E-02	4.9719
DUSP19	3.8405	0.9916	12.1777	4.84E-04	1.14E-02	14.3252
SLC25A5-AS1	-3.7892	1.1255	12.1657	4.87E-04	1.14E-02	0.0723
MATR3	-2.1400	4.9514	12.1648	4.87E-04	1.14E-02	0.2269
RHBDF2	3.6110	1.0155	12.1632	4.87E-04	1.14E-02	12.2184
NHS	2.9298	0.9174	12.1566	4.89E-04	1.14E-02	7.6199
PDE1A	2.3358	0.6093	12.1461	4.92E-04	1.15E-02	5.0482
ASH1L	-2.8993	4.9912	12.1397	4.94E-04	1.15E-02	0.1340
ANGPTL4	3.7393	1.2388	12.1253	4.97E-04	1.16E-02	13.3545
DCTN1	2.5475	0.7782	12.1235	4.98E-04	1.16E-02	5.8462
RN7SL636P	2.6760	0.7979	12.1062	5.03E-04	1.16E-02	6.3909
C8orf82	2.8551	1.1576	12.0801	5.10E-04	1.18E-02	7.2353
RP11-429P3.4	5.8567	1.2623	12.0733	5.12E-04	1.18E-02	57.9484
U91328.19	3.8485	1.2563	12.0722	5.12E-04	1.18E-02	14.4046
NTAN1	2.5503	0.8179	12.0676	5.13E-04	1.18E-02	5.8574
GABRB2	4.6409	1.9707	12.0641	5.14E-04	1.18E-02	24.9491
GPATCH8	-3.2128	2.3667	12.0585	5.16E-04	1.18E-02	0.1079
FAM216B	4.1835	0.9482	12.0435	5.20E-04	1.19E-02	18.1704
RP11-212I21.4	2.5144	0.8324	12.0383	5.21E-04	1.19E-02	5.7135
MITF	2.3491	0.6486	12.0195	5.26E-04	1.20E-02	5.0951
AC016629.8	4.0492	1.2247	12.0168	5.27E-04	1.20E-02	16.5553
GINS4	2.6589	0.7735	12.0121	5.29E-04	1.20E-02	6.3155
RP11-443B7.1	2.3304	0.6863	12.0036	5.31E-04	1.20E-02	5.0294
RP11-169D4.1	2.4321	0.6550	11.9893	5.35E-04	1.21E-02	5.3969
PPP6R2	3.0769	2.9632	11.9891	5.35E-04	1.21E-02	8.4382
LHX9	3.0032	0.8708	11.9653	5.42E-04	1.22E-02	8.0177
TCF23	2.7696	1.0549	11.9416	5.49E-04	1.23E-02	6.8193
LINC00608	2.3604	0.7970	11.9172	5.56E-04	1.25E-02	5.1351
PRKAR1B	2.7065	0.7055	11.9055	5.60E-04	1.25E-02	6.5273
PPCDC	3.4443	1.3922	11.9001	5.61E-04	1.25E-02	10.8849
RLIM	2.2641	0.8461	11.8703	5.70E-04	1.27E-02	4.8036
NAGK	3.7129	1.2848	11.8658	5.72E-04	1.27E-02	13.1129
TMEM56*	-2.1653	6.6609	11.8529	5.76E-04	1.27E-02	0.2229
PPIG	-2.3774	5.8812	11.8525	5.76E-04	1.27E-02	0.1925
RP11-883A18.3	6.1330	2.5153	11.8418	5.79E-04	1.28E-02	70.1791
TTYH3	-2.7229	6.7538	11.8352	5.81E-04	1.28E-02	0.1515
RUVBL1	2.3835	0.6246	11.8240	5.85E-04	1.29E-02	5.2181
DNAJC22	-3.4127	2.9593	11.8235	5.85E-04	1.29E-02	0.0939
CTC-277H1.7	2.6768	0.7511	11.8113	5.89E-04	1.29E-02	6.3945

FTX	2.3679	0.7607	11.8082	5.90E-04	1.29E-02	5.1619
CTB-33O18.3	3.3113	1.0157	11.8047	5.91E-04	1.29E-02	9.9266
ZNF319	2.3243	0.6846	11.7996	5.92E-04	1.29E-02	5.0082
RP4-777D9.2	4.3660	1.1556	11.7912	5.95E-04	1.29E-02	20.6210
RPPH1	3.2376	2.1546	11.7878	5.96E-04	1.29E-02	9.4321
SP6	-4.9522	1.6525	11.7851	5.97E-04	1.29E-02	0.0323
RUNX3	5.3744	4.4411	11.7736	6.01E-04	1.30E-02	41.4829
SEC24B	-3.2817	3.6501	11.7705	6.02E-04	1.30E-02	0.1028
PCDH7	3.6104	1.3355	11.7687	6.02E-04	1.30E-02	12.2132
SLC15A2	3.6186	0.9456	11.7629	6.04E-04	1.30E-02	12.2829
PTPRC*	2.7596	5.5875	11.7589	6.06E-04	1.30E-02	6.7720
TNFRSF9	3.0866	0.7435	11.7552	6.07E-04	1.30E-02	8.4948
DDX52	-2.9585	2.8313	11.7451	6.10E-04	1.30E-02	0.1286
GK3P	3.5799	1.4378	11.7429	6.11E-04	1.30E-02	11.9576
MUC16	3.9595	1.6312	11.7331	6.14E-04	1.31E-02	15.5567
CDH7	3.3596	1.2853	11.7327	6.14E-04	1.31E-02	10.2643
NSMAF	3.2957	1.3946	11.7318	6.14E-04	1.31E-02	9.8198
GFRA1	4.5912	1.7466	11.7127	6.21E-04	1.32E-02	24.1048
DDX10	2.5046	0.7764	11.7065	6.23E-04	1.32E-02	5.6749
BNC2	4.1440	1.9349	11.7063	6.23E-04	1.32E-02	17.6797
RP11-582J16.4	3.3372	1.2219	11.7009	6.25E-04	1.32E-02	10.1062
AC013474.4	3.1557	0.6482	11.6868	6.29E-04	1.33E-02	8.9116
AL157830.1	3.6542	0.9717	11.6810	6.31E-04	1.33E-02	12.5900
IL3RA	4.2282	2.3960	11.6792	6.32E-04	1.33E-02	18.7418
GRIA2	2.8865	0.8615	11.6682	6.36E-04	1.33E-02	7.3945
STARD7-AS1	4.2319	0.9873	11.6678	6.36E-04	1.33E-02	18.7904
USP21	-2.8656	0.7003	11.6643	6.37E-04	1.33E-02	0.1372
UNC13A	3.5295	1.2119	11.6593	6.39E-04	1.33E-02	11.5472
DRAXIN	2.8258	0.8533	11.6422	6.45E-04	1.34E-02	7.0899
QTRTD1	3.3756	0.8808	11.6366	6.47E-04	1.34E-02	10.3789
KIAA1841*	3.7687	2.3484	11.6193	6.53E-04	1.34E-02	13.6302
C12orf29	4.2955	1.2797	11.6248	6.51E-04	1.34E-02	19.6367
PLA2G4F	-4.3029	1.0222	11.6194	6.53E-04	1.34E-02	0.0507
GNG7	3.0150	1.3037	11.6185	6.53E-04	1.34E-02	8.0835
NFATC2IP	2.4957	2.4415	11.6118	6.55E-04	1.34E-02	5.6401
MAP1S	2.2518	0.6976	11.6103	6.56E-04	1.34E-02	4.7627
BICD1	3.0557	0.6712	11.6101	6.56E-04	1.34E-02	8.3149
GAL3ST1	2.4327	0.6102	11.6015	6.59E-04	1.35E-02	5.3990
NIPBL	-3.5109	2.6029	11.5922	6.62E-04	1.35E-02	0.0877
LINC01397	3.9606	1.1804	11.5901	6.63E-04	1.35E-02	15.5689
LDB2	3.0223	0.7481	11.5866	6.64E-04	1.35E-02	8.1247

KRT16	3.7243	3.8152	11.5735	6.69E-04	1.36E-02	13.2171
CLDN3	-2.1421	4.0928	11.5557	6.75E-04	1.37E-02	0.2265
CDH8	3.8257	2.2997	11.5535	6.76E-04	1.37E-02	14.1796
KRT12	-3.3570	0.6925	11.5465	6.79E-04	1.37E-02	0.0976
DENND4B	-3.6898	1.5495	11.5421	6.80E-04	1.37E-02	0.0775
SMCR5	4.3010	0.9929	11.5405	6.81E-04	1.37E-02	19.7122
MEF2BNB	4.3620	2.4174	11.5390	6.82E-04	1.37E-02	20.5633
ERN1	3.2143	2.5311	11.5240	6.87E-04	1.38E-02	9.2811
PCYT1B	2.9658	0.8317	11.4920	6.99E-04	1.40E-02	7.8124
RP11-575F12.3	3.2972	0.7502	11.4834	7.02E-04	1.40E-02	9.8299
CCDC34	3.3132	0.9865	11.4719	7.07E-04	1.41E-02	9.9395
SEPT4	4.6508	1.5930	11.4693	7.08E-04	1.41E-02	25.1210
RNU2-8P	3.6412	1.4505	11.4661	7.09E-04	1.41E-02	12.4771
LURAP1	4.0445	0.9105	11.4366	7.20E-04	1.43E-02	16.5018
THADA	2.5099	0.9114	11.4354	7.21E-04	1.43E-02	5.6956
DTWD1	3.8895	1.9900	11.4300	7.23E-04	1.43E-02	14.8207
RFT1	4.4158	1.2803	11.3987	7.35E-04	1.45E-02	21.3447
OMA1	3.3974	1.2053	11.3980	7.35E-04	1.45E-02	10.5373
EVA1A	2.9975	0.8331	11.3968	7.36E-04	1.45E-02	7.9860
WDR61	2.9645	1.1367	11.3925	7.37E-04	1.45E-02	7.8057
SLC8A3	2.6546	0.7233	11.3888	7.39E-04	1.45E-02	6.2966
EBLN2	3.1322	0.7353	11.3865	7.40E-04	1.45E-02	8.7674
ZBTB48	-4.0396	2.1553	11.3818	7.42E-04	1.45E-02	0.0608
RP11-296C13.1	3.8488	2.5166	11.3768	7.44E-04	1.45E-02	14.4076
FCGR3A	-3.6675	1.0153	11.3660	7.48E-04	1.46E-02	0.0787
DRAM1	3.1997	2.2130	11.3626	7.49E-04	1.46E-02	9.1877
PLA2G10	-2.2027	5.2349	11.3480	7.55E-04	1.47E-02	0.2172
ALPK2	3.6441	1.0068	11.3399	7.59E-04	1.47E-02	12.5019
RORB	2.1853	0.6300	11.3377	7.59E-04	1.47E-02	4.5481
ZNF532	5.3392	2.1222	11.3362	7.60E-04	1.47E-02	40.4819
ITK	3.7990	1.1818	11.3324	7.62E-04	1.47E-02	13.9188
IGF2BP1	3.8970	1.7486	11.3217	7.66E-04	1.48E-02	14.8973
SHMT2	-4.3671	1.3519	11.3141	7.69E-04	1.48E-02	0.0485
RP11-489E7.1	3.8132	1.2944	11.2937	7.78E-04	1.49E-02	14.0565
PPIE	3.0863	1.7886	11.2906	7.79E-04	1.49E-02	8.4929
RP11-531A24.5	3.2890	1.0066	11.2823	7.83E-04	1.49E-02	9.7744
CACNA1E	3.6921	2.1039	11.2806	7.83E-04	1.49E-02	12.9252
ST8SIA1	2.6779	0.9039	11.2798	7.84E-04	1.49E-02	6.3991
RP1-228P16.4	3.4527	0.7536	11.2778	7.84E-04	1.49E-02	10.9491
LY75	3.8621	2.8281	11.2752	7.86E-04	1.49E-02	14.5419
NPM1	-2.5381	4.9533	11.2748	7.86E-04	1.49E-02	0.1722

P2RY14	5.4128	1.6135	11.2332	8.03E-04	1.52E-02	42.6009
SULT1A2	-2.4070	3.1840	11.2182	8.10E-04	1.53E-02	0.1885
PEG3	3.3877	0.8540	11.2139	8.12E-04	1.53E-02	10.4661
RP1-102H19.8	3.3905	1.1421	11.2119	8.13E-04	1.53E-02	10.4869
CSF1	3.1710	1.0560	11.2100	8.14E-04	1.53E-02	9.0068
SMG7-AS1	3.7385	1.0516	11.2014	8.17E-04	1.53E-02	13.3472
MIB2	2.5007	1.0975	11.1958	8.20E-04	1.53E-02	5.6595
WDR7	2.8396	0.7823	11.1952	8.20E-04	1.53E-02	7.1584
CAPN10	3.6868	1.1391	11.1932	8.21E-04	1.53E-02	12.8781
AADAC	-2.5713	5.5659	11.1931	8.21E-04	1.53E-02	0.1683
C12orf66	2.7906	0.9816	11.1787	8.27E-04	1.54E-02	6.9191
CRIM1	-1.9755	5.4781	11.1784	8.28E-04	1.54E-02	0.2543
RP11-321E2.13	8.2761	7.1961	11.1782	8.28E-04	1.54E-02	309.9852
SRCIN1	3.8418	1.3970	11.1734	8.30E-04	1.54E-02	14.3387
SLC30A9	3.5679	4.6432	11.1719	8.30E-04	1.54E-02	11.8593
HIST1H4E	7.1562	2.5209	11.1671	8.33E-04	1.54E-02	142.6366
FAM49A	3.5660	2.1831	11.1534	8.39E-04	1.55E-02	11.8437
IQUB	3.3863	0.8357	11.1503	8.40E-04	1.55E-02	10.4565
FAM57B	2.3185	0.8426	11.1502	8.40E-04	1.55E-02	4.9880
XBP1	-2.6177	3.2574	11.1466	8.42E-04	1.55E-02	0.1629
SMPD4P1	2.8629	0.6528	11.1451	8.43E-04	1.55E-02	7.2745
FUT2	-2.9232	3.7845	11.1312	8.49E-04	1.55E-02	0.1318
FAM83H-AS1	3.0611	0.9012	11.1297	8.50E-04	1.55E-02	8.3461
ZNF682	3.0704	0.8977	11.1240	8.52E-04	1.55E-02	8.4002
RGS7	4.6616	2.3376	11.1219	8.53E-04	1.55E-02	25.3099
C19orf25	2.6879	1.2795	11.1218	8.53E-04	1.55E-02	6.4436
PPP2R2B	2.1707	0.5891	11.1165	8.56E-04	1.55E-02	4.5025
AUH	-2.7602	1.8621	11.1135	8.57E-04	1.55E-02	0.1476
APOPT1	3.8628	1.8738	11.1105	8.58E-04	1.55E-02	14.5482
LMO7DN	2.9798	1.3436	11.0996	8.63E-04	1.56E-02	7.8886
YIPF6	-2.6139	6.3881	11.0992	8.64E-04	1.56E-02	0.1634
TNPO2	-2.0277	5.1474	11.0908	8.68E-04	1.56E-02	0.2452
RP11-281N10.1	2.9120	1.0364	11.0868	8.69E-04	1.57E-02	7.5266
STEAP4	2.7726	2.7797	11.0781	8.74E-04	1.57E-02	6.8336
SEC22C	3.7558	1.5846	11.0762	8.74E-04	1.57E-02	13.5090
MRPL46	3.9448	1.0669	11.0639	8.80E-04	1.57E-02	15.3992
TLL2	3.6663	0.8385	11.0630	8.81E-04	1.57E-02	12.6956
RTKN2	2.6679	1.1054	11.0616	8.81E-04	1.57E-02	6.3550
AC007386.2	3.3068	1.2810	11.0607	8.82E-04	1.57E-02	9.8955
CCDC180	2.2255	0.5885	11.0450	8.89E-04	1.58E-02	4.6766
ZNF618	3.4187	1.4384	11.0380	8.93E-04	1.59E-02	10.6936

STARD7	-2.6780	5.5533	11.0364	8.93E-04	1.59E-02	0.1563
CDH24	2.2204	0.5860	11.0316	8.96E-04	1.59E-02	4.6601
GPR155	4.0019	1.3865	11.0263	8.98E-04	1.59E-02	16.0211
DNAL1	3.7289	3.8515	11.0250	8.99E-04	1.59E-02	13.2586
RP11-84A19.2	4.5571	3.0823	11.0201	9.01E-04	1.59E-02	23.5404
SCPEP1	4.0406	1.3843	11.0190	9.02E-04	1.59E-02	16.4571
CDH4	3.2881	3.1573	11.0173	9.03E-04	1.59E-02	9.7685
GPANK1	3.5111	1.6957	11.0084	9.07E-04	1.59E-02	11.4013
ABCA13	4.2453	1.4061	11.0034	9.09E-04	1.59E-02	18.9655
KLF16	-3.2769	1.0579	11.0021	9.10E-04	1.59E-02	0.1032
CTD-2319I12.9	2.3569	0.7986	10.9874	9.17E-04	1.60E-02	5.1227
RP11-725D20.1	2.5091	0.8575	10.9810	9.21E-04	1.61E-02	5.6926
TNFRSF12A	-3.6718	2.9384	10.9798	9.21E-04	1.61E-02	0.0785
CD160	2.7049	0.6945	10.9554	9.33E-04	1.61E-02	6.5202
FAM47E	-3.4083	0.8970	10.9543	9.34E-04	1.61E-02	0.0942
RP11-282K24.3	2.7559	1.1385	10.9531	9.34E-04	1.61E-02	6.7547
NLRP8	2.9578	1.0152	10.9520	9.35E-04	1.61E-02	7.7695
TTC31	-4.4628	1.1881	10.9501	9.36E-04	1.61E-02	0.0453
DNPH1	-3.5507	1.0777	10.9495	9.36E-04	1.61E-02	0.0853
SLC16A12	2.8378	0.7619	10.9469	9.38E-04	1.61E-02	7.1491
ZNF331	4.7478	2.7981	10.9466	9.38E-04	1.61E-02	26.8678
DNAH12	2.2847	0.6929	10.9439	9.39E-04	1.61E-02	4.8725
CHST9	4.1454	1.9461	10.9418	9.40E-04	1.61E-02	17.6963
FBXO27	3.7144	1.0609	10.9414	9.40E-04	1.61E-02	13.1261
SUPT20H	3.8903	1.6395	10.9414	9.40E-04	1.61E-02	14.8280
STXBP6	-3.0587	4.8187	10.9412	9.40E-04	1.61E-02	0.1200
RP11-295P9.8	5.3275	2.5972	10.9225	9.50E-04	1.62E-02	40.1551
LSM14B	-3.2694	3.4932	10.9189	9.52E-04	1.62E-02	0.1037
ZNF143	-3.3022	1.5226	10.9180	9.52E-04	1.62E-02	0.1014
NFRKB	3.5683	1.7086	10.9168	9.53E-04	1.62E-02	11.8620
SPATA18	3.4307	0.9340	10.9133	9.55E-04	1.62E-02	10.7833
TSC22D3	4.2050	6.3895	10.9078	9.58E-04	1.62E-02	18.4425
KIAA0586	2.9549	4.1320	10.9014	9.61E-04	1.63E-02	7.7536
APOL1	2.8295	2.9805	10.8860	9.69E-04	1.64E-02	7.1084
SLC38A6	2.3855	0.6273	10.8821	9.71E-04	1.64E-02	5.2253
KLF17	2.5282	0.7797	10.8676	9.79E-04	1.65E-02	5.7685
SERPINB3	3.2380	1.0968	10.8589	9.83E-04	1.65E-02	9.4346
ITGA2	3.2126	3.4311	10.8488	9.89E-04	1.66E-02	9.2704
USP30	2.9949	0.9390	10.8483	9.89E-04	1.66E-02	7.9717
RP11-383C5.3	3.6716	0.8482	10.8450	9.91E-04	1.66E-02	12.7430
IFT22	3.3403	0.9733	10.8427	9.92E-04	1.66E-02	10.1281

LINC00661	3.6589	1.0329	10.8415	9.93E-04	1.66E-02	12.6308
MICB	3.9845	1.6232	10.8398	9.93E-04	1.66E-02	15.8289
ASXL3	3.7382	1.3336	10.8287	9.99E-04	1.66E-02	13.3451
RP11-348N5.7	-3.0201	0.7022	10.8241	1.00E-03	1.67E-02	0.1233
TIA1	3.2550	2.3254	10.8207	1.00E-03	1.67E-02	9.5468
RP11-100M12.2	3.7631	0.9104	10.8130	1.01E-03	1.67E-02	13.5774
CXXC4	3.4263	1.4656	10.8045	1.01E-03	1.68E-02	10.7503
GPR133	2.9070	0.7440	10.8034	1.01E-03	1.68E-02	7.5008
DNAH2	2.8506	1.0288	10.7988	1.02E-03	1.68E-02	7.2128
DNAJC27*	4.0690	1.3558	10.7707	1.03E-03	1.70E-02	16.7834
PKHD1L1	2.8877	0.7502	10.7731	1.03E-03	1.70E-02	7.4011
ADRA2A	2.4591	0.8503	10.7618	1.04E-03	1.70E-02	5.4988
MAP1B	3.7953	1.1801	10.7583	1.04E-03	1.70E-02	13.8839
RP11-524C21.2	4.3318	1.7532	10.7580	1.04E-03	1.70E-02	20.1369
CDCP1	2.2953	4.2638	10.7518	1.04E-03	1.70E-02	4.9085
KSR2	3.2425	1.5440	10.7495	1.04E-03	1.70E-02	9.4645
YTHDC2	4.3654	3.4130	10.7494	1.04E-03	1.70E-02	20.6115
ZMYM4	2.4974	3.2293	10.7492	1.04E-03	1.70E-02	5.6468
MIR3663HG	3.5336	0.8534	10.7479	1.04E-03	1.70E-02	11.5807
TRAM1	-2.1040	5.6799	10.7446	1.05E-03	1.70E-02	0.2326
FER	4.5648	2.6149	10.7445	1.05E-03	1.70E-02	23.6668
SETBP1	4.1663	1.9555	10.7410	1.05E-03	1.70E-02	17.9545
USP17L7	3.7581	1.3313	10.7368	1.05E-03	1.70E-02	13.5297
RP11-327F22.1	3.6116	1.5805	10.7333	1.05E-03	1.70E-02	12.2236
ANKRD55	2.9807	1.7670	10.7299	1.05E-03	1.70E-02	7.8939
CTB-89H12.4	-4.0359	1.2919	10.7292	1.05E-03	1.70E-02	0.0610
CEP19	3.4690	1.4176	10.7212	1.06E-03	1.70E-02	11.0730
DNAJB5P1	2.7566	1.1850	10.7195	1.06E-03	1.70E-02	6.7580
CCDC83	2.9175	1.3929	10.7063	1.07E-03	1.71E-02	7.5555
TBC1D8B	2.7572	0.9482	10.7039	1.07E-03	1.71E-02	6.7606
COL16A1	2.9707	1.4131	10.6924	1.08E-03	1.72E-02	7.8392
GRK5	3.3009	0.9582	10.6902	1.08E-03	1.72E-02	9.8554
ERCC6	2.9247	1.0619	10.6807	1.08E-03	1.73E-02	7.5930
LUC7L3	-2.3607	4.7329	10.6685	1.09E-03	1.74E-02	0.1947
HS6ST3	2.6590	0.6846	10.6675	1.09E-03	1.74E-02	6.3158
PDPR	3.2761	1.5626	10.6647	1.09E-03	1.74E-02	9.6871
UNC5CL	-4.1026	4.5571	10.6626	1.09E-03	1.74E-02	0.0582
ARHGAP20	2.8614	0.7525	10.6606	1.09E-03	1.74E-02	7.2674
HOXA10-AS	3.3779	0.8855	10.6491	1.10E-03	1.74E-02	10.3953
CSF2RB	2.8484	3.1031	10.6288	1.11E-03	1.76E-02	7.2021
KIAA1324	-2.8755	3.0435	10.6229	1.12E-03	1.76E-02	0.1363

LAMA3	-2.0832	4.0789	10.6215	1.12E-03	1.76E-02	0.2360
ZCCHC17	-4.0126	1.8582	10.5912	1.14E-03	1.79E-02	0.0620
TMEM60	-3.1758	1.8129	10.5882	1.14E-03	1.79E-02	0.1107
MDM1	3.6367	1.0429	10.5822	1.14E-03	1.79E-02	12.4383
IL1R1	5.1771	3.0658	10.5806	1.14E-03	1.79E-02	36.1787
KDM1B	-2.5604	4.0721	10.5797	1.14E-03	1.79E-02	0.1695
CLCA2	2.2971	0.5845	10.5747	1.15E-03	1.79E-02	4.9147
FOPNL	3.3552	1.8203	10.5733	1.15E-03	1.79E-02	10.2330
RNU6-47P	2.0512	0.6072	10.5616	1.15E-03	1.80E-02	4.1444
SIAH3*	2.8892	0.9506	10.5461	1.16E-03	1.81E-02	7.4088
TRHDE	3.6056	2.8987	10.5461	1.16E-03	1.81E-02	12.1728
C14orf1	-2.9883	3.2433	10.5397	1.17E-03	1.81E-02	0.1260
SNORA7	2.5110	2.9595	10.5397	1.17E-03	1.81E-02	5.7002
RP11-259A24.1	3.3133	1.5661	10.5388	1.17E-03	1.81E-02	9.9401
TRAPPC10	3.5252	1.8278	10.5346	1.17E-03	1.81E-02	11.5127
MCF2L2	4.2538	3.8450	10.5292	1.18E-03	1.82E-02	19.0773
LINC01249	3.7864	1.2560	10.5227	1.18E-03	1.82E-02	13.7980
ANKRD18EP	3.3788	0.7228	10.5138	1.18E-03	1.82E-02	10.4018
RAB34	4.4463	3.2016	10.5136	1.19E-03	1.82E-02	21.7999
JADE3	3.4075	1.3937	10.4983	1.19E-03	1.83E-02	10.6108
KIF13B	3.7845	2.0437	10.4980	1.20E-03	1.83E-02	13.7798
IL17RE	2.7089	0.7978	10.4933	1.20E-03	1.83E-02	6.5384
SYT11	2.1365	0.5906	10.4931	1.20E-03	1.83E-02	4.3969
G6PC3	4.2251	1.1871	10.4924	1.20E-03	1.83E-02	18.7013
KIAA1407	2.2822	0.6970	10.4923	1.20E-03	1.83E-02	4.8641
STARD10	2.7753	3.4690	10.4881	1.20E-03	1.83E-02	6.8462
RP11-624C23.1	2.5986	0.6733	10.4877	1.20E-03	1.83E-02	6.0568
RP11-126O22.1	6.3546	5.3399	10.4734	1.21E-03	1.84E-02	81.8314
GLT8D2	3.2412	0.8091	10.4724	1.21E-03	1.84E-02	9.4558
PAK3	3.2704	2.0575	10.4684	1.21E-03	1.84E-02	9.6489
ZNF585B	-2.8544	3.3048	10.4682	1.21E-03	1.84E-02	0.1383
RP11-354E11.2	3.2541	0.9942	10.4644	1.22E-03	1.84E-02	9.5407
GDPD3	-3.0180	3.0010	10.4603	1.22E-03	1.84E-02	0.1235
SRRM3	3.3118	1.2864	10.4582	1.22E-03	1.84E-02	9.9302
KCNN3	2.1413	0.6931	10.4546	1.22E-03	1.84E-02	4.4116
REL	3.1550	1.6275	10.4529	1.22E-03	1.84E-02	8.9075
ACAP1	2.6259	0.9137	10.4501	1.23E-03	1.84E-02	6.1727
MCM8	2.0858	0.5805	10.4458	1.23E-03	1.84E-02	4.2452
NRBP1	3.9228	1.6968	10.4448	1.23E-03	1.84E-02	15.1667
LANCL1	2.4090	0.8286	10.4278	1.24E-03	1.86E-02	5.3112
ATP2C1	2.6553	5.6323	10.4254	1.24E-03	1.86E-02	6.2997

RP11-379C10.1	3.9685	0.8481	10.4227	1.24E-03	1.86E-02	15.6547
SCUBE1	2.6990	2.9677	10.4213	1.25E-03	1.86E-02	6.4935
NEURL3	-3.5325	1.2356	10.4188	1.25E-03	1.86E-02	0.0864
RP5-973N23.4	2.7260	0.8919	10.4166	1.25E-03	1.86E-02	6.6162
AC002472.13	3.5857	1.4664	10.4125	1.25E-03	1.86E-02	12.0064
EXOC6B	-2.5131	4.3657	10.3986	1.26E-03	1.87E-02	0.1752
G0S2	2.4872	12.2245	10.3931	1.26E-03	1.87E-02	5.6068
DAPP1	3.3461	2.0479	10.3818	1.27E-03	1.88E-02	10.1690
ALPK1	3.6475	3.8554	10.3800	1.27E-03	1.88E-02	12.5317
ATP8B4	4.0897	1.2221	10.3726	1.28E-03	1.89E-02	17.0262
ZNF419	2.6611	0.8667	10.3477	1.30E-03	1.91E-02	6.3254
TOM1	-2.7188	2.4197	10.3441	1.30E-03	1.91E-02	0.1519
PKD1L2	2.9756	1.7427	10.3314	1.31E-03	1.92E-02	7.8660
CPEB1	2.7881	5.8307	10.3207	1.32E-03	1.93E-02	6.9070
CDK2	-2.9380	4.1222	10.3202	1.32E-03	1.93E-02	0.1305
ZNF583	2.0716	0.5637	10.3051	1.33E-03	1.94E-02	4.2036
ICAM1	3.3784	1.1668	10.3003	1.33E-03	1.94E-02	10.3991
WNT3	2.9210	0.9250	10.2990	1.33E-03	1.94E-02	7.5738
KIF6	3.5074	0.9991	10.2945	1.33E-03	1.95E-02	11.3721
ABHD10	-3.8113	1.6501	10.2806	1.34E-03	1.96E-02	0.0712
MED12	-3.4612	1.6272	10.2778	1.35E-03	1.96E-02	0.0908
VNN1	-2.7797	2.9481	10.2717	1.35E-03	1.96E-02	0.1456
RP11-529E10.7	3.3427	3.2622	10.2655	1.36E-03	1.97E-02	10.1452
LRRTM4	3.8366	0.9219	10.2651	1.36E-03	1.97E-02	14.2868
MTFMT	-4.2879	3.7937	10.2486	1.37E-03	1.98E-02	0.0512
RP11-1060J15.5	-2.7019	0.6381	10.2476	1.37E-03	1.98E-02	0.1537
CHMP2A	-2.2902	2.9442	10.2449	1.37E-03	1.98E-02	0.2044
CSF2RA	3.9919	2.0606	10.2424	1.37E-03	1.98E-02	15.9104
CTD-2015H6.2	4.6033	0.9006	10.2394	1.37E-03	1.98E-02	24.3075
C12orf55	3.4415	1.1286	10.2380	1.38E-03	1.98E-02	10.8641
RP11-304C12.4	2.1572	0.6454	10.2359	1.38E-03	1.98E-02	4.4605
RP3-468B3.2	3.3635	1.2346	10.2299	1.38E-03	1.98E-02	10.2925
CHRDL1	3.8012	1.2710	10.2287	1.38E-03	1.98E-02	13.9407
MLLT3	1.9221	9.1818	10.2164	1.39E-03	1.99E-02	3.7898
GALNT11	2.1786	0.8076	10.2153	1.39E-03	1.99E-02	4.5272
ZDHHC21	-2.6831	4.6131	10.2000	1.40E-03	2.01E-02	0.1557
CTD-2246P4.1	2.5643	0.8289	10.1897	1.41E-03	2.01E-02	5.9149
MEMO1	3.4485	2.8858	10.1862	1.41E-03	2.02E-02	10.9169
HDAC6	3.3320	1.0830	10.1803	1.42E-03	2.02E-02	10.0701
ATM	3.9103	1.5461	10.1756	1.42E-03	2.02E-02	15.0356
SPATA6	3.0989	1.0807	10.1724	1.43E-03	2.02E-02	8.5677

USP36	3.1442	1.1827	10.1683	1.43E-03	2.03E-02	8.8407
AP2A2	2.7901	0.8526	10.1630	1.43E-03	2.03E-02	6.9169
ATRNL1	2.6772	0.7746	10.1610	1.43E-03	2.03E-02	6.3962
BPHL	3.2395	1.1091	10.1605	1.43E-03	2.03E-02	9.4447
RP11-638I2.2	4.0375	1.0428	10.1575	1.44E-03	2.03E-02	16.4213
RP11-355F16.1	3.0573	1.0991	10.1543	1.44E-03	2.03E-02	8.3243
AP5B1	-3.2999	4.8663	10.1481	1.44E-03	2.03E-02	0.1015
AC114763.1	4.3997	1.4979	10.1288	1.46E-03	2.05E-02	21.1074
CAST	2.0482	8.9736	10.1155	1.47E-03	2.06E-02	4.1358
NR2F2-AS1	3.0182	1.3096	10.1149	1.47E-03	2.06E-02	8.1014
MORC3	3.1848	5.8636	10.1136	1.47E-03	2.06E-02	9.0931
MIR490	3.2274	1.1281	10.1049	1.48E-03	2.07E-02	9.3657
RP11-122M14.1	2.7168	1.0280	10.1003	1.48E-03	2.07E-02	6.5740
EIF4H	-2.5122	1.2888	10.0970	1.49E-03	2.07E-02	0.1753
CAMKK2	2.2959	2.3527	10.0888	1.49E-03	2.08E-02	4.9105
RPGR*	4.1902	3.0035	10.0726	1.50E-03	2.09E-02	18.2550
THYN1	4.1809	2.9713	10.0788	1.50E-03	2.09E-02	18.1374
TRAPPC3L	3.6151	0.9601	10.0712	1.51E-03	2.09E-02	12.2531
ZBTB8B	2.6727	1.3151	10.0687	1.51E-03	2.09E-02	6.3761
RP11-526H11.1	4.3387	0.8592	10.0684	1.51E-03	2.09E-02	20.2343
JAM2	2.0627	0.5681	10.0656	1.51E-03	2.09E-02	4.1777
RP11-422J15.1	3.1108	1.6338	10.0651	1.51E-03	2.09E-02	8.6383
C6orf132	-2.3464	6.2608	10.0647	1.51E-03	2.09E-02	0.1966
GPR144	2.8874	0.9732	10.0640	1.51E-03	2.09E-02	7.3995
FPGT	-3.7752	2.7020	10.0551	1.52E-03	2.09E-02	0.0730
SNX20	3.2978	1.0269	10.0494	1.52E-03	2.10E-02	9.8340
RNU6-1064P	2.2258	5.9932	10.0477	1.53E-03	2.10E-02	4.6777
ABI3BP	2.6968	0.8072	10.0435	1.53E-03	2.10E-02	6.4838
RCSD1	2.8697	0.9520	10.0332	1.54E-03	2.11E-02	7.3090
GABRB3	2.1663	0.5878	10.0326	1.54E-03	2.11E-02	4.4888
RECK	3.2312	0.7183	10.0267	1.54E-03	2.11E-02	9.3903
CTBP2	2.0224	4.0287	10.0229	1.55E-03	2.11E-02	4.0625
RP11-540A21.2	3.6839	1.3647	10.0205	1.55E-03	2.11E-02	12.8522
VAX1	3.7981	2.1645	10.0158	1.55E-03	2.12E-02	13.9101
TAGAP	2.2874	4.0853	10.0131	1.55E-03	2.12E-02	4.8819
RP11-430B1.1	3.6050	1.3800	10.0108	1.56E-03	2.12E-02	12.1681
CAMKK1	3.7238	1.1445	10.0016	1.56E-03	2.12E-02	13.2126
SLC8A1-AS1	5.2946	3.6336	9.9812	1.58E-03	2.14E-02	39.2507
FBN1	4.1152	1.6226	9.9804	1.58E-03	2.14E-02	17.3296
FBXL17	2.6265	5.8607	9.9780	1.58E-03	2.14E-02	6.1752
QRSL1	2.8619	0.7346	9.9756	1.59E-03	2.14E-02	7.2698

KRR1	3.2554	1.2295	9.9751	1.59E-03	2.14E-02	9.5495
KIAA0101*	2.3658	0.8733	9.9564	1.60E-03	2.15E-02	5.1545
LRAT	4.8487	4.9796	9.9636	1.60E-03	2.15E-02	28.8143
PTAFR	2.7742	0.8977	9.9615	1.60E-03	2.15E-02	6.8408
SPRR3	2.1859	8.9155	9.9589	1.60E-03	2.15E-02	4.5502
SOCS3	3.3705	7.6501	9.9577	1.60E-03	2.15E-02	10.3425
ATP8A2	2.6460	1.2133	9.9558	1.60E-03	2.15E-02	6.2592
ATP2A2	1.9825	5.5393	9.9506	1.61E-03	2.15E-02	3.9518
BAHD1	-2.6272	1.6517	9.9417	1.62E-03	2.16E-02	0.1619
CTB-193M12.3	4.0468	1.6392	9.9416	1.62E-03	2.16E-02	16.5276
P2RY13	3.3191	1.6958	9.9378	1.62E-03	2.16E-02	9.9804
RGS17	2.2780	0.6472	9.9360	1.62E-03	2.16E-02	4.8499
SLC7A11-AS1	2.8941	0.8915	9.9339	1.62E-03	2.16E-02	7.4339
AC007952.5	4.4196	0.9171	9.9296	1.63E-03	2.16E-02	21.4016
SEMA4D	3.7939	4.5854	9.9276	1.63E-03	2.16E-02	13.8702
TMEM184A	2.6427	0.8081	9.9267	1.63E-03	2.16E-02	6.2452
MIR940	3.0424	1.2100	9.9038	1.65E-03	2.19E-02	8.2386
BTN2A2	2.3836	0.6708	9.9025	1.65E-03	2.19E-02	5.2184
ARMC4	2.6632	0.8687	9.8984	1.65E-03	2.19E-02	6.3342
TNS1	4.2260	1.4830	9.8933	1.66E-03	2.19E-02	18.7140
CCP110	2.9008	1.5987	9.8924	1.66E-03	2.19E-02	7.4684
ZNF674-AS1	3.3347	1.4753	9.8859	1.67E-03	2.19E-02	10.0891
FRMD4A	4.1497	1.8684	9.8806	1.67E-03	2.20E-02	17.7499
MAST3	3.4946	0.9621	9.8793	1.67E-03	2.20E-02	11.2714
ITFG3	-4.1341	2.1564	9.8752	1.68E-03	2.20E-02	0.0570
RP11-401E5.2	3.5445	1.2297	9.8731	1.68E-03	2.20E-02	11.6677
CTD-2650P22.2	2.1007	0.8907	9.8706	1.68E-03	2.20E-02	4.2891
REPS2	3.6405	2.8498	9.8665	1.68E-03	2.20E-02	12.4709
GRIK2	3.1078	0.9305	9.8609	1.69E-03	2.21E-02	8.6206
KPNA3	-2.9125	1.8741	9.8575	1.69E-03	2.21E-02	0.1328
KCNJ14	3.1770	1.0006	9.8563	1.69E-03	2.21E-02	9.0444
CTD-3010D24.3	-3.3273	1.0696	9.8527	1.70E-03	2.21E-02	0.0996
PFKM	6.0940	2.1987	9.8520	1.70E-03	2.21E-02	68.3083
CDC42BPA	-2.6724	4.0956	9.8377	1.71E-03	2.22E-02	0.1569
SNTG1	2.0832	0.6789	9.8269	1.72E-03	2.23E-02	4.2376
TTN-AS1	3.4804	0.8513	9.8254	1.72E-03	2.23E-02	11.1612
KIAA0825	2.9892	2.5193	9.8206	1.73E-03	2.23E-02	7.9403
STX5	-2.2144	2.2357	9.8203	1.73E-03	2.23E-02	0.2155
KIAA0125	2.5129	1.1152	9.8149	1.73E-03	2.24E-02	5.7075
GJC1	3.0555	0.9762	9.8092	1.74E-03	2.24E-02	8.3138
MC5R	3.8171	0.9153	9.8089	1.74E-03	2.24E-02	14.0945

TLR2	3.3677	1.7673	9.8062	1.74E-03	2.24E-02	10.3220
GPR180	3.0129	1.0587	9.7949	1.75E-03	2.25E-02	8.0718
ATXN7	-2.2783	4.8090	9.7832	1.76E-03	2.26E-02	0.2061
AKR1B1	-4.1661	1.4688	9.7788	1.77E-03	2.27E-02	0.0557
RECQL	2.0535	0.5945	9.7762	1.77E-03	2.27E-02	4.1511
CTNNA1	1.9868	7.2017	9.7749	1.77E-03	2.27E-02	3.9635
RP1-65J11.5	2.2467	0.8981	9.7712	1.77E-03	2.27E-02	4.7461
SMC5	-3.1027	3.2141	9.7696	1.77E-03	2.27E-02	0.1164
CXCL1	3.2150	4.2572	9.7622	1.78E-03	2.27E-02	9.2854
ARSD	3.1841	1.0735	9.7620	1.78E-03	2.27E-02	9.0887
PNN	-2.8353	2.3096	9.7618	1.78E-03	2.27E-02	0.1401
AMT	2.4849	0.6499	9.7356	1.81E-03	2.30E-02	5.5981
NXPE3	3.3271	1.6160	9.7296	1.81E-03	2.31E-02	10.0360
SRSF1	-2.6062	6.7615	9.7257	1.82E-03	2.31E-02	0.1642
PAK4	3.0844	2.1562	9.7212	1.82E-03	2.31E-02	8.4819
GIGYF2	-2.5511	4.1582	9.7166	1.83E-03	2.31E-02	0.1706
PPP1R13B	-2.7059	4.2899	9.7146	1.83E-03	2.31E-02	0.1533
TMEM130	3.0061	2.0815	9.7092	1.83E-03	2.32E-02	8.0341
GBP2	2.7790	3.6944	9.7074	1.84E-03	2.32E-02	6.8636
PI4KA*	2.4970	1.0550	9.6992	1.84E-03	2.32E-02	5.6452
HLA-DMA	1.9950	0.6622	9.7008	1.84E-03	2.32E-02	3.9860
LDB3	3.1312	0.9539	9.6920	1.85E-03	2.33E-02	8.7617
SLC25A11	-3.6652	2.4644	9.6903	1.85E-03	2.33E-02	0.0788
WIPF2	-1.7717	8.7401	9.6897	1.85E-03	2.33E-02	0.2929
INSIG2	-2.1743	1.6491	9.6884	1.85E-03	2.33E-02	0.2215
RP11-544A12.8	2.9034	0.7350	9.6878	1.85E-03	2.33E-02	7.4820
PKDCC	1.9167	0.7560	9.6843	1.86E-03	2.33E-02	3.7757
PCED1B-AS1	3.4090	0.8286	9.6789	1.86E-03	2.33E-02	10.6219
MXRA7	2.3126	0.9944	9.6786	1.86E-03	2.33E-02	4.9679
METTL16	2.9778	1.0889	9.6777	1.87E-03	2.33E-02	7.8776
MGAT4C*	3.7642	2.5802	9.6725	1.87E-03	2.33E-02	13.5876
METTL8	2.9393	1.1268	9.6672	1.88E-03	2.34E-02	7.6703
HDAC9	3.4690	4.3673	9.6629	1.88E-03	2.34E-02	11.0729
MANF	-3.0156	1.3861	9.6604	1.88E-03	2.34E-02	0.1237
ANO8	2.8626	0.9930	9.6565	1.89E-03	2.34E-02	7.2733
BRD9	2.3941	0.7757	9.6429	1.90E-03	2.36E-02	5.2564
RPS3A	2.4400	2.0559	9.6421	1.90E-03	2.36E-02	5.4263
WFDC2	2.8868	4.3941	9.6377	1.91E-03	2.36E-02	7.3962
MVK	3.1305	1.2458	9.6377	1.91E-03	2.36E-02	8.7572
LRRC37B	2.5459	0.8217	9.6282	1.92E-03	2.37E-02	5.8397
TMCO5B	4.3885	1.2050	9.6219	1.92E-03	2.37E-02	20.9452

PRSS12	2.7358	1.4309	9.6199	1.92E-03	2.37E-02	6.6615
RP11-350J20.5	3.5756	1.1705	9.6196	1.93E-03	2.37E-02	11.9221
KLHL32	2.3252	0.7338	9.5964	1.95E-03	2.40E-02	5.0112
ZNF37BP	3.2529	1.1777	9.5887	1.96E-03	2.40E-02	9.5330
CEP250	2.1813	0.8271	9.5877	1.96E-03	2.40E-02	4.5356
SSX2IP	-3.5244	2.1548	9.5749	1.97E-03	2.42E-02	0.0869
DNAJC8	2.5039	1.0807	9.5675	1.98E-03	2.42E-02	5.6720
SCAMP1-AS1	2.9751	1.1520	9.5654	1.98E-03	2.42E-02	7.8631
DACH2	2.0825	0.6032	9.5641	1.98E-03	2.42E-02	4.2355
RP11-649A18.5	3.7436	1.1279	9.5594	1.99E-03	2.43E-02	13.3949
ETV1	3.6325	1.6775	9.5500	2.00E-03	2.44E-02	12.4017
SYT15	2.2911	1.0060	9.5492	2.00E-03	2.44E-02	4.8944
CFLAR	1.9103	6.0449	9.5446	2.01E-03	2.44E-02	3.7588
CFB	-3.4502	1.0046	9.5385	2.01E-03	2.45E-02	0.0915
ZCCHC4	3.1209	0.7442	9.5306	2.02E-03	2.45E-02	8.6994
RP11-90P13.1	3.0562	1.0786	9.5283	2.02E-03	2.45E-02	8.3180
MAGI2-AS3	2.7557	1.8660	9.5232	2.03E-03	2.46E-02	6.7537
TIPARP	3.5292	3.4004	9.5213	2.03E-03	2.46E-02	11.5446
GPR64	3.7692	0.9320	9.5212	2.03E-03	2.46E-02	13.6343
RP1-28O17.1	3.5583	0.7802	9.5181	2.03E-03	2.46E-02	11.7799
FOXC1	-4.6639	1.5090	9.5145	2.04E-03	2.46E-02	0.0394
UTP6	3.9814	1.6085	9.5062	2.05E-03	2.47E-02	15.7948
UBR5	-2.8289	3.8096	9.5036	2.05E-03	2.47E-02	0.1407
AC012358.8	4.2520	1.4963	9.4998	2.05E-03	2.47E-02	19.0539
DDHD1	4.0703	1.6954	9.4988	2.06E-03	2.47E-02	16.7995
CTA-211A9.5	3.7374	1.2567	9.4910	2.06E-03	2.48E-02	13.3370
CERKL	4.1779	1.2436	9.4907	2.07E-03	2.48E-02	18.1002
ADAMTS6	3.9961	1.0892	9.4879	2.07E-03	2.48E-02	15.9565
C1orf186	2.2542	0.6254	9.4855	2.07E-03	2.48E-02	4.7708
TUBA1A	3.1902	1.7323	9.4822	2.07E-03	2.48E-02	9.1272
AMBRA1	-3.5081	4.7170	9.4793	2.08E-03	2.48E-02	0.0879
PGRMC2	-2.1862	5.6041	9.4702	2.09E-03	2.49E-02	0.2197
SNORA70	5.3181	1.6693	9.4576	2.10E-03	2.50E-02	39.8944
ASH2L	3.3001	3.6589	9.4563	2.10E-03	2.50E-02	9.8496
MTURN	-2.9778	3.2783	9.4556	2.11E-03	2.50E-02	0.1269
TTLL9	2.6748	1.0353	9.4449	2.12E-03	2.51E-02	6.3856
CCBE1	3.5734	1.3049	9.4420	2.12E-03	2.51E-02	11.9046
VPS26B	-2.9528	1.7878	9.4413	2.12E-03	2.51E-02	0.1292
GAREML	3.1158	0.8267	9.4386	2.12E-03	2.52E-02	8.6683
SLC26A5	3.0085	0.6784	9.4344	2.13E-03	2.52E-02	8.0474
CTD-2310F14.1	4.6173	2.7873	9.4339	2.13E-03	2.52E-02	24.5446

UBE2D4	3.0756	1.5345	9.4278	2.14E-03	2.52E-02	8.4306
SF3B4	-2.3085	0.7473	9.4255	2.14E-03	2.52E-02	0.2019
TOP3A	3.3620	2.0278	9.4236	2.14E-03	2.52E-02	10.2816
SCLY	2.4686	0.8427	9.4234	2.14E-03	2.52E-02	5.5352
FIGN	3.4806	1.2536	9.4162	2.15E-03	2.53E-02	11.1625
SNX29	2.5793	1.5002	9.4098	2.16E-03	2.53E-02	5.9764
CDH5	2.6549	2.1652	9.4063	2.16E-03	2.54E-02	6.2982
H3F3B	2.1091	11.6121	9.4018	2.17E-03	2.54E-02	4.3143
DNAJC28	3.7219	1.2505	9.3841	2.19E-03	2.56E-02	13.1952
PDE2A	2.3004	0.7807	9.3830	2.19E-03	2.56E-02	4.9259
CNFN	-2.5664	3.4473	9.3810	2.19E-03	2.56E-02	0.1688
PAK1	-2.2924	5.2411	9.3796	2.19E-03	2.56E-02	0.2041
SRP19	-3.1933	3.2953	9.3765	2.20E-03	2.56E-02	0.1093
ATP8B2	3.2046	1.1829	9.3710	2.20E-03	2.57E-02	9.2190
SIGLEC9	3.7602	0.9723	9.3647	2.21E-03	2.57E-02	13.5498
SAMD15*	2.6368	1.1090	9.3626	2.21E-03	2.57E-02	6.2195
RP11-277P12.20	3.4055	2.3436	9.3608	2.22E-03	2.57E-02	10.5966
RN7SKP38	2.2591	0.6222	9.3567	2.22E-03	2.58E-02	4.7869
UBXN4	2.5583	3.1183	9.3515	2.23E-03	2.58E-02	5.8901
UNC50	-2.8453	0.8848	9.3507	2.23E-03	2.58E-02	0.1391
TNR	3.3786	2.0392	9.3434	2.24E-03	2.59E-02	10.4010
TTF2	2.6833	1.0902	9.3406	2.24E-03	2.59E-02	6.4231
SNHG14	3.6784	2.8691	9.3332	2.25E-03	2.60E-02	12.8025
MTCH1	-2.2046	5.9094	9.3330	2.25E-03	2.60E-02	0.2170
C1RL	3.0736	0.9889	9.3170	2.27E-03	2.62E-02	8.4189
MIR143HG	2.0931	0.6488	9.3153	2.27E-03	2.62E-02	4.2667
TSTA3	2.8355	1.9574	9.3075	2.28E-03	2.62E-02	7.1381
CEP70	-2.0876	0.9620	9.3023	2.29E-03	2.63E-02	0.2353
DGKG	2.2415	0.6706	9.2984	2.29E-03	2.63E-02	4.7289
ERVW-1	2.5206	2.1418	9.2944	2.30E-03	2.64E-02	5.7384
CDC20B	2.0010	0.6980	9.2924	2.30E-03	2.64E-02	4.0027
DENND2D	-3.2362	2.3634	9.2842	2.31E-03	2.64E-02	0.1061
SLAMF7	2.6638	1.2365	9.2842	2.31E-03	2.64E-02	6.3369
RP11-736N17.4	3.7693	1.0415	9.2782	2.32E-03	2.65E-02	13.6352
GAK	-2.3437	3.9272	9.2752	2.32E-03	2.65E-02	0.1970
FANCA	2.4415	0.9610	9.2719	2.33E-03	2.65E-02	5.4319
RGS6	2.7753	1.0326	9.2702	2.33E-03	2.65E-02	6.8462
PLEKHG5	2.8116	0.7955	9.2665	2.33E-03	2.65E-02	7.0204
RP11-123C21.2	4.5265	1.5455	9.2543	2.35E-03	2.67E-02	23.0470
RP4-564F22.5	3.3869	0.9944	9.2496	2.36E-03	2.67E-02	10.4606
RP11-87E22.2	3.0058	1.1508	9.2483	2.36E-03	2.67E-02	8.0325

ELMO2	3.6104	2.1344	9.2482	2.36E-03	2.67E-02	12.2139
FAM160B1	-2.8509	3.9397	9.2347	2.37E-03	2.69E-02	0.1386
SNAP25	2.2488	0.6802	9.2315	2.38E-03	2.69E-02	4.7528
EDN1	-2.1788	5.1179	9.2315	2.38E-03	2.69E-02	0.2209
RP11-51J9.5	2.7421	1.2515	9.2255	2.39E-03	2.69E-02	6.6902
GNPDA1	-2.9287	3.1211	9.2245	2.39E-03	2.69E-02	0.1313
NDUFA6-AS1	1.9861	0.6333	9.2244	2.39E-03	2.69E-02	3.9617
FH	2.3441	0.8128	9.2237	2.39E-03	2.69E-02	5.0774
SPCS3	-2.4091	7.8661	9.2233	2.39E-03	2.69E-02	0.1883
LOXL2	2.9654	1.2759	9.2095	2.41E-03	2.70E-02	7.8106
SLC12A4	2.4125	0.5879	9.2085	2.41E-03	2.70E-02	5.3238
NAA35	3.4414	1.3879	9.2085	2.41E-03	2.70E-02	10.8630
RAP2A	-2.6538	4.4258	9.2084	2.41E-03	2.70E-02	0.1589
SLC22A18	1.9796	1.4234	9.2024	2.42E-03	2.70E-02	3.9439
CATIP	3.2977	1.1897	9.1590	2.48E-03	2.77E-02	9.8335
ZNF827	2.3548	1.9105	9.1552	2.48E-03	2.77E-02	5.1152
CTD-3131K8.3	3.1197	1.2313	9.1454	2.49E-03	2.78E-02	8.6921
DGCR6L	-2.1152	0.6445	9.1449	2.49E-03	2.78E-02	0.2308
C9orf91	2.8969	0.7895	9.1442	2.50E-03	2.78E-02	7.4481
PCBP1-AS1	-1.7890	5.1866	9.1414	2.50E-03	2.78E-02	0.2894
STAG3L3	3.9576	1.5118	9.1350	2.51E-03	2.79E-02	15.5363
ADAMDEC1	2.5210	0.8203	9.1342	2.51E-03	2.79E-02	5.7397
MAP2	3.2094	1.6136	9.1317	2.51E-03	2.79E-02	9.2498
PLIN1	3.7824	1.4462	9.1204	2.53E-03	2.80E-02	13.7600
RAE1	4.3424	1.2976	9.1184	2.53E-03	2.80E-02	20.2851
IREB2	-2.7684	3.4835	9.1133	2.54E-03	2.81E-02	0.1468
PSMB5	-2.4637	2.9302	9.1110	2.54E-03	2.81E-02	0.1813
PTGR1	2.4092	2.4332	9.0982	2.56E-03	2.82E-02	5.3119
HIAT1	3.0144	0.8605	9.0941	2.56E-03	2.83E-02	8.0805
DVL2	2.2945	0.7915	9.0909	2.57E-03	2.83E-02	4.9059
TK2	1.9769	0.6154	9.0894	2.57E-03	2.83E-02	3.9363
PLAT	3.1160	2.3118	9.0839	2.58E-03	2.83E-02	8.6697
PLA2G16	4.2855	2.6181	9.0832	2.58E-03	2.83E-02	19.5012
HIST1H3B	7.0434	5.2724	9.0782	2.59E-03	2.84E-02	131.9127
DPY19L2P1	2.0473	0.7600	9.0686	2.60E-03	2.85E-02	4.1334
SPTLC3*	2.1495	0.7713	9.0544	2.62E-03	2.87E-02	4.4367
CSF3R	3.1135	4.5354	9.0545	2.62E-03	2.87E-02	8.6545
DPP10	2.7803	0.9812	9.0430	2.64E-03	2.88E-02	6.8700
ZNF180	2.2500	0.6318	9.0385	2.64E-03	2.89E-02	4.7568
RP11-941H19.2	6.6829	5.0871	9.0309	2.65E-03	2.90E-02	102.7464
IL1RN	2.1336	10.9790	9.0047	2.69E-03	2.94E-02	4.3881

ZIC5	3.6725	1.3439	8.9884	2.72E-03	2.96E-02	12.7507
LBR	-2.2078	4.5319	8.9806	2.73E-03	2.97E-02	0.2165
SOX11	2.8010	4.2177	8.9740	2.74E-03	2.97E-02	6.9691
CCL3	3.3679	2.0473	8.9735	2.74E-03	2.97E-02	10.3238
WWC2	2.4302	0.7886	8.9724	2.74E-03	2.97E-02	5.3897
RP11-575A19.2	-3.1987	1.0792	8.9659	2.75E-03	2.98E-02	0.1089
HOXB8	2.7021	0.8201	8.9434	2.78E-03	3.01E-02	6.5076
ERMP1	3.3354	2.3868	8.9428	2.79E-03	3.01E-02	10.0937
MSANTD4	-3.2777	0.8123	8.9408	2.79E-03	3.01E-02	0.1031
PRR15L	-2.0142	3.5777	8.9371	2.79E-03	3.02E-02	0.2475
ARHGEF28	3.0018	1.3847	8.9337	2.80E-03	3.02E-02	8.0098
PBX3	3.8158	1.4129	8.9196	2.82E-03	3.04E-02	14.0826
HEATR6	1.9615	0.6524	8.9176	2.82E-03	3.04E-02	3.8947
RSRC2	-2.6145	2.7386	8.9160	2.83E-03	3.04E-02	0.1633
SOX4	2.4802	10.5198	8.9124	2.83E-03	3.05E-02	5.5796
RP11-849H4.2	4.2777	1.3252	8.9101	2.84E-03	3.05E-02	19.3958
ZNF280D	3.4179	2.5196	8.9021	2.85E-03	3.06E-02	10.6880
COPS8	2.4625	0.8599	8.8994	2.85E-03	3.06E-02	5.5115
ZFP30	2.9555	1.1308	8.8979	2.85E-03	3.06E-02	7.7567
CERS6-AS1	2.3477	0.7153	8.8967	2.86E-03	3.06E-02	5.0902
AMY2B	2.4610	2.7110	8.8907	2.87E-03	3.06E-02	5.5062
ZDHHC4	-2.4144	0.8407	8.8890	2.87E-03	3.06E-02	0.1876
PPP1R7	-3.3607	2.0656	8.8658	2.91E-03	3.10E-02	0.0973
KREMEN1	3.5431	1.5652	8.8613	2.91E-03	3.11E-02	11.6567
MSH2	2.9395	2.8211	8.8441	2.94E-03	3.13E-02	7.6715
TMEM9B	-2.2474	6.4727	8.8429	2.94E-03	3.13E-02	0.2106
CWC25	-3.0662	2.6721	8.8416	2.94E-03	3.13E-02	0.1194
RNF39	-2.5004	0.8139	8.8410	2.95E-03	3.13E-02	0.1767
ACTL6A	2.4710	0.7837	8.8390	2.95E-03	3.13E-02	5.5445
RP11-56P23.1	1.8912	0.5640	8.8315	2.96E-03	3.14E-02	3.7095
TNN	2.0315	0.6597	8.8301	2.96E-03	3.14E-02	4.0882
DPY19L2P2	1.9186	0.6488	8.8251	2.97E-03	3.14E-02	3.7806
ZNF280B	2.4659	1.2141	8.8201	2.98E-03	3.15E-02	5.5246
SYNE3	3.4715	1.6854	8.8189	2.98E-03	3.15E-02	11.0925
GGCT	-3.0697	1.5253	8.8160	2.99E-03	3.15E-02	0.1191
KLRD1	3.0877	1.4875	8.8122	2.99E-03	3.15E-02	8.5014
CEP41	3.3018	1.6029	8.8113	2.99E-03	3.15E-02	9.8618
MMP1	-3.4073	1.2492	8.8096	3.00E-03	3.15E-02	0.0943
PRRX1	4.8248	5.0280	8.8044	3.01E-03	3.16E-02	28.3400
RAB26	2.3769	0.7341	8.8030	3.01E-03	3.16E-02	5.1942
TMEM51	2.6211	2.4186	8.7925	3.02E-03	3.18E-02	6.1522

MFSD1	2.6136	1.8566	8.7872	3.03E-03	3.18E-02	6.1204
POM121L9P	3.0923	1.2830	8.7807	3.04E-03	3.19E-02	8.5288
RP11-10N16.2	1.8385	0.5385	8.7717	3.06E-03	3.20E-02	3.5765
TRIM71	2.6332	0.8223	8.7676	3.07E-03	3.21E-02	6.2039
EIF4A3	3.3044	4.3776	8.7627	3.07E-03	3.21E-02	9.8790
RP11-426L16.3	1.9191	0.7980	8.7563	3.09E-03	3.22E-02	3.7820
SLCO5A1	3.8352	2.2607	8.7528	3.09E-03	3.22E-02	14.2727
KIAA1107	2.7597	0.8500	8.7423	3.11E-03	3.24E-02	6.7728
KIAA1211L	2.4324	0.6140	8.7404	3.11E-03	3.24E-02	5.3981
CACNA1B	2.6162	1.0282	8.7403	3.11E-03	3.24E-02	6.1313
ENO3	1.9431	0.6718	8.7386	3.12E-03	3.24E-02	3.8452
ARHGEF9	3.2819	2.5894	8.7371	3.12E-03	3.24E-02	9.7267
FYB	2.9340	3.0030	8.7300	3.13E-03	3.25E-02	7.6422
URM1	-2.4515	1.1984	8.7283	3.13E-03	3.25E-02	0.1828
RP11-43N5.1	-3.3215	0.8916	8.7112	3.16E-03	3.27E-02	0.1000
AC106876.2	-3.0416	1.2567	8.7104	3.16E-03	3.27E-02	0.1214
SMEK1	-1.9256	7.7071	8.6910	3.20E-03	3.31E-02	0.2632
DNAJC6	1.9854	0.6719	8.6854	3.21E-03	3.31E-02	3.9598
ESYT1	-2.7610	3.3477	8.6819	3.21E-03	3.31E-02	0.1475
ATP6AP2	-2.7536	3.8709	8.6812	3.22E-03	3.31E-02	0.1483
PCMTD2	-3.2964	1.5537	8.6800	3.22E-03	3.31E-02	0.1018
IGF1	2.8998	1.4717	8.6738	3.23E-03	3.32E-02	7.4631
PRKRIRP9	2.8082	0.8884	8.6444	3.28E-03	3.37E-02	7.0040
CASC15	3.2417	0.7977	8.6386	3.29E-03	3.38E-02	9.4594
C5orf15	-2.2563	4.9362	8.6375	3.29E-03	3.38E-02	0.2093
AC091969.1	2.0569	0.7122	8.6346	3.30E-03	3.38E-02	4.1609
SCIMP	2.1024	0.6219	8.6237	3.32E-03	3.40E-02	4.2941
CLVS1	1.8770	0.5763	8.6195	3.33E-03	3.40E-02	3.6731
UNC79	2.5872	1.1657	8.6157	3.33E-03	3.40E-02	6.0093
ACSBG2	2.3057	0.9635	8.6157	3.33E-03	3.40E-02	4.9440
RP11-118B18.1	2.0310	0.7752	8.6150	3.33E-03	3.40E-02	4.0868
ZNF483	3.0417	1.8312	8.6137	3.34E-03	3.40E-02	8.2345
KLF9	2.6432	3.4014	8.6052	3.35E-03	3.42E-02	6.2474
PTGR2	2.5627	1.1148	8.5985	3.36E-03	3.43E-02	5.9082
OSTCP1	4.0597	1.3386	8.5900	3.38E-03	3.44E-02	16.6765
WWTR1	-2.0450	4.2458	8.5864	3.39E-03	3.44E-02	0.2423
AC007040.11	2.6869	0.8819	8.5864	3.39E-03	3.44E-02	6.4394
RP11-490M8.1	-2.5002	0.6855	8.5755	3.41E-03	3.46E-02	0.1767
C12orf77	2.1338	0.7311	8.5730	3.41E-03	3.46E-02	4.3888
C10orf53	3.6973	1.4475	8.5557	3.44E-03	3.49E-02	12.9714
FBXW4	2.5209	4.7300	8.5518	3.45E-03	3.49E-02	5.7392

UBA52P6	-2.0950	0.5880	8.5375	3.48E-03	3.52E-02	0.2341
TPRN	-2.1674	3.8377	8.5274	3.50E-03	3.53E-02	0.2226
RBFOX3	1.9322	0.7903	8.5255	3.50E-03	3.53E-02	3.8163
ELP5	3.0392	1.9665	8.5120	3.53E-03	3.56E-02	8.2205
RAPGEF5	-2.4293	2.2497	8.5081	3.54E-03	3.56E-02	0.1857
PAX8	3.1006	1.8695	8.5080	3.54E-03	3.56E-02	8.5780
SEC63P2	2.7408	0.7099	8.5072	3.54E-03	3.56E-02	6.6842
CTSC	2.9296	1.9498	8.5028	3.55E-03	3.56E-02	7.6190
PREP	-2.6535	3.4574	8.4990	3.55E-03	3.57E-02	0.1589
RP11-21L23.2	2.3740	0.6957	8.4979	3.56E-03	3.57E-02	5.1837
RNF185	-3.1051	2.2571	8.4959	3.56E-03	3.57E-02	0.1162
PDE4C	2.4877	3.0265	8.4880	3.57E-03	3.57E-02	5.6088
LRCH3	2.7851	1.7572	8.4878	3.58E-03	3.57E-02	6.8930
NOVA1	2.5413	2.2364	8.4872	3.58E-03	3.57E-02	5.8210
GALR1	4.5192	1.2428	8.4862	3.58E-03	3.57E-02	22.9313
ZBTB4	-2.9650	3.8360	8.4858	3.58E-03	3.57E-02	0.1281
GNPTAB	2.4687	1.6870	8.4854	3.58E-03	3.57E-02	5.5354
ZFYVE26	3.1109	1.5451	8.4783	3.59E-03	3.58E-02	8.6392
SGIP1	3.8455	0.8914	8.4778	3.60E-03	3.58E-02	14.3748
ANKRD18B	3.9440	3.0537	8.4746	3.60E-03	3.58E-02	15.3910
TNFSF14	3.8169	1.0471	8.4737	3.60E-03	3.58E-02	14.0926
VRK2	2.6031	0.8455	8.4667	3.62E-03	3.59E-02	6.0761
NAA38	1.5845	0.8981	8.4656	3.62E-03	3.59E-02	2.9991
CTC-457E21.1	4.2884	1.6661	8.4622	3.63E-03	3.59E-02	19.5412
ZNF431	3.1851	4.0117	8.4546	3.64E-03	3.61E-02	9.0949
CLDN7	-1.6249	5.8771	8.4508	3.65E-03	3.61E-02	0.3242
MYRIP	2.7083	1.4349	8.4493	3.65E-03	3.61E-02	6.5353
RILPL2	3.1926	3.4378	8.4381	3.67E-03	3.62E-02	9.1426
DMXL2	2.9616	1.7556	8.4377	3.68E-03	3.62E-02	7.7900
CORO7	4.0353	1.0920	8.4373	3.68E-03	3.62E-02	16.3961
USP48	-2.8750	1.3213	8.4355	3.68E-03	3.63E-02	0.1363
AC090099.1	3.2307	0.9963	8.4317	3.69E-03	3.63E-02	9.3874
CCDC144CP	2.7389	1.0645	8.4236	3.70E-03	3.64E-02	6.6755
MIA3	-2.1734	3.0739	8.4218	3.71E-03	3.64E-02	0.2217
FANCF	3.9886	2.9534	8.4203	3.71E-03	3.64E-02	15.8745
SMURF2	-2.6301	5.0664	8.4063	3.74E-03	3.67E-02	0.1615
ZNF326	-1.9674	5.2497	8.4017	3.75E-03	3.67E-02	0.2557
APOA1BP	-2.6527	2.5789	8.3902	3.77E-03	3.69E-02	0.1590
PDDC1	2.0303	0.6007	8.3800	3.79E-03	3.71E-02	4.0848
RP3-507I15.1	2.6630	1.8809	8.3783	3.80E-03	3.71E-02	6.3336
BRAT1	3.0241	0.9492	8.3758	3.80E-03	3.71E-02	8.1349

AC004156.3	3.1980	2.5657	8.3744	3.81E-03	3.71E-02	9.1766
HYKK	3.1130	1.1659	8.3662	3.82E-03	3.73E-02	8.6517
PDE4B	2.7525	2.0177	8.3643	3.83E-03	3.73E-02	6.7389
WDR3	2.9493	2.1821	8.3482	3.86E-03	3.76E-02	7.7237
PPIF	2.7488	5.5032	8.3458	3.87E-03	3.76E-02	6.7217
TSPAN15	-1.8929	5.6545	8.3430	3.87E-03	3.76E-02	0.2693
REV3L-IT1	2.7964	1.6974	8.3429	3.87E-03	3.76E-02	6.9472
RP11-689P11.3	3.4600	0.9528	8.3410	3.88E-03	3.76E-02	11.0045
DCAF12	-1.8190	6.7809	8.3279	3.90E-03	3.79E-02	0.2834
TAOK3	-1.7448	6.4994	8.3215	3.92E-03	3.79E-02	0.2984
GPC3	3.1354	1.0481	8.3213	3.92E-03	3.79E-02	8.7871
PSME2	-2.0958	3.0368	8.3162	3.93E-03	3.80E-02	0.2339
CARS2	2.2041	0.9123	8.3161	3.93E-03	3.80E-02	4.6078
SLC4A7	-2.8167	2.0909	8.3153	3.93E-03	3.80E-02	0.1419
BAALC-AS1	1.8594	0.5872	8.3044	3.95E-03	3.81E-02	3.6285
MRPL27	-2.6349	1.8754	8.3041	3.96E-03	3.81E-02	0.1610
NFKB1	2.2278	3.1741	8.3020	3.96E-03	3.81E-02	4.6843
FUT6	3.1415	0.9790	8.3016	3.96E-03	3.81E-02	8.8246
AC079612.2*	3.1409	1.7490	8.2749	4.02E-03	3.84E-02	8.8205
FOXO3*	-2.0472	5.2326	8.2746	4.02E-03	3.84E-02	0.2420
RP11-519G16.3	2.9914	1.4747	8.2864	3.99E-03	3.84E-02	7.9526
FABP5P15	2.9068	1.1652	8.2816	4.00E-03	3.84E-02	7.4995
C20orf166	3.3502	1.5372	8.2796	4.01E-03	3.84E-02	10.1981
APOBEC3G	3.0058	1.7745	8.2781	4.01E-03	3.84E-02	8.0321
RP11-611E13.2	-3.3015	0.9982	8.2773	4.01E-03	3.84E-02	0.1014
LMF2	-2.4816	0.8482	8.2769	4.02E-03	3.84E-02	0.1790
BCL2L14	2.0730	1.9329	8.2752	4.02E-03	3.84E-02	4.2076
SULT2B1	2.3159	0.7443	8.2746	4.02E-03	3.84E-02	4.9793
ANKRD20A8P	4.1910	1.9747	8.2546	4.06E-03	3.88E-02	18.2645
ZKSCAN8	3.2784	1.1391	8.2534	4.07E-03	3.88E-02	9.7030
DENR	-2.2293	3.5997	8.2520	4.07E-03	3.88E-02	0.2133
LCP2	2.5672	7.1436	8.2483	4.08E-03	3.88E-02	5.9267
RP4-601P9.1	1.9040	0.6222	8.2368	4.10E-03	3.90E-02	3.7424
CC2D2A	3.6323	1.2319	8.2367	4.11E-03	3.90E-02	12.4003
RP11-680B3.2	3.3599	1.7058	8.2338	4.11E-03	3.90E-02	10.2670
SLC12A9	3.1734	1.8065	8.2332	4.11E-03	3.90E-02	9.0215
GPC4	3.4939	0.9413	8.2328	4.11E-03	3.90E-02	11.2662
RP11-319G9.3	2.9280	1.9458	8.2289	4.12E-03	3.90E-02	7.6105
XKR9	3.3955	0.9951	8.2199	4.14E-03	3.92E-02	10.5230
LINC00511	-2.7196	2.8301	8.2094	4.17E-03	3.94E-02	0.1518
TMEM125	-3.4119	1.7578	8.2014	4.19E-03	3.95E-02	0.0940

PALM2	3.4777	1.5743	8.2001	4.19E-03	3.95E-02	11.1405
APOOL	2.5561	8.8316	8.1959	4.20E-03	3.96E-02	5.8813
NLRP2	3.7796	0.9959	8.1939	4.20E-03	3.96E-02	13.7332
NDUFA2	2.1216	2.7631	8.1928	4.21E-03	3.96E-02	4.3517
GON4L	2.9793	2.3090	8.1922	4.21E-03	3.96E-02	7.8861
NCKAP1L	3.0360	1.0801	8.1881	4.22E-03	3.96E-02	8.2022
CBLN2	2.6974	0.8868	8.1876	4.22E-03	3.96E-02	6.4864
ARHGEF3	3.4986	1.1600	8.1871	4.22E-03	3.96E-02	11.3030
RP5-1125A11.1	-3.4865	1.0084	8.1819	4.23E-03	3.97E-02	0.0892
ANKRD44	2.9199	2.2091	8.1767	4.24E-03	3.97E-02	7.5680
FAM198B	-3.0293	3.4168	8.1683	4.26E-03	3.99E-02	0.1225
ZNF556	2.1716	0.7260	8.1652	4.27E-03	3.99E-02	4.5053
FLII	-3.0032	1.2944	8.1648	4.27E-03	3.99E-02	0.1247
AC010987.5	3.3016	2.0906	8.1576	4.29E-03	4.00E-02	9.8598
HIPK1-AS1	2.9204	0.9630	8.1561	4.29E-03	4.00E-02	7.5705
ERICH1	2.3728	0.9724	8.1553	4.29E-03	4.00E-02	5.1795
RNU6-428P	5.2044	5.1318	8.1526	4.30E-03	4.00E-02	36.8708
PABPN1	-1.4774	9.5267	8.1513	4.30E-03	4.00E-02	0.3591
SEPT10	2.4414	3.7238	8.1489	4.31E-03	4.01E-02	5.4318
ETV7	1.9690	0.5950	8.1427	4.32E-03	4.02E-02	3.9148
ENTPD1-AS1	3.0047	1.7144	8.1314	4.35E-03	4.04E-02	8.0263
FAM133A*	2.4338	1.7707	8.1242	4.37E-03	4.05E-02	5.4031
TRIM27	1.7550	1.4844	8.1190	4.38E-03	4.06E-02	3.3753
LINC01207	-1.9331	5.3292	8.1157	4.39E-03	4.06E-02	0.2619
TRIM23	2.8901	1.3765	8.1107	4.40E-03	4.07E-02	7.4131
LINC00672	3.2152	3.2657	8.0992	4.43E-03	4.09E-02	9.2866
GABRA3	1.8348	0.6336	8.0992	4.43E-03	4.09E-02	3.5671
CDIP1	-2.5628	1.7206	8.0986	4.43E-03	4.09E-02	0.1692
SCUBE2	1.9831	0.7786	8.0963	4.44E-03	4.09E-02	3.9534
GABRA4	1.9355	0.8133	8.0945	4.44E-03	4.09E-02	3.8250
RP11-724N1.1	2.1196	1.3052	8.0907	4.45E-03	4.10E-02	4.3457
WDR47	-3.4707	1.4784	8.0881	4.46E-03	4.10E-02	0.0902
NLRP9	1.8242	0.5390	8.0822	4.47E-03	4.11E-02	3.5411
DPF3	3.6570	1.7124	8.0812	4.47E-03	4.11E-02	12.6146
SLC7A1	-2.8969	4.7118	8.0808	4.47E-03	4.11E-02	0.1343
MTMR4	-2.8350	2.3070	8.0712	4.50E-03	4.12E-02	0.1401
YTHDF1	-2.8385	4.1293	8.0698	4.50E-03	4.12E-02	0.1398
GEN1*	2.4855	1.1685	8.0660	4.51E-03	4.13E-02	5.6003
TXNRD1*	-1.8167	8.0706	8.0595	4.53E-03	4.13E-02	0.2839
BTBD3	-2.6954	4.3068	8.0666	4.51E-03	4.13E-02	0.1544
OLFM2	4.5849	1.2624	8.0638	4.52E-03	4.13E-02	23.9991

DUOX2	1.8749	7.9996	8.0633	4.52E-03	4.13E-02	3.6677
WDFY3	2.6648	1.9511	8.0597	4.53E-03	4.13E-02	6.3415
ROR1	1.9645	0.7991	8.0590	4.53E-03	4.13E-02	3.9027
ZNF226	2.5937	1.0547	8.0575	4.53E-03	4.13E-02	6.0364
LPCAT2	3.3219	1.2513	8.0523	4.54E-03	4.13E-02	9.9996
TIFA	3.6280	2.0967	8.0518	4.55E-03	4.13E-02	12.3630
ADAP2	2.3120	0.8041	8.0441	4.57E-03	4.15E-02	4.9656
ARL11	2.6264	1.4846	8.0390	4.58E-03	4.15E-02	6.1749
UBA2	2.8021	2.6308	8.0385	4.58E-03	4.15E-02	6.9745
ARRDC5	3.7745	1.5581	8.0384	4.58E-03	4.15E-02	13.6846
TWISTNB	3.2338	1.0795	8.0342	4.59E-03	4.16E-02	9.4076
ELK1	3.3411	2.1112	8.0262	4.61E-03	4.17E-02	10.1338
SH2D4A	2.6427	1.6781	8.0251	4.61E-03	4.17E-02	6.2452
PDAP1	-3.1715	2.4704	8.0250	4.61E-03	4.17E-02	0.1110
BNIP3P1	3.1883	1.1979	8.0166	4.64E-03	4.18E-02	9.1155
SHFM1	2.3123	2.5547	8.0145	4.64E-03	4.19E-02	4.9667
FAM230A	1.8100	0.5489	8.0023	4.67E-03	4.21E-02	3.5063
HHIP	2.7370	1.9051	7.9989	4.68E-03	4.21E-02	6.6670
TIMM10B	-2.8489	3.6788	7.9971	4.69E-03	4.21E-02	0.1388
FMN2	-3.5088	2.3313	7.9971	4.69E-03	4.21E-02	0.0879
DYNC1H1	2.5576	3.5503	7.9956	4.69E-03	4.21E-02	5.8871
IL4R	-2.7689	1.9872	7.9947	4.69E-03	4.21E-02	0.1467
FBXL3	2.4592	3.4906	7.9931	4.70E-03	4.21E-02	5.4991
ELP2	2.5534	1.2192	7.9909	4.70E-03	4.22E-02	5.8700
GRIA3	1.9203	0.5820	7.9867	4.71E-03	4.22E-02	3.7851
CHRNG	1.8484	0.8130	7.9844	4.72E-03	4.22E-02	3.6010
IP6K2	1.5991	6.3893	7.9807	4.73E-03	4.23E-02	3.0295
NKTR	2.9756	2.2623	7.9803	4.73E-03	4.23E-02	7.8660
TUBGCP3	-3.3623	1.0057	7.9776	4.74E-03	4.23E-02	0.0972
MYLIP	-2.2615	6.3821	7.9734	4.75E-03	4.24E-02	0.2086
NETO2	3.3044	1.2379	7.9700	4.76E-03	4.24E-02	9.8795
LINC00910	2.4312	0.7627	7.9664	4.77E-03	4.24E-02	5.3936
DCUN1D2	-3.1868	3.0290	7.9661	4.77E-03	4.24E-02	0.1098
RP11-654C22.1	-4.3508	1.0916	7.9608	4.78E-03	4.25E-02	0.0490
SARS	-2.5860	2.8853	7.9594	4.78E-03	4.25E-02	0.1665
HARS	1.8612	0.5640	7.9591	4.78E-03	4.25E-02	3.6330
RFNG	-2.4651	1.1914	7.9523	4.80E-03	4.26E-02	0.1811
RHOG	2.2071	4.3149	7.9495	4.81E-03	4.26E-02	4.6175
RNASEH2B	3.5378	1.6550	7.9491	4.81E-03	4.26E-02	11.6143
TMPRSS11E	2.6709	9.1632	7.9463	4.82E-03	4.27E-02	6.3684
ST6GALNAC1	-1.7773	4.2871	7.9405	4.83E-03	4.28E-02	0.2917

AL109658.1	3.0767	1.4195	7.9361	4.85E-03	4.28E-02	8.4367
RP11-613D13.8	1.9399	0.6817	7.9318	4.86E-03	4.29E-02	3.8369
COMMD1	-2.9362	0.9685	7.9253	4.87E-03	4.30E-02	0.1307
RPH3AL	4.3672	1.5001	7.9201	4.89E-03	4.31E-02	20.6382
CBFA2T3	-3.0544	1.9054	7.9183	4.89E-03	4.31E-02	0.1204
ABHD15	3.7555	1.6383	7.9171	4.90E-03	4.31E-02	13.5057
WDR12	3.7306	2.3623	7.9137	4.91E-03	4.31E-02	13.2749
GOPC	-1.8691	4.7793	7.9137	4.91E-03	4.31E-02	0.2737
RP11-490O6.2	1.9319	0.5808	7.9117	4.91E-03	4.31E-02	3.8155
BLM	2.2401	0.8541	7.9108	4.91E-03	4.31E-02	4.7242
RP11-464D20.6	2.7315	0.8881	7.9106	4.91E-03	4.31E-02	6.6414
TCTN2	2.4623	1.0296	7.9068	4.92E-03	4.32E-02	5.5108
CD109	1.8896	0.6908	7.9027	4.94E-03	4.32E-02	3.7053
RP11-18H21.1	2.2529	0.7752	7.9016	4.94E-03	4.32E-02	4.7665
SLC9A3R2	-2.6434	0.8389	7.8983	4.95E-03	4.33E-02	0.1600
TGIF2	2.5545	0.7918	7.8954	4.96E-03	4.33E-02	5.8746
KIAA0907	2.3467	3.3704	7.8951	4.96E-03	4.33E-02	5.0867
MGA	3.1931	1.7826	7.8924	4.96E-03	4.33E-02	9.1455
POLR2I	3.6652	1.6227	7.8883	4.98E-03	4.34E-02	12.6865
SCAI	2.6653	1.9456	7.8850	4.98E-03	4.34E-02	6.3436
NTNG2	2.8953	1.2070	7.8836	4.99E-03	4.34E-02	7.4400
RP11-390E23.6	-2.8602	2.7054	7.8795	5.00E-03	4.35E-02	0.1377
HOXC6	1.9334	0.6114	7.8751	5.01E-03	4.36E-02	3.8195
RAI1	2.8015	1.1033	7.8669	5.03E-03	4.38E-02	6.9718
CEP290	2.2126	0.6982	7.8605	5.05E-03	4.39E-02	4.6352
NDUFS6	-2.8806	2.8673	7.8575	5.06E-03	4.39E-02	0.1358
SLC37A2	-2.3959	1.0806	7.8547	5.07E-03	4.39E-02	0.1900
SEMA3D	1.8870	0.6053	7.8486	5.09E-03	4.41E-02	3.6986
NTN4	2.5208	1.4846	7.8406	5.11E-03	4.42E-02	5.7391
SRRT	-2.9790	2.7547	7.8365	5.12E-03	4.43E-02	0.1268
RANBP9	-2.0947	3.0037	7.8323	5.13E-03	4.43E-02	0.2341
SCGB2A2	1.9068	0.6677	7.8304	5.14E-03	4.43E-02	3.7497
AKIP1	-4.0015	1.1385	7.8297	5.14E-03	4.43E-02	0.0624
MYLK3	3.3071	1.4318	7.8294	5.14E-03	4.43E-02	9.8981
SESN3	-2.7793	5.0291	7.8278	5.14E-03	4.43E-02	0.1457
WDR66	2.4678	2.5275	7.8238	5.16E-03	4.44E-02	5.5320
GRIN2B*	3.1569	1.6430	7.8173	5.17E-03	4.45E-02	8.9193
PLXNB2	2.1160	3.3324	7.8169	5.18E-03	4.45E-02	4.3349
C18orf61	2.4102	1.2705	7.8147	5.18E-03	4.45E-02	5.3155
LRMP	-3.5483	1.2876	7.8140	5.18E-03	4.45E-02	0.0855
HCLS1	2.4797	1.0835	7.8130	5.19E-03	4.45E-02	5.5777

METTL6	3.6616	2.0227	7.8124	5.19E-03	4.45E-02	12.6551
ZGRF1	4.2580	1.5850	7.8117	5.19E-03	4.45E-02	19.1335
AGMO	2.8781	1.0217	7.7978	5.23E-03	4.48E-02	7.3520
SCFD1	2.2925	2.3854	7.7957	5.24E-03	4.48E-02	4.8989
FAM171A1	3.2259	2.3652	7.7949	5.24E-03	4.48E-02	9.3559
C10orf10	-2.2335	4.6274	7.7949	5.24E-03	4.48E-02	0.2126
COPS7A	-2.8300	2.0263	7.7788	5.29E-03	4.51E-02	0.1406
RSBN1L	-2.1537	5.0195	7.7756	5.30E-03	4.52E-02	0.2247
PROX1-AS1	2.6409	1.1147	7.7747	5.30E-03	4.52E-02	6.2370
AC093391.2	3.2998	1.7026	7.7720	5.31E-03	4.52E-02	9.8477
USP49	2.0479	0.7537	7.7564	5.35E-03	4.56E-02	4.1351
OR52N5	5.5908	1.7892	7.7539	5.36E-03	4.56E-02	48.1940
TM4SF1-AS1	-2.3806	0.7130	7.7519	5.37E-03	4.56E-02	0.1920
UTP11L	-3.7436	1.6821	7.7518	5.37E-03	4.56E-02	0.0747
RP11-372E1.6	2.5956	0.9283	7.7432	5.39E-03	4.57E-02	6.0443
CCPG1	2.1612	2.3929	7.7385	5.41E-03	4.58E-02	4.4728
NDE1	3.0041	1.5811	7.7336	5.42E-03	4.59E-02	8.0226
NFYB	-2.6868	2.9279	7.7316	5.43E-03	4.59E-02	0.1553
CLP1	-4.2308	1.0909	7.7274	5.44E-03	4.60E-02	0.0533
TEX10	4.6869	1.2680	7.7198	5.46E-03	4.62E-02	25.7571
FTSJ1	-1.9285	0.5692	7.7104	5.49E-03	4.64E-02	0.2627
BCAS4	3.0492	1.7141	7.7053	5.51E-03	4.65E-02	8.2777
RP11-154D6.2	2.3665	1.9062	7.6949	5.54E-03	4.67E-02	5.1567
ACADVL	-2.0349	2.8627	7.6896	5.55E-03	4.68E-02	0.2440
NME7	1.8195	0.7351	7.6856	5.57E-03	4.69E-02	3.5296
KBTBD11	-2.8003	1.3681	7.6806	5.58E-03	4.70E-02	0.1436
RPGRIP1L	2.7644	1.1255	7.6774	5.59E-03	4.70E-02	6.7946
LONP1	2.6305	1.3545	7.6697	5.62E-03	4.72E-02	6.1924
NSD1	2.8645	1.0804	7.6684	5.62E-03	4.72E-02	7.2831
GRPEL1	-1.5853	0.8258	7.6661	5.63E-03	4.72E-02	0.3333
PPP1R14D	-1.8689	4.2434	7.6642	5.63E-03	4.72E-02	0.2738
RP11-102K13.5	1.7379	0.7295	7.6636	5.63E-03	4.72E-02	3.3355
RAPGEF1	-2.0390	2.3350	7.6631	5.64E-03	4.72E-02	0.2433
GPR183	-4.2503	1.0076	7.6611	5.64E-03	4.72E-02	0.0525
OGFOD1	1.9572	0.7710	7.6572	5.65E-03	4.73E-02	3.8831
KIAA2018	2.0785	3.0776	7.6540	5.66E-03	4.73E-02	4.2238
FAM159B	3.4390	1.2672	7.6505	5.68E-03	4.74E-02	10.8454
TTC7A	-2.7902	3.5460	7.6488	5.68E-03	4.74E-02	0.1446
RP1-90J20.11	2.3907	0.8268	7.6483	5.68E-03	4.74E-02	5.2441
PROX1*	2.7916	2.3350	7.6436	5.70E-03	4.75E-02	6.9239
ALG9	3.0669	1.3806	7.6429	5.70E-03	4.75E-02	8.3797

ADIPOR2*	-1.8314	8.3881	7.6398	5.71E-03	4.75E-02	0.2810
PPP2R5C	1.7472	7.8956	7.6394	5.71E-03	4.75E-02	3.3570
LINC01268	3.0760	0.9308	7.6368	5.72E-03	4.75E-02	8.4329
RP11-505P4.6	1.7914	0.6795	7.6345	5.73E-03	4.75E-02	3.4615
RP11-411K7.1	1.7974	0.6810	7.6343	5.73E-03	4.75E-02	3.4758
ME2	-1.7824	4.2456	7.6335	5.73E-03	4.75E-02	0.2907
NLRP3	3.0961	1.3306	7.6335	5.73E-03	4.75E-02	8.5507
RNF217	3.3578	1.1180	7.6322	5.73E-03	4.75E-02	10.2521
PIK3R5	2.7210	0.8928	7.6282	5.75E-03	4.75E-02	6.5935
PRCP	2.6810	1.6412	7.6254	5.76E-03	4.76E-02	6.4130
SCNN1B	-2.5534	2.1324	7.6230	5.76E-03	4.76E-02	0.1704
PRR14L	1.9019	1.5713	7.6227	5.76E-03	4.76E-02	3.7371
MIR7-3HG	2.7344	0.8662	7.6127	5.80E-03	4.78E-02	6.6549
HNRNPA1P54	2.4581	3.1584	7.6078	5.81E-03	4.79E-02	5.4948
NUDT14	-2.5972	2.1540	7.6065	5.82E-03	4.79E-02	0.1653
PAPPA	2.7247	0.8692	7.5964	5.85E-03	4.81E-02	6.6103
SNAPC5	-3.1022	1.5164	7.5932	5.86E-03	4.82E-02	0.1164
MTA3	-2.1490	3.8641	7.5851	5.89E-03	4.84E-02	0.2255
HSPE1	2.2327	1.8372	7.5839	5.89E-03	4.84E-02	4.7002
LGALS2	-2.5512	2.4067	7.5734	5.92E-03	4.86E-02	0.1706
CHSY1	2.8092	1.6404	7.5733	5.92E-03	4.86E-02	7.0090
RALYL	2.3838	1.7068	7.5709	5.93E-03	4.86E-02	5.2190
HGSNAT	-2.9703	1.9167	7.5707	5.93E-03	4.86E-02	0.1276
EPHA1	3.3555	1.1399	7.5700	5.93E-03	4.86E-02	10.2356
XRCC5	2.2058	7.2278	7.5664	5.95E-03	4.86E-02	4.6134
RP11-88H9.2	2.4661	1.3761	7.5661	5.95E-03	4.86E-02	5.5255
TANC2	3.1862	1.3840	7.5617	5.96E-03	4.87E-02	9.1022
BLNK	3.4393	1.2914	7.5606	5.97E-03	4.87E-02	10.8474
RNF130	2.4451	2.7765	7.5601	5.97E-03	4.87E-02	5.4455
ANKMY1	2.9386	1.1299	7.5550	5.98E-03	4.88E-02	7.6669
PDLIM3	2.7538	1.9371	7.5529	5.99E-03	4.88E-02	6.7449
TFAM	2.6319	2.6320	7.5504	6.00E-03	4.88E-02	6.1983
EFCAB2	1.8848	0.5963	7.5472	6.01E-03	4.89E-02	3.6931
RPS6KC1	2.9469	1.1044	7.5384	6.04E-03	4.91E-02	7.7106
ASPN	1.7863	0.6843	7.5369	6.04E-03	4.91E-02	3.4493
QPRT	3.3668	2.9325	7.5365	6.05E-03	4.91E-02	10.3161
LYRM9	2.0259	0.7172	7.5355	6.05E-03	4.91E-02	4.0725
DHX29	-3.5864	1.7157	7.5338	6.06E-03	4.91E-02	0.0832
AC079354.3	4.3843	2.8206	7.5333	6.06E-03	4.91E-02	20.8839
MAPK13	-1.9490	4.2136	7.5311	6.06E-03	4.91E-02	0.2590
STYXL1	-2.9140	1.2953	7.5287	6.07E-03	4.91E-02	0.1327

RGS22	3.1636	1.0825	7.5272	6.08E-03	4.91E-02	8.9604
POLR3A	2.4930	0.7940	7.5266	6.08E-03	4.91E-02	5.6294
ARHGEF7	-2.3230	1.9358	7.5195	6.10E-03	4.93E-02	0.1999
CR1	2.7911	0.9015	7.5166	6.11E-03	4.93E-02	6.9213
MAD2L1	3.1089	1.3865	7.5151	6.12E-03	4.93E-02	8.6270
RAB21	1.6344	6.7634	7.5147	6.12E-03	4.93E-02	3.1047
MRPS33	-1.9845	0.6951	7.5134	6.12E-03	4.93E-02	0.2527
KIAA1244	-2.2990	1.8745	7.5101	6.14E-03	4.93E-02	0.2032
SLC30A8	2.7319	1.0276	7.5036	6.16E-03	4.95E-02	6.6433
NUP205	-3.0068	1.5480	7.5008	6.17E-03	4.95E-02	0.1244
ZNF530	1.7085	0.5546	7.4960	6.18E-03	4.96E-02	3.2683
SERPINB7	4.0489	1.4905	7.4958	6.18E-03	4.96E-02	16.5515
PAIP1	2.3757	1.3519	7.4933	6.19E-03	4.96E-02	5.1899
RP11-486P11.1	3.4195	0.9266	7.4913	6.20E-03	4.97E-02	10.6996
PLA2G7	2.6464	0.8385	7.4883	6.21E-03	4.97E-02	6.2610
SAMD12	2.8942	2.9396	7.4865	6.22E-03	4.97E-02	7.4341
RP11-14I17.2	3.5847	1.0550	7.4858	6.22E-03	4.97E-02	11.9980
CARS	-2.6969	1.2341	7.4838	6.23E-03	4.97E-02	0.1542
RP11-542K23.7	3.2762	1.0910	7.4832	6.23E-03	4.97E-02	9.6882
RASA2-IT1	2.1743	0.6408	7.4779	6.25E-03	4.98E-02	4.5135
TNKS2	-1.7505	7.0657	7.4733	6.26E-03	4.99E-02	0.2972

Table S5. Top 500 Single-Gene LDA Classifiers. The top 500 single-gene linear discriminant analysis classifiers and their respective misclassification rate in flaxseed lignan supplement vs placebo treatment in all samples regardless of the ENL phenotype.

Gene	bresub error	Gene	bresub error	Gene	bresub error	Gene	bresub error
PROX1	0.2845	F11R	0.3842	CASP3	0.4032	CTDSPL	0.4194
WNT5A	0.2913	EPHB1	0.3853	SLC7A7	0.4043	KDM4C	0.4200
KCTD12	0.3089	PSME2	0.3864	RHOB	0.4044	PRKCI	0.4200
TLR2	0.3172	IGF2BP1	0.3872	GEMIN6	0.4052	CAV1	0.4204
REL	0.3278	SERPINA1	0.3881	ATF4	0.4062	RBL2	0.4206
FOXO3	0.3373	KLF4	0.3883	SPRY2	0.4062	S100A9	0.4211
SPDEF	0.3377	HRH1	0.3885	FGFR3	0.4069	CDK8	0.4214
PTCH1	0.3437	SLCO2A1	0.3892	EPHB4	0.4071	AGPAT3	0.4214
GADD45B	0.3465	PYGO2	0.3893	TSTA3	0.4080	BCL2L2	0.4215
TRAFD1	0.3478	SLC30A4	0.3897	SORD	0.4081	DDR2	0.4221
CDH5	0.3512	DUOX2	0.3900	ACAT1	0.4085	CLDN3	0.4222
TJP3	0.3561	NEUROD1	0.3912	HOPX	0.4090	SLC30A5	0.4222
FGFR1	0.3609	TNFRSF21	0.3921	EPAS1	0.4094	REG1A	0.4223
MAML1	0.3626	BMPR2	0.3921	REST	0.4095	NOS2	0.4225
FAM129A	0.3660	SLC5A11	0.3927	HMOX1	0.4098	RAD52	0.4227
HES1	0.3667	ELP3	0.3929	JAK2	0.4098	SPPL3	0.4228
PTPN22	0.3673	XRCC6	0.3930	TDG	0.4100	TFAP2C	0.4233
MAPK8	0.3677	FUT2	0.3932	SLC7A6	0.4101	NPAT	0.4234
VDR	0.3680	P4HB	0.3942	PDE6D	0.4103	SLC9A2	0.4242
BMP4	0.3700	PLA2G10	0.3944	FOXD2	0.4104	AXIN1	0.4242
PECAM1	0.3702	TFRC	0.3944	ACSL4	0.4110	SLC2A13	0.4246
APC	0.3709	FGF18	0.3948	TNFRSF10D	0.4115	ADRB1	0.4252
DOT1L	0.3716	BST1	0.3953	SLC50A1	0.4126	TP53	0.4253
HOXA10	0.3718	ITLN1	0.3955	BMI1	0.4134	HOXA13	0.4257
PCNA	0.3754	IL13RA1	0.3956	BRD2	0.4140	TLR4	0.4259
PAX6	0.3756	IL6ST	0.3957	SLC20A1	0.4150	SHH	0.4259
IL18	0.3764	TTN	0.3957	SLC4A4	0.4153	MAPK6	0.4260
SLC39A7	0.3777	RUNX2	0.3970	SLC28A1	0.4157	APOA1	0.4262
TJP1	0.3789	NCAM1	0.3972	PPARGC1A	0.4164	WNT2B	0.4264
WNT7A	0.3793	DEFA5	0.3996	SOD2	0.4171	PKN2	0.4264
COX4I1	0.3799	MYB	0.4000	SREBF2	0.4180	LIG3	0.4265
RELB	0.3801	NOTCH3	0.4013	STAT3	0.4188	TGFB2	0.4270
TNK2	0.3816	PODXL	0.4015	CCL15	0.4192	CXCL2	0.4272
GSTM3	0.3827	PIWIL2	0.4027	GREM1	0.4192	SLC35A2	0.4276
MYO9B	0.3833	CA13	0.4030	PRKDC	0.4193	ADAM10	0.4280

Gene	bresub error	Gene	bresub error	Gene	bresub error	Gene	bresub error
CA3	0.4296	MFSD2A	0.4420	MGST1	0.4479	PRKCB	0.4556
HOXD10	0.4296	CA1	0.4422	TNFAIP1	0.4480	SLC3A1	0.4557
CXCL16	0.4297	MLLT4	0.4429	CYP4F2	0.4486	SLC6A6	0.4558
SLC5A8	0.4297	MUC17	0.4429	PARK7	0.4487	LAMA1	0.4558
ELF3	0.4297	SLC7A11	0.4429	HOXD13	0.4489	FOXC1	0.4559
AQP11	0.4298	GDF15	0.4433	TJP2	0.4489	IFNAR1	0.4560
TNFRSF1A	0.4301	RB1	0.4433	PLCD3	0.4490	SLC38A2	0.4563
PIK3CA	0.4302	BMF	0.4434	EPHB6	0.4491	IGF1R	0.4566
TFCP2L1	0.4309	GSN	0.4440	BCL2L13	0.4492	ATG16L1	0.4569
MAPK13	0.4316	RAC1	0.4440	PTGIR	0.4492	PPM1D	0.4570
MSH3	0.4320	YWHAB	0.4441	CD59	0.4493	PAWR	0.4570
CA14	0.4325	BCL2	0.4443	FASLG	0.4494	PTPN6	0.4575
TYRO3	0.4325	BCO2	0.4444	FABP6	0.4495	FFAR4	0.4582
ICAM1	0.4327	XIST	0.4444	IRF8	0.4498	SOCS6	0.4584
FOXP2	0.4334	FABP3	0.4444	NTS	0.4498	REG3A	0.4585
NOTCH1	0.4335	TNFRSF10A	0.4444	SLC31A1	0.4500	ABCG2	0.4587
CAMK2B	0.4342	TNFRSF11A	0.4444	MAPK10	0.4500	MYC	0.4589
EGF	0.4342	COX5B	0.4445	SMAD7	0.4500	PLA2G4B	0.4590
THRA	0.4343	RBPJ	0.4445	AQP7	0.4509	SLC25A28	0.4591
MEIS1	0.4365	KDM4B	0.4446	ITGAM	0.4511	SAT2	0.4593
NRIP1	0.4368	PRKCE	0.4446	KIT	0.4512	COMMD3	0.4595
HPGD	0.4370	RELA	0.4446	CTNNA1	0.4514	GUCA2B	0.4597
CDK2	0.4375	TCF4	0.4446	HMGCR	0.4514	MELK	0.4599
ID1	0.4380	REG4	0.4447	POU2F3	0.4514	ENC1	0.4602
SLC10A2	0.4380	ITGB1	0.4448	GJC1	0.4517	NUMB	0.4604
CAT	0.4381	HSD17B2	0.4454	ADAM17	0.4523	IL2RG	0.4607
GCNT3	0.4382	IL1B	0.4456	FOS	0.4523	MSH6	0.4608
ACADS	0.4382	ISL1	0.4458	FZD7	0.4524	MAP2K2	0.4609
HSPA1B	0.4383	FOXA3	0.4458	PLCB2	0.4533	RAD51D	0.4609
MGAT3	0.4394	ACKR2	0.4460	TNF	0.4537	HIST3H3	0.4609
DEFA6	0.4395	SLC23A3	0.4460	LRIG1	0.4538	ID3	0.4610
CDCP1	0.4396	TGM4	0.4460	DPYSL3	0.4539	SEPTIN4	0.4610
CA7	0.4401	NFKB1	0.4461	HOXB7	0.4542	DNAJB6	0.4612
TGFA	0.4404	PDK1	0.4463	CDK2AP1	0.4544	PLAU	0.4613
SAR1B	0.4409	COL6A3	0.4470	PRKCD	0.4546	PLCB4	0.4616
ILKAP	0.4411	FOXK2	0.4470	SLC39A9	0.4546	ECM1	0.4618
MPC1	0.4416	PDE4C	0.4471	DPP4	0.4552	SLC5A1	0.4618
AIFM2	0.4417	SLC16A3	0.4475	ATOH1	0.4553	FLT1	0.4619

Gene	bresub error
C11orf30	0.4621
CACNA2D1	0.4621
PCDH7	0.4622
BMP2	0.4623
ID4	0.4624
LGALS3	0.4624
FOXO1	0.4624
GSR	0.4624
HOXB6	0.4625
SMAD3	0.4625
LMNB2	0.4626
SIRT1	0.4626
LPAR4	0.4627
SLC1A4	0.4627
TCN2	0.4627
SLC25A39	0.4628
YWHAH	0.4628
KLK6	0.4629
PPARG	0.4629
ATG5	0.4629
CD44	0.4629
ESR1	0.4629
HOXA5	0.4629
PTBP1	0.4629
ACTB	0.4630
ALDH1A1	0.4630
APP	0.4630
AQP8	0.4630
BACH1	0.4630
BCL2L1	0.4630
CD9	0.4630
CDC42	0.4630
CDH2	0.4630
CDKN1A	0.4630
CLDN8	0.4630
COL3A1	0.4630
CSPG5	0.4630
CTNND1	0.4630

Gene	bresub error
CXCL3	0.4630
DAPK2	0.4630
DBN1	0.4630
DHRS9	0.4630
DLL4	0.4630
EGFR	0.4630
FABP2	0.4630
FASN	0.4630
FGF5	0.4630
HAS2	0.4630
HEY1	0.4630
HNF4A	0.4630
HRAS	0.4630
HSPD1	0.4630
IHH	0.4630
IL1A	0.4630
JUN	0.4630
KCNS3	0.4630
KLK1	0.4630
LAMB1	0.4630
LATS2	0.4630
LPAR1	0.4630
LYPLA1	0.4630
MEF2C	0.4630
MSI1	0.4630
MUC2	0.4630
MUC4	0.4630
NEU3	0.4630
NOTCH4	0.4630
OCLN	0.4630
PDE4D	0.4630
PLCG2	0.4630
PPARD	0.4630
PRKACB	0.4630
PRSS23	0.4630
PTGFRN	0.4630
PTPN2	0.4630
RBL1	0.4630

Gene	bresub error
RPS4X	0.4630
SDC1	0.4630
SERPINH1	0.4630
SLC39A8	0.4630
SLC3A2	0.4630
TP63	0.4630
TPT1	0.4630
TREH	0.4630
UHRF1	0.4630
VNN1	0.4630
WLS	0.4630
YWHAZ	0.4630
ZEB2	0.4630
ALOX15	0.4630
ALPI	0.4630
AMPD3	0.4630
APOC3	0.4630
AQP3	0.4630
ARRB2	0.4630
AXIN2	0.4630
BCL10	0.4630
BRAF	0.4630
CA2	0.4630
CACNB2	0.4630
CBX5	0.4630
CCL28	0.4630
CDCA7	0.4630
CDK15	0.4630
CDK7	0.4630
CDKN2A	0.4630
CDX1	0.4630
CEACAM1	0.4630
CEACAM5	0.4630
CEBPB	0.4630
CYB5R1	0.4630
CYBB	0.4630
CYP4F3	0.4630
DAPK3	0.4630

Gene	bresub error
DDIT4	0.4630
FDFT1	0.4630
FGF9	0.4630
FTH1	0.4630
GADD45A	0.4630
GSTA1	0.4630
HMGB1	0.4630
HNRNPA1	0.4630
HOXA9	0.4630
HOXC8	0.4630
IL22RA1	0.4630
IL2RB	0.4630
IL4R	0.4630
ILF3	0.4630
KDM5A	0.4630
LAMC2	0.4630
LCT	0.4630
LGALS3BP	0.4630
LGR4	0.4630
LTBP1	0.4630
MAML3	0.4630
MET	0.4630
MMP9	0.4630
MPC2	0.4630
MUC3A	0.4630
NAIP	0.4630
NOG	0.4630
NUPR1	0.4630
PDE4A	0.4630
PLIN2	0.4630
POLG	0.4630
PRKAR1A	0.4630
PROM2	0.4630
RBBP9	0.4630
SIGIRR	0.4630
SLC16A1	0.4630
SLC22A23	0.4630
SLC25A37	0.4630

Gene	bresub error
SLC44A3	0.4630
SLC51B	0.4630
SLC5A3	0.4630
SLC7A9	0.4630
SP1	0.4630
SQSTM1	0.4630
STK11	0.4630
TAT	0.4630
TAZ	0.4630
THBS1	0.4630
TNFAIP3	0.4630
TNFSF10	0.4630
TPM3	0.4630
TRIM31	0.4630

Gene	bresub error
TSC2	0.4630
ULK1	0.4630
VIL1	0.4630
WNT3	0.4630
ZBTB33	0.4630
HDAC2	0.4631
ICK	0.4631
INSR	0.4631
POR	0.4631
CDC25B	0.4631
SLC17A5	0.4632
SOX9	0.4632
TLR5	0.4632
GSS	0.4633

Gene	bresub error
SLC40A1	0.4634
SLC44A1	0.4634
MSH2	0.4634
BAD	0.4635
FGFR2	0.4635
ERBB2	0.4636
KLF6	0.4637
MEP1A	0.4638
PTPRD	0.4638
FSTL1	0.4639
HOXB5	0.4641
MACC1	0.4643
HAS3	0.4644
POLB	0.4645

Gene	bresub error
SLC35A3	0.4645
SLC6A19	0.4645
HIF3A	0.4645
HMGCS2	0.4645
MAB21L2	0.4645
MEN1	0.4645
SLC37A4	0.4646
SLC4A7	0.4646
MALL	0.4647
CEACAM19	0.4648
GNL1	0.4648
SLC28A2	0.4649
FLNB	0.4650
IGFBP3	0.4650

Table S6. Top 500 Two-Gene LDA Classifiers. The top 500 two-gene linear discriminant analysis classifiers and their respective misclassification rate in flaxseed lignan supplement vs placebo treatment in all samples regardless of their ENL phenotype.

Gene 1	Gene 2	bresub error
NIFK	PROX1	0.2140
KCTD12	TNK2	0.2157
BMP4	PROX1	0.2183
NR6A1	PROX1	0.2191
KCTD12	NOS2	0.2230
KCTD12	TNFRSF10D	0.2232
KCTD12	SLC7A7	0.2237
KCTD12	SLCO2A1	0.2249
KCTD12	TDG	0.2252
FOXD2	KCTD12	0.2258
KCTD12	PDE6D	0.2263
PRKCH	PROX1	0.2264
CHI3L1	NR6A1	0.2268
DDX11	PROX1	0.2273
KCTD12	PCNA	0.2283
MAML1	XRCC6	0.2290
MAML1	SPDEF	0.2314
CHI3L1	PROX1	0.2317
NR6A1	WNT5A	0.2318
TNK2	XRCC6	0.2319
NR6A1	PECAM1	0.2322
HES1	MYO9B	0.2323
KCTD12	MYO9B	0.2325
ID2	PROX1	0.2332
SPDEF	TNK2	0.2336
MAML1	SLC4A4	0.2336
KCTD12	MAML1	0.2338
KCTD12	NOTCH1	0.2340
KCTD12	SLC7A6	0.2344
BMP4	NR6A1	0.2346
HES1	MAML1	0.2351
CHI3L1	NIFK	0.2352
PRKCH	PECAM1	0.2356
PPAP2A	TDG	0.2360
ESR1	FOXD2	0.2361

Gene 1	Gene 2	bresub error
SLC4A4	TNK2	0.2363
CA14	TDG	0.2363
SLC5A11	TNFRSF10D	0.2367
CSPG5	TDG	0.2368
MAML1	SREBF2	0.2368
RASSF1	TDG	0.2369
HOXA10	TNK2	0.2370
DDR2	PROX1	0.2373
TBRG1	SLCO2A1	0.2374
MAPK7	SLCO2A1	0.2374
BMP4	PRKCH	0.2374
HOXA10	MAML1	0.2376
CASP3	KCTD12	0.2378
MYC	TDG	0.2379
NOTCH4	XRCC6	0.2379
RBL1	XRCC6	0.2379
MYO9B	SLC4A4	0.2382
ALOX15	XRCC6	0.2383
ADAM17	SLCO2A1	0.2385
SLC25A13	TDG	0.2386
CA14	FOXD2	0.2386
MAPK7	TDG	0.2387
MYO9B	XRCC6	0.2388
RBL1	TDG	0.2389
IL2RB	XRCC6	0.2390
NR6A1	TIMP3	0.2391
KCTD12	SPDEF	0.2391
CSPG5	XRCC6	0.2393
MYO9B	SPDEF	0.2394
SLC3A1	SLCO2A1	0.2394
MLH1	TDG	0.2395
TJP1	TNK2	0.2395
ESR1	TNFRSF10D	0.2395
BMP4	DDX11	0.2397
PCNA	SPDEF	0.2397

Gene 1	Gene 2	bresub error
MAML1	SHH	0.2398
PLCB2	SLCO2A1	0.2401
XIST	XRCC6	0.2404
TGM4	XRCC6	0.2404
TBRG1	TDG	0.2404
BCL2L1	XRCC6	0.2405
BMPR2	TDG	0.2405
GADD45B	PROX1	0.2406
TBRG1	FOXD2	0.2406
PRKCH	WNT5A	0.2406
PRKCI	TNK2	0.2407
ADAM17	TDG	0.2408
KCTD12	LIG3	0.2408
C11orf30	XRCC6	0.2409
TAT	XRCC6	0.2409
MAB21L2	XRCC6	0.2409
BMP4	REG1B	0.2410
SERPINA1	TNK2	0.2411
MYC	TNFRSF10D	0.2412
PPAP2A	TNFRSF10D	0.2413
PROX1	WNT5A	0.2414
NEU3	XRCC6	0.2414
ATF4	TNK2	0.2414
PPAP2A	PDE6D	0.2414
PPAP2A	SLCO2A1	0.2414
BCO2	XRCC6	0.2415
HOXC8	XRCC6	0.2416
SLC1A4	XRCC6	0.2416
SLC7A7	SPDEF	0.2419
ID3	XRCC6	0.2419
CA14	PDE6D	0.2420
MYC	FOXD2	0.2420
PRKCI	SLC1A4	0.2420
LAMB1	PRKCI	0.2422
IER3	XRCC6	0.2422
COL3A1	PRKCI	0.2423
DFFA	PROX1	0.2423
UHRF1	XRCC6	0.2423

Gene 1	Gene 2	bresub error
ITGAM	TDG	0.2423
NOTCH4	PRKCI	0.2424
DDX11	PECAM1	0.2424
ADAM17	TNFRSF10D	0.2426
NOS2	SLC4A4	0.2426
MAML1	PRKCI	0.2427
ADAM17	FOXD2	0.2427
HOXA10	MYO9B	0.2427
SPDEF	TDG	0.2428
TBRG1	PDE6D	0.2428
ATF4	MAML1	0.2428
SPDEF	TNFRSF10D	0.2429
DCN	PROX1	0.2430
MAML1	TJP1	0.2430
KCTD12	MAB21L2	0.2430
NIFK	NR6A1	0.2431
PRKCI	TSC2	0.2431
ALOX15	SLC4A4	0.2432
ADAM17	PDE6D	0.2432
NAIP	SLCO2A1	0.2433
WNT4	SLCO2A1	0.2433
PLCB2	FOXD2	0.2434
RASSF1	TNFRSF10D	0.2437
HOXA2	PROX1	0.2437
MAPK7	PDE6D	0.2438
MSH6	SLCO2A1	0.2438
HSPD1	SLC4A4	0.2438
SLCO2A1	SPDEF	0.2438
ID2	NR6A1	0.2439
NOG	XRCC6	0.2439
SLC3A1	TDG	0.2439
PTPN22	TDG	0.2439
CA14	SLCO2A1	0.2439
ELP3	IL18	0.2439
NEU3	PRKCI	0.2439
CA14	TNFRSF10D	0.2440
CSPG5	PRKCI	0.2440
CSPG5	SLC4A4	0.2441

Gene 1	Gene 2	bresub error
ESR1	PDE6D	0.2442
PRKCH	NR6A1	0.2443
MAPK7	TNFRSF10D	0.2443
SLC4A4	TAT	0.2443
IL2RB	PRKCI	0.2445
UHRF1	PRKCI	0.2445
TP63	SLC4A4	0.2446
RASSF1	FOXD2	0.2446
FASLG	PRKCI	0.2446
UHRF1	IL18	0.2447
CSPG5	ATF4	0.2447
SLC3A1	FOXD2	0.2447
PLCB2	TNFRSF10D	0.2448
NIFK	TRAFD1	0.2448
BMPR2	IL18	0.2449
NOG	SLC4A4	0.2450
GJD2	ATF4	0.2450
HAS2	XRCC6	0.2450
ITGAL	SLCO2A1	0.2450
RBL1	SLC4A4	0.2450
LDHB	XRCC6	0.2451
XIST	SLC4A4	0.2451
CYP4F3	XRCC6	0.2452
LPAR4	XRCC6	0.2452
FOXD2	MSH6	0.2453
IHH	SLC4A4	0.2453
GJD2	PRKCI	0.2454
PTPN22	TNK2	0.2455
PDX1	XRCC6	0.2455
PDE6D	SPDEF	0.2456
OGG1	SLCO2A1	0.2456
BMP4	NIFK	0.2456
SLC25A13	TNFRSF10D	0.2456
FOXD2	MAPK7	0.2458
CCDC80	SLCO2A1	0.2458
MLH1	PDE6D	0.2458
TBRG1	TNFRSF10D	0.2458
BCL2L1	SLC4A4	0.2459

Gene 1	Gene 2	bresub error
CDC25B	SLC4A4	0.2459
PROX1	REL	0.2460
COLEC12	TDG	0.2460
FPR1	XRCC6	0.2460
BMPR2	SLCO2A1	0.2461
PLD3	TDG	0.2461
UHRF1	ATF4	0.2461
SLC3A1	PDE6D	0.2461
NOS2	XRCC6	0.2461
DAPK3	SLC4A4	0.2461
MYC	PDE6D	0.2462
GABRB3	TNFRSF10D	0.2462
PLCB2	PDE6D	0.2462
WLS	SLC4A4	0.2463
NOTCH4	ATF4	0.2464
COLEC12	SLCO2A1	0.2464
MLH1	TNFRSF10D	0.2464
PLD3	SLCO2A1	0.2465
IL18	RBBP9	0.2466
COL3A1	RUNX2	0.2467
MAML1	SERPINA1	0.2467
MAP2K2	KCTD12	0.2468
SLC4A4	TNFRSF10D	0.2468
IL18	KCTD12	0.2469
BMP4	TRAFD1	0.2470
HMOX1	IL18	0.2470
GSS	PRKCI	0.2470
MELK	PRKCI	0.2471
NAIP	TTN	0.2471
CDK7	SLC4A4	0.2471
NOD2	SLCO2A1	0.2471
C11orf30	PRKCI	0.2472
MAML1	RUNX2	0.2472
PCNA	XRCC6	0.2473
PLCG2	SLC4A4	0.2473
MSH6	TDG	0.2474
PLCB2	TDG	0.2474
HAS2	HES1	0.2475

Gene 1	Gene 2	bresub error
PRKCI	TAT	0.2476
FGF9	SLC4A4	0.2477
MYO9B	TJP1	0.2477
HOXA13	XRCC6	0.2477
RELA	XRCC6	0.2478
PTPN2	SLC4A4	0.2478
HES1	NOS2	0.2478
MEN1	SLC4A4	0.2479
PLCG2	HES1	0.2480
CDC25B	XRCC6	0.2480
BTD	SLCO2A1	0.2481
IL18	SLC4A4	0.2481
ACADS	MAML1	0.2481
WNT4	FOXD2	0.2481
PECAM1	PROX1	0.2481
SLC6A19	XRCC6	0.2481
HES1	TAT	0.2482
ATF4	TSC2	0.2482
PROX1	PTK7	0.2482
COL3A1	XRCC6	0.2482
LAMB1	RUNX2	0.2482
HOXC4	XRCC6	0.2482
CYB5R1	SLC4A4	0.2484
FGF5	SLC4A4	0.2484
ATF4	TAT	0.2484
NEU3	ATF4	0.2485
ICAM1	SLC7A6	0.2485
HIF3A	SLC4A4	0.2485
LRIG1	TTN	0.2485
KCTD12	SORD	0.2486
CHI3L1	TIMP3	0.2486
ATF4	IL2RB	0.2486
CDCA7	PRKCI	0.2487
MAB21L2	SLCO2A1	0.2488
MAML1	TTN	0.2488
SIGIRR	TDG	0.2488
NOG	PRKCI	0.2489
TNFRSF1B	XRCC6	0.2490

Gene 1	Gene 2	bresub error
KCTD12	KIT	0.2492
CDK7	XRCC6	0.2492
PTPN22	TNFRSF10D	0.2492
HES1	HOXA13	0.2492
MEN1	XRCC6	0.2492
PLCB4	SLCO2A1	0.2492
NEU3	HES1	0.2493
ATF4	CAV1	0.2494
ABHD5	XRCC6	0.2494
FOXP4	TDG	0.2494
BIRC2	XRCC6	0.2494
CSPG5	SLCO2A1	0.2494
PPARG	SLC4A4	0.2494
GJB2	XRCC6	0.2495
FOXD2	ICAM1	0.2495
SPI1	XRCC6	0.2495
MELK	RUNX2	0.2496
HMOX1	SLCO2A1	0.2497
PPAP2B	PROX1	0.2498
RELA	SLC4A4	0.2499
MALL	RUNX2	0.2499
PCDH7	XRCC6	0.2499
TNFRSF10D	TSC2	0.2500
KCTD12	SLC28A2	0.2500
ALPI	HES1	0.2500
TBRG1	IL18	0.2500
ITGAL	FOXD2	0.2500
MSH6	PDE6D	0.2500
LDHB	PRKCI	0.2500
COL3A1	FOXD2	0.2501
CCL15	KCTD12	0.2501
CDX1	SLC4A4	0.2501
IL18	MSH6	0.2501
BTD	FOXD2	0.2501
NAIP	SLC4A4	0.2501
CYB5R1	PRKCI	0.2501
PTAFR	TNFRSF10D	0.2501
PDE6D	PTPN22	0.2502

Gene 1	Gene 2	bresub error
MSH3	SLCO2A1	0.2502
MYO9B	SERPINA1	0.2502
GJD2	XRCC6	0.2502
IGF2BP1	PROX1	0.2502
PDE4B	SLC4A4	0.2502
MAML1	TJP3	0.2502
POR	XRCC6	0.2503
NANOG	PROX1	0.2503
SLC3A1	TNFRSF10D	0.2503
LIG3	SPDEF	0.2503
PECAM1	TLR2	0.2503
CA3	SLCO2A1	0.2504
HES1	WNT3	0.2505
NEU3	SLC4A4	0.2505
PRKCH	CHI3L1	0.2505
ATF4	FASLG	0.2505
HES1	IL2RB	0.2505
SLC4A4	SLCO2A1	0.2506
PLS3	PROX1	0.2506
ATF4	SLC1A4	0.2506
HOXA10	SLC7A7	0.2506
CCND1	SLCO2A1	0.2506
ATF4	HOXA13	0.2506
CGNL1	SLCO2A1	0.2506
ELP3	SLCO2A1	0.2508
NOS2	SPDEF	0.2508
KLK6	IL18	0.2508
HES1	TAZ	0.2508
REST	SLC4A4	0.2508
COL3A1	IL18	0.2509
PECAM1	REG1B	0.2509
ATF4	DAPK3	0.2509
SLC3A1	IL18	0.2510
GCLM	XRCC6	0.2510
BTD	IL18	0.2510
SLC27A2	SLCO2A1	0.2511
NUPR1	XRCC6	0.2511
IL18	TAT	0.2511

Gene 1	Gene 2	bresub error
NOS2	PTPN22	0.2512
ID2	XRCC6	0.2512
HOXC4	PRKCI	0.2512
ITGAM	TNFRSF10D	0.2512
HOXA13	SLC4A4	0.2512
PRKACA	TDG	0.2512
RASSF1	SLCO2A1	0.2513
SLCO2A1	TSC2	0.2514
DDIT4	XRCC6	0.2514
PLA2G4B	SLC4A4	0.2514
MYC	SLCO2A1	0.2514
CA13	KCTD12	0.2515
SLC7A7	XRCC6	0.2515
NFKB2	TDG	0.2515
RUNX2	TLR5	0.2515
GJC1	XRCC6	0.2515
MSH2	SLC4A4	0.2515
SLC25A36	SLC4A4	0.2516
CCND1	TDG	0.2517
PRKACA	SLCO2A1	0.2517
CEACAM19	IL18	0.2519
GSS	XRCC6	0.2519
MSH6	TNFRSF10D	0.2519
BMP4	SLC39A5	0.2520
PTAFR	SLCO2A1	0.2521
SLC7A7	TJP1	0.2521
HES1	SLC1A4	0.2521
PECAM1	PTK7	0.2521
ATF4	LIG3	0.2522
NIFK	TIMP3	0.2522
PODXL	XRCC6	0.2522
PCNA	PRKCI	0.2522
HES1	RBBP9	0.2523
DAPK3	PRKCI	0.2524
PLA2G4B	XRCC6	0.2524
IER3	PRKCI	0.2524
HOXA2	PECAM1	0.2524
ADAM10	MAML1	0.2524

Gene 1	Gene 2	bresub error
CDKN2A	SLC4A4	0.2524
MALL	PRKCI	0.2524
GJC1	KCTD12	0.2524
MACC1	XRCC6	0.2524
ERBB4	SLC4A4	0.2525
SIGIRR	XRCC6	0.2525
LIG3	XRCC6	0.2525
ISL1	NR6A1	0.2525
FSTL1	SLC4A4	0.2525
NOD2	IL18	0.2525
ALOX15	PRKCI	0.2525
DEFA5	SLC4A4	0.2525
ATF4	NOG	0.2525
HOXB5	SLC4A4	0.2526
DPEP1	XRCC6	0.2526
TNFRSF10D	XRCC6	0.2527
PPARG	XRCC6	0.2527
MAPK9	XRCC6	0.2527
UHRF1	RUNX2	0.2527
CAV1	PRKCI	0.2528
CSPG5	IL18	0.2528
RUNX2	SLC1A4	0.2528
FFAR4	KCTD12	0.2528
LIG3	SREBF2	0.2529
FOXD2	PTPN22	0.2530
NOTCH3	PROX1	0.2530
SLC4A4	SLC7A7	0.2530
CEACAM3	XRCC6	0.2530
ORMDL3	XRCC6	0.2531
PLCB2	IL18	0.2531
SLC34A2	TNFRSF10D	0.2531
EGFR	SLC4A4	0.2532
GSTM3	TDG	0.2532
ID2	SLC4A4	0.2532
NTHL1	XRCC6	0.2532
CCDC80	IL18	0.2533
HES1	PCNA	0.2533
IL2RB	SLC4A4	0.2533

Gene 1	Gene 2	bresub error
SORD	XRCC6	0.2533
FPR1	SLC4A4	0.2533
ICAM1	TDG	0.2533
HOXA10	HOXA13	0.2534
PRKCI	SORD	0.2534
DCN	XRCC6	0.2534
PTPN2	XRCC6	0.2535
PTPN22	SLCO2A1	0.2535
KCTD12	ZDHHC20	0.2535
HOXA13	PRKCI	0.2535
POR	PRKCI	0.2535
MYO9B	SREBF2	0.2535
CCDC80	FOXD2	0.2536
MUC19	SLCO2A1	0.2536
SLC39A5	PECAM1	0.2536
RUNX2	TSC2	0.2536
MALL	TTN	0.2536
LIN28A	XRCC6	0.2536
IL18	SERPINA1	0.2537
PIK3R1	XRCC6	0.2537
KCTD12	SLC38A2	0.2537
PCNA	SHH	0.2537
DEFA5	XRCC6	0.2538
WNT4	PDE6D	0.2538
SLC25A36	XRCC6	0.2538
RAD51D	FOXD2	0.2538
FAM129A	PROX1	0.2539
ADRB1	PRKCI	0.2539
CDKN2A	XRCC6	0.2540
ATF4	MAB21L2	0.2540
CDX1	XRCC6	0.2540
SLC5A1	XRCC6	0.2540
ABCC1	XRCC6	0.2540
HOXA5	KCTD12	0.2541
HES1	RELA	0.2542
NOD2	FOXD2	0.2542
SLC7A6	SPDEF	0.2542
NOTCH4	SLC4A4	0.2543

Gene 1	Gene 2	bresub error
DHFR	FOXD2	0.2543
IL18	MALL	0.2543
SLC1A4	SREBF2	0.2543
ALOX15	HOXA10	0.2544
SLC5A9	TDG	0.2544
GJB2	SLC4A4	0.2544
CAMK2B	SLC4A4	0.2544
CCL15	XRCC6	0.2544
HEY1	FOXD2	0.2544
CDH5	XRCC6	0.2544
PARD3	PROX1	0.2545
IL18	TLR5	0.2545
PLCB2	SLC7A6	0.2546
MAP2K2	XRCC6	0.2546
CEACAM19	SLCO2A1	0.2546
SLC4A4	TFRC	0.2547
CYB5R1	HES1	0.2547
PLCB4	FOXD2	0.2547
FOXD2	OGG1	0.2547
CDH5	RUNX2	0.2547
HOXA13	SREBF2	0.2547
PRKCI	WNT3	0.2548
NIFK	PECAM1	0.2548
SLC35A1	HES1	0.2549
DHFR	SLCO2A1	0.2549

Gene 1	Gene 2	bresub error
COLEC12	TNFRSF10D	0.2550
OGG1	TDG	0.2551
GNL1	HES1	0.2551
BMPR2	FOXD2	0.2551
HSPD1	PRKCI	0.2551
CEACAM19	RUNX2	0.2551
POR	RUNX2	0.2551
BAMBI	XRCC6	0.2552
SST	SLCO2A1	0.2552
SLC6A19	SLC4A4	0.2553
SLC39A8	ATF4	0.2553
ELP3	TDG	0.2553
ICAM1	PDE6D	0.2553
HMOX1	TDG	0.2553
RBBP9	SLC4A4	0.2553
GCLM	SLC4A4	0.2553
PRKCH	NIFK	0.2553
IL1R2	XRCC6	0.2553
BCL2L13	KCTD12	0.2553
MELK	SLC7A6	0.2554
REG1A	XRCC6	0.2555
ITGAL	PDE6D	0.2555
ELP3	SREBF2	0.2555
CSPG5	TNFRSF10D	0.2555
GSS	RUNX2	0.2556

Table S7. Top 500 Three-Gene LDA Classifiers. The top 500 three-gene linear discriminant analysis classifiers and their respective misclassification rate in flaxseed lignan supplement vs placebo treatment in all samples regardless of their ENL phenotype.

Gene 1	Gene 2	Gene 3	bresub error	Gene 1	Gene 2	Gene 3	bresub error
PCDH7	KCTD12	SPDEF	0.2014	PRKCH	SLC39A5	WNT5A	0.2115
EPHB6	KCTD12	SPDEF	0.2015	BMP4	PROX1	WNT5A	0.2121
KCTD12	MAML1	SPDEF	0.2021	CHI3L1	NR6A1	PROX1	0.2121
BMP4	FGFR1	WNT5A	0.2024	EP400	KCTD12	SPDEF	0.2122
KCTD12	NOTCH1	SPDEF	0.2027	BMP4	IER3	WNT5A	0.2123
KCTD12	SPDEF	TNK2	0.2031	BMP4	NR6A1	REL	0.2124
HOXA13	KCTD12	SPDEF	0.2034	KCTD12	MGAT3	SPDEF	0.2124
KCTD12	SLC7A7	SPDEF	0.2036	PRKCH	SLC39A5	PROX1	0.2126
BMP4	NR6A1	WNT5A	0.2052	KCTD12	MYO9B	SPDEF	0.2127
KCTD12	SORD	SPDEF	0.2059	DDX11	PROX1	ZNF365	0.2128
KCTD12	LIG3	SPDEF	0.2067	SLC39A5	NR6A1	PROX1	0.2130
GJC1	KCTD12	SPDEF	0.2071	BMP4	SLC39A5	WNT5A	0.2130
KCTD12	SLC13A2	SPDEF	0.2076	KEAP1	NIFK	PROX1	0.2132
AXIN1	KCTD12	SPDEF	0.2081	BMP4	REL	WNT5A	0.2132
XIST	KCTD12	SPDEF	0.2083	NIFK	PPAP2B	PROX1	0.2135
BMP4	TLR2	WNT5A	0.2085	CCL15	KCTD12	SPDEF	0.2135
KCTD12	SLC28A1	SPDEF	0.2086	KCTD12	NOTCH1	TNK2	0.2138
KCTD12	KIT	SPDEF	0.2087	IL36RN	NIFK	PROX1	0.2139
KCTD12	PCNA	SPDEF	0.2089	PRKCH	DDX11	PROX1	0.2139
KCTD12	MAB21L2	SPDEF	0.2089	BMP4	PROX1	REL	0.2139
DDX11	IER3	PROX1	0.2094	KCTD12	LIG3	TNK2	0.2140
ACSL4	KCTD12	SPDEF	0.2099	HOXA9	KCTD12	TNK2	0.2141
BMP4	REG1B	WNT5A	0.2102	BMP4	NOS1	WNT5A	0.2141
KCTD12	SLC2A5	SPDEF	0.2103	SLC10A2	KCTD12	SPDEF	0.2141
IL1R2	DDX11	PROX1	0.2107	DDX11	PROX1	REG1B	0.2142
BMP4	PRKCH	WNT5A	0.2108	TGM2	NIFK	PROX1	0.2143
CHI3L1	NIFK	PROX1	0.2109	BMP4	PPAP2B	WNT5A	0.2143
BMP4	NFKB2	NR6A1	0.2110	PRKCH	PROX1	PTK7	0.2143
BMP4	PRNP	WNT5A	0.2112	HES1	IL18	MAML1	0.2144
BMP4	DDX11	WNT5A	0.2113	BMP4	WNT5A	ZNF365	0.2144
KCTD12	PTPN22	SPDEF	0.2113	BMP4	NFKB2	PROX1	0.2145
CA13	KCTD12	SPDEF	0.2114	DDX11	ID2	PROX1	0.2145
BMP4	TLR3	WNT5A	0.2115	NR6A1	PPAP2B	PROX1	0.2145
PRKCH	CHI3L1	PROX1	0.2115	BMP4	NR6A1	PROX1	0.2145

Gene 1	Gene 2	Gene 3	bresub error	Gene 1	Gene 2	Gene 3	bresub error
KCTD12	SLC13A2	TNK2	0.2145	NR6A1	PROX1	PTPN11	0.2160
IL18	MAML1	SREBF2	0.2145	ADAM17	KCTD12	TNK2	0.2160
PROX1	PTK7	REG1B	0.2145	BMP4	PROX1	PTK7	0.2160
DDX11	NIFK	PROX1	0.2145	AKTIP	NIFK	PROX1	0.2161
SLC39A5	DDX11	PROX1	0.2145	CSPG5	KCTD12	TNK2	0.2161
PLCD1	NIFK	PROX1	0.2145	KCTD12	SLC7A11	SPDEF	0.2161
NPAT	KCTD12	SPDEF	0.2146	BMP4	DDX11	PROX1	0.2161
BMP4	TGM2	WNT5A	0.2147	BMP4	TGM2	PROX1	0.2162
BMP4	NR6A1	PTK7	0.2147	LPAR4	KCTD12	SPDEF	0.2162
KCTD12	PECAM1	TNFRSF10D	0.2149	HES1	IL18	MYO9B	0.2162
NIFK	PROX1	TNFRSF10A	0.2150	CHI3L1	PROX1	TIMP3	0.2163
SLC39A5	NIFK	PROX1	0.2150	NIFK	PROX1	REG1B	0.2164
NIFK	PROX1	PTK7	0.2151	DCN	NIFK	PROX1	0.2164
CA14	KCTD12	TNK2	0.2151	KCTD12	PSME2	SPDEF	0.2165
BMP4	IL1R2	WNT5A	0.2151	HOXA9	KCTD12	SPDEF	0.2165
RBL1	KCTD12	TNK2	0.2152	MYC	KCTD12	TNK2	0.2165
NIFK	NR6A1	PROX1	0.2152	HOXA10	IL18	MAML1	0.2166
HEY1	KCTD12	TNK2	0.2152	PRKCH	NIFK	PROX1	0.2166
NFKB2	NR6A1	PROX1	0.2152	ITGB1	KCTD12	SPDEF	0.2166
GADD45B	PRKCH	PROX1	0.2153	RAD51D	KCTD12	TNK2	0.2166
NIFK	PROX1	TIMP3	0.2153	PRKCH	NR6A1	PROX1	0.2166
DDX11	PROX1	TIMP3	0.2153	CDH3	NIFK	PROX1	0.2167
KEAP1	NR6A1	PROX1	0.2154	CHI3L1	DDX11	PROX1	0.2168
IER3	NIFK	PROX1	0.2154	KCTD12	NOS2	SPDEF	0.2168
HOXA2	DDX11	PROX1	0.2154	KCTD12	MAB21L2	TNK2	0.2168
KCTD12	LRIG1	SPDEF	0.2154	TGFB2	KCTD12	TNK2	0.2168
NR6A1	PROX1	WNT5A	0.2154	BMP4	DOT1L	PROX1	0.2168
NR6A1	PROX1	TLR3	0.2154	HOXD13	KCTD12	TNK2	0.2169
BMP4	SLC5A9	PROX1	0.2155	PRKCH	SLC6A14	PROX1	0.2169
PLS3	NIFK	PROX1	0.2155	KCTD12	MSH6	TNK2	0.2169
BMP4	PECAM1	WNT5A	0.2156	KCTD12	SPDEF	TNFRSF10D	0.2169
PRKCH	SLC39A5	PTK7	0.2157	DCN	DDX11	PROX1	0.2170
KCTD12	PTPN22	SLC7A7	0.2158	KDM4A	KCTD12	SPDEF	0.2170
COL1A1	NIFK	PROX1	0.2158	KCTD12	TAT	TNK2	0.2170
NIFK	PROX1	PTPN11	0.2159	FGFR3	KCTD12	SPDEF	0.2170
TLR4	KCTD12	TNK2	0.2159	KCTD12	TAZ	TNK2	0.2170
KCTD12	PTPN22	TNK2	0.2159	ADAMTS4	NIFK	PROX1	0.2170

Gene 1	Gene 2	Gene 3	bresub error	Gene 1	Gene 2	Gene 3	bresub error
NR6A1	PROX1	TIMP3	0.2170	KCTD12	MAML1	TNFRSF10D	0.2179
KCNH2	NIFK	PROX1	0.2170	NIFK	PROX1	SLC13A1	0.2179
GADD45B	NR6A1	PROX1	0.2171	BMP4	MAPK7	PROX1	0.2179
IL1R2	NIFK	PROX1	0.2172	ISL1	KCTD12	TNK2	0.2179
GABRB3	KCTD12	TNK2	0.2172	CHI3L1	ID2	PROX1	0.2179
NR6A1	PROX1	SLC13A1	0.2172	BMP4	SST	WNT5A	0.2180
TBRG1	KCTD12	TNK2	0.2172	IL1R2	CHI3L1	PROX1	0.2180
PRKCH	NR6A1	REG1B	0.2172	CHI3L1	PROX1	ZNF365	0.2180
BCL2L14	NIFK	PROX1	0.2172	BMP4	PRKCH	PROX1	0.2180
BMP4	CEACAM3	WNT5A	0.2173	KCTD12	TNK2	TSC2	0.2180
KCTD12	MLH1	TNK2	0.2173	MAPK9	PROX1	TLR2	0.2180
BMP4	DOT1L	WNT5A	0.2173	TGFB2	KCTD12	SPDEF	0.2181
CHI3L1	DCN	PROX1	0.2173	ID2	NIFK	PROX1	0.2181
KCTD12	MAML1	NOS2	0.2174	NIFK	PROX1	ZNF365	0.2182
KCTD12	SPDEF	THRA	0.2174	COL1A2	NIFK	PROX1	0.2182
LPAR1	KCTD12	TNK2	0.2174	APEX1	NIFK	PROX1	0.2182
NIFK	PLA2G4D	PROX1	0.2174	CFTR	NIFK	PROX1	0.2183
HAS2	KCTD12	SPDEF	0.2174	GADD45B	NIFK	PROX1	0.2183
SLC39A5	NR6A1	WNT5A	0.2175	IL1R2	PROX1	PTK7	0.2183
CHI3L1	KEAP1	PROX1	0.2175	PLAT	PRKCH	PROX1	0.2183
CAV1	PROX1	REL	0.2175	PRKCH	PROX1	SLC13A1	0.2183
DAB2IP	NR6A1	PROX1	0.2176	MAPK9	NR6A1	PROX1	0.2183
PLS3	NR6A1	PROX1	0.2176	HOXA13	MAML1	SPDEF	0.2184
SLC5A11	KCTD12	TNK2	0.2176	MAP2K2	KCTD12	SPDEF	0.2184
BMP4	PROX1	TLR2	0.2176	BMP4	PTK7	WNT5A	0.2185
SLC39A5	PROX1	PTK7	0.2176	KCTD12	NAIP	TNK2	0.2185
BMP4	COLEC12	WNT5A	0.2177	ABCG5	NR6A1	PROX1	0.2185
BCL2L14	DDX11	PROX1	0.2177	GADD45B	CHI3L1	PROX1	0.2185
PRKCH	GCM1	PROX1	0.2177	BMP4	PROX1	REG1B	0.2185
FFAR4	KCTD12	TNK2	0.2177	DDX11	KEAP1	PROX1	0.2185
BMP4	ID2	WNT5A	0.2177	PLAT	NR6A1	PROX1	0.2185
PRKCH	CHI3L1	NR6A1	0.2178	ADAMTS4	CHI3L1	PROX1	0.2185
HOXB5	NIFK	PROX1	0.2178	PRKCH	APEX1	PROX1	0.2185
GSTM3	KCTD12	SPDEF	0.2178	PLS3	DDX11	PROX1	0.2185
ELP3	KCTD12	TNK2	0.2178	ABCC1	NIFK	PROX1	0.2187
NIFK	PARD3	PROX1	0.2178	MELK	KCTD12	TNK2	0.2187
ABCG5	NIFK	PROX1	0.2178	CHI3L1	PROX1	REG1B	0.2187

Gene 1	Gene 2	Gene 3	bresub error	Gene 1	Gene 2	Gene 3	bresub error
IL1R2	PROX1	TIMP3	0.2187	CDKN1C	DDX11	PROX1	0.2193
MAML1	PTPN22	SPDEF	0.2187	PRKCH	PLA2G4D	PROX1	0.2193
CDKN1C	NIFK	PROX1	0.2187	CDKN1C	CHI3L1	PROX1	0.2193
ABCC1	CHI3L1	PROX1	0.2187	HAS2	KCTD12	TNK2	0.2194
MAML1	SHH	SREBF2	0.2188	SLC39A5	PROX1	TIMP3	0.2194
NR6A1	PROX1	PTK7	0.2188	COL3A1	KCTD12	TNK2	0.2195
ID2	PROX1	PTK7	0.2188	ADAM10	IL18	MAML1	0.2195
KCTD12	SLC28A2	SPDEF	0.2188	CDKN1C	NR6A1	PROX1	0.2195
PPAP2A	KCTD12	TNK2	0.2188	PRKAR1B	KCTD12	TNK2	0.2195
CEACAM3	NIFK	PROX1	0.2188	PLS3	PRKCH	PROX1	0.2195
PTAFR	KCTD12	TNK2	0.2189	CEACAM3	CHI3L1	PROX1	0.2195
PLCB2	KCTD12	TNK2	0.2189	IL18	MAML1	TJP1	0.2195
SLC6A14	NIFK	PROX1	0.2189	DPYSL3	KCTD12	TNK2	0.2195
DDX11	PROX1	PTK7	0.2189	DBN1	KCTD12	SPDEF	0.2196
BMP4	NR6A1	REG1B	0.2189	PRKCH	DCN	PROX1	0.2196
KCTD12	TNFRSF10D	TNK2	0.2189	PROX1	PTK7	TIMP3	0.2196
ELP3	IL18	MAML1	0.2189	PRKCH	COL1A2	PROX1	0.2196
DDX11	PROX1	PTPN11	0.2189	RIPK1	NIFK	PROX1	0.2196
BMP4	PLA2G7	PROX1	0.2189	FGF9	KCTD12	SPDEF	0.2196
PRKCH	SLC39A5	DDX11	0.2190	HES1	IL18	SERPINA1	0.2197
BMP4	RBP4	WNT5A	0.2190	HOXD10	KCTD12	TNK2	0.2197
BMP4	RBP4	PROX1	0.2190	BCL2L14	CHI3L1	PROX1	0.2197
ICAM1	KCTD12	TNK2	0.2190	PRKCH	ABCG5	PROX1	0.2197
CEACAM3	DDX11	PROX1	0.2190	HRH1	NIFK	PROX1	0.2198
BMP4	HOXC6	PROX1	0.2190	BMP4	TBRG1	PROX1	0.2198
HOXA13	KCTD12	NOS2	0.2191	BMP4	HOXA2	WNT5A	0.2198
NR6A1	PRKCB	PROX1	0.2191	GJD2	NIFK	PROX1	0.2198
BMP4	IL1R2	PROX1	0.2192	NIFK	PROX1	WNT3	0.2198
CHI3L1	PARD3	PROX1	0.2192	BMP4	NR6A1	TLR2	0.2199
COL1A1	NR6A1	PROX1	0.2192	GJC1	KCTD12	TNK2	0.2199
HOXA10	MAML1	TDG	0.2192	BMP4	BRIX1	WNT5A	0.2199
GCM1	NR6A1	PROX1	0.2192	DCN	ID2	PROX1	0.2200
HOXB6	KCTD12	TNK2	0.2192	MAML1	TDG	XRCC6	0.2200
PRKCH	CHI3L1	DDX11	0.2192	BMP4	HOXD13	PROX1	0.2200
DCN	NR6A1	PROX1	0.2192	CHI3L1	NR6A1	TIMP3	0.2200
FFAR4	KCTD12	SLC7A7	0.2192	IL18	SERPINA1	TNK2	0.2200
NR6A1	PLA2G4D	PROX1	0.2193	NOTCH4	KCTD12	TNK2	0.2201

Gene 1	Gene 2	Gene 3	bresub error	Gene 1	Gene 2	Gene 3	bresub error
NFKB2	PRKCH	PROX1	0.2201	FGFR1	TLR2	WNT5A	0.2206
ITGB1	KCTD12	TNK2	0.2201	IER3	NR6A1	PROX1	0.2206
HOXA2	CHI3L1	PROX1	0.2201	KCTD12	LIG3	TNFRSF10D	0.2206
BMP4	SLC5A9	NR6A1	0.2201	NR6A1	PECAM1	TLR2	0.2207
IL18	MAML1	SHH	0.2201	KCTD12	NOTCH1	TNFRSF10D	0.2207
KEAP1	PROX1	TIMP3	0.2201	PLS3	CHI3L1	PROX1	0.2207
FAM129A	PROX1	TLR3	0.2202	SLC25A36	KCTD12	SPDEF	0.2208
EPHB6	KCTD12	SLC7A7	0.2202	SCNN1B	NIFK	PROX1	0.2208
BMP4	PRKCH	PTK7	0.2202	PRKCH	SCNN1B	PROX1	0.2208
DDX11	NR6A1	PROX1	0.2202	BCO2	KCTD12	SPDEF	0.2208
PRKCH	ADAMTS4	CHI3L1	0.2202	ICK	KCTD12	SPDEF	0.2208
PRKCH	PROX1	PTPN11	0.2202	IL18	MAML1	TTN	0.2208
BMP4	NFKB2	NOS1	0.2202	BMP4	PRNP	PROX1	0.2208
PRKCH	KEAP1	PROX1	0.2203	NR6A1	REG1B	WNT5A	0.2209
HES1	MAML1	TDG	0.2203	BMP4	PTK7	REG1B	0.2209
KCTD12	PECAM1	PTPN22	0.2203	IHH	KCTD12	TNK2	0.2209
COL1A2	NR6A1	PROX1	0.2203	HOXA13	KCTD12	TNFRSF10D	0.2209
KCTD12	MMP1	TNK2	0.2203	ESR1	KCTD12	TNFRSF10D	0.2209
BMP4	NR6A1	PECAM1	0.2203	IL18	SLC4A4	TNK2	0.2210
CHI3L1	PROX1	PTPN11	0.2204	KCTD12	PTPN6	SPDEF	0.2210
KCTD12	PTPN22	TNFRSF10D	0.2204	PTGFRN	NIFK	PROX1	0.2210
KCTD12	PLA2G7	TNK2	0.2204	KCTD12	TNFRSF10D	TSC2	0.2210
CDK2AP1	NIFK	PROX1	0.2204	HES1	MYO9B	TDG	0.2210
KCTD12	TFAP2C	TNK2	0.2204	HEY1	KCTD12	TNFRSF10D	0.2210
TGM2	CHI3L1	PROX1	0.2204	AKTIP	NR6A1	PROX1	0.2210
PRKCH	ADAMTS4	PROX1	0.2204	BMP4	NFKB2	PRKCH	0.2210
KCTD12	NOS2	TNK2	0.2204	RBP4	KCTD12	SPDEF	0.2210
BMP4	RASSF1	PROX1	0.2204	BMP4	NR6A1	TLR3	0.2211
BMP4	TBRG1	NR6A1	0.2204	BMP4	ITGB2	WNT5A	0.2211
IL1R2	NR6A1	PROX1	0.2204	ANXA3	CASP1	PTGES	0.2211
TGM2	DCN	PROX1	0.2205	PRKCH	ABCG5	NR6A1	0.2211
CHI3L1	PROX1	TFAP2A	0.2205	KCTD12	SLC23A3	SPDEF	0.2211
CA5B	NR6A1	PROX1	0.2205	PRKCH	NFKB1	PROX1	0.2211
IL18	MAML1	SLC4A4	0.2205	KCTD12	SLC7A7	TNFRSF10A	0.2211
ELP3	MAML1	SPDEF	0.2205	ID2	NR6A1	PROX1	0.2212
HOXC6	KCTD12	TNFRSF10D	0.2205	CDK8	MAML1	XRCC6	0.2212
NR6A1	PROX1	ZNF365	0.2206	IL18	MAML1	SERPINA1	0.2212

Gene 1	Gene 2	Gene 3	bresub error	Gene 1	Gene 2	Gene 3	bresub error
SLC39A5	TGM2	WNT5A	0.2212	PROX1	TIMP3	ZNF365	0.2218
HAS2	NIFK	PROX1	0.2213	IL18	TDG	TLR2	0.2218
CHI3L1	PPAP2B	PROX1	0.2213	PRKCH	SLC5A9	PROX1	0.2218
CA14	KCTD12	TNFRSF10D	0.2213	NIFK	PROX1	TFAP2A	0.2218
IL18	MAML1	XRCC6	0.2213	BMP4	NRCAM	PROX1	0.2218
HOXA10	IL18	TNK2	0.2213	IER3	PROX1	TIMP3	0.2218
IKBKB	NIFK	PROX1	0.2214	ICAM1	KCTD12	TNFRSF10D	0.2218
BMP4	NANOG	WNT5A	0.2214	KCTD12	TLR5	TNK2	0.2218
AKTIP	CHI3L1	PROX1	0.2214	NIFK	PROX1	SLC30A6	0.2219
MAML1	SREBF2	TJP3	0.2214	BMP4	FGF18	WNT5A	0.2219
CHI3L1	NIFK	NR6A1	0.2214	PCDH7	KCTD12	TNK2	0.2219
CDCA7	NIFK	PROX1	0.2214	BMP4	TRAFD1	WNT5A	0.2219
XIST	KCTD12	TNK2	0.2214	TDG	TNK2	XRCC6	0.2219
IL18	MAML1	TJP3	0.2214	UHRF1	KCTD12	TNK2	0.2219
HOXC8	KCTD12	SPDEF	0.2214	ITGAM	KCTD12	TNK2	0.2219
NRCAM	KCTD12	TNK2	0.2215	GSTM3	KCTD12	TNFRSF10D	0.2219
NR6A1	PROX1	REG1B	0.2215	CA3	KCTD12	SPDEF	0.2219
BMP4	PROX1	ZNF365	0.2215	PRKCH	DDR2	PROX1	0.2219
GABRB3	KCTD12	TNFRSF10D	0.2215	KCTD12	SLC7A7	TNFRSF10D	0.2219
HOXA13	KCTD12	TNK2	0.2215	FOXP4	KCTD12	TNK2	0.2220
KCTD12	TNK2	WNT3	0.2215	KCTD12	RBL2	SPDEF	0.2220
NIFK	OGG1	PROX1	0.2215	HES1	MYO9B	SREBF2	0.2220
KCTD12	NOS2	PTPN22	0.2216	KCTD12	NOTCH3	TNK2	0.2220
HOXD10	KCTD12	TNFRSF10D	0.2216	SLC39A5	CHI3L1	PROX1	0.2221
ALPI	KCTD12	SPDEF	0.2216	CEACAM3	NR6A1	PROX1	0.2221
GCM1	NIFK	PROX1	0.2216	HES1	IL18	PCNA	0.2221
SLC3A1	KCTD12	TNK2	0.2216	PRNP	IL18	TDG	0.2221
ELP3	KCTD12	TNFRSF10D	0.2216	DDR2	NIFK	PROX1	0.2221
BMP4	CFTR	WNT5A	0.2216	RBL1	KCTD12	TNFRSF10D	0.2221
BMP4	FN1	WNT5A	0.2216	HES1	HOXA13	IL18	0.2221
HOXA2	NIFK	PROX1	0.2216	BMP4	PROX1	SLC25A13	0.2221
KCTD12	SLC2A5	TNFRSF10D	0.2216	GSTM3	IL18	TDG	0.2221
EPHB6	KCTD12	TNFRSF10D	0.2217	HOXA13	MAML1	SREBF2	0.2221
PRKCH	SST	PROX1	0.2217	NIFK	PROX1	SLC2A2	0.2221
HOXD13	KCTD12	TNFRSF10D	0.2217	IER3	PROX1	PTK7	0.2221
CYP4F3	NIFK	PROX1	0.2218	LPAR1	KCTD12	TNFRSF10D	0.2221
COL1A2	ID2	PROX1	0.2218	DDX11	IL18	TDG	0.2221

Gene 1	Gene 2	Gene 3	bresub error	Gene 1	Gene 2	Gene 3	bresub error
SLC4A4	TDG	TNK2	0.2222	CHI3L1	PROX1	TNFRSF10A	0.2224
CHGA	KCTD12	TNFRSF10D	0.2222	KCTD12	MAML1	PDE6D	0.2224
MAML1	SREBF2	XRCC6	0.2222	CHI3L1	NIFK	PTGES	0.2224
KCTD12	MLH1	TNFRSF10D	0.2222	NFKB2	NIFK	PROX1	0.2224
RAD51D	KCTD12	TNFRSF10D	0.2222	ELP3	KCTD12	SLC7A7	0.2224
MELK	KCTD12	TNFRSF10D	0.2222	KCTD12	MSH6	TNFRSF10D	0.2224
CDH5	KCTD12	SPDEF	0.2222	KCTD12	NAIP	TNFRSF10D	0.2225
PRKCH	AKTIP	PROX1	0.2222	PRKCH	PROX1	TFAP2A	0.2225
DAPK2	KCTD12	SPDEF	0.2222	IL18	NR6A1	TDG	0.2225
TLR4	KCTD12	TNFRSF10D	0.2222	UHRF1	KCTD12	TNFRSF10D	0.2225
ABCG5	CHI3L1	PROX1	0.2222	MYC	KCTD12	TNFRSF10D	0.2225
CYP4F3	NR6A1	PROX1	0.2222	NR6A1	PROX1	SLC2A2	0.2225
KCTD12	SLC13A2	TNFRSF10D	0.2222	CDK7	DDX11	PROX1	0.2225
MAML1	SREBF2	TTN	0.2222	NR6A1	TLR2	WNT5A	0.2225
PROX1	PTPN11	TIMP3	0.2222	TLR4	KCTD12	SLC7A7	0.2226
NIFK	PROX1	TLR3	0.2222	NFKB2	NR6A1	WNT5A	0.2226
IL18	MAML1	PRKCI	0.2223	PRNP	NIFK	PROX1	0.2226
CA13	KCTD12	TNK2	0.2223	ELP3	IL18	TDG	0.2226
ADAM17	KCTD12	TNFRSF10D	0.2223	KCTD12	NR5A2	SPDEF	0.2226
NIFK	PROX1	WNT5A	0.2223	GJC1	KCTD12	TNFRSF10D	0.2226
PPAP2A	KCTD12	TNFRSF10D	0.2223	HOXA2	CHI3L1	NR6A1	0.2226
SLC5A9	NIFK	PROX1	0.2223	PRKCH	DDX11	ID2	0.2226
RELB	NR6A1	PROX1	0.2223	HOXD10	KCTD12	SLC7A7	0.2226
HOXD13	KCTD12	NOS2	0.2223	NR6A1	PECAM1	REL	0.2227
NIFK	NOG	PROX1	0.2223	SST	NIFK	PROX1	0.2227
MAML1	SLC4A4	TDG	0.2223	RBL1	KCTD12	NOS2	0.2227
ALOX15	KCTD12	TNK2	0.2223	SLC6A20	NR6A1	PROX1	0.2227
LAMB1	KCTD12	TNK2	0.2224	BMP4	PRKCH	DDX11	0.2227
HOXD10	KCTD12	NOS2	0.2224	CHI3L1	PROX1	PTK7	0.2227
KCTD12	MAB21L2	TNFRSF10D	0.2224	SLC5A11	KCTD12	TNFRSF10D	0.2227
HES1	MYO9B	SLCO2A1	0.2224	CDKN1C	ID2	PROX1	0.2227

Table S8. Top 500 high-ENL Single-Gene LDA Classifiers. The top 500 single-gene linear discriminant analysis classifiers and their respective misclassification rate in flaxseed lignan supplement vs placebo treatment in high-ENL excreters.

Gene	bresub error	Gene	bresub error	Gene	bresub error	Gene	bresub error
ANXA3	0.2454	PLA2G4C	0.2966	FOXP2	0.3194	SAT2	0.3333
NR6A1	0.2498	CHI3L1	0.2968	PKM	0.3201	TTN	0.3334
KCTD12	0.2521	FOXO3	0.2975	RUNX2	0.3203	RAD52	0.3335
EPHB1	0.2557	SLC3A1	0.2984	FOXK2	0.3204	REG1B	0.3341
PRKCH	0.2573	HES1	0.2992	ZNF365	0.3209	SLC17A5	0.3342
GCM1	0.2597	HMGCS1	0.3021	ADAMTS4	0.3222	ID2	0.3345
PTGIR	0.2626	IL1R2	0.304	SLC28A1	0.3224	ANXA5	0.3347
PROX1	0.2662	SLC5A8	0.3041	SORD	0.3225	TIMP3	0.3347
DLL1	0.2681	PCNA	0.3044	TRAFD1	0.3231	SHH	0.3352
PAX6	0.2687	IL18	0.3079	SLC35A2	0.3232	SOX4	0.3353
PARD3	0.2719	CDCP1	0.3086	HSD17B2	0.3234	CAT	0.3355
F11R	0.2732	SLC20A1	0.3093	PDE6D	0.324	BMI1	0.3357
PPAP2C	0.2732	PRKAR1A	0.3097	WNT5A	0.3244	REL	0.3359
ELP3	0.2801	HDAC1	0.3115	HOXD8	0.3247	GCLC	0.3375
NIFK	0.2802	TNFRSF10A	0.3116	TNF	0.3257	DDX5	0.339
RHOB	0.2808	TFAP2A	0.3117	HOXB8	0.3262	CCDC80	0.3394
SLC50A1	0.2815	BCL2	0.3121	FGFBP1	0.3262	ALDH1A1	0.3398
NOS2	0.2828	MSI2	0.3122	SLC7A6	0.3266	DAPK3	0.3401
PTGFRN	0.2852	CTDSPL	0.3126	SREBF2	0.3266	FAS	0.3403
CD44	0.2869	TFRC	0.3126	VDR	0.3271	SLC40A1	0.3403
DFFA	0.2869	TDG	0.3127	SPDEF	0.3271	SLC30A5	0.3407
EIF6	0.2881	GRB7	0.3129	HMGB1	0.3274	PLIN2	0.3411
SLC6A14	0.2888	HRH1	0.3131	HIST3H3	0.3284	PTPN11	0.3414
MAPK8	0.2889	TNK2	0.3133	ILF3	0.3284	PRSS23	0.3415
PTGES	0.2909	ACAT1	0.3135	SLCO2A1	0.3286	HOXA10	0.3429
CHAC1	0.2919	SLC39A4	0.3135	MMP1	0.3287	PLCD1	0.3436
DPP4	0.292	CDK15	0.3155	BECN1	0.329	PPAP2B	0.344
SLC9A2	0.2935	CYB5R1	0.3159	PLS3	0.3292	FDFT1	0.3446
BMP4	0.294	CDCA7	0.3173	ABCC1	0.3308	KEAP1	0.3447
REG1A	0.2946	KLF4	0.3177	MUC4	0.3312	ADAM9	0.3448
LEF1	0.2959	CD36	0.3184	SLC4A4	0.3315	CASP3	0.3448
EFNB1	0.2963	TFAP2C	0.3184	BMPR2	0.332	DUOX2	0.3449
TJP3	0.2963	GREM1	0.3186	FN1	0.3325	HMOX1	0.345
COX4I1	0.2964	MPC2	0.3186	PTCH1	0.3332	LCT	0.3463

Gene	bresub error	Gene	bresub error	Gene	bresub error	Gene	bresub error
BIRC5	0.3468	DEFA5	0.3599	PRKCI	0.3729	SLC30A7	0.3872
SLC7A7	0.347	CA5B	0.3603	SPRY2	0.3729	DHFR	0.3873
IL13RA1	0.3478	PRKACB	0.3606	TP53	0.3738	ADRB1	0.3876
HIF3A	0.3484	BRD2	0.3607	NTS	0.3746	STK11	0.3876
HNRNPA1	0.3486	APOA1	0.3611	KIT	0.3762	MSH2	0.3879
PPM1D	0.3487	RASSF1	0.3613	MAML1	0.3762	PROM2	0.388
SPPL3	0.3489	LRIG1	0.3614	CFLAR	0.3767	GADD45A	0.3887
SLC39A9	0.3489	FAM129A	0.3616	SLC2A2	0.3768	NOTCH3	0.3887
EPHB4	0.3493	COX5B	0.3619	PPARD	0.3772	EGF	0.3896
COL1A1	0.3499	FOXA1	0.3638	BST1	0.3775	CDK7	0.3908
IGF2BP1	0.3505	TSC22D3	0.3641	FGFR1	0.3775	EPHB6	0.3915
STAT3	0.351	SLC39A7	0.3643	DUSP1	0.3794	SLC16A9	0.3918
GSTM3	0.3515	ECM1	0.3654	LATS2	0.3802	SLC51A	0.3921
CAMK2D	0.3517	MUC2	0.3658	XRCC6	0.3806	CLDN8	0.3924
CYP4F3	0.3517	DPYSL3	0.3659	BCL10	0.381	HOXD10	0.3925
NR3C1	0.352	CDK8	0.3671	LIG3	0.3816	TPT1	0.3926
CEACAM19	0.3529	ITLN1	0.3672	FGF18	0.3819	ZDHHC20	0.3926
PTPN22	0.3533	TAT	0.3673	SLC35D1	0.3826	KLK1	0.393
BAK1	0.3534	PDE4C	0.3674	YWHAH	0.3829	AXIN2	0.393
PLA2G4D	0.3535	FGFR3	0.3675	KITLG	0.3829	POLG	0.3932
CCL15	0.3539	MYB	0.368	GPX1	0.3833	NEUROD1	0.3934
ALPI	0.3546	BMP2	0.3681	RUNX3	0.3834	DOT1L	0.3937
AGPAT3	0.3551	LYPLA1	0.3685	TJP1	0.3835	FOXJ3	0.3937
WNT7A	0.3561	ADAM10	0.3686	HNF4G	0.3836	SLC22A23	0.3938
CA7	0.3562	BCL2A1	0.3687	LAMA1	0.3838	ACKR2	0.394
DEFA6	0.3565	CHP1	0.3689	IFNAR1	0.3846	TYRO3	0.394
AIFM2	0.3572	SLC9A8	0.3695	GADD45B	0.3847	SERPINB2	0.3941
DDX11	0.3576	ARRB2	0.3698	GABRB3	0.385	PLA2G10	0.3944
HOXB7	0.3577	MAPK7	0.3698	SLFN5	0.3852	FOXA3	0.3944
CAV1	0.3586	PLAU	0.3699	PTGS2	0.3855	ABCG2	0.3949
ABCG5	0.3593	COLEC12	0.3701	TNFRSF10B	0.3856	MMP9	0.395
SLC38A2	0.3593	MELK	0.3701	DDR2	0.3858	EPAS1	0.3959
VIL1	0.3594	CXCL16	0.3703	FOXJ2	0.3864	FFAR2	0.396
CA4	0.3595	SLC35B3	0.3709	BACH1	0.3865	AATF	0.3963
OTUB1	0.3595	HLTF	0.3711	IL6ST	0.3865	GEMIN6	0.3966
ITGB2	0.3597	PIWIL2	0.3723	HMGCR	0.387	BTD	0.3969
SPI1	0.3598	SCD	0.3726	SELL	0.3871	HOXC6	0.3969

Gene	bresub error	Gene	bresub error	Gene	bresub error	Gene	bresub error
BCL2L11	0.397	CD59	0.4099	FASN	0.4189	BMF	0.4275
ERBB4	0.397	SLC5A11	0.41	NFIL3	0.4193	MUC3A	0.4279
APC	0.3972	HHIP	0.4101	SP1	0.4197	SCP2	0.4283
NCAM1	0.3974	P4HB	0.4109	NFKB2	0.4199	CACNA1C	0.4284
HDAC2	0.3976	RELA	0.4113	TNFRSF21	0.4202	ATG16L1	0.429
TFCP2L1	0.398	REG4	0.4115	TLR4	0.4204	CA13	0.4291
PLD3	0.3981	AXIN1	0.4118	JAK1	0.4205	CASP4	0.4291
OAT	0.3987	PLAUR	0.4122	ANXA1	0.4212	GCLM	0.4293
MAML3	0.3988	CDX2	0.4123	LRRK2	0.4213	FLT1	0.4296
GDF15	0.3994	MLLT4	0.4125	MAPK10	0.4213	NOG	0.4296
NPAT	0.3999	GJB2	0.4126	SPIB	0.4214	SIRT1	0.4298
HOXD13	0.4002	CDK2	0.4127	FABP6	0.4215	TNFRSF1B	0.431
RBL2	0.4006	CTNNA1	0.4129	YWHAE	0.422	CEACAM7	0.4312
PARK7	0.4013	S100A8	0.4132	POLB	0.4222	NUMB	0.4315
SST	0.4017	ILKAP	0.4133	WNT3	0.4224	CFTR	0.4316
SLC9A3R1	0.4017	TNFSF13B	0.4133	ILF2	0.4228	SLC31A1	0.4318
TLR2	0.4025	COL6A3	0.4135	SLC37A4	0.4228	SFN	0.432
SLC1A1	0.4029	BAD	0.4136	DNAJB6	0.423	YAP1	0.432
MEF2C	0.4033	FOXO1	0.4147	ALCAM	0.4233	SLC30A6	0.4321
SPP1	0.4039	ODC1	0.4148	LIFR	0.4234	CDKAL1	0.4325
SLC35A3	0.4048	LGR4	0.4155	NR2C2	0.4237	SLC16A1	0.4334
HPGD	0.4048	NRCAM	0.4157	WNT4	0.4238	PRKCA	0.4348
SLC30A4	0.4051	PRKCD	0.4157	MEP1A	0.4244	MAB21L2	0.4349
SLC25A33	0.4056	ACSL4	0.4159	SLC16A4	0.4248	LAMP1	0.4352
SLC39A5	0.4065	BAX	0.4159	PODXL	0.4249	CA3	0.4353
TNFRSF1A	0.4068	APEX1	0.4164	NRIP1	0.425	MAPK13	0.4355
TPM3	0.4072	HAS3	0.4164	YWHAZ	0.4256	ATG5	0.4358
SLC38A1	0.4074	DCN	0.4166	ACADS	0.4257	CD9	0.4359
PECAM1	0.4077	POU2F3	0.4167	SLC23A3	0.4257	GSN	0.436
PYGO2	0.4079	BBC3	0.4169	MSH3	0.4258	MYO9B	0.4366
REG3A	0.408	SLC7A9	0.4169	SOD1	0.4258	VIM	0.4368
MGAT3	0.4082	NOTCH4	0.4175	FTH1	0.426	CA14	0.4368
MALL	0.4084	AQP11	0.4178	CDH5	0.4261	RELB	0.4374
GJC1	0.4086	HMGCS2	0.4178	PRKDC	0.4263	LGALS3	0.4377
ANGPTL4	0.4089	PDK1	0.4181	WNT2B	0.4264	THRA	0.4385
RBP4	0.4091	C11orf30	0.4186	SLC3A2	0.4269	ATF4	0.4401
MPC1	0.4091	IL22RA1	0.4188	MAP2K2	0.4271	APOB	0.441

Gene	bresub error	Gene	bresub error	Gene	bresub error	Gene	bresub error
PLAA	0.441	RPS4X	0.4452	FOXP1	0.4494	AMPD3	0.4542
PLA2G7	0.4416	SLC25A20	0.4452	HOXB6	0.4499	HCAR2	0.4545
YY1	0.4416	TNFSF10	0.4454	PRKCB	0.4502	JUN	0.4545
KDM4C	0.4419	DUT	0.4455	PLCB3	0.4507	SDC1	0.4545
NOTCH1	0.4424	SLC34A2	0.4457	IL2RG	0.4509	BCL2L1	0.4546
PTK2B	0.4427	SEPTIN4	0.4457	NANOG	0.4509	PCDH7	0.4548
SLC28A2	0.4429	TLR3	0.4462	PTPN6	0.4513	SLC4A7	0.4549
FZD7	0.4431	MACC1	0.4463	KCNH2	0.4514	TGFA	0.4549
RCN3	0.4434	CDK2AP1	0.4463	MEIS1	0.4515	XIST	0.455
NEU3	0.4438	SYP	0.447	CCL28	0.4517	TNFAIP3	0.455
BCL2L2	0.4441	CA12	0.4473	TSC2	0.4517	EP400	0.4553
RBL1	0.4442	CKS2	0.4476	PPARGC1A	0.4519	MXI1	0.4553
CKAP4	0.4442	SLC25A13	0.4478	HOXB3	0.452	ID3	0.4555
YWHAB	0.4443	TLR5	0.4482	SOD2	0.4521	CA1	0.4557
LPAR4	0.4446	FOS	0.4489	CXCL2	0.4524	NOS1	0.4558
S100A9	0.4448	FLNB	0.4491	ERBB2	0.4532	GNL1	0.456
HSPD1	0.4449	SLC5A9	0.4492	HOXD1	0.4539	PTPRD	0.4562

Table S9. Top 500 high-ENL Two-Gene LDA Classifiers. The top 500 two-gene linear discriminant analysis classifiers and their respective misclassification rate in flaxseed lignan supplement vs placebo treatment in high-ENL excreters.

Gene 1	Gene 2	bresub error
MMP1	PTGIR	0.1568
CYP4F3	PTGIR	0.1721
DLL1	PTGIR	0.1729
PLS3	PTGIR	0.1741
FOXA1	KCTD12	0.1770
COX4I1	SLC39A4	0.1785
KCTD12	MAPK13	0.1788
KCTD12	RBL2	0.1846
NR6A1	PTGIR	0.1847
VDR	KCTD12	0.1848
CDKN1C	KCTD12	0.1850
PTGFRN	KCTD12	0.1876
OTUB1	PTGIR	0.1891
DPP4	SLC17A5	0.1895
DPP4	REG1A	0.1911
KCTD12	NOS2	0.1935
DPP4	MAPK13	0.1941
DPP4	SLC39A4	0.1942
FGFR3	KCTD12	0.1950
COX4I1	FOXO3	0.1951
REG1A	SPDEF	0.1953
DPP4	TNFRSF10A	0.1966
KCTD12	SPDEF	0.1981
SLC39A5	KCTD12	0.1989
CYB5R1	TNFRSF10A	0.1991
KCTD12	TFAP2C	0.1993
CCL15	KCTD12	0.1994
DPP4	PTGFRN	0.1996
OTUB1	EPHB1	0.2002
FOXO3	SLC39A4	0.2002
BMP4	OTUB1	0.2004
KCTD12	SORD	0.2008
KCTD12	THRA	0.2008
KCTD12	RELA	0.2014
BST1	OTUB1	0.2015

Gene 1	Gene 2	bresub error
BCL2	KCTD12	0.2018
PTGIR	TRAFD1	0.2019
OTUB1	DPYSL3	0.2020
PPM1D	KCTD12	0.2020
CYB5R1	KCTD12	0.2021
BMP4	EPHB1	0.2024
BCL2L14	PTGIR	0.2024
KCTD12	TNFRSF10B	0.2025
SAT2	KCTD12	0.2025
LCT	PTGIR	0.2026
ELP3	SPDEF	0.2026
DEFA5	KCTD12	0.2027
DPP4	DEFA5	0.2028
OTUB1	PAX6	0.2028
PROX1	PTGIR	0.2034
GCM1	KCTD12	0.2035
GJC1	KCTD12	0.2035
FOXA1	TFAP2C	0.2036
KCTD12	SLC2A5	0.2037
CSPG5	KCTD12	0.2038
DPP4	VDR	0.2041
DPEP1	PTGIR	0.2045
PCDH7	KCTD12	0.2049
DPP4	TFAP2C	0.2050
KCTD12	LIG3	0.2051
COX4I1	CTDSPL	0.2054
GCM1	PKM	0.2055
CDCA7	KCTD12	0.2055
FOXO1	KCTD12	0.2057
KCTD12	MEN1	0.2057
PARD3	PTGIR	0.2057
RBP4	KCTD12	0.2058
KCTD12	SLC28A1	0.2059
SLC50A1	COX4I1	0.2059
CTDSPL	KCTD12	0.2061

Gene 1	Gene 2	bresub error
COX4I1	MAPK8	0.2061
KCTD12	MAB21L2	0.2062
DPP4	SPDEF	0.2063
BMP4	NR6A1	0.2063
OTUB1	CYP4F3	0.2065
DEFA6	KCTD12	0.2067
AXIN2	KCTD12	0.2067
ELP3	KCTD12	0.2068
KCTD12	MYO9B	0.2069
GRB7	KCTD12	0.2070
BMP4	GCM1	0.2071
BMF	PTGIR	0.2073
PTGFRN	CYB5R1	0.2083
KCTD12	SYP	0.2088
KCTD12	SPI1	0.2090
BCL2L1	KCTD12	0.2090
DPP4	ELP3	0.2090
KCTD12	KIT	0.2092
FOXO3	SLC30A5	0.2092
F11R	FOXO3	0.2093
PTGIR	WNT5A	0.2094
SLC9A2	TNFRSF10A	0.2095
SLC30A5	SLC39A4	0.2095
ELP3	SLC39A4	0.2095
ACSL4	CYB5R1	0.2095
KCTD12	PTPN22	0.2096
IL36RN	PTGIR	0.2096
GPX1	KCTD12	0.2097
BMP4	PAX6	0.2097
BMI1	KCTD12	0.2099
GSTA4	KCTD12	0.2100
SPDEF	TFAP2C	0.2101
PTGFRN	TFAP2C	0.2103
HOXD10	KCTD12	0.2103
BMP4	MMP1	0.2104
ACSL4	KCTD12	0.2104
KCTD12	TSC2	0.2104
CTDSPL	TNFRSF10A	0.2105

Gene 1	Gene 2	bresub error
KCTD12	TNF	0.2109
KCTD12	PDE4C	0.2109
OTUB1	CDK15	0.2109
DPP4	SLC28A1	0.2110
BMI1	COX4I1	0.2110
BECN1	KCTD12	0.2111
BCL2	CYB5R1	0.2113
DPP4	FOXA3	0.2116
AQP11	KCTD12	0.2117
CA14	KCTD12	0.2119
LEF1	CYB5R1	0.2119
KCTD12	SLC39A4	0.2123
DPP4	CTDSPL	0.2125
RB1	KCTD12	0.2127
PTGFRN	MAPK7	0.2128
REG1A	NOS2	0.2130
COX4I1	TTN	0.2131
DPP4	PDE4C	0.2132
PTGFRN	PECAM1	0.2132
KCTD12	PCNA	0.2134
IKBKAP	KCTD12	0.2135
DUT	KCTD12	0.2135
ATP2A3	KCTD12	0.2137
DPP4	SLC7A7	0.2137
DEFA5	TNFRSF10A	0.2139
DPP4	HIST3H3	0.2140
DPP4	TFRC	0.2141
CA7	GCM1	0.2141
GCM1	LCT	0.2141
EPHB6	KCTD12	0.2142
SLC9A2	SPDEF	0.2143
FGF9	KCTD12	0.2143
LPAR4	KCTD12	0.2144
SPDEF	SPI1	0.2145
GCM1	PROX1	0.2145
REG1A	CTDSPL	0.2145
ODC1	PARD3	0.2146
EPHB1	PARD3	0.2146

Gene 1	Gene 2	bresub error
KCTD12	TNFRSF10A	0.2146
SLC39A4	TJP3	0.2147
COX4I1	SLC7A7	0.2148
DLL1	GCM1	0.2148
DPP4	TNK2	0.2148
PPM1D	SPDEF	0.2149
MMP1	PARD3	0.2150
DOT1L	MMP1	0.2150
CCL15	SLC30A5	0.2150
KCTD12	PSME2	0.2151
SLC50A1	FOXO3	0.2152
CYB5R1	TFAP2C	0.2153
CDCA7	CYB5R1	0.2153
F11R	DEFA5	0.2153
DLL1	MMP1	0.2154
CYB5R1	SLC28A1	0.2154
KCTD12	SLC2A2	0.2154
BMI1	TNFRSF10A	0.2155
SLC9A2	KCTD12	0.2155
KCTD12	PAWR	0.2155
GCM1	KEAP1	0.2157
MYO9B	SPDEF	0.2158
FGFR2	KCTD12	0.2158
XIST	PTGIR	0.2159
CYB5R1	HMGCL	0.2159
GCM1	NIFK	0.2159
LEF1	KCTD12	0.2159
CCL15	TNFRSF10A	0.2159
BCL2	DPP4	0.2160
KCTD12	MAML1	0.2160
SLC2A5	SPDEF	0.2160
OTUB1	PLD3	0.2161
BECN1	TNFRSF10A	0.2161
PRKCH	EPHB1	0.2161
PARD3	PLD3	0.2161
NRCAM	KCTD12	0.2161
PTGFRN	CTDSPL	0.2161
F11R	SLC39A4	0.2161

Gene 1	Gene 2	bresub error
SDC1	GCM1	0.2162
KCTD12	SLC38A2	0.2163
DLL1	NR6A1	0.2164
OTUB1	PROX1	0.2165
RUNX2	SLC39A4	0.2165
DPP4	KCTD12	0.2165
BAK1	PTGIR	0.2168
IFITM2	KCTD12	0.2169
DPP4	LEF1	0.2169
CYP4F3	EPHB1	0.2169
FOXO3	IL18	0.2170
GABRB3	PARD3	0.2170
DPP4	PDE6D	0.2171
PTGFRN	CCL15	0.2172
OTUB1	GCM1	0.2172
DEFA6	TNFRSF10A	0.2173
KCTD12	NOTCH1	0.2174
BCL2	MAPK7	0.2175
DPP4	BMI1	0.2176
LEF1	MAPK7	0.2177
REG1A	CCL15	0.2177
EP400	KCTD12	0.2177
F11R	CCL15	0.2177
SORD	TNFRSF10A	0.2178
REG1A	CYB5R1	0.2180
ANXA5	GCM1	0.2180
DPP4	FOXO3	0.2180
KCTD12	MFSD2A	0.2181
DPP4	CCL28	0.2181
SORD	SPDEF	0.2182
PRKCH	PTGIR	0.2182
PCDH7	SPDEF	0.2183
DLL1	EPHB1	0.2183
CYP4F3	PLD3	0.2183
GCM1	ID2	0.2184
ACSL4	MAPK7	0.2185
PRKCH	DLL1	0.2185
MMP1	ODC1	0.2185

Gene 1	Gene 2	bresub error
FOXA1	FOXO3	0.2186
CTDSPL	IL18	0.2186
FOXK2	PTGIR	0.2186
SLC17A5	IL18	0.2187
CYP4F3	DFFA	0.2187
KCTD12	SLC1A4	0.2188
PRKCH	DFFA	0.2188
BMP4	CYP4F3	0.2189
F11R	SLC17A5	0.2191
PTGFRN	NOS2	0.2192
REG1A	PDE4C	0.2193
CA14	PTGIR	0.2193
KCTD12	TNFSF13B	0.2193
BIRC5	PLD3	0.2194
SLC50A1	TJP3	0.2194
PRKCH	PAX6	0.2194
KCTD12	PLD3	0.2194
DFFA	PROX1	0.2195
GCM1	PLA2G4D	0.2195
KCTD12	SLC5A3	0.2195
PARD3	PAX6	0.2196
MMP1	PROX1	0.2196
BMP4	DFFA	0.2197
ELP3	PTGFRN	0.2197
OTUB1	ALPI	0.2198
KCTD12	ORMDL3	0.2199
PLD3	PROX1	0.2199
REG1A	MAPK7	0.2199
KCTD12	LMNB2	0.2200
PARD3	WNT5A	0.2201
CDCP1	SLC39A4	0.2201
MMP1	PLD3	0.2202
CHAC1	GCM1	0.2202
RHOB	SLC30A5	0.2202
PPARD	SLC9A2	0.2203
KCTD12	TNK2	0.2203
EPHB1	MMP1	0.2203
GCM1	PARD3	0.2204

Gene 1	Gene 2	bresub error
OTUB1	WNT5A	0.2204
EFNB1	NR6A1	0.2205
IL36RN	KCTD12	0.2205
PLA2G4C	DLL1	0.2205
REG1A	SLC28A1	0.2206
ELP3	DEFA5	0.2206
CTDSPL	SLC30A5	0.2206
ILF3	SLC39A4	0.2207
NOS1	MMP1	0.2208
DAPK3	PARD3	0.2209
PTGIR	PTPN11	0.2209
OTUB1	PTK7	0.2210
NR6A1	PARD3	0.2210
AGPAT3	KCTD12	0.2210
FOXO3	TJP3	0.2211
DFFA	PARD3	0.2211
PARD3	PTK7	0.2211
CD36	SLC39A4	0.2212
FOXP2	SLC39A4	0.2213
HIF3A	MMP1	0.2213
BCL2	PTGFRN	0.2213
FOXA1	IL18	0.2214
BMP4	ITGB2	0.2214
BCL2L14	KCTD12	0.2215
SLC3A1	ODC1	0.2215
PTGFRN	BMI1	0.2215
CDCP1	DEFA5	0.2216
TNF	TNFRSF10A	0.2216
PTGFRN	PCNA	0.2216
SLC3A1	DLL1	0.2217
KCTD12	SLC22A23	0.2217
LEF1	CTDSPL	0.2217
CYB5R1	SPDEF	0.2218
OTUB1	DLL1	0.2218
ELP3	NOS2	0.2219
KCTD12	SLC4A7	0.2220
DPP4	LATS2	0.2221
MMP1	NR6A1	0.2221

Gene 1	Gene 2	bresub error
LEF1	DEFA5	0.2221
F11R	RUNX2	0.2221
AQP11	SPDEF	0.2221
MAP2K2	KCTD12	0.2221
AKTIP	GCM1	0.2222
COX4I1	SLC7A6	0.2222
REG1A	DEFA5	0.2223
GCM1	MMP1	0.2223
DPP4	SLC9A2	0.2223
SAT2	SPDEF	0.2223
F11R	COX4I1	0.2223
DPP4	TNF	0.2223
BECN1	SPDEF	0.2224
DAPK3	DFFA	0.2224
HES1	IL18	0.2224
BMP4	DLL1	0.2224
REG1A	TNK2	0.2225
PCNA	SLC30A5	0.2225
PLA2G4C	MMP1	0.2225
DCN	GCM1	0.2225
ELP3	CYB5R1	0.2225
GABRB3	PROX1	0.2226
ELP3	MAPK7	0.2226
CYP4F3	MMP1	0.2226
IGF2	KCTD12	0.2227
NR6A1	PLD3	0.2227
F11R	TNK2	0.2227
GCM1	PECAM1	0.2228
JAK2	KCTD12	0.2228
EPHB1	PROX1	0.2229
OTUB1	ODC1	0.2229
KCTD12	MMP1	0.2229
MAPK7	SLC28A1	0.2230
GPX1	SPDEF	0.2230
PPM1D	TNFRSF10A	0.2230
HIST3H3	KCTD12	0.2230
POLB	KCTD12	0.2230
PRKCH	GCM1	0.2230

Gene 1	Gene 2	bresub error
KIT	TNFRSF10A	0.2230
MAML1	SLC39A4	0.2231
DPP4	SLC7A6	0.2232
KCTD12	PDE4B	0.2233
IL1R2	KCTD12	0.2234
ITLN1	SLC39A4	0.2234
FOXA1	SLC7A6	0.2234
SLC34A2	PTGIR	0.2234
OTUB1	EPHB4	0.2234
FOXA1	TDG	0.2235
NR6A1	TIMP3	0.2235
LIG3	TNFRSF10A	0.2235
DEFA6	SPDEF	0.2236
PRKCH	PLD3	0.2236
GCM1	TIMP3	0.2237
PCDH7	SLC28A1	0.2237
DPP4	GADD45A	0.2238
BMP4	FOXJ2	0.2238
MMP1	PAX6	0.2238
KCTD12	NR5A2	0.2239
PDE4C	SLC30A5	0.2239
DPP4	CCL15	0.2239
CHI3L1	PROX1	0.2239
KEAP1	NR6A1	0.2239
SLC17A5	CDCP1	0.2240
SLC39A4	TDG	0.2240
DPP4	TFCP2L1	0.2241
OTUB1	MMP1	0.2241
NR6A1	ODC1	0.2241
EPHB1	FOXK2	0.2241
CTDSPL	TDG	0.2242
FOXA1	RUNX2	0.2242
IER3	PTGIR	0.2242
HMGCL	MAPK7	0.2242
CYB5R1	ICAM1	0.2242
BECN1	SLC28A1	0.2242
SLC50A1	HIST3H3	0.2242
F11R	MPC2	0.2243

Gene 1	Gene 2	bresub error
KCTD12	SLC23A3	0.2243
PRNP	PTGIR	0.2243
SLC28A1	SORD	0.2244
ELP3	CTDSPL	0.2245
BMP4	DAPK3	0.2245
KCTD12	RAD52	0.2245
PRKACB	SLC39A4	0.2245
COX4I1	SHH	0.2246
F11R	HIST3H3	0.2246
NR6A1	PROX1	0.2246
DEFA5	SREBF2	0.2246
REG1A	KCTD12	0.2247
SDC1	NR6A1	0.2247
OTUB1	PECAM1	0.2248
HDAC1	TJP3	0.2248
AIFM2	KCTD12	0.2248
RELA	SPDEF	0.2248
ACSL4	DPP4	0.2249
COX4I1	KIT	0.2249
GCM1	NAA10	0.2249
NOS2	TFAP2C	0.2249
PTGFRN	SPDEF	0.2249
F11R	RHOB	0.2250
RELA	TNFRSF10A	0.2250
SLC17A5	SREBF2	0.2250
CHI3L1	NR6A1	0.2250
PRKCH	WNT5A	0.2250
SLC17A5	KCTD12	0.2250
DPP4	CDCA7	0.2250
NR6A1	PKM	0.2251
CTDSPL	SLC28A1	0.2251
BMP4	KCTD12	0.2251
NR2C2	PTGIR	0.2251
PARK7	KCTD12	0.2251
CHI3L1	GCM1	0.2251
FOXO3	RUNX2	0.2252
BMI1	IL18	0.2252
PTGFRN	SORD	0.2252

Gene 1	Gene 2	bresub error
CTDSPL	SPDEF	0.2253
DPP4	GRB7	0.2253
KCTD12	P4HB	0.2253
FOXA1	HSD17B2	0.2254
PPM1D	PTGFRN	0.2254
DEFA6	SLC28A1	0.2254
FOXA1	SLC30A5	0.2254
DPP4	RELA	0.2254
F11R	FOXP2	0.2255
EPHB1	PTGIR	0.2255
HES1	MAPK8	0.2255
MSI1	EPHB1	0.2255
ADAMTS4	MMP1	0.2255
KCTD12	SLC51A	0.2255
DPP4	BECN1	0.2256
PRKCH	MMP1	0.2256
GSTA4	SPDEF	0.2256
PPM1D	NOS2	0.2256
EPHB1	IER3	0.2256
SAT2	TFAP2C	0.2256
PCNA	SLC28A1	0.2256
CCL15	TJP3	0.2256
EFNB1	GCM1	0.2257
CCL15	SREBF2	0.2257
F11R	TNF	0.2258
KCTD12	PIK3CA	0.2258
F11R	CD36	0.2258
SLC39A4	TTN	0.2258
SLC10A2	CYB5R1	0.2259
ELP3	TNK2	0.2259
NIFK	NR6A1	0.2259
PTAFR	KCTD12	0.2259
HOXB8	OTUB1	0.2259
EPHB1	TIMP3	0.2259
LEF1	BECN1	0.2260
IL18	SLC39A4	0.2260
HCAR2	KCTD12	0.2260
HMGCS1	KCTD12	0.2260

Gene 1	Gene 2	bresub error
LEF1	REG1A	0.2260
MMP1	ZNF365	0.2261
ELP3	HIST3H3	0.2261
SLC39A4	SREBF2	0.2261
DPP4	COX4I1	0.2261
F11R	MAPK8	0.2262
BMP4	SLC3A1	0.2262
KCTD12	SLC1A1	0.2262
LCT	NR6A1	0.2262
DPP4	PCNA	0.2262
FOXO3	PRKAR1A	0.2262
G6PD	KCTD12	0.2262
SPDEF	TNFRSF10B	0.2263
BMI1	SLC30A5	0.2263
PLA2G4C	CHI3L1	0.2263
OTUB1	GABRB3	0.2264
PCNA	SPDEF	0.2264
CA7	NR6A1	0.2264
PCNA	TJP3	0.2264
CDCA7	MAPK7	0.2264
RHOB	TJP3	0.2264
COL3A1	PTGIR	0.2264
CYB5R1	TLR3	0.2266
KCTD12	PECAM1	0.2266
DPP4	MAPK8	0.2267

Gene 1	Gene 2	bresub error
FOXK2	PLD3	0.2267
SLC17A5	RUNX2	0.2267
FOXD2	KCTD12	0.2267
F11R	TDG	0.2267
MAPK7	TFAP2C	0.2267
SYP	TNFRSF10A	0.2268
KCTD12	MUC19	0.2268
CAT	SLC39A4	0.2268
BMP4	PARD3	0.2268
CDK8	DPP4	0.2268
KCTD12	MAPK7	0.2269
F11R	TFRC	0.2269
SLC3A1	PARD3	0.2269
ANGPTL4	GCM1	0.2269
FOXO1	TFAP2C	0.2269
BMI1	TJP3	0.2269
SLC39A4	TFRC	0.2270
BECN1	IL18	0.2270
EPHB1	PPAP2B	0.2270
LEF1	PCNA	0.2271
MSI1	PTGIR	0.2271
COX4I1	SLCO2A1	0.2271
BMI1	SLC28A1	0.2272
MAB21L2	TNFRSF10A	0.2272
DFFA	DLL1	0.2272

Table S10. Top 500 high-ENL Three-Gene LDA Classifiers. The top 500 three-gene linear discriminant analysis classifiers and their respective misclassification rate in flaxseed lignan supplement vs placebo treatment in high-ENL excreters.

Gene 1	Gene 2	Gene 3	bresub error	Gene 1	Gene 2	Gene 3	bresub error
NRCAM	GCM1	MMP1	0.1157	DPP4	REG1A	MAPK13	0.1460
NRCAM	MMP1	PLD3	0.1282	DPEP1	MMP1	PTGIR	0.1460
CA14	GCM1	MMP1	0.1291	BST1	MMP1	PLD3	0.1460
OTUB1	CYP4F3	PTGIR	0.1296	OTUB1	MMP1	PTGIR	0.1465
GCM1	MMP1	PECAM1	0.1298	AQP11	DPP4	MAPK13	0.1472
BMP4	GCM1	MMP1	0.1331	ESR1	MMP1	PTGIR	0.1473
COX4I1	FOXO3	SLC39A4	0.1340	DPP4	ELP3	MAPK13	0.1476
DLL1	MMP1	PTGIR	0.1342	SLC34A2	DLL1	PTGIR	0.1476
MMP1	PTGIR	WNT5A	0.1346	KCTD12	MEN1	RBL2	0.1478
CYP4F3	MMP1	PTGIR	0.1375	DPP4	DEFA5	MAPK13	0.1478
DLL1	GCM1	MMP1	0.1377	OTUB1	PLS3	PTGIR	0.1480
CYP4F3	NR6A1	PTGIR	0.1380	PTGFRN	KCTD12	RBL2	0.1481
GABRB3	MMP1	PTGIR	0.1385	PLS3	ITGB1	PTGIR	0.1482
FOXA1	KCTD12	TFAP2C	0.1387	LPAR1	MMP1	PTGIR	0.1484
SLC34A2	MMP1	PTGIR	0.1394	GCM1	MMP1	TSC2	0.1485
SLC3A1	MMP1	PTGIR	0.1410	TBRG1	MMP1	PTGIR	0.1487
SLC34A2	CYP4F3	PTGIR	0.1413	DHFR	MMP1	PTGIR	0.1489
FOXJ2	MMP1	PTGIR	0.1413	NRCAM	SLC39A5	MMP1	0.1489
XIST	DLL1	PTGIR	0.1413	PLS3	PROX1	PTGIR	0.1489
DPP4	SLC9A2	VDR	0.1414	COL3A1	DLL1	PTGIR	0.1491
SST	CYP4F3	PTGIR	0.1417	ITGB2	CYP4F3	PTGIR	0.1491
DLL1	NR6A1	PTGIR	0.1434	PRNP	MMP1	PTGIR	0.1491
KCTD12	NOS2	RBL2	0.1434	MMP1	NR6A1	PTGIR	0.1495
COX4I1	SLC39A4	TFCP2L1	0.1443	LEF1	KCTD12	RBL2	0.1496
IL36RN	DLL1	PTGIR	0.1450	CDKN1C	KCTD12	SLC28A1	0.1496
ISL1	MMP1	PTGIR	0.1451	DPEP1	CYP4F3	PTGIR	0.1497
CYP4F3	FGF18	PTGIR	0.1452	DPP4	REG1A	VDR	0.1497
CYP4F3	ITGB1	PTGIR	0.1453	OTUB1	DLL1	PTGIR	0.1498
CYP4F3	FOXK2	PTGIR	0.1453	XIST	CYP4F3	PTGIR	0.1499
IL36RN	CYP4F3	PTGIR	0.1453	DPP4	SLFN5	VDR	0.1501
PTGFRN	KCTD12	MAPK13	0.1453	DLL1	ITGB1	PTGIR	0.1505
CEACAM19	MMP1	PTGIR	0.1455	GCM1	MMP1	PTGIR	0.1505

Gene 1	Gene 2	Gene 3	bresub error	Gene 1	Gene 2	Gene 3	bresub error
CYP4F3	DAPK3	PTGIR	0.1505	PTGFRN	CDKN1C	KCTD12	0.1537
MMP1	PLD3	PTGIR	0.1507	HAS2	MMP1	PTGIR	0.1538
XIST	MMP1	PTGIR	0.1507	CCDC80	MMP1	PTGIR	0.1539
DPP4	PTGFRN	MAPK13	0.1509	KCTD12	LRIG1	RBL2	0.1540
CDKN1C	KCTD12	PTPN22	0.1511	BCL2	KCTD12	MAPK13	0.1541
CA14	MMP1	PTGIR	0.1511	CYP4F3	MAPK13	PTGIR	0.1541
PLS3	MMP1	PTGIR	0.1514	PLS3	PTGIR	TRAFD1	0.1542
ITGB2	DLL1	PTGIR	0.1515	BMF	NR6A1	PTGIR	0.1542
FPR1	MMP1	PTGIR	0.1515	MMP1	PTGIR	SLC28A1	0.1543
RBP4	ACADS	KCTD12	0.1516	PPAP2A	MMP1	PTGIR	0.1543
BCL2	KCTD12	RBL2	0.1517	COX4I1	CTDSPL	SLC39A4	0.1543
PLS3	EPHB1	PTGIR	0.1518	PTGFRN	FGFR3	KCTD12	0.1544
PRNP	CYP4F3	PTGIR	0.1519	GCM1	MMP1	PLA2G7	0.1544
BMP4	MMP1	PTGIR	0.1520	MSI1	CYP4F3	PTGIR	0.1545
DPP4	HMGCS1	MAPK13	0.1521	ILKAP	MMP1	PTGIR	0.1545
PTGFRN	GCM1	KCTD12	0.1521	RBP4	MMP1	PTGIR	0.1546
COL3A1	MMP1	PTGIR	0.1522	KCTD12	RBL2	SLC28A1	0.1546
IGF1	MMP1	PTGIR	0.1525	AQP11	KCTD12	MAPK13	0.1549
ACSL4	KCTD12	RBL2	0.1526	BCL2L14	DLL1	PTGIR	0.1549
LPAR1	CYP4F3	PTGIR	0.1527	BCL2L14	SLC34A2	PTGIR	0.1550
CYP4F3	GCM1	MMP1	0.1527	CYP4F3	MMP1	PLD3	0.1550
DPP4	MAPK13	SLC39A4	0.1528	SLC9A2	KCTD12	TNFRSF10A	0.1551
ITGB2	MMP1	PTGIR	0.1528	CSPG5	MMP1	PTGIR	0.1551
CDCA7	KCTD12	RBL2	0.1528	PRKAR1B	MMP1	PTGIR	0.1551
IL36RN	MMP1	PTGIR	0.1528	DLL1	MEN1	PTGIR	0.1551
CYP4F3	EPHB4	PTGIR	0.1528	IL36RN	NR6A1	PTGIR	0.1551
ITGAL	MMP1	PTGIR	0.1532	COX4I1	IFNAR1	SLC39A4	0.1552
CYP4F3	EPHB1	PTGIR	0.1533	EPHB6	MMP1	PTGIR	0.1554
BAK1	NR6A1	PTGIR	0.1533	BST1	MMP1	PTGIR	0.1554
DLL1	EPHB1	PTGIR	0.1533	DPP4	COX4I1	SLC39A4	0.1555
PRNP	DLL1	PTGIR	0.1533	DAPK3	DLL1	PTGIR	0.1557
PLS3	FOXK2	PTGIR	0.1533	LEF1	FOXA1	KCTD12	0.1557
DPEP1	DLL1	PTGIR	0.1533	FGFR1	MMP1	PTGIR	0.1558
GCM1	MMP1	REL	0.1533	DPEP1	PLS3	PTGIR	0.1559
LPAR1	DLL1	PTGIR	0.1536	CYP4F3	PTGIR	TSC2	0.1559
EPHB1	MMP1	PTGIR	0.1536	MMP1	PECAM1	PTGIR	0.1560
MAPK14	MMP1	PTGIR	0.1537	PTGFRN	KCTD12	MEN1	0.1561

Gene 1	Gene 2	Gene 3	bresub error	Gene 1	Gene 2	Gene 3	bresub error
REG1A	SLC28A1	SPDEF	0.1561	NRCAM	AIFM2	KCTD12	0.1584
SLFN5	VDR	KCTD12	0.1562	MMP1	PTGIR	TAT	0.1584
NR6A1	PROX1	PTGIR	0.1562	CYP4F3	GCM1	PTGIR	0.1585
BCL2L14	MMP1	PTGIR	0.1563	DLL1	PAX6	PTGIR	0.1585
MEN1	MMP1	PTGIR	0.1563	NR6A1	PARD3	PTGIR	0.1586
WNT4	MMP1	PTGIR	0.1564	AIFM2	MMP1	PTGIR	0.1586
EPHB4	MMP1	PTGIR	0.1564	MSI1	DLL1	PTGIR	0.1586
DPP4	ELP3	VDR	0.1567	FOXK2	NR6A1	PTGIR	0.1587
KCTD12	MAPK13	SLC28A1	0.1567	TGFB2	MMP1	PTGIR	0.1589
SLC35A2	COX4I1	SLC39A4	0.1567	KDM4A	MMP1	PTGIR	0.1590
MMP1	PAX6	PTGIR	0.1568	LCT	NR6A1	PTGIR	0.1591
DLL1	GCM1	MMP9	0.1569	RASSF1	MMP1	PTGIR	0.1591
DDX11	MMP1	PTGIR	0.1569	SLC9A2	KCTD12	TFAP2C	0.1592
GPX1	KCTD12	RBL2	0.1570	NRCAM	PPARD	SLC9A2	0.1592
PTGFRN	KCTD12	TFAP2C	0.1571	CYP4F3	PAX6	PTGIR	0.1592
AQP11	KCTD12	RBL2	0.1571	HOXD8	MMP1	PTGIR	0.1594
CYP4F3	ILKAP	PTGIR	0.1571	NRCAM	MMP1	ODC1	0.1594
BIRC5	CYP4F3	PTGIR	0.1572	CYP4F3	GABRB3	PTGIR	0.1594
PLS3	PAX6	PTGIR	0.1573	HOXA2	DLL1	PTGIR	0.1594
OTUB1	EPHB4	PTGIR	0.1573	DLL1	FOXK2	PTGIR	0.1594
SLC9A2	CYP4F3	PTGIR	0.1573	BMF	PLS3	PTGIR	0.1595
PLS3	FGF18	PTGIR	0.1575	GCM1	KCTD12	NOS2	0.1595
GCM1	KCTD12	SLC28A1	0.1575	DUT	GCM1	MMP1	0.1595
CYP4F3	MEN1	PTGIR	0.1576	MMP1	PTGIR	TNFRSF10D	0.1596
KCTD12	LRIG1	MAPK13	0.1577	SLC3A1	CYP4F3	PTGIR	0.1596
VDR	KCTD12	NOG	0.1577	COX4I1	KIT	SLC39A4	0.1597
MMP1	PTGIR	PTK7	0.1578	FOXJ2	NR6A1	PTGIR	0.1597
FGFR3	KCTD12	TFAP2C	0.1578	DPP4	PTGFRN	REG1A	0.1597
KCTD12	RBL2	SLC2A5	0.1579	HOXA9	MMP1	PTGIR	0.1598
PLS3	DFFA	PTGIR	0.1580	CDKN1C	KCTD12	SPDEF	0.1599
CCL15	COX4I1	SLC39A4	0.1581	KCTD12	MAPK13	TNFRSF10B	0.1599
KCTD12	SLC28A1	SPDEF	0.1582	DPEP1	VDR	KCTD12	0.1599
IGF1	DLL1	PTGIR	0.1582	HOXD1	NR6A1	PTGIR	0.1600
BRIX1	MMP1	PTGIR	0.1583	GRB7	KCTD12	RBL2	0.1601
SST	DLL1	PTGIR	0.1583	HSPD1	CYP4F3	PTGIR	0.1601
CEACAM3	MMP1	PTGIR	0.1583	DPP4	MAPK13	SORD	0.1601
FOXA1	KCTD12	SLC28A1	0.1584	PTGFRN	KCTD12	SPDEF	0.1602

Gene 1	Gene 2	Gene 3	bresub error	Gene 1	Gene 2	Gene 3	bresub error
CCDC80	PLS3	PTGIR	0.1602	CCDC80	CYP4F3	PTGIR	0.1614
SLC9A2	KCTD12	SPDEF	0.1602	NRCAM	MMP1	PTGIR	0.1614
XIST	NR6A1	PTGIR	0.1602	BCL2L14	CYP4F3	PTGIR	0.1615
ABCC1	MMP1	PTGIR	0.1602	DLL1	FGF18	PTGIR	0.1615
DAPK3	MMP1	PTGIR	0.1603	PTGFRN	CA14	KCTD12	0.1615
KCTD12	MAPK13	TFAP2C	0.1603	ACSL4	MMP1	PTGIR	0.1616
COX4I1	MAML1	SLC39A4	0.1604	PTGFRN	ALPI	KCTD12	0.1616
LPAR1	PLS3	PTGIR	0.1604	OTUB1	DLL1	GCM1	0.1616
DPP4	REG1A	TFAP2C	0.1605	ISL1	DLL1	PTGIR	0.1616
PLS3	DAPK3	PTGIR	0.1605	SLC35B3	COX4I1	SLC39A4	0.1616
KCTD12	MEN1	SPDEF	0.1605	MMP1	PTGIR	RCN3	0.1617
FGF5	MMP1	PTGIR	0.1605	DPP4	ELP3	PTGFRN	0.1618
IER3	NR6A1	PTGIR	0.1606	CDCA7	KCTD12	MAPK13	0.1618
PRKCH	CYP4F3	PTGIR	0.1607	CAV1	KCTD12	RBL2	0.1618
SLC39A5	MMP1	PTGIR	0.1607	FGFR3	KCTD12	SLC28A1	0.1618
CYP4F3	PTGIR	PTK2	0.1609	ITGAL	DLL1	PTGIR	0.1619
PTGFRN	KCTD12	NOS2	0.1609	NR6A1	PTGIR	PTPN11	0.1619
GCM1	KCTD12	SPDEF	0.1609	DPP4	BMI1	MAPK13	0.1619
FOXA1	KCTD12	PLD3	0.1609	ACADS	KCTD12	PECAM1	0.1619
DPP4	REG1A	TSC2	0.1609	NRCAM	KCTD12	NOS2	0.1620
POR	CYP4F3	PTGIR	0.1610	HEY1	MMP1	PTGIR	0.1620
PLS3	BIRC5	PTGIR	0.1610	ERBB4	MMP1	PTGIR	0.1620
IGF1	CYP4F3	PTGIR	0.1610	KLK6	MMP1	PTGIR	0.1621
NFKB2	MMP1	PTGIR	0.1611	DLL1	MMP1	PLD3	0.1621
BCL2	KCTD12	SPDEF	0.1611	PLS3	GCM1	PTGIR	0.1621
CACNA2D1	MMP1	PTGIR	0.1611	PLS3	MEN1	PTGIR	0.1622
COX4I1	PDK1	SLC39A4	0.1611	ELP3	GCM1	KCTD12	0.1623
DPP4	FOXA1	MAPK13	0.1611	FPR1	VDR	KCTD12	0.1624
RBL1	MMP1	PTGIR	0.1612	CDCA7	CDKN1C	KCTD12	0.1624
ABCG5	MMP1	PTGIR	0.1612	SAT2	KCTD12	MAPK13	0.1625
CDCA7	GCM1	KCTD12	0.1612	WNT4	DLL1	PTGIR	0.1625
MMP1	POU2F3	PTGIR	0.1612	EPHB6	FOXA1	KCTD12	0.1625
BCL2L14	NR6A1	PTGIR	0.1612	DPP4	SLC9A2	MAPK13	0.1626
KCTD12	NOS2	SLC28A1	0.1612	MMP1	PTGIR	RBBP9	0.1626
DLL1	PTGIR	WNT5A	0.1613	HOXA2	MMP1	PTGIR	0.1626
NR6A1	PTGIR	WNT5A	0.1613	NOTCH4	MMP1	PTGIR	0.1626
NRCAM	MMP1	PTK7	0.1613	PLS3	FOXJ2	PTGIR	0.1626

Gene 1	Gene 2	Gene 3	bresub error	Gene 1	Gene 2	Gene 3	bresub error
DPP4	MAPK13	TNF	0.1626	OTUB1	PROX1	PTGIR	0.1639
PTGFRN	GRB7	KCTD12	0.1627	BCL2	KCTD12	TFAP2C	0.1640
KCTD12	MALL	RBL2	0.1627	NPAT	CYP4F3	PTGIR	0.1640
PTGFRN	KCTD12	LRIG1	0.1627	FOXP4	MMP1	PTGIR	0.1640
PTGFRN	KCTD12	SLC23A3	0.1627	APEX1	MMP1	PTGIR	0.1640
PARD3	PTGIR	TRAFD1	0.1627	CYP4F3	GCM1	MMP9	0.1641
MMP1	NAIP	PTGIR	0.1627	DPP4	VDR	SLC39A4	0.1641
RASSF1	GCM1	MMP1	0.1628	MMP1	PTGIR	TRAFD1	0.1642
ALPI	MMP1	PTGIR	0.1628	MAPK14	CYP4F3	PTGIR	0.1642
KCTD12	NOS2	TSC2	0.1629	DPP4	REG1A	SLC28A1	0.1642
SLC5A9	MMP1	PTGIR	0.1629	BCL2	CDKN1C	KCTD12	0.1642
DUT	MMP1	PTGIR	0.1630	DUT	SLC9A2	KCTD12	0.1642
PLS3	GABRB3	PTGIR	0.1630	SLC27A2	CYP4F3	PTGIR	0.1642
CCDC80	DLL1	PTGIR	0.1630	OTUB1	FOXJ2	PTGIR	0.1642
ALOX15	DLL1	PTGIR	0.1631	KCTD12	SPDEF	TFAP2C	0.1642
HAS2	CYP4F3	PTGIR	0.1631	DPP4	PTGFRN	TFAP2C	0.1643
KCTD12	RBL2	SPI1	0.1632	TGM2	MMP1	PTGIR	0.1643
SLC9A2	VDR	KCTD12	0.1632	LPAR1	VDR	KCTD12	0.1644
DLL1	GRB7	PTGIR	0.1632	DPP4	MAPK13	TNFRSF10A	0.1644
CDKN1C	KCTD12	NOS2	0.1632	PLS3	XIST	PTGIR	0.1644
DPP4	PCNA	SLC39A4	0.1633	DPEP1	NR6A1	PTGIR	0.1644
DPEP1	KCTD12	RBL2	0.1634	PTGFRN	RBP4	KCTD12	0.1645
DPP4	NPAT	SLC9A2	0.1634	DPP4	DEFA5	SLC39A4	0.1645
BST1	DLL1	PTGIR	0.1634	FOXJ2	GCM1	MMP1	0.1645
CDKAL1	DPP4	MAPK13	0.1635	KLK6	CYP4F3	PTGIR	0.1645
SLC39A5	CYP4F3	PTGIR	0.1635	DPP4	VDR	SLC2A13	0.1646
CYP4F3	DFFA	PTGIR	0.1635	WNT4	CYP4F3	PTGIR	0.1646
COX4I1	SLC39A4	SLC7A7	0.1636	GJB1	MMP1	PTGIR	0.1647
PLS3	PLD3	PTGIR	0.1636	NPAT	SLC9A2	KCTD12	0.1647
NPAT	MMP1	PTGIR	0.1636	DPP4	SLC17A5	SLC39A4	0.1647
KCTD12	MAPK13	PTPN22	0.1636	GEMIN6	CYP4F3	PTGIR	0.1647
MMP1	PTGIR	SLC25A13	0.1637	DPP4	MAPK13	PCNA	0.1647
GCM1	KCTD12	PTPN22	0.1637	CYP4F3	GRB7	PTGIR	0.1648
ADAM17	MMP1	PTGIR	0.1637	BAK1	GCM1	MMP1	0.1648
DPP4	HMGCS1	SLC39A4	0.1638	CCND1	MMP1	PTGIR	0.1648
HOXC6	MMP1	PTGIR	0.1639	IL36RN	GCM1	MMP1	0.1649
PLS3	SLC39A5	PTGIR	0.1639	CYP4F3	PTGIR	TLR2	0.1649

Gene 1	Gene 2	Gene 3	bresub error	Gene 1	Gene 2	Gene 3	bresub error
KCTD12	MAPK13	SPI1	0.1649	VDR	KCTD12	TFAP2C	0.1656
SLC5A9	GCM1	MMP1	0.1649	DPP4	PPM1D	MAPK13	0.1656
DPP4	VDR	JAK2	0.1649	VDR	KCTD12	TSC2	0.1657
UHRF1	MMP1	PTGIR	0.1649	DPP4	RELA	SLC39A4	0.1657
HSPD1	MMP1	PTGIR	0.1649	MMP1	PTGIR	SIGIRR	0.1658
CYP4F3	PTGIR	ZNF365	0.1650	AQP7	MMP1	PTGIR	0.1658
PLS3	PTGIR	ZNF365	0.1650	BAK1	IL36RN	PTGIR	0.1658
DPP4	ELP3	SLC39A4	0.1650	DPP4	ELP3	DEFA5	0.1658
FOXA1	KCTD12	SLC2A5	0.1651	CYP4F3	PTGIR	TNFRSF10D	0.1658
ALOX15	CYP4F3	PTGIR	0.1651	SLC3A1	DLL1	PTGIR	0.1658
PLS3	SST	PTGIR	0.1651	FOXA1	KCTD12	LRIG1	0.1658
IRF8	MMP1	PTGIR	0.1651	PLS3	GCM1	MMP1	0.1658
DPP4	LEF1	REG1A	0.1651	KCTD12	SLC28A1	TNFRSF10A	0.1658
REG1A	PTPN22	SPDEF	0.1651	MMP1	PTGIR	TLR5	0.1658
DUT	CYP4F3	PTGIR	0.1651	CEACAM19	NR6A1	PTGIR	0.1658
KCTD12	NOS2	SPDEF	0.1651	GEMIN6	MMP1	PTGIR	0.1659
PLS3	EPHB4	PTGIR	0.1651	GRB7	MMP1	PTGIR	0.1659
PLS3	NR6A1	PTGIR	0.1652	COX4I1	MEN1	SLC39A4	0.1659
OTUB1	MEN1	PTGIR	0.1652	ELP3	KCTD12	MAPK13	0.1659
BTD	MMP1	PTGIR	0.1652	MMP1	PLA2G7	PTGIR	0.1659
CYP4F3	ODC1	PTGIR	0.1652	GRB7	KCTD12	SPDEF	0.1659
MYB	COX4I1	SLC39A4	0.1653	ACSL4	PTGFRN	KCTD12	0.1660
SLC39A5	DLL1	PTGIR	0.1653	DOT1L	MMP1	PTGIR	0.1661
DPP4	REG1A	GCM1	0.1653	VDR	KCTD12	SLC28A1	0.1661
LEF1	VDR	KCTD12	0.1653	DPP4	GPX1	MAPK13	0.1661
CYP4F3	PTGIR	WNT5A	0.1654	KCTD12	OGG1	TFAP2C	0.1661
PLS3	NR2C2	PTGIR	0.1654	PTGFRN	KCTD12	MMP1	0.1662
LPAR1	NR6A1	PTGIR	0.1654	DUT	FOXA1	KCTD12	0.1662
DPP4	VDR	TFAP2C	0.1654	DPP4	SLC9A2	TFAP2C	0.1663
CDKN1C	KCTD12	RBL2	0.1654	KCTD12	MAPK13	TNFRSF10A	0.1663
PLS3	PTGIR	WNT5A	0.1654	COX4I1	FOXA1	SLC39A4	0.1663
CEACAM19	GCM1	MMP1	0.1654	EPHB6	DLL1	PTGIR	0.1663
CYP4F3	FGF18	ODC1	0.1654	IGF1	PLS3	PTGIR	0.1663
FGFR1	CYP4F3	PTGIR	0.1655	COL3A1	CYP4F3	PTGIR	0.1663
VDR	AIFM2	KCTD12	0.1655	HOXD1	MMP1	PTGIR	0.1664
DPP4	SLC39A4	TNF	0.1656	DPP4	PTGFRN	TNFRSF10A	0.1664
RBP4	GCM1	MMP1	0.1656	NRCAM	SLC39A5	SLC3A1	0.1664

Gene 1	Gene 2	Gene 3	bresub error
LCT	PTGIR	TRAFD1	0.1665
SLC10A2	MMP1	PTGIR	0.1665
TP63	CYP4F3	PTGIR	0.1665
BDKRB2	CYP4F3	PTGIR	0.1666
COX4I1	FOXO3	MAPK8	0.1666
DPP4	LPAR1	VDR	0.1666
ITGB1	MMP1	PTGIR	0.1666
PLS3	ILKAP	PTGIR	0.1666
DPP4	MAPK13	TNFRSF10B	0.1666
MMP1	PTGIR	SLC25A28	0.1667
BST1	GCM1	MMP1	0.1667
ADAM17	CYP4F3	PTGIR	0.1667
OTUB1	EPHB1	PTGIR	0.1667
PTAFR	MMP1	PTGIR	0.1667
PLS3	ESR1	PTGIR	0.1667
GPX1	KCTD12	MAPK13	0.1668
DLL1	GABRB3	PTGIR	0.1668
DPP4	DEFA5	GCM1	0.1668
DLL1	GSTA1	PTGIR	0.1668
PCDH7	KCTD12	RBL2	0.1668
PRKACA	CYP4F3	PTGIR	0.1668
BMP4	GABRB3	MMP1	0.1669
DPP4	BMI1	DEFA5	0.1669
SLC9A2	DLL1	PTGIR	0.1669
PLS3	GSTA1	PTGIR	0.1669
DUT	PLS3	PTGIR	0.1669
OTUB1	DLL1	DPYSL3	0.1669
MMP1	ODC1	PTGIR	0.1669
MAPK14	DLL1	PTGIR	0.1670
MMP1	PTGIR	REL	0.1670
MMP1	PTGIR	SPDEF	0.1670
OTUB1	LCT	PTGIR	0.1670
DLL1	PTGIR	SLC25A28	0.1670
EPHB6	PTGFRN	KCTD12	0.1670
KCTD12	NOS2	PECAM1	0.1670
GJC1	COX4I1	SLC39A4	0.1670
DPP4	VDR	MAPK13	0.1671
OTUB1	GCM1	PECAM1	0.1671
MMP1	NR2C2	PTGIR	0.1671
DLL1	GCM1	KCTD12	0.1671
ISL1	GCM1	MMP1	0.1672
ELP3	FOXA1	KCTD12	0.1672
CYP4F3	HOXD8	PTGIR	0.1672
IL36RN	ITGB2	PTGIR	0.1672
PTGFRN	SLC39A5	KCTD12	0.1673
ACSL4	FOXA1	KCTD12	0.1673
FOXJ2	MMP1	PLD3	0.1673
DPP4	MAPK13	TFAP2C	0.1673
REG1A	FOXA1	KCTD12	0.1673
CDK2AP1	MMP1	PTGIR	0.1673
ID3	MMP1	PTGIR	0.1673
FOXK2	PTGIR	TRAFD1	0.1674
PLS3	AQP7	PTGIR	0.1674
CYP4F3	GSTA1	PTGIR	0.1674
KCTD12	MAPK13	TAT	0.1674
IGF1R	MMP1	PTGIR	0.1674
PLS3	RBP4	PTGIR	0.1674
BCL2	KCTD12	NOS2	0.1674
PTGFRN	KCTD12	SLC28A1	0.1674
AQP11	DPP4	ELP3	0.1674
SLFN5	CYP4F3	PTGIR	0.1675
DPP4	DEFA6	SLC39A4	0.1675
FOXA1	GRB7	KCTD12	0.1675
FOXA1	KCTD12	SPDEF	0.1675
BCL2	CA14	KCTD12	0.1675
FPR1	DLL1	PTGIR	0.1675

Table S11. Top 500 low-ENL Single-Gene LDA Classifiers. The top 500 single-gene linear discriminant analysis classifiers and their respective misclassification rate in flaxseed lignan supplement vs placebo treatment in low-ENL excreters.

Gene	bresub error	Gene	bresub error	Gene	bresub error	Gene	bresub error
ANXA3	0.2454	PLA2G4C	0.2966	FOXP2	0.3194	SAT2	0.3333
NR6A1	0.2498	CHI3L1	0.2968	PKM	0.3201	TTN	0.3334
KCTD12	0.2521	FOXO3	0.2975	RUNX2	0.3203	RAD52	0.3335
EPHB1	0.2557	SLC3A1	0.2984	FOXK2	0.3204	REG1B	0.3341
PRKCH	0.2573	HES1	0.2992	ZNF365	0.3209	SLC17A5	0.3342
GCM1	0.2597	HMGCS1	0.3021	ADAMTS4	0.3222	ID2	0.3345
PTGIR	0.2626	IL1R2	0.304	SLC28A1	0.3224	ANXA5	0.3347
PROX1	0.2662	SLC5A8	0.3041	SORD	0.3225	TIMP3	0.3347
DLL1	0.2681	PCNA	0.3044	TRAFD1	0.3231	SHH	0.3352
PAX6	0.2687	IL18	0.3079	SLC35A2	0.3232	SOX4	0.3353
PARD3	0.2719	CDCP1	0.3086	HSD17B2	0.3234	CAT	0.3355
F11R	0.2732	SLC20A1	0.3093	PDE6D	0.324	BMI1	0.3357
PPAP2C	0.2732	PRKAR1A	0.3097	WNT5A	0.3244	REL	0.3359
ELP3	0.2801	HDAC1	0.3115	HOXD8	0.3247	GCLC	0.3375
NIFK	0.2802	TNFRSF10A	0.3116	TNF	0.3257	DDX5	0.339
RHOB	0.2808	TFAP2A	0.3117	HOXB8	0.3262	CCDC80	0.3394
SLC50A1	0.2815	BCL2	0.3121	FGFBP1	0.3262	ALDH1A1	0.3398
NOS2	0.2828	MSI2	0.3122	SLC7A6	0.3266	DAPK3	0.3401
PTGFRN	0.2852	CTDSPL	0.3126	SREBF2	0.3266	FAS	0.3403
CD44	0.2869	TFRC	0.3126	VDR	0.3271	SLC40A1	0.3403
DFFA	0.2869	TDG	0.3127	SPDEF	0.3271	SLC30A5	0.3407
EIF6	0.2881	GRB7	0.3129	HMGB1	0.3274	PLIN2	0.3411
SLC6A14	0.2888	HRH1	0.3131	HIST3H3	0.3284	PTPN11	0.3414
MAPK8	0.2889	TNK2	0.3133	ILF3	0.3284	PRSS23	0.3415
PTGES	0.2909	ACAT1	0.3135	SLCO2A1	0.3286	HOXA10	0.3429
CHAC1	0.2919	SLC39A4	0.3135	MMP1	0.3287	PLCD1	0.3436
DPP4	0.292	CDK15	0.3155	BECN1	0.329	PPAP2B	0.344
SLC9A2	0.2935	CYB5R1	0.3159	PLS3	0.3292	FDFT1	0.3446
BMP4	0.294	CDCA7	0.3173	ABCC1	0.3308	KEAP1	0.3447
REG1A	0.2946	KLF4	0.3177	MUC4	0.3312	ADAM9	0.3448
LEF1	0.2959	CD36	0.3184	SLC4A4	0.3315	CASP3	0.3448
EFNB1	0.2963	TFAP2C	0.3184	BMPR2	0.332	DUOX2	0.3449
TJP3	0.2963	GREM1	0.3186	FN1	0.3325	HMOX1	0.345
COX4I1	0.2964	MPC2	0.3186	PTCH1	0.3332	LCT	0.3463

Gene	bresub error	Gene	bresub error	Gene	bresub error	Gene	bresub error
BIRC5	0.3468	DEFA5	0.3599	PRKCI	0.3729	SLC30A7	0.3872
SLC7A7	0.347	CA5B	0.3603	SPRY2	0.3729	DHFR	0.3873
IL13RA1	0.3478	PRKACB	0.3606	TP53	0.3738	ADRB1	0.3876
HIF3A	0.3484	BRD2	0.3607	NTS	0.3746	STK11	0.3876
HNRNPA1	0.3486	APOA1	0.3611	KIT	0.3762	MSH2	0.3879
PPM1D	0.3487	RASSF1	0.3613	MAML1	0.3762	PROM2	0.388
SPPL3	0.3489	LRIG1	0.3614	CFLAR	0.3767	GADD45A	0.3887
SLC39A9	0.3489	FAM129A	0.3616	SLC2A2	0.3768	NOTCH3	0.3887
EPHB4	0.3493	COX5B	0.3619	PPARD	0.3772	EGF	0.3896
COL1A1	0.3499	FOXA1	0.3638	BST1	0.3775	CDK7	0.3908
IGF2BP1	0.3505	TSC22D3	0.3641	FGFR1	0.3775	EPHB6	0.3915
STAT3	0.351	SLC39A7	0.3643	DUSP1	0.3794	SLC16A9	0.3918
GSTM3	0.3515	ECM1	0.3654	LATS2	0.3802	SLC51A	0.3921
CAMK2D	0.3517	MUC2	0.3658	XRCC6	0.3806	CLDN8	0.3924
CYP4F3	0.3517	DPYSL3	0.3659	BCL10	0.381	HOXD10	0.3925
NR3C1	0.352	CDK8	0.3671	LIG3	0.3816	TPT1	0.3926
CEACAM19	0.3529	ITLN1	0.3672	FGF18	0.3819	ZDHHC20	0.3926
PTPN22	0.3533	TAT	0.3673	SLC35D1	0.3826	KLK1	0.393
BAK1	0.3534	PDE4C	0.3674	YWHAH	0.3829	AXIN2	0.393
PLA2G4D	0.3535	FGFR3	0.3675	KITLG	0.3829	POLG	0.3932
CCL15	0.3539	MYB	0.368	GPX1	0.3833	NEUROD1	0.3934
ALPI	0.3546	BMP2	0.3681	RUNX3	0.3834	DOT1L	0.3937
AGPAT3	0.3551	LYPLA1	0.3685	TJP1	0.3835	FOXJ3	0.3937
WNT7A	0.3561	ADAM10	0.3686	HNF4G	0.3836	SLC22A23	0.3938
CA7	0.3562	BCL2A1	0.3687	LAMA1	0.3838	ACKR2	0.394
DEFA6	0.3565	CHP1	0.3689	IFNAR1	0.3846	TYRO3	0.394
AIFM2	0.3572	SLC9A8	0.3695	GADD45B	0.3847	SERPINB2	0.3941
DDX11	0.3576	ARRB2	0.3698	GABRB3	0.385	PLA2G10	0.3944
HOXB7	0.3577	MAPK7	0.3698	SLFN5	0.3852	FOXA3	0.3944
CAV1	0.3586	PLAU	0.3699	PTGS2	0.3855	ABCG2	0.3949
ABCG5	0.3593	COLEC12	0.3701	TNFRSF10B	0.3856	MMP9	0.395
SLC38A2	0.3593	MELK	0.3701	DDR2	0.3858	EPAS1	0.3959
VIL1	0.3594	CXCL16	0.3703	FOXJ2	0.3864	FFAR2	0.396
CA4	0.3595	SLC35B3	0.3709	BACH1	0.3865	AATF	0.3963
OTUB1	0.3595	HLTF	0.3711	IL6ST	0.3865	GEMIN6	0.3966
ITGB2	0.3597	PIWIL2	0.3723	HMGCR	0.387	BTD	0.3969
SPI1	0.3598	SCD	0.3726	SELL	0.3871	HOXC6	0.3969

Gene	bresub error	Gene	bresub error	Gene	bresub error	Gene	bresub error
BCL2L11	0.397	CD59	0.4099	FASN	0.4189	BMF	0.4275
ERBB4	0.397	SLC5A11	0.41	NFIL3	0.4193	MUC3A	0.4279
APC	0.3972	HHIP	0.4101	SP1	0.4197	SCP2	0.4283
NCAM1	0.3974	P4HB	0.4109	NFKB2	0.4199	CACNA1C	0.4284
HDAC2	0.3976	RELA	0.4113	TNFRSF21	0.4202	ATG16L1	0.429
TFCP2L1	0.398	REG4	0.4115	TLR4	0.4204	CA13	0.4291
PLD3	0.3981	AXIN1	0.4118	JAK1	0.4205	CASP4	0.4291
OAT	0.3987	PLAUR	0.4122	ANXA1	0.4212	GCLM	0.4293
MAML3	0.3988	CDX2	0.4123	LRRK2	0.4213	FLT1	0.4296
GDF15	0.3994	MLLT4	0.4125	MAPK10	0.4213	NOG	0.4296
NPAT	0.3999	GJB2	0.4126	SPIB	0.4214	SIRT1	0.4298
HOXD13	0.4002	CDK2	0.4127	FABP6	0.4215	TNFRSF1B	0.431
RBL2	0.4006	CTNNA1	0.4129	YWHAE	0.422	CEACAM7	0.4312
PARK7	0.4013	S100A8	0.4132	POLB	0.4222	NUMB	0.4315
SST	0.4017	ILKAP	0.4133	WNT3	0.4224	CFTR	0.4316
SLC9A3R1	0.4017	TNFSF13B	0.4133	ILF2	0.4228	SLC31A1	0.4318
TLR2	0.4025	COL6A3	0.4135	SLC37A4	0.4228	SFN	0.432
SLC1A1	0.4029	BAD	0.4136	DNAJB6	0.423	YAP1	0.432
MEF2C	0.4033	FOXO1	0.4147	ALCAM	0.4233	SLC30A6	0.4321
SPP1	0.4039	ODC1	0.4148	LIFR	0.4234	CDKAL1	0.4325
SLC35A3	0.4048	LGR4	0.4155	NR2C2	0.4237	SLC16A1	0.4334
HPGD	0.4048	NRCAM	0.4157	WNT4	0.4238	PRKCA	0.4348
SLC30A4	0.4051	PRKCD	0.4157	MEP1A	0.4244	MAB21L2	0.4349
SLC25A33	0.4056	ACSL4	0.4159	SLC16A4	0.4248	LAMP1	0.4352
SLC39A5	0.4065	BAX	0.4159	PODXL	0.4249	CA3	0.4353
TNFRSF1A	0.4068	APEX1	0.4164	NRIP1	0.425	MAPK13	0.4355
TPM3	0.4072	HAS3	0.4164	YWHAZ	0.4256	ATG5	0.4358
SLC38A1	0.4074	DCN	0.4166	ACADS	0.4257	CD9	0.4359
PECAM1	0.4077	POU2F3	0.4167	SLC23A3	0.4257	GSN	0.436
PYGO2	0.4079	BBC3	0.4169	MSH3	0.4258	MYO9B	0.4366
REG3A	0.408	SLC7A9	0.4169	SOD1	0.4258	VIM	0.4368
MGAT3	0.4082	NOTCH4	0.4175	FTH1	0.426	CA14	0.4368
MALL	0.4084	AQP11	0.4178	CDH5	0.4261	RELB	0.4374
GJC1	0.4086	HMGCS2	0.4178	PRKDC	0.4263	LGALS3	0.4377
ANGPTL4	0.4089	PDK1	0.4181	WNT2B	0.4264	THRA	0.4385
RBP4	0.4091	C11orf30	0.4186	SLC3A2	0.4269	ATF4	0.4401
MPC1	0.4091	IL22RA1	0.4188	MAP2K2	0.4271	APOB	0.441

Gene	bresub error	Gene	bresub error	Gene	bresub error	Gene	bresub error
PLAA	0.441	RPS4X	0.4452	FOXP1	0.4494	AMPD3	0.4542
PLA2G7	0.4416	SLC25A20	0.4452	HOXB6	0.4499	HCAR2	0.4545
YY1	0.4416	TNFSF10	0.4454	PRKCB	0.4502	JUN	0.4545
KDM4C	0.4419	DUT	0.4455	PLCB3	0.4507	SDC1	0.4545
NOTCH1	0.4424	SLC34A2	0.4457	IL2RG	0.4509	BCL2L1	0.4546
PTK2B	0.4427	SEPTIN4	0.4457	NANOG	0.4509	PCDH7	0.4548
SLC28A2	0.4429	TLR3	0.4462	PTPN6	0.4513	SLC4A7	0.4549
FZD7	0.4431	MACC1	0.4463	KCNH2	0.4514	TGFA	0.4549
RCN3	0.4434	CDK2AP1	0.4463	MEIS1	0.4515	XIST	0.455
NEU3	0.4438	SYP	0.447	CCL28	0.4517	TNFAIP3	0.455
BCL2L2	0.4441	CA12	0.4473	TSC2	0.4517	EP400	0.4553
RBL1	0.4442	CKS2	0.4476	PPARGC1A	0.4519	MXI1	0.4553
CKAP4	0.4442	SLC25A13	0.4478	HOXB3	0.452	ID3	0.4555
YWHAB	0.4443	TLR5	0.4482	SOD2	0.4521	CA1	0.4557
LPAR4	0.4446	FOS	0.4489	CXCL2	0.4524	NOS1	0.4558
S100A9	0.4448	FLNB	0.4491	ERBB2	0.4532	GNL1	0.456
HSPD1	0.4449	SLC5A9	0.4492	HOXD1	0.4539	PTPRD	0.4562

Table S12. Top 500 low-ENL Two-Gene LDA Classifiers. The top 500 two-gene linear discriminant analysis classifiers and their respective misclassification rate in flaxseed lignan supplement vs placebo treatment in low-ENL excreters.

Gene 1	Gene 2	bresub error
ANGPTL4	RELA	0.1702
SEPTIN4	TSTA3	0.1773
ISL1	TSTA3	0.1799
ANGPTL4	GJB1	0.1803
NFKB1	TRAFD1	0.1824
NFKB1	TSTA3	0.1835
ANGPTL4	TSTA3	0.1861
PECAM1	TSTA3	0.1862
BCL2	ANGPTL4	0.1871
NFKB1	PROX1	0.1906
SEPTIN4	TLR2	0.1921
GJB1	SEPTIN4	0.1925
GJC1	TSTA3	0.1927
ACTA2	ANGPTL4	0.1928
POR	ANGPTL4	0.1929
COL1A1	NFKB1	0.1930
LEF1	NFKB1	0.1933
POR	NFKB1	0.1936
NFKB1	TIMP3	0.1937
ANGPTL4	SLC28A1	0.1939
NFKB1	SEPTIN4	0.1939
NFKB1	RAD52	0.1939
POR	PECAM1	0.1949
PROX1	TSTA3	0.1951
FGFR1	TRAFD1	0.1952
POR	SEPTIN4	0.1955
TRAFD1	TSTA3	0.1959
EPHB4	NFKB1	0.1960
BCL2	GJC1	0.1963
NFKB2	ANGPTL4	0.1965
ITGAM	TSTA3	0.1966
ISL1	POR	0.1970
BMP4	ANGPTL4	0.1971
FGFR1	PECAM1	0.1971
ALPI	IER3	0.1973
BMP4	NFKB1	0.1980

Gene 1	Gene 2	bresub error
GJC1	POR	0.1982
GJB1	NFKB1	0.1983
KCNH2	TSTA3	0.1983
TIMP3	TRAFD1	0.1991
SLC5A11	ANGPTL4	0.1992
COL1A2	PECAM1	0.1994
ANGPTL4	TRAFD1	0.1994
NOS1	NFKB1	0.1996
ISL1	GJB1	0.1998
ANGPTL4	SEPTIN4	0.1999
ANGPTL4	NFKB1	0.2000
PRKACA	NFKB1	0.2000
CACNA1C	NFKB1	0.2006
DOT1L	NFKB1	0.2006
BTD	ANGPTL4	0.2007
RELA	TLR2	0.2007
NOTCH3	TSTA3	0.2008
SEPTIN4	TIMP3	0.2008
IL1R1	CDCP1	0.2008
NFKB1	REG1B	0.2010
BRIX1	TIMP3	0.2011
NOD2	TSTA3	0.2013
BMP4	TSTA3	0.2014
PECAM1	SEPTIN4	0.2014
TLR2	TRAFD1	0.2014
IL1R1	NFKB1	0.2014
FGFR1	TSTA3	0.2015
ISL1	NFKB2	0.2016
ALPI	SPDEF	0.2020
ISL1	SLC5A11	0.2020
ISL1	TRAFD1	0.2021
NFKB1	PECAM1	0.2023
ITGAM	POR	0.2024
ANGPTL4	MAPK7	0.2025
BMP4	NFKB2	0.2025
GADD45B	COL1A2	0.2025

Gene 1	Gene 2	bresub error
PTGFRN	ANGPTL4	0.2026
PRKACA	ANGPTL4	0.2026
CDCP1	PTGES	0.2027
ANGPTL4	RAD52	0.2027
SST	ALPI	0.2030
HOXA2	NFKB1	0.2030
ANGPTL4	DPYSL3	0.2030
ISL1	TLR5	0.2033
COL1A1	JAG1	0.2034
CACNA1C	SEPTIN4	0.2034
PLCB4	TSTA3	0.2035
NOD2	RELA	0.2035
ITGAM	KCNH2	0.2038
HOXB6	ANGPTL4	0.2039
GEMIN6	TSTA3	0.2039
LEF1	TIMP3	0.2039
RASSF1	IER3	0.2039
BRIX1	NFKB1	0.2041
ISL1	COL1A2	0.2041
COL1A2	TSTA3	0.2042
ANGPTL4	COL1A2	0.2043
NFKB1	SLC1A4	0.2043
FOXD2	KCTD17	0.2043
IL4R	KCTD17	0.2043
ABCG5	JAG1	0.2044
JAG1	PTGES	0.2045
ANGPTL4	TLR5	0.2045
NIFK	TSTA3	0.2045
POR	COL1A2	0.2046
CDC25B	IER3	0.2048
TGFB2	IER3	0.2048
SST	SPDEF	0.2051
PROX1	TLR2	0.2053
BMP4	SLC5A11	0.2053
CDCP1	JAG1	0.2053
BMP4	TLR5	0.2054
ITGAM	SLC5A11	0.2055
BMP4	ISL1	0.2055

Gene 1	Gene 2	bresub error
ANGPTL4	DCN	0.2056
COL1A2	NFKB1	0.2056
NFKB1	NR2C2	0.2058
CDK8	KCTD17	0.2062
ISL1	SLC5A9	0.2064
BMP4	PECAM1	0.2065
GADD45B	CDCP1	0.2066
DCN	KCNH2	0.2066
BECN1	JAG1	0.2066
NFKB1	WNT5A	0.2066
NR6A1	TSTA3	0.2067
SLC5A9	ANGPTL4	0.2067
GJC1	ACTA2	0.2068
ALPI	ULK1	0.2070
CHI3L1	NFKB1	0.2072
SEPTIN4	WNT5A	0.2072
ISL1	PTGFRN	0.2072
ISL1	ANGPTL4	0.2074
ALPI	KCTD17	0.2075
JAG1	LRRK2	0.2075
GJB1	NANOG	0.2076
COL1A2	PROX1	0.2077
BMF	HOXA10	0.2077
NEU3	NFKB1	0.2077
POR	PROX1	0.2078
ISL1	PECAM1	0.2079
CASP1	TSC22D3	0.2079
PECAM1	TLR2	0.2079
ANGPTL4	FN1	0.2080
KCTD17	SPDEF	0.2081
ITGAM	ANGPTL4	0.2082
HOXA10	TTN	0.2082
ANGPTL4	PECAM1	0.2083
COL1A1	TLR2	0.2085
COL1A1	TSTA3	0.2085
BRIX1	TSTA3	0.2085
IER3	IL2RB	0.2089
ISL1	TLR2	0.2089

Gene 1	Gene 2	bresub error
ANGPTL4	GRB7	0.2089
ITGB2	IER3	0.2089
PTK7	TSTA3	0.2091
COL1A2	GJB1	0.2091
SLC6A6	HOXA10	0.2091
IL1R1	JAG1	0.2091
COL1A2	TRAFD1	0.2093
SLC34A2	ANGPTL4	0.2094
TSTA3	WNT5A	0.2094
ANGPTL4	MALL	0.2095
CACNA1C	ISL1	0.2097
SLC5A11	DCN	0.2097
SLC39A5	ANGPTL4	0.2097
TLR2	TSTA3	0.2097
CEACAM3	TSTA3	0.2098
FGFR1	ANGPTL4	0.2098
DDX11	TSTA3	0.2099
BMP4	TRAFD1	0.2099
APC	ANGPTL4	0.2099
NANOG	TRAFD1	0.2100
GJC1	GJB1	0.2100
PTPN2	JAG1	0.2100
CDCP1	CHI3L1	0.2100
MEN1	TSTA3	0.2101
CDK15	IER3	0.2102
HOXA10	IGFBP3	0.2102
NFKB1	TLR2	0.2102
ISL1	KCNH2	0.2103
ALPI	MYO9B	0.2104
WNT4	TSTA3	0.2104
NFKB1	TNFRSF10A	0.2104
ISL1	DPYSL3	0.2105
WNT4	NFKB1	0.2105
DDR2	TSTA3	0.2106
SLC39A8	IER3	0.2107
NANOG	TSTA3	0.2107
TLR5	TSTA3	0.2107
GADD45B	CHI3L1	0.2108

Gene 1	Gene 2	bresub error
BMP4	KCNH2	0.2108
TGM2	ANGPTL4	0.2108
PECAM1	RELA	0.2110
SST	TGFB2	0.2110
NANOG	TLR2	0.2111
COL1A2	TLR3	0.2112
PROX1	SEPTIN4	0.2112
GJC1	KCNH2	0.2114
BMP4	ITGAM	0.2117
AQP7	IER3	0.2118
FGFR1	POR	0.2118
HOXA10	MAML1	0.2118
BRIX1	NANOG	0.2119
GJC1	RELA	0.2121
JAG1	SYP	0.2121
DDR2	NANOG	0.2122
ANGPTL4	NANOG	0.2123
NANOG	SIRT1	0.2123
PLA2G10	NR5A2	0.2123
TLR2	WNT5A	0.2123
GJB1	PECAM1	0.2124
GADD45B	JAG1	0.2124
ITGAM	GJB1	0.2124
BMP4	GJC1	0.2125
FOXK2	KCTD17	0.2125
CACNA1C	LEF1	0.2125
ANGPTL4	KCNH2	0.2126
HOXA10	SERPINA1	0.2126
COL1A1	LEF1	0.2126
ANGPTL4	TIMP3	0.2126
DDR2	NFKB1	0.2126
BMF	IER3	0.2126
HOXA10	ULK1	0.2127
NANOG	TIMP3	0.2128
ANGPTL4	TLR2	0.2128
LEF1	TLR2	0.2129
HOXA10	SHH	0.2129
MEIS1	TLR2	0.2129

Gene 1	Gene 2	bresub error
TGM2	TSTA3	0.2130
CDK15	HOXA10	0.2130
PLA2G10	HES1	0.2131
ANGPTL4	MLH1	0.2131
SST	HOXA13	0.2131
MSI1	IER3	0.2131
SLC5A11	TSTA3	0.2132
ITGAM	NFKB2	0.2132
BMP4	BRIX1	0.2133
DCN	TSTA3	0.2133
ITGAM	SEPTIN4	0.2133
COL1A1	PROX1	0.2134
HOXA10	HOXA13	0.2134
ANGPTL4	BRIX1	0.2134
HOXA10	XRCC6	0.2134
ALPI	CHAC1	0.2137
BMP4	NOTCH3	0.2137
GADD45B	IL1R1	0.2137
GADD45B	MEIS1	0.2138
NFKB2	NANOG	0.2138
HES1	MYO9B	0.2139
BRIX1	TLR2	0.2139
MSI1	KCTD17	0.2139
CACNA1C	GADD45B	0.2140
GJC1	TRAFD1	0.2140
LEF1	ANGPTL4	0.2140
SST	CDC25B	0.2141
GJC1	NFKB2	0.2141
GJC1	MAPK7	0.2141
CA7	TJP1	0.2141
SLC39A8	HOXA10	0.2142
SCP2	TSC22D3	0.2143
DDR2	TIMP3	0.2143
HOXA10	LCT	0.2144
BMP4	DPYSL3	0.2144
JAG1	P2RX4	0.2144
GJC1	PLCB4	0.2144
COL1A1	GADD45B	0.2145

Gene 1	Gene 2	bresub error
PECAM1	TIMP3	0.2146
ITGAM	NANOG	0.2146
NANOG	REG1B	0.2147
ANGPTL4	PROX1	0.2147
ALPI	SLCO2A1	0.2147
PTGFRN	NANOG	0.2148
LATS2	COL1A2	0.2148
CACNA1C	ANGPTL4	0.2148
ISL1	BRIX1	0.2148
ISL1	TIMP3	0.2148
IER3	LCT	0.2148
NOS1	TLR2	0.2149
ITGAM	TRAFD1	0.2149
GJC1	DCN	0.2149
PRKCH	NFKB1	0.2150
GADD45B	P2RX4	0.2151
BMP4	DCN	0.2151
MYO9B	TJP1	0.2152
ISL1	NFKB1	0.2152
HRAS	MYO9B	0.2152
FGFR1	COL1A2	0.2153
HOXA10	KCTD17	0.2153
PLA2G10	HOXA10	0.2153
POR	TRAFD1	0.2154
ANGPTL4	CGNL1	0.2154
IL4R	NOS2	0.2155
COL1A2	TLR2	0.2155
CA7	HES1	0.2155
HOXD10	KCTD17	0.2155
ALPI	HOXA10	0.2155
AQP7	HOXA10	0.2156
GADD45B	PDZK1IP1	0.2156
NFKB2	TSTA3	0.2156
EPHB4	TSTA3	0.2158
ANGPTL4	SIRT1	0.2158
NANOG	TLR5	0.2158
BMP4	NANOG	0.2158
GUCA2B	HOXA10	0.2158

Gene 1	Gene 2	bresub error
CACNA1C	NANOG	0.2159
SLC34A2	TSTA3	0.2159
POR	KCNH2	0.2159
GJC1	SLC5A9	0.2159
TGFB2	CA7	0.2159
MEIS1	TSTA3	0.2160
PLA2G10	SLC2A13	0.2160
ITGAM	NFKB1	0.2161
CA13	HOXA10	0.2161
CD44	HOXA10	0.2161
FAM129A	TSTA3	0.2161
GPX2	HOXA10	0.2162
ATF4	CA7	0.2162
MAML1	SREBF2	0.2164
CACNA1C	COL1A1	0.2164
MEIS1	NFKB1	0.2164
SIRT1	TIMP3	0.2165
CGNL1	TSTA3	0.2165
HRAS	ULK1	0.2166
DAPK1	COL1A2	0.2167
ALPI	FOXK2	0.2167
CDCP1	LRRK2	0.2168
ITGAM	NOTCH3	0.2168
PTK7	TLR2	0.2168
ILF2	COL1A2	0.2169
CHI3L1	NANOG	0.2169
FN1	TSTA3	0.2170
TGFBR2	JAG1	0.2170
PECAM1	RAD52	0.2170
BMP4	GJB1	0.2171
RAD52	TLR2	0.2171
IER3	NOTCH1	0.2171
HRAS	IER3	0.2172
DPYSL3	NANOG	0.2172
SPDEF	ULK1	0.2172
MAML1	SPDEF	0.2172
CDH3	HOXA10	0.2172
JAG1	PDZK1IP1	0.2173

Gene 1	Gene 2	bresub error
RELA	TSTA3	0.2173
COL1A1	TLR3	0.2173
ITGAM	COL1A2	0.2174
KCNH2	NANOG	0.2174
HOXA10	TNK2	0.2174
IER3	PTGIR	0.2174
SLC10A2	ALPI	0.2175
IER3	PLIN3	0.2175
CA7	PRKCI	0.2175
ISL1	ITGAM	0.2175
ANXA5	HOXA10	0.2175
FFAR4	HOXA10	0.2175
ANGPTL4	WNT5A	0.2176
CDCP1	NFKB1	0.2176
DDIT4	TSTA3	0.2176
SST	IL2RB	0.2176
TGFB2	HOXA10	0.2176
CACNA1C	DCN	0.2176
COL1A1	LATS2	0.2177
ALPI	MAML1	0.2177
GSTA4	JAG1	0.2178
BRIX1	DCN	0.2178
SLC39A8	SST	0.2178
CA1	CDCP1	0.2178
SLC5A11	NANOG	0.2178
ADAM10	CA7	0.2179
ISL1	RAD52	0.2179
CHAC1	HOXA10	0.2179
CACNA1C	COL1A2	0.2179
AQP7	SLCO2A1	0.2179
FOXP4	TSTA3	0.2179
LPAR1	SPDEF	0.2180
IER3	PTPN22	0.2180
GADD45B	TGFBR2	0.2180
CACNA1C	DDR2	0.2181
ISL1	NANOG	0.2181
NANOG	NR6A1	0.2182
ILF2	TLR2	0.2182

Gene 1	Gene 2	bresub error
POR	TSTA3	0.2182
HRAS	HOXA10	0.2183
MYO9B	SREBF2	0.2183
ADAMTS4	COL1A2	0.2183
ITGAM	PTGFRN	0.2183
GJC1	SEPTIN4	0.2184
COL1A1	ILF2	0.2184
ANGPTL4	EPHB4	0.2184
NFKB2	KCNH2	0.2184
MAML1	XRCC6	0.2184
PLS3	JAG1	0.2184
HOXA13	PRKCI	0.2184
CA7	CD44	0.2185
CA3	IER3	0.2185
MAML1	SHH	0.2185
CHI3L1	JAG1	0.2185
ATF4	HOXA13	0.2185
IL18	TJP1	0.2185
MYC	ANGPTL4	0.2185
ITGAM	TLR5	0.2186
DCN	TIMP3	0.2186
MALL	TSTA3	0.2186
FOXA1	JAG1	0.2187
ANGPTL4	DDR2	0.2187
DPYSL3	KCNH2	0.2188
CASP4	JAG1	0.2188
ADAM10	MYO9B	0.2188
BMF	FGF5	0.2188
CACNA1C	PECAM1	0.2188
ANGPTL4	NOTCH3	0.2188
BMF	PRKCI	0.2189
ATF4	ULK1	0.2189
PRNP	ANGPTL4	0.2189
JAG1	TSC22D3	0.2189
IER3	SPDEF	0.2189
GJC1	SLC5A11	0.2189
ABCG5	COL1A2	0.2190
ALPI	FOXD2	0.2191

Gene 1	Gene 2	bresub error
IER3	TNFRSF10D	0.2191
ITGAM	SLC5A9	0.2191
HOXA13	SREBF2	0.2191
ITGB2	IL2RB	0.2191
CA7	CXCL2	0.2191
SST	PLIN3	0.2192
TGFB2	IL4R	0.2192
CA3	HOXA10	0.2193
NFKB1	SLC26A6	0.2193
GJC1	PTGFRN	0.2193
BMF	ATF4	0.2193
PRKACA	TSTA3	0.2194
HRAS	SLCO2A1	0.2194
PRKCI	ULK1	0.2194
COL1A2	FN1	0.2194
MSI1	HOXA10	0.2194
HOXA13	XRCC6	0.2194
GJC1	ANGPTL4	0.2195
ISL1	NOTCH3	0.2195
NOS1	TSTA3	0.2195
FGFR1	GJC1	0.2196
TGFB2	SLCO2A1	0.2196
HOXA13	SHH	0.2196
SLC10A2	SPDEF	0.2197
FGF5	ULK1	0.2197
MSI1	SLC2A13	0.2197
HAS2	HOXA10	0.2197
HRAS	SST	0.2197
APOB	JAG1	0.2197
CA7	SREBF2	0.2198
IER3	IL4R	0.2198
NFKB2	COL1A2	0.2199
BMF	XRCC6	0.2199
ALPI	IL4R	0.2199
PTGFRN	HOXA10	0.2200
ULK1	XRCC6	0.2200
SHH	ULK1	0.2200
DDX11	NANOG	0.2200

Gene 1	Gene 2	bresub error
LEF1	TSTA3	0.2200
SIRT1	TSTA3	0.2200
MSI1	SLCO2A1	0.2201
ANGPTL4	DDX11	0.2201
HOXA10	SMAD3	0.2201
NOS1	ANGPTL4	0.2201
SLC9A2	HOXA10	0.2201
POR	GJB1	0.2201
CDK7	HOXA10	0.2202
HRAS	IL4R	0.2202
PLA2G10	FUT2	0.2202
RBP4	HOXA10	0.2202
COL1A1	SIRT1	0.2203
FGF5	HOXA10	0.2203
BMP4	SIRT1	0.2203
COL1A2	NANOG	0.2203
SLC39A5	HOXA10	0.2203
GADD45B	APOB	0.2203
KCTD17	XRCC6	0.2203
DAB2IP	IL23R	0.2203
MSI1	CA7	0.2204
TJP1	ULK1	0.2204
TP63	HOXA10	0.2204
HOXA10	SPDEF	0.2204

Gene 1	Gene 2	bresub error
ITGAL	HOXA10	0.2204
ACKR2	COL1A2	0.2204
SLC10A2	IER3	0.2204
HRAS	FOXK2	0.2204
CD44	HOXA13	0.2205
HOXA10	SLC16A4	0.2205
NPAT	HOXA10	0.2205
MSI1	SST	0.2205
CACNA1C	HOXA10	0.2206
FUT2	KCTD17	0.2206
PLA2G10	REST	0.2206
HOXA10	PLIN3	0.2206
GJC1	NANOG	0.2206
TGFBR2	COL1A2	0.2206
CD44	ULK1	0.2206
ATF4	TNK2	0.2206
SERPINB2	JAG1	0.2206
TGFB2	FOXD2	0.2207
TNK2	XRCC6	0.2207
CA7	SMAD3	0.2207
SLC2A13	XRCC6	0.2207
CASP1	CDCP1	0.2208
SST	TNFRSF10D	0.2208
SREBF2	ULK1	0.2208

Table S13. Top 500 low-ENL Three-Gene LDA Classifiers. The top 500 three-gene linear discriminant analysis classifiers and their respective misclassification rate in flaxseed lignan supplement vs placebo treatment in low-ENL excreters.

Gene 1	Gene 2	Gene 3	bresub error	Gene 1	Gene 2	Gene 3	bresub error
CDH3	SEPTIN4	TSTA3	0.1427	FN1	RELA	TSTA3	0.1609
PLA2G10	HOXA13	ULK1	0.1429	PLA2G10	FFAR4	MAML1	0.1612
PLA2G10	HOXA13	MAML1	0.1429	RBP4	SEPTIN4	TSTA3	0.1612
FAM129A	SEPTIN4	TSTA3	0.1450	ANGPTL4	SEPTIN4	TSTA3	0.1613
CACNB4	RELA	TSTA3	0.1465	DCN	RELA	TSTA3	0.1614
NANOG	RELA	TSTA3	0.1468	PLA2G10	HOXA13	REST	0.1614
CYP4F3	HOXA13	KCTD17	0.1523	POR	SEPTIN4	TSTA3	0.1615
POR	NFKB1	TRAFD1	0.1537	NOD2	ANGPTL4	RELA	0.1615
RELA	SEPTIN4	TSTA3	0.1537	POR	GJB1	SEPTIN4	0.1616
WNT4	RELA	TSTA3	0.1540	FAM129A	COL1A2	TLR3	0.1619
GPX2	PLA2G10	MAML1	0.1542	DEFA6	HOXA13	KCTD17	0.1619
PLA2G10	FOXD2	MAML1	0.1547	BMP4	ANGPTL4	RELA	0.1620
NFKB1	TRAFD1	TSTA3	0.1551	SLC5A1	SEPTIN4	TSTA3	0.1620
CDK8	PLA2G10	MAML1	0.1557	PLA2G10	PRKCE	ULK1	0.1620
FOXP4	SEPTIN4	TSTA3	0.1565	KDM4B	PLA2G10	MAML1	0.1621
PPAP2B	SEPTIN4	TSTA3	0.1570	CCND1	SEPTIN4	TSTA3	0.1624
NFKB2	SEPTIN4	TSTA3	0.1576	SEPTIN4	TNFRSF10A	TSTA3	0.1625
PLA2G10	HOXA13	NR5A2	0.1577	MSI1	PLA2G10	MAML1	0.1627
ANGPTL4	RELA	TSTA3	0.1580	ISL1	PECAM1	TSTA3	0.1627
RELA	TRAFD1	TSTA3	0.1581	FGFR1	TRAFD1	TSTA3	0.1627
PECAM1	SEPTIN4	TSTA3	0.1581	RELA	SLC16A4	TSTA3	0.1628
SEPTIN4	TRAFD1	TSTA3	0.1583	FOXP4	ANGPTL4	RELA	0.1631
PLA2G10	FOXD2	HOXA13	0.1584	PLA2G10	FOXK2	ULK1	0.1631
PLA2G10	CYP4F3	HOXA13	0.1587	PLA2G10	FOXD2	ULK1	0.1632
RELA	SLC1A4	TSTA3	0.1588	COL1A2	DDIT4	RPSA	0.1632
PLA2G10	MAML1	PRKCE	0.1590	APC	SEPTIN4	TSTA3	0.1632
PLA2G10	HOXA13	SLCO2A1	0.1591	IGF2BP1	RELA	TSTA3	0.1634
PTK7	RELA	TSTA3	0.1595	COL1A2	NFKB1	TLR3	0.1635
PLA2G10	CHAC1	HOXA13	0.1596	CDK8	PLA2G10	ULK1	0.1641
PLA2G10	FOXK2	MAML1	0.1596	ANGPTL4	KIT	RELA	0.1641
SLC34A2	SEPTIN4	TSTA3	0.1598	HOXA9	NFKB1	TSTA3	0.1643
ANGPTL4	IGF2BP1	RELA	0.1600	SLC34A2	RELA	TSTA3	0.1643
CACNA1C	DDIT4	RPSA	0.1600	SLC39A5	SEPTIN4	TSTA3	0.1644
PLA2G10	MAML1	NR5A2	0.1600	MSI1	HOXA13	KCTD17	0.1645
PLA2G10	HOXA13	PRKCE	0.1602	PPM1D	SEPTIN4	TSTA3	0.1645
CDK8	PLA2G10	HOXA13	0.1602	CACNA1C	NFKB1	TRAFD1	0.1646
GEMIN6	TRAFD1	TSTA3	0.1608	ANGPTL4	EPHB4	RELA	0.1648
PLA2G10	FOXK2	HOXA13	0.1609	CGNL1	RELA	TSTA3	0.1648

Gene 1	Gene 2	Gene 3	bresub error	Gene 1	Gene 2	Gene 3	bresub error
WNT4	ANGPTL4	RELA	0.1648	HOXD13	SEPTIN4	TSTA3	0.1673
HOXA9	NFKB1	TRAFD1	0.1649	EPHB4	NFKB1	TRAFD1	0.1673
PLA2G10	FOXO3	HOXA13	0.1650	IGF2BP1	SEPTIN4	TSTA3	0.1673
GPX2	PLA2G10	ULK1	0.1651	LPAR4	PECAM1	TSTA3	0.1674
KDM4B	PLA2G10	HOXA13	0.1651	KDM4B	PLA2G10	SLC2A13	0.1674
FOXK2	HOXA13	KCTD17	0.1653	FOXP4	RELA	TSTA3	0.1674
HOXA13	NFKB1	TSTA3	0.1653	NANOG	TRAFD1	TSTA3	0.1675
FOXO3	HOXA13	KCTD17	0.1654	PRKCH	SEPTIN4	TSTA3	0.1675
LEF1	NFKB1	TRAFD1	0.1657	ISL1	TRAFD1	TSTA3	0.1677
FAM129A	PROX1	TLR3	0.1657	MSI1	PLA2G10	HOXA13	0.1677
PLA2G10	PRKCE	SLC2A13	0.1657	MSH3	PECAM1	TSTA3	0.1677
SEPTIN4	TSC2	TSTA3	0.1657	FGFR1	POR	TRAFD1	0.1679
CDK7	SEPTIN4	TSTA3	0.1657	DDIT4	REL	RPSA	0.1679
POR	ANGPTL4	SEPTIN4	0.1658	CDH3	ISL1	TSTA3	0.1679
DDIT4	RPSA	TIMP3	0.1659	ANGPTL4	PECAM1	TSTA3	0.1680
PRKACA	RELA	TSTA3	0.1659	ISL1	ANGPTL4	TSTA3	0.1680
PLA2G10	HOXA13	HOXA9	0.1659	SEPTIN4	TSTA3	WNT5A	0.1682
ISL1	RELA	TSTA3	0.1661	PLA2G10	MAML1	PIK3CA	0.1683
NFKB2	RELA	TSTA3	0.1662	CDH3	PECAM1	TSTA3	0.1683
POR	GJB1	PECAM1	0.1662	POR	ANGPTL4	PECAM1	0.1684
SST	PTPN22	SPDEF	0.1662	PLA2G10	HOXA10	MAML1	0.1684
POR	PECAM1	SEPTIN4	0.1663	PLCB4	SEPTIN4	TSTA3	0.1684
ACTG2	SEPTIN4	TSTA3	0.1663	PLA2G10	HOXA13	TNK2	0.1684
PRKCH	NFKB1	TRAFD1	0.1663	FAM129A	TRAFD1	TSTA3	0.1685
ITGAL	SEPTIN4	TSTA3	0.1664	DDIT4	RPSA	TLR2	0.1685
NOS1	TRAFD1	TSTA3	0.1664	NR2C2	PECAM1	TSTA3	0.1685
FAM129A	DDIT4	TSTA3	0.1664	ALPI	IER3	SPDEF	0.1685
KDM4B	PLA2G10	ULK1	0.1664	GPX2	PLA2G10	SLC2A13	0.1686
PTK7	SEPTIN4	TSTA3	0.1665	COL1A2	NFKB1	TSTA3	0.1686
SEPTIN4	TFAP2C	TSTA3	0.1666	FGFR1	NANOG	TSTA3	0.1687
NOD2	GJB1	RELA	0.1667	CDH3	TRAFD1	TSTA3	0.1688
DOT1L	RELA	TSTA3	0.1668	HMGCL	SEPTIN4	TSTA3	0.1689
LPAR4	SEPTIN4	TSTA3	0.1668	ACTA2	ANGPTL4	RELA	0.1690
LEF1	NFKB1	TSTA3	0.1668	NFKB1	RAD52	TRAFD1	0.1690
FOXP4	ISL1	TSTA3	0.1669	SST	ALPI	SPDEF	0.1691
VNN1	HOXA13	KCTD17	0.1669	BMP4	NOD2	RELA	0.1691
NOS1	RELA	TSTA3	0.1670	FGFR1	POR	PECAM1	0.1691
MSI1	PLA2G10	ULK1	0.1670	RELA	TSTA3	WNT5A	0.1691
NOD2	RELA	WNT5A	0.1671	NFKB1	TLR2	TRAFD1	0.1691
BRIX1	RELA	TSTA3	0.1671	DDIT4	TRAFD1	TSTA3	0.1692
APC	PROX1	TSTA3	0.1672	FAM129A	PROX1	TSTA3	0.1693

Gene 1	Gene 2	Gene 3	bresub error	Gene 1	Gene 2	Gene 3	bresub error
HOXD13	ANGPTL4	RELA	0.1694	HOXB8	PECAM1	TSTA3	0.1714
IL1R2	SEPTIN4	TSTA3	0.1695	CDK8	PLA2G10	REST	0.1714
TREH	ANGPTL4	RELA	0.1695	PLA2G10	FFAR4	ULK1	0.1714
ISL1	WNT4	TSTA3	0.1695	APC	SLC6A20	TSTA3	0.1714
FAM129A	GEMIN6	TSTA3	0.1696	TP63	NFKB1	TSTA3	0.1714
APC	FGFR1	TSTA3	0.1696	ANGPTL4	MAPK7	RELA	0.1715
GPX2	PLA2G10	HOXA13	0.1697	PECAM1	TSTA3	WNT5A	0.1715
ANGPTL4	CACNB4	RELA	0.1697	POR	SEPTIN4	TRAFD1	0.1715
PDE4C	SEPTIN4	TSTA3	0.1698	PLA2G10	FFAR4	HOXA13	0.1715
APC	PPAP2B	TSTA3	0.1698	NOD2	SEPTIN4	TSTA3	0.1715
LEF1	DDIT4	RPSA	0.1698	POR	NFKB1	OGG1	0.1715
NFKB2	ANGPTL4	RELA	0.1699	FAM129A	WNT4	TSTA3	0.1716
NFKB1	TIMP3	TRAFD1	0.1699	WNT4	SEPTIN4	TSTA3	0.1716
FAM129A	TLR3	TSTA3	0.1700	COL1A2	CTDSPL	NFKB1	0.1716
PLA2G10	PRKCE	REST	0.1700	NFKB1	PROX1	TSTA3	0.1716
APC	NFKB1	TSTA3	0.1701	PTAFR	RELA	TSTA3	0.1717
SEPTIN4	TSTA3	ZBTB33	0.1701	NFKB1	PPAP2B	TSTA3	0.1717
GJB1	SEPTIN4	TFAP2C	0.1701	SLC10A2	SST	SPDEF	0.1718
PLA2G10	MYO9B	ULK1	0.1701	ISL1	POR	PECAM1	0.1718
NFKB1	RELA	TSTA3	0.1701	ANGPTL4	MLH1	RELA	0.1719
ITGB2	SST	SPDEF	0.1702	PTAFR	SEPTIN4	TSTA3	0.1719
BRIX1	NFKB1	TSTA3	0.1703	ANGPTL4	RELA	WNT5A	0.1720
PECAM1	RELA	TSTA3	0.1703	APC	PTK7	TSTA3	0.1720
BCL2	ANGPTL4	RELA	0.1703	NFKB1	NR2C2	TSTA3	0.1720
HOXA13	KCTD17	NOS2	0.1704	ANGPTL4	TRAFD1	TSTA3	0.1720
LEF1	RELA	TSTA3	0.1704	PLA2G10	FOXD2	REST	0.1720
ALPI	HOXA13	SPDEF	0.1704	CDK8	PLA2G10	SLC2A13	0.1721
NOS1	NFKB1	TRAFD1	0.1705	POR	NFKB1	TSTA3	0.1721
LPAR4	NFKB1	TSTA3	0.1705	NFKB1	REL	TRAFD1	0.1721
FAM129A	FGFR1	TSTA3	0.1705	ISL1	SEPTIN4	TSTA3	0.1721
GEMIN6	NFKB1	TSTA3	0.1706	ITGB2	IER3	SPDEF	0.1721
APC	ISL1	TSTA3	0.1706	ISL1	POR	ANGPTL4	0.1721
GJB1	SEPTIN4	TSTA3	0.1708	NOS1	NFKB1	TSTA3	0.1721
FAM129A	LEF1	TSTA3	0.1709	PLA2G10	MAML1	TP53	0.1721
ANGPTL4	MSH3	RELA	0.1709	NR2C2	RELA	TSTA3	0.1722
ITGB2	IER3	NR5A2	0.1709	FAM129A	PTK7	TSTA3	0.1723
POLB	SEPTIN4	TSTA3	0.1710	PTAFR	ANGPTL4	RELA	0.1723
NFKB1	OGG1	TSTA3	0.1710	FOXP4	POR	SEPTIN4	0.1723
PLA2G10	PIK3CA	ULK1	0.1710	ANGPTL4	RELA	TLR5	0.1723
FAM129A	POR	SEPTIN4	0.1711	HOXA13	IL4R	KCTD17	0.1724
PROX1	SEPTIN4	TSTA3	0.1712	GEMIN6	PTK7	TSTA3	0.1724

Gene 1	Gene 2	Gene 3	bresub error	Gene 1	Gene 2	Gene 3	bresub error
GRB7	NFKB1	TSTA3	0.1724	NFKB2	PECAM1	TSTA3	0.1737
PLA2G10	JAK2	MAML1	0.1724	NFKB1	SLC1A4	TSTA3	0.1737
ISL1	NR2C2	TSTA3	0.1725	PLA2G10	HOXA10	HOXA13	0.1738
POR	NR2C2	PECAM1	0.1725	PLA2G10	JAK2	ULK1	0.1738
SLC1A4	TRAFD1	TSTA3	0.1726	ITGAM	SEPTIN4	TSTA3	0.1738
NANOG	RELA	TLR2	0.1726	C11orf30	SEPTIN4	TSTA3	0.1738
NOS1	SEPTIN4	TSTA3	0.1726	FGFR1	PECAM1	TSTA3	0.1738
APC	ANGPTL4	TSTA3	0.1726	PLA2G10	JAK2	SLC2A13	0.1738
PECAM1	TLR5	TSTA3	0.1727	ISL1	POR	GJB1	0.1738
POR	CTDSPL	SEPTIN4	0.1727	FOXP4	PECAM1	TSTA3	0.1738
RELA	TLR2	TRAFD1	0.1728	ISL1	NOD2	TSTA3	0.1738
KDM4B	PLA2G10	REST	0.1728	SLC27A2	SEPTIN4	TSTA3	0.1738
PLA2G10	NR5A2	ULK1	0.1728	APC	TRAFD1	TSTA3	0.1738
CACNA2D1	TRAFD1	TSTA3	0.1729	FAM129A	NR2C2	TSTA3	0.1739
NFKB1	PTK7	TSTA3	0.1729	ISL1	PLCB4	TSTA3	0.1739
DDIT4	RPSA	TSTA3	0.1729	BST1	ANGPTL4	RELA	0.1740
FOXD2	HOXA13	KCTD17	0.1730	SLC31A1	NFKB1	TRAFD1	0.1740
ANGPTL4	DCN	RELA	0.1730	SEPTIN4	TLR2	TSTA3	0.1740
POR	WNT4	GJB1	0.1730	FOXP4	WNT4	TSTA3	0.1741
ISL1	NFKB2	TSTA3	0.1730	ISL1	MALL	TSTA3	0.1741
ANGPTL4	PECAM1	RELA	0.1730	FGFR1	POR	WNT4	0.1742
PLA2G10	MAML1	POU2F3	0.1731	ANGPTL4	FN1	RELA	0.1742
TGFB2	ALPI	SPDEF	0.1731	GEMIN6	SLC34A2	TSTA3	0.1742
BRIX1	NFKB1	TRAFD1	0.1731	COL1A2	RPSA	TLR3	0.1742
BRIX1	TRAFD1	TSTA3	0.1732	BTD	NFKB1	TSTA3	0.1742
HOXC6	ANGPTL4	RELA	0.1733	PLA2G10	BCL2L13	MAML1	0.1743
POR	CTDSPL	DDIT4	0.1733	COL1A2	NFKB1	TRAFD1	0.1743
PRKACA	NFKB1	TSTA3	0.1733	PTGFRN	NFKB1	TSTA3	0.1743
GJC1	ANGPTL4	RELA	0.1733	FAM129A	CDH5	TSTA3	0.1743
SAT2	PECAM1	TSTA3	0.1734	SLC31A1	SEPTIN4	TSTA3	0.1743
MALL	PECAM1	TSTA3	0.1734	HOXB8	PLCB4	TSTA3	0.1743
PLA2G10	FOXK2	REST	0.1735	PLA2G10	HOXA13	KCTD17	0.1744
GEMIN6	RELA	TSTA3	0.1735	POLB	NFKB1	TSTA3	0.1744
KCNH2	PECAM1	TSTA3	0.1735	ACSL4	SEPTIN4	TSTA3	0.1744
CACNA1C	CTDSPL	NFKB1	0.1735	GJB1	TNFRSF10A	SEPTIN4	0.1744
SLC6A20	TRAFD1	TSTA3	0.1736	WNT4	GJB1	RELA	0.1744
POR	COL1A2	NFKB1	0.1736	FGFR1	WNT4	TSTA3	0.1744
NFKB1	SLC16A4	TSTA3	0.1736	IER3	PTPN22	SPDEF	0.1744
LPAR1	CDC25B	SPDEF	0.1736	CACNA2D1	RELA	TSTA3	0.1744
FGFR1	RELA	TSTA3	0.1736	FAM129A	SLC1A4	TSTA3	0.1744
PLA2G10	CHAC1	PRKCE	0.1737	TGFB2	HOXA13	KCTD17	0.1745

Gene 1	Gene 2	Gene 3	bresub error	Gene 1	Gene 2	Gene 3	bresub error
HOXA13	KCTD17	MYO9B	0.1745	NANOG	SEPTIN4	TSTA3	0.1752
POR	CTDSPL	NFKB1	0.1745	PLA2G10	SLC2A13	SLC7A7	0.1752
CSPG5	ANGPTL4	RELA	0.1746	FGFR1	NOS1	TSTA3	0.1753
PLA2G10	TGFB2	HOXA13	0.1746	GJC1	ISL1	TSTA3	0.1753
FAM129A	BRIX1	TSTA3	0.1746	FAM129A	PRKACA	TSTA3	0.1753
CA5B	PECAM1	TSTA3	0.1746	ISL1	TNFRSF10A	TSTA3	0.1754
ITGAM	ANGPTL4	RELA	0.1746	POR	SLC31A1	SEPTIN4	0.1754
FAM129A	TGM2	TSTA3	0.1746	POR	HOXA9	NFKB1	0.1754
CCND1	NFKB1	TSTA3	0.1746	DCN	TRAFD1	TSTA3	0.1754
FGFR1	SEPTIN4	TSTA3	0.1746	DAB2IP	NFKB1	TSTA3	0.1754
MYC	ANGPTL4	RELA	0.1746	PRKACA	TRAFD1	TSTA3	0.1754
DOT1L	NFKB1	TSTA3	0.1746	SLC10A2	ALPI	SPDEF	0.1754
POR	BRIX1	NFKB1	0.1747	DDR2	NFKB1	TSTA3	0.1754
GJC1	NOD2	RELA	0.1747	GJC1	POR	GJB1	0.1754
PLA2G10	HOXA13	POU2F3	0.1747	GPX2	PLA2G10	CHAC1	0.1754
FOXP4	ANGPTL4	TSTA3	0.1747	ALPI	HOXA13	KCTD17	0.1754
PLA2G10	LTBP1	SLC2A13	0.1748	NFKB1	TNF	TRAFD1	0.1755
HOXA13	KCTD17	MAPK13	0.1748	EPHB4	RELA	WNT5A	0.1755
COL1A2	CTDSPL	DDIT4	0.1748	ISL1	SAT2	TSTA3	0.1755
ISL1	TSTA3	WNT5A	0.1748	FAM129A	RAD52	TLR3	0.1755
SEPTIN4	TLR5	TSTA3	0.1749	APC	SLC1A4	TSTA3	0.1756
SLC34A2	TRAFD1	TSTA3	0.1749	CDK8	HOXA13	KCTD17	0.1756
APC	DDIT4	TSTA3	0.1749	LEF1	POR	NFKB1	0.1756
RELA	SEPTIN4	TLR2	0.1749	ISL1	NANOG	TSTA3	0.1756
PLA2G10	ALCAM	FUT2	0.1749	PLA2G10	FOXD2	SLC2A13	0.1756
PLA2G10	HOXA13	MYO9B	0.1750	ISL1	CACNB4	TSTA3	0.1756
LDHB	SEPTIN4	TSTA3	0.1750	SST	HOXA13	SPDEF	0.1756
CDH3	ANGPTL4	TSTA3	0.1750	NFKB1	PROX1	TRAFD1	0.1757
SLC2A10	NFKB1	TSTA3	0.1750	COL1A2	NFKB1	RELA	0.1757
GJB1	IGF2BP1	RELA	0.1750	FOXP4	TRAFD1	TSTA3	0.1757
FGFR2	SEPTIN4	TSTA3	0.1750	LEF1	RELA	TLR2	0.1758
PTK7	RELA	TLR2	0.1751	XIST	SEPTIN4	TSTA3	0.1758
POR	NFKB1	NR2C2	0.1751	PLA2G10	HOXA9	NR5A2	0.1758
WNT4	ANGPTL4	TSTA3	0.1751	APC	PDE4C	TSTA3	0.1758
PLA2G10	FOXK2	SLC2A13	0.1751	ISL1	SLC34A2	TSTA3	0.1759
PECAM1	PPAP2B	TSTA3	0.1751	NFKB1	TLR3	TSTA3	0.1759
WNT4	RELA	WNT5A	0.1751	SEPTIN4	TLR3	TSTA3	0.1759
GEMIN6	SEPTIN4	TSTA3	0.1752	SST	CDC25B	SPDEF	0.1759
POR	SLC5A1	SEPTIN4	0.1752	DCN	SEPTIN4	TSTA3	0.1759
CTDSPL	DDIT4	TLR2	0.1752	POR	NFKB1	RAD52	0.1759
HOXA13	MAML1	SPDEF	0.1752	PLA2G10	SLC7A7	ULK1	0.1759

Gene 1	Gene 2	Gene 3	bresub error	Gene 1	Gene 2	Gene 3	bresub error
BRIX1	DDIT4	TSTA3	0.1759	HOXB8	NOD2	TSTA3	0.1764
PLA2G10	CYP4F3	ULK1	0.1759	BRIX1	DDIT4	RPSA	0.1764
CDK7	NFKB1	TSTA3	0.1759	NFKB1	PDE4C	TSTA3	0.1764
PLCE1	NFKB1	TSTA3	0.1760	PPAP2A	SEPTIN4	TSTA3	0.1764
PLA2G10	HOXA10	ULK1	0.1760	SEPTIN4	SIRT1	TSTA3	0.1764
SEPTIN4	SLC5A3	TSTA3	0.1760	PECAM1	TRAFD1	TSTA3	0.1764
NFKB2	TRAFD1	TSTA3	0.1760	GEMIN6	NFKB2	TSTA3	0.1765
FGFR1	LPAR4	TSTA3	0.1760	KDM4A	PLA2G10	ULK1	0.1765
ANGPTL4	GJB1	RELA	0.1760	POR	RAD52	SEPTIN4	0.1765
ANGPTL4	NOTCH3	RELA	0.1760	GJC1	SEPTIN4	TSTA3	0.1765
SLC39A8	HOXA13	KCTD17	0.1760	ISL1	IGF2BP1	TSTA3	0.1765
LEF1	DDIT4	TSTA3	0.1760	CDH5	SEPTIN4	TSTA3	0.1766
CACNA1C	LEF1	NFKB1	0.1761	CHGA	SEPTIN4	TSTA3	0.1766
NOD2	POR	ANGPTL4	0.1761	GPX2	PLA2G10	SLCO2A1	0.1766
ITGAM	PECAM1	TSTA3	0.1761	ANGPTL4	NANOG	RELA	0.1766
POR	NFKB1	REG1B	0.1761	POR	NFKB1	SLC1A4	0.1766
NFKB1	TNFRSF10A	TSTA3	0.1761	ID3	SEPTIN4	TSTA3	0.1766
MMP1	NFKB1	TSTA3	0.1761	FAM129A	PPAP2B	TSTA3	0.1766
PLA2G10	PRKCE	SLCO2A1	0.1761	BTD	PECAM1	TSTA3	0.1766
BCL2L1	NFKB1	TSTA3	0.1761	IGF2BP1	PECAM1	TSTA3	0.1766
FGFR1	PPAP2B	TSTA3	0.1762	POR	PRKACA	NFKB1	0.1766
POR	CDH5	SEPTIN4	0.1762	BRIX1	SEPTIN4	TSTA3	0.1766
ANGPTL4	SEPTIN4	TFAP2C	0.1762	NOD2	POR	GJB1	0.1766
PLA2G10	POU2F3	ULK1	0.1762	POR	MALL	PECAM1	0.1767
FGFR1	PTK7	TSTA3	0.1762	ANGPTL4	KCNH2	RELA	0.1767
NFKB2	WNT4	TSTA3	0.1763	ACSL4	ISL1	TSTA3	0.1767
CEACAM19	SEPTIN4	TSTA3	0.1763	PLA2G10	BCL2L13	SLC2A13	0.1767
LEF1	COL1A2	NFKB1	0.1763	MAPK7	RELA	TSTA3	0.1767
NEU3	SEPTIN4	TSTA3	0.1763	SLC25A36	SEPTIN4	TSTA3	0.1768
PLA2G10	HOXA13	JAK2	0.1763	PLA2G10	GCLM	HOXA13	0.1768
POR	SLC34A2	SEPTIN4	0.1763	MSI1	PLA2G10	CHAC1	0.1768
CACNA1C	FAM129A	SEPTIN4	0.1763	SEPTIN4	TSTA3	ZNF365	0.1768
CACNA2D1	SEPTIN4	TSTA3	0.1763	PPAP2A	ANGPTL4	RELA	0.1768
SLC5A9	NFKB1	TRAFD1	0.1763	APC	IGF2BP1	TSTA3	0.1768
HEY1	SEPTIN4	TSTA3	0.1763	PLCB4	PECAM1	TSTA3	0.1768
ISL1	PYGO2	TSTA3	0.1764	NOS1	POR	NFKB1	0.1769
CDK8	PLA2G10	CHAC1	0.1764	PLA2G10	HMGCR	HOXA13	0.1769
CGNL1	RELA	TLR2	0.1764	DOT1L	FAM129A	TSTA3	0.1769
ITGB2	CDC25B	IER3	0.1764	NFKB1	REG1B	TRAFD1	0.1769
NFKB1	OGG1	TIMP3	0.1764	DDIT4	PROX1	RPSA	0.1769
FOXP4	GEMIN6	TSTA3	0.1764	ISL1	IL23R	TSTA3	0.1769

Gene 1	Gene 2	Gene 3	bresub error	Gene 1	Gene 2	Gene 3	bresub error
HOXB8	POR	ANGPTL4	0.1769	MALL	RELA	TSTA3	0.1769
APC	WNT4	TSTA3	0.1769	WNT4	TSTA3	WNT5A	0.1769
FAM129A	ISL1	TSTA3	0.1769	PRKCH	NFKB1	TLR3	0.1770
CTDSPL	NFKB1	TIMP3	0.1769	POR	NFKB1	SLC26A6	0.1770
FAM129A	DDX11	TSTA3	0.1769	PLA2G10	CHAC1	FOXD2	0.1770
WNT4	PECAM1	TSTA3	0.1769	DDIT4	SEPTIN4	TSTA3	0.1770
LEF1	NFKB1	TIMP3	0.1769	PLA2G10	PLAU	HOXA13	0.1770

Table S14. Frequencies of Top 500 Three-Gene Classifiers. Frequencies of genes participating in the top 500 three-gene linear discriminant analysis classifiers supplement vs placebo.

Gene	Low ENL	High ENL
ABCC1	0	1
ABCG5	0	1
ACADS	0	2
ACSL4	2	4
ACTA2	1	0
ACTG2	1	0
ADAM17	0	2
AIFM2	0	3
ALCAM	1	0
ALOX15	0	2
ALPI	6	2
ANGPTL4	48	0
APC	15	0
APEX1	0	1
AQP11	0	4
AQP7	0	2
BAK1	0	3
BCL2	1	7
BCL2L1	1	0
BCL2L13	2	0
BCL2L14	0	5
BDKRB2	0	1
BIRC5	0	2
BMF	0	2
BMI1	0	2
BMP4	2	3
BRIX1	9	1
BST1	1	4
BTD	2	1
C11orf30	1	0
CA14	0	4
CA5B	1	0
CACNA1C	5	0
CACNA2D1	3	1
CACNB4	3	0
CAV1	0	1
CCDC80	0	4
CCL15	0	1
CCND1	2	1
CDC25B	3	0
CDCA7	0	4
CDH3	5	0
CDH5	3	0
CDK2AP1	0	1
CDK7	2	0
CDK8	7	0
CDKAL1	0	1

Gene	Low ENL	High ENL
CDKN1C	0	8
CEACAM19	1	3
CEACAM3	0	1
CGNL1	2	0
CHAC1	6	0
CHGA	1	0
COL1A2	11	0
COL3A1	0	3
COX4I1	0	17
CSPG5	1	1
CTDSPL	8	1
CYP4F3	3	65
DAB2IP	1	0
DAPK3	0	4
DCN	4	0
DDIT4	18	0
DDR2	1	0
DDX11	1	1
DEFA5	0	5
DEFA6	1	1
DFFA	0	2
DHFR	0	1
DLL1	0	46
DOT1L	3	1
DPEP1	0	7
DPP4	0	53
DPYSL3	0	1
DUT	0	6
ELP3	0	9
EPHB1	0	5
EPHB4	3	4
EPHB6	0	4
ERBB4	0	1
ESR1	0	2
FAM129A	25	0
FFAR4	3	0
FGF18	0	4
FGF5	0	1
FGFR1	15	2
FGFR2	1	0
FGFR3	0	3
FN1	2	0
FOXA1	0	15
FOXD2	7	0
FOXJ2	0	6
FOXK2	6	5
FOXO3	2	2

Gene	Low ENL	High ENL
FOXP4	10	1
FPR1	0	3
FUT2	1	0
GABRB3	0	5
GCLM	1	0
GCM1	0	37
GEMIN6	9	2
GJB1	13	1
GJC1	5	1
GPX1	0	3
GPX2	6	0
GRB7	1	7
GSTA1	0	3
HAS2	0	2
HEY1	1	1
HMGCL	1	0
HMGCR	1	0
HMGCS1	0	2
HOXA10	3	0
HOXA13	46	0
HOXA2	0	2
HOXA9	5	1
HOXB8	4	0
HOXC6	1	1
HOXD1	0	2
HOXD13	2	0
HOXD8	0	2
HSPD1	0	2
ID3	1	1
IER3	5	1
IFNAR1	0	1
IGF1	0	4
IGF1R	0	1
IGF2BP1	7	0
IL1R2	1	0
IL23R	1	0
IL36RN	0	7
IL4R	1	0
ILKAP	0	3
IRF8	0	1
ISL1	29	3
ITGAL	1	2
ITGAM	3	0
ITGB1	0	4
ITGB2	4	4
JAK2	4	1
KCNH2	2	0

Gene	Low ENL	High ENL
KCTD12	0	114
KCTD17	16	0
KDM4A	1	1
KDM4B	5	0
KIT	1	1
KLK6	0	2
LCT	0	3
LDHB	1	0
LEF1	11	4
LPAR1	1	7
LPAR4	4	0
LRIG1	0	4
LTBP1	1	0
MALL	4	1
MAML1	17	1
MAPK13	1	36
MAPK14	0	3
MAPK7	2	0
MAPK8	0	1
MEN1	0	9
MLH1	1	0
MMP1	1	145
MMP9	0	2
MSH3	2	0
MSI1	5	2
MYB	0	1
MYC	1	0
MYO9B	3	0
NAIP	0	1
NANOG	7	0
NEU3	1	0
NFKB1	78	0
NFKB2	8	1
NOD2	10	0
NOG	0	1
NOS1	7	0
NOS2	1	10
NOTCH3	1	0
NOTCH4	0	1
NPAT	0	4
NR2C2	7	2
NR5A2	5	0
NR6A1	0	21
NRCAM	0	10
ODC1	0	4
OGG1	3	1
OTUB1	0	13

Gene	Low ENL	High ENL
PARD3	0	2
PAX6	0	4
PCDH7	0	1
PCNA	0	2
PDE4C	3	0
PDK1	0	1
PECAM1	33	5
PIK3CA	2	0
PLA2G10	83	0
PLA2G7	0	2
PLAU	1	0
PLCB4	4	0
PLCE1	1	0
PLD3	0	8
PLS3	0	37
POLB	2	0
POR	44	1
POU2F3	3	1
PPAP2A	2	1
PPAP2B	6	0
PPARD	0	1
PPM1D	1	1
PRKACA	5	1
PRKAR1B	0	1
PRKCE	7	0
PRKCH	3	1
PRNP	0	3
PROX1	7	3
PTAFR	3	1
PTGFRN	1	25
PTGIR	0	278
PTK2	0	1
PTK7	8	2
PTPN11	0	1
PTPN22	2	4
PYGO2	1	0
RAD52	4	0
RASSF1	0	2
RBBP9	0	1
RBL1	0	1
RBL2	0	19
RBP4	1	5
RCN3	0	1
REG1A	0	11
REG1B	2	0
REL	2	2
RELA	80	1

Gene	Low ENL	High ENL
REST	6	0
RPSA	10	0
SAT2	2	1
SEPTIN4	89	0
SIGIRR	0	1
SIRT1	1	0
SLC10A2	2	1
SLC16A4	2	0
SLC17A5	0	1
SLC1A4	6	0
SLC23A3	0	1
SLC25A13	0	1
SLC25A28	0	2
SLC25A36	1	0
SLC26A6	1	0
SLC27A2	1	1
SLC28A1	0	14
SLC2A10	1	0
SLC2A13	10	1
SLC2A5	0	2
SLC31A1	3	0
SLC34A2	6	4
SLC35A2	0	1
SLC35B3	0	1
SLC39A4	0	26
SLC39A5	1	7
SLC39A8	1	0
SLC3A1	0	4
SLC5A1	2	0
SLC5A3	1	0
SLC5A9	1	2
SLC6A20	2	0
SLC7A7	2	1
SLC9A2	0	13
SLCO2A1	3	0
SLFN5	0	3
SORD	0	1
SPDEF	14	14
SPI1	0	2
SST	6	3
TAT	0	2
TBRG1	0	1
TFAP2C	3	14
TFCP2L1	0	1
TGFB2	3	1
TGM2	1	1
TIMP3	5	0

Gene	Low ENL	High ENL
TLR2	10	1
TLR3	9	0
TLR5	3	1
TNF	1	2
TNFRSF10A	4	5
TNFRSF10B	0	2
TNFRSF10D	0	2
TNK2	1	0
TP53	1	0
TP63	1	1
TRAFD1	44	5
TREH	1	0
TSC2	1	5
TSTA3	252	0
UHRF1	0	1
ULK1	18	0
VDR	0	21
VNN1	1	0
WNT4	16	3
WNT5A	9	5
XIST	1	5
ZBTB33	1	0
ZNF365	0	0
